# *ENHANCING CTR1-10 ETHYLENE RESPONSE2* is a novel allele involved in CONSTITUTIVE TRIPLE-RESPONSE1-mediated ethylene receptor signaling in Arabidopsis

**DOI:** 10.1186/1471-2229-14-48

**Published:** 2014-02-15

**Authors:** Aibei Xu, Wei Zhang, Chi-Kuang Wen

**Affiliations:** 1 National Key Laboratory of Plant Molecular Genetics and National Center for Plant Gene Research (Shanghai), Institute of Plant Physiology and Ecology, Shanghai Institutes for Biological Sciences, Chinese Academy of Sciences, 300 Fenglin Rd, Shanghai 200032, China

**Keywords:** Arabidopsis, Ethylene signaling, CTR1, ETR1, ECR2

## Abstract

**Background:**

The signal output of ethylene receptor family members is mediated by unknown mechanisms to activate the Raf-like protein CONSTITUTIVE TRIPLE RESPONSE1 (CTR1) in negatively regulating ethylene signaling. The physical interaction between the ethylene receptor histidine kinase (HK) domain and CTR1 N terminus is essential to the CTR1-mediated receptor signal output. To advance our knowledge of the involvement of CTR1-mediated ethylene receptor signaling, we performed a genetic screen for mutations that enhanced the constitutive ethylene response in the weak *ctr1-10* allele.

**Results:**

We isolated a loss-of-function allele of *ENHANCING ctr1-10 ETHYLENE RESPONSE2* (*ECR2*) and found that *ecr2-1 ctr1-10* and the strong allele *ctr1-1* conferred a similar, typical constitutive ethylene response phenotype. Genetic analyses and transformation studies suggested that ECR2 acts downstream of the ethylene receptors and upstream of the transcription factors ETHYLENE INSENSITIVE3 (EIN3) and EIN3-LIKE1 (EIL1), which direct the expression of ethylene response genes. Signal output by the N terminus of the ethylene receptor ETHYLENE RESPONSE1 (ETR1) can be mediated by a pathway independent of CTR1. Expression of the N terminus of the ethylene-insensitive etr1-1 but not the full-length isoform rescued the *ecr2-1 ctr1-10* phenotype, which indicates the involvement of ECR2 in CTR1-mediated but not -independent, ethylene receptor signaling. *ECR2* was mapped to the centromere region on chromosome 2. With incomplete sequence and annotation information and rare chromosome recombination events in this region, the cloning of *ECR2* is challenging and still in progress.

**Conclusions:**

*ECR2* is a novel allele involved in the ethylene receptor signaling that is mediated by CTR1. CTR1 activation by ethylene receptors may require ECR2 for suppressing the ethylene response.

## Background

Ethylene is a gaseous hormone regulating many aspects of plant growth and development. The dicotyledonous model plant Arabidopsis has five ethylene receptors that physically act at the endoplasmic reticulum (ER) with the Raf-like protein CONSTITUTIVE TRIPLE-RESPONSE1 (CTR1) to negatively regulate ethylene signaling [1-4]. In the absence of ethylene, the receptor signal output is mediated by an unknown mechanism(s) to activate CTR1, and CTR1 is presumably activated to phosphorylate the downstream signaling component ETHYLENE INSENSITIVE2 (EIN2). Phosphorylated EIN2 stays at the ER and cannot induce the ethylene response. With ethylene binding to ethylene receptors, the receptor signal output is prevented and CTR1 is not activated. Unphosphorylated EIN2 undergoes proteolytic cleavage by an unknown mechanism to produce a C-terminal fragment, which enters the nucleus to induce the ethylene response [5,6]. Targets of the EIN2 C terminus remain to be identified. The transcription factors EIN3 and EIN3-LIKE1 (EIL1) direct the expression of ethylene-responsive genes [7], and EIN2 C-terminus–induced ethylene responses are prevented in the *ein3-1* loss-of-function mutant [6]. Conceivably, the EIN2 C terminus could mediate the ethylene signaling to EIN3 and EIL1.

The ethylene receptors are structurally similar to histidine kinases (HKs) of prokaryotic two-component modules, and studies have revealed the domain functions of the ethylene receptors. The N terminus has three or four transmembrane domains (TMs) that bind a copper cofactor for ethylene binding and are required for localization at the ER [8-11]. Following the TMs is the GAF domain for non-covalent receptor interaction to mediate inter-receptor signaling [12-14]. The C-terminal portion is the HK domain, which is believed to function in the receptor signal output via direct interaction with the CTR1 N terminus [2,4]. CTR1 has serine/threonine kinase activity, and the ethylene response is inversely associated with CTR1 kinase activity [4,15]. These studies suggest that the HK domain mediates ethylene receptor signaling to the CTR1 N terminus, thus activating CTR1 to suppress the ethylene response, although the underlying biochemical mechanisms are elusive.

Recent studies suggest that the HK domain can be dispensable to ethylene receptor signal output and that CTR1 is not the only component mediating the signaling. Mutations that delete ETHYLENE RESPONSE1 (ETR1) HK and receiver domains have little effect on the receptor signal output [13,16], and expression of the truncated etr1^1-349^ isoform that lacks the site for interacting with CTR1 rescues the *ctr1* loss-of-function mutant phenotype. Conceivably, the ETR1 receptor signal output can be mediated via the N terminus to an alternative pathway independent of CTR1 [3,14]. Of note, ETR1 receptor signaling in kinase-defective *ctr1* mutants occurs only when HK-domain–lacking ETR1 isoforms are expressed. A kinase-defective ctr1 isoform may dock at the ETR1 HK domain and actively prevent ETR1 N-terminal signaling to the CTR1-independent alternative pathway [14,17].

The *ctr1-10* mutation results from a T-DNA insertion at the 5′-untranslated region (5′-UTR) of *CTR1*[18]. The 5′-UTR of a mRNA may be highly structured and contain upstream AUGs (uAUGs) and internal ribosome entry sites that can affect the translation. With increased upstream open reading frames (uORFs) and a highly structured nature, a long 5′-UTR can be translationally inhibitory [19-23]. With a higher *CTR1* level in *ctr1-10* than in the wild type [18], the T-DNA insertion may substantially increase the 5′-UTR length of the *CTR1* transcript to affect translation efficiency, thus reducing the CTR1 level and thus activity as compared with the wild type.

The underlying biochemical mechanisms of CTR1 activation by the ethylene receptors remain to be unraveled. Mutations that enhance the constitutive ethylene response in *ctr1-10* could have a role involving CTR1 activation, protein stability or ethylene signaling. Here we report that the loss-of-function mutation of *ENHANCING ctr1-10 ETHYLENE RESPONSE2* (*ECR2*) enhanced the ethylene response in *ctr1-10* comparable to the strong allele *ctr1-1* and the ethylene-treated wild type. Results from extensive genetic and transformation studies suggested that ECR2 acts downstream of the ethylene receptors and upstream of the positive ethylene response regulators EIN3 and EIL1. We discuss possible roles for ECR2 in the negative regulation of the ethylene response.

## Results

### *ctr1-10* is a weak allele

The *ctr1-10* mutation results from a T-DNA insertion at the 5′-UTR, and the mutant shows a weak constitutive ethylene response phenotype [18]. The nature of the mutation that causes the weak phenotype remains to be determined.

**Figure 1:**
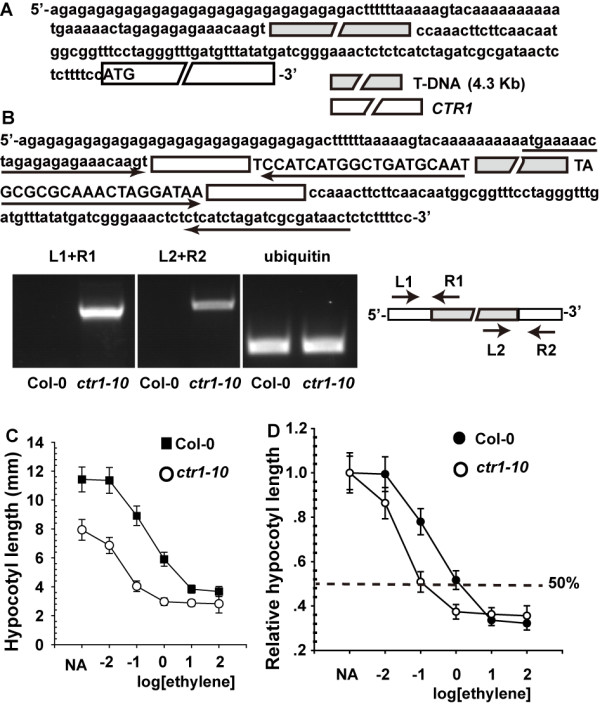
**Gene structure and ethylene-dose response assay of the mutant *constitutive triple response 1–10 *(*ctr1-10*). (A)***ctr1-10* gene structure and cDNA sequence flanking the T-DNA. The T-DNA is in gray and *CTR1* coding region white. **(B)** The T-DNA sequence is transcribed as part of the *ctr1-10* 5′-untranslated region (5′-UTR). Arrows indicate the primer orientation and corresponding cDNA sequences for RT-PCR. Capital letters are the T-DNA sequence and lowercase letters are the *CTR1* cDNA sequence. T-DNA is in gray and the 5′-UTR white. RT-PCR analysis of gene expression. Ethylene dose–response assay for hypocotyl measurement **(C)** and relative hypocotyl length **(D)** of etiolated wild-type (Col-0) and *ctr1-10* seedlings. The dotted line in **(D)** indicates 50% growth inhibition. Data are mean ± SD (*n* ≥ 20).

Given that the T-DNA inserts at the 5′-UTR, we expected that the T-DNA fragment is transcribed as part of the 5′-UTR of *ctr1-10* mRNA (Figure 1A). Indeed, RT-PCR with primers pairing the 5′-UTR and T-DNA sequences showed the presence of a chimeric cDNA fragment containing the sequence of the *ctr1-10* 5′-UTR and the T-DNA (Figure 1B). For the same gene, a longer 5′-UTR can be translationally more inhibitory than a shorter one [19,20,22,23]. With a 4.3-kb T-DNA at the 5′-UTR, we hypothesized that *ctr1-10* could produce a lower level of CTR1 than the wild type, for a weak constitutive ethylene response phenotype.

Ethylene inhibits hypocotyl growth and the ethylene response can be quantified by measurement of hypocotyls. Hypocotyls were shorter for *ctr1-10* than wild-type (Col-0) seedlings over a wide range of ethylene concentrations (Figure 1C). When growth for wild-type and *ctr1-10* seedlings are normalized to growth with no added ethylene, it becomes apparent that *ctr1-10* seedlings are more sensitive to ethylene with a shift from 1 μL L^-1^ for wild-type to 0.1 μL L^-1^ for the mutant (Figure 1D). These data suggest increased ethylene sensitivity with the *ctr1-10* mutation.

### Genetic screen for *ctr1-10* enhancer mutations

To isolate components that could be involved in CTR1-mediated ethylene receptor signaling, we performed an enhancer screen for *ctr1-10*. Alleles that enhanced the *ctr1-10* mutant phenotype were designated *enhancing ctr1-10 ethylene response* (*ecr*). Here we describe the isolation of *ecr2-1 ctr1-10*.

**Figure 2:**
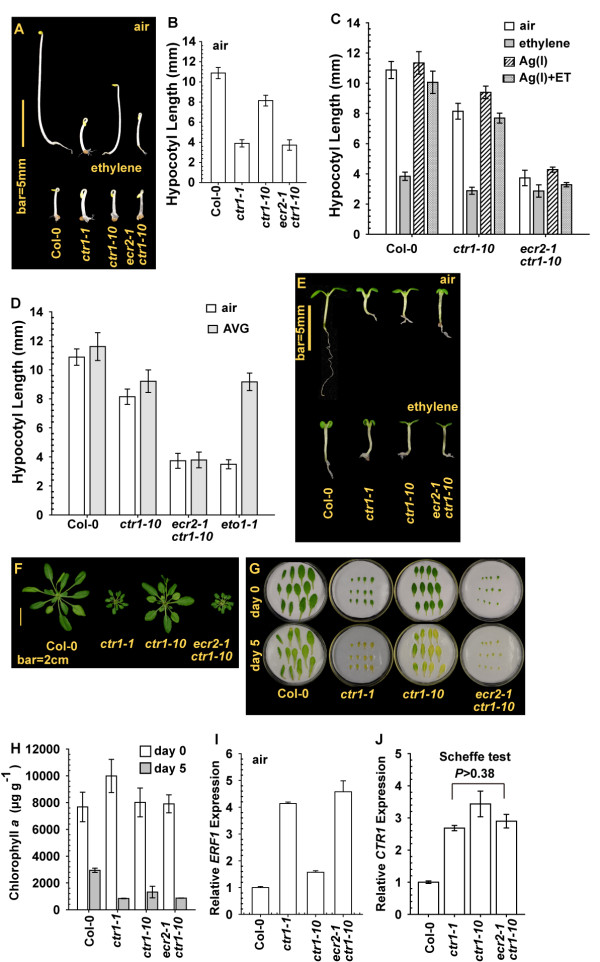
**Characterization of mutant *enhancing ctr1-10 ethylene response2-1 *(*ecr2-1*) *ctr1-10*. (A)** Phenotype of etiolated *ecr2-1 ctr1-10* seedlings, with the wild type (Col-0), *ctr1-10*, and *ctr1-1* as the control. Hypocotyl measurement of etiolated seedlings **(B)**, with treatment with silver **(C)** and aminoethoxyvinylglycine (AVG) **(D)**. Phenotype of light-grown seedlings **(E)** and rosettes **(F)**. Senescence phenotype **(G)** and chlorophyll *a* content **(H)** of leaves before (day 0) and after (day 5) detachment. Quantitative RT-PCR (qRT-PCR) analysis of *ERF1***(I)** and *CTR1***(J)** mRNA expression. Data are mean±SD for seedling hypocotyl and chlorophyll *a* content and mean±SE for gene expression.

Without ethylene treatment, etiolated seedlings of *ecr2-1 ctr1-10* and the strong allele *ctr1-1* were identical in phenotype and hypocotyl length (Scheffe test, *P* = 0.65); *ctr1-10* seedlings were longer than the two genotypes and shorter than the wild type (Col-0) (Scheffe test, *P* < 10^-57^; Figure 2A and 2B). As expected, ethylene treatment inhibited the seedling growth in the wild type (Col-0), *ctr1-10,* and *ecr2-1 ctr1-10* (Figure 2C). Of note, *ecr2-1 ctr1-10* seedlings already showed a constitutive ethylene response phenotype, with a short hypocotyl, so that the effect of ethylene on the seedling growth inhibition was minor (by 0.80458±0.1776 mm; 99% confidence level).

The silver ion Ag(I) may bind ETR1 and prevent ethylene inhibition of ETR1 receptor signaling [11,24]. Silver treatment largely prevented ethylene-induced inhibition of seedling hypocotyl growth in wild-type (Col-0) and *ctr1-10* but not *ecr2-1 ctr1-10* seedlings (Figure 2C). Of note, the seedling hypocotyls were longer for silver-treated than non-silver–treated *ecr2-1 ctr1-10* seedlings (Student’s *t* test, *P* < 0.01), regardless of ethylene treatment; the difference was minor and could be of little biological significance. These results suggest that ETR1 receptor signaling that was mediated in part in *ctr1-10* was prevented in *ecr2-1 ctr1-10*.

Aminoethoxyvinylglycine (AVG) prevents the biosynthesis of the immediate ethylene biosynthesis precursor 1-aminocyclopropane-1-carboxylic (ACC), and the treatment can reduce endogenous ethylene production [25]. AVG (10 μM) treatment did not rescue the reduced growth of *ecr2-1 ctr1-10* seedlings, so *ecr2-1* did not enhance the production of ethylene to cause reduced growth (Figure 2D). ETHYLENE OVERPRODUCER1 (ETO1) is a negative regulator of the ACC-synthesing enzyme ACC SYNTHASE5 (ACS5) and the *eto1-1* allele produces a higher level of ACC, and therefore ethylene, than does the wild type [26,27]. As a control, *eto1-1* seedlings showed a short hypocotyl, and growth was rescued with AVG treatment (Figure 2D); AVG treatment in this study was sufficient to prevent endogenous ethylene production.

We examined the growth inhibition phenotype at other developmental stages. Grown under light, ethylene treatment inhibited cotyledon expansion and root elongation in wild-type (Col-0) seedlings. Seedlings of *ctr1-1*, *ctr1-10*, and *ecr2-1 ctr1-10* showed the growth inhibition phenotype regardless of ethylene treatment (Figure 2E). At the adult stage, rosettes were larger for the wild type (Col-0) than the three mutants, with *ctr1-1* and *ecr2-1 ctr1-10* being phenotypically similar and both producing a smaller rosette than *ctr1-10* (Figure 2F).

We examined alterations in other aspects of the ethylene response. Leaves of the wild type, *ctr1-1*, *ctr1-10*, and *ecr2-1 ctr1-10* showed the senescence phenotype to various degrees 5 days after detachment (Figure 2G). Detached leaves of the mutants showed a similar chlorophyll *a* level, with a similar or slightly higher level in *ctr1-10* than *ctr1-1* and *ecr2-1 ctr1-10* (Scheffe test, *P* = 0.075 and 0.03, respectively) (Figure 2H). *ETHYLENE RESPONSE FACTOR1* (*ERF1*) expression is directed by EIN3 and the expression is associated with degrees of ethylene response [7,28]. *ERF1* expression was slightly higher in *ctr1-10* than the wild type and highly induced in *ctr1-1* and *ecr2-1 ctr1-10* without ethylene treatment (Figure 2I). *CTR1* levels in *ctr1-1*, *ctr1-10*, and *ecr2-1 ctr1-10* were identical (Scheffe test, *P* > 0.38) and higher than in the wild type (Figure 2J). The *ecr2-1* allele did not affect *CTR1* expression in *ctr1-10*.

### *ecr2-1* is a recessive, loss-of-function mutation

**Figure 3:**
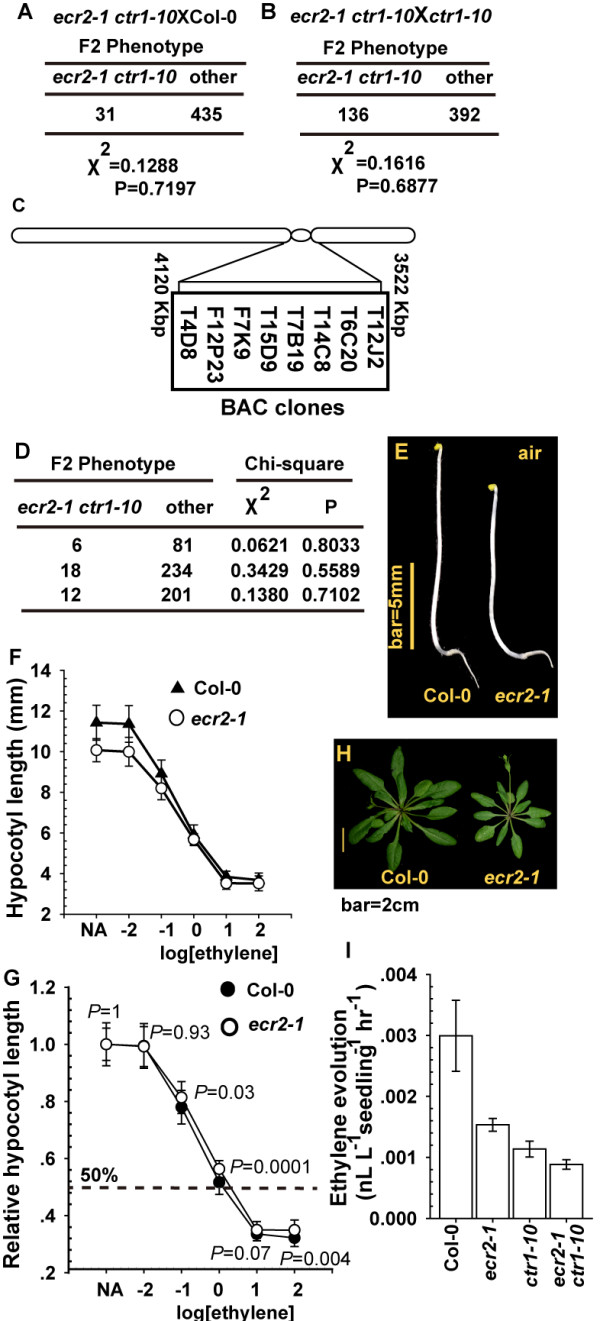
**Isolation of *ecr2-1*. (A)** and **(B)** Genetic analyses and chi-square test of *ecr2-1*. Numbers are individuals scored in the F2 generation. Other: other phenotype that is not *ecr2-1 ctr1-10* growth inhibition phenotype. **(C)***ECR2* is mapped to a region spanning the centromere at chromosome 2, and the BAC clones are indicated. **(D)** Results for 3 independent test crosses for *ecr2-1* with *ctr1-10*. Phenotype **(E)** and ethylene dose–response assay for the hypocotyl measurement **(F)** and relative hypocotyl length **(G)** of etiolated wild-type (Col-0) and *ecr2-1* seedlings. The dotted line indicates 50% growth inhibition for **(G)**; *P* values for Student’s *t* test are indicated. **(H)** Rosette phenotype of *ecr2-1*. **(I)** Ethylene evolution of light-grown seedlings (5 days after germination). Data are mean±SD of 3 independent measurements.

To genetically evaluate whether the effect of the *ecr2-1* mutation on the *ctr1-10* mutant phenotype is associated with single or multiple alleles, we crossed *ecr2-1 ctr1-10* with the wild type (Col-0) and *ctr1-10*. In the F2 (filial) generation of the wild type (Col-0) cross, 466 individuals were scored, and only 31 showed the *ecr2-1 ctr1-10* growth inhibition phenotype (Figure 3A; segregation ratio = 1:15). In the F2 generation of the *ctr1-10* cross, 528 individuals were scored, and 136 showed the *ecr2-1 ctr1-10* growth inhibition phenotype (Figure 3B; segregation ratio = 1:3). Results from both genetic analyses suggested that the *ecr2-1* loss-of-function mutation was recessive and intergenic to *ctr1-10*. The independent segregation of *ecr2-1* and *ctr1-10* suggested that *ECR2* and *CTR1* are in distinct linkage groups.

We attempted to clone *ECR2* by map-based cloning and mapped *ECR2* to a 598-kb region spanning the centromere at chromosome 2 (Figure 3C), which agreed with *ECR2* and *CTR1* (chromosome 5) being in distinct linkage groups. However, we did not obtain more recombinants to advance the mapping to a narrower region. This region contains sequence gaps (indicated as [gapbp]ExpandNs or a string of Ns in Additional file 1) and fragments with repeated sequences, retrotransposon, transposase, and transposable elements. We sequenced 39 annotated genes (excluding those annotated as retrotransposon, transposase, and transposable elements) but did not identify any mutation (Additional file 1). Among the genes, T-DNA insertion mutants for At2g07981and At2g08986 were each genetically crossed with *ctr1-10* to test whether any of these was *ECR2*, and these mutations did not enhance the *ctr1-10* phenotype.

The mutation that affects *ECR2* remained to be identified and we attempted to isolate the *ecr2-1* single mutant. The F2 seedlings from the genetic crossing of *ecr2-1 ctr1-10* and the wild type (Col-0) were crossed with *ctr1-10*, and the individual that produced the *ecr2-1 ctr1-10* mutant phenotype in the F2 generation with a 1:15 segregation ratio was considered the *ecr2-1* mutant. We isolated *ecr2-1*, and results from 3 independent crossings with *ctr1-10* showed a 1:15 segregation (Figure 3D). Grown in dark, *ecr2-1* seedlings produced a shorter hypocotyl than did the wild type (Col-0) at low ethylene concentrations (Figure 3E and 3F); without ethylene treatment, the height of *ecr2-1* seedlings was 1.355±0.62 mm (99% confidence level) shorter than that of wild-type seedlings. When this data was normalized, we found that wild-type and *ecr2-1* seedlings had indistinguishable sensitivity to ethylene (Figure 3G). Although the relative hypocotyl length was longer (by 4.6±2.9%; 99% confidence level) for *ecr2-1* seedlings than wild-type seedlings with 1 μL L^-1^ ethylene, the difference was small and could be of little biological significance. At the adult stage, the rosette was smaller for *ecr2-1* than the wild type (Figure 3H). We excluded the possibility that the mutant phenotype of *ecr2-1* and *ecr2-1 ctr1-10* could be due to greater ethylene evolution than the wild type (Col-0), as supported by ethylene evolution (Figure 3I).

These results suggest that the *ecr2-1* allele conferred a minor growth inhibition throughout development. As compared with the wild type (Col-0), etiolated *ecr2-1* seedlings showed no alteration in ethylene sensitivity.

### CTR1-independent but not -dependent ETR1 receptor signaling occurs in *ecr2-1 ctr1-10*

Silver treatment prevents the inhibition of ETR1 receptor signaling by ethylene [13,24]. Silver treatment reversed the ethylene effect on growth of *ctr1-10* but not *ecr2-1 ctr1-10* seedlings (Figure 2C), so ETR1 receptor signaling was prevented in *ecr2-1 ctr1-10* but partly mediated in *ctr1-10*. We evaluated whether ETR1 receptor signaling could occur in *ecr2-1 ctr1-10*.

**Figure 4:**
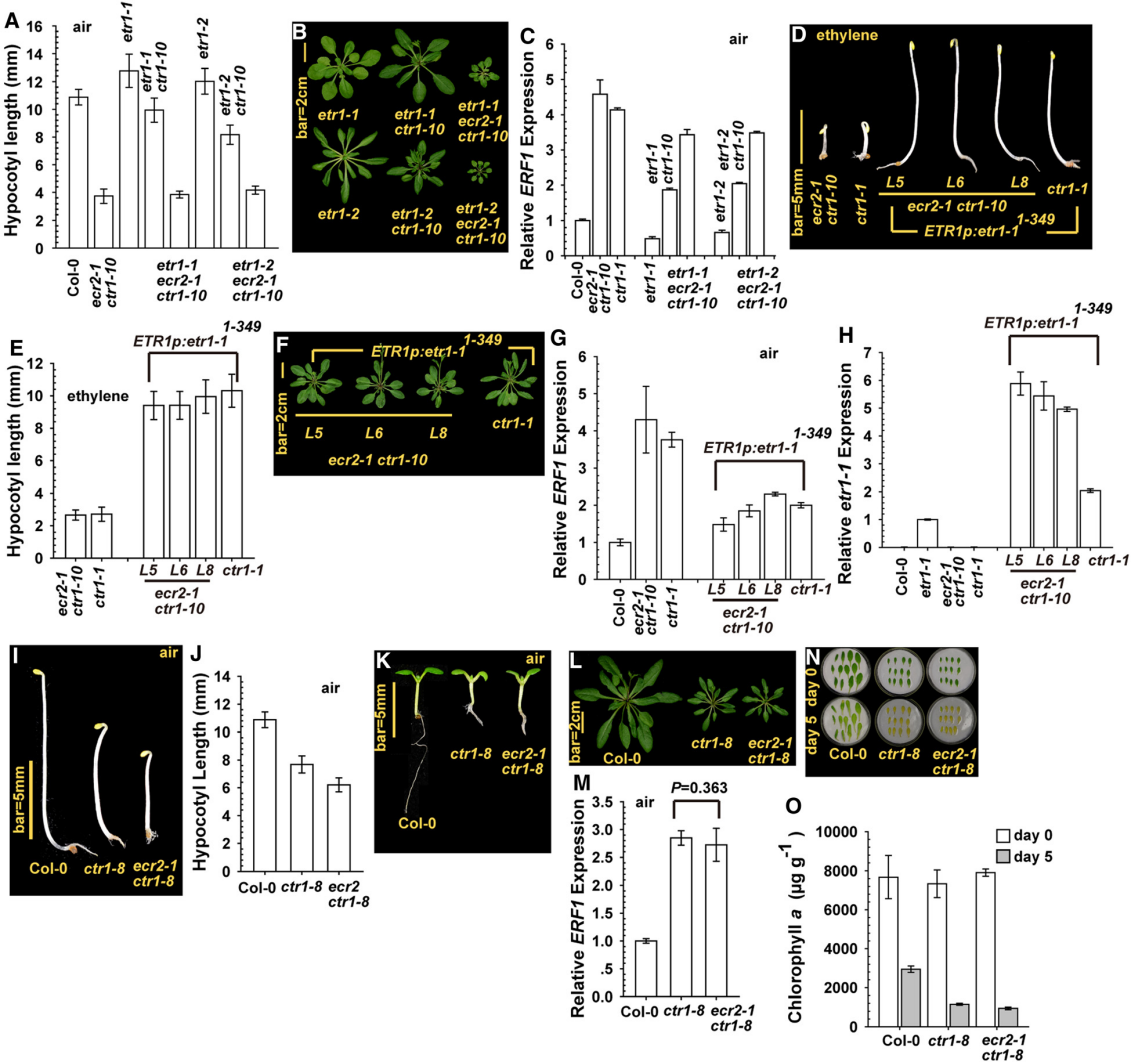
**ECR2 is involved in the CTR1-dependent but not -independent ethylene receptor signaling.** Etiolated seedling hypocotyl measurement **(A)**, rosette phenotype **(B)**, and *ERF1* expression **(C)** of *ecr2-1 ctr1-10* with and without the ethylene-insensitive *etr1-1* or *etr1-2* allele. Etiolated seedling phenotype **(D)**, hypocotyl measurement **(E)**, rosette phenotype **(F)** and *ERF1* mRNA expression **(G)** in *ecr2-1 ctr1-10* lines expressing *ETR1p:etr1-1*^*1-349*^. **(H)***etr1-1* mRNA levels in *etr1-1* as the control and *etr1-1*^*1-349*^ levels in *ecr2-1 ctr1-10 ETR1p:etr1-1*^*1-349*^ lines, respectively. Etiolated seedling phenotype **(I)** and hypocotyl measurement **(J)**, light-grown seedlings **(K)**, rosette phenotype **(L)**, and *ERF1* expression **(M)** in *ecr2-1 ctr1-8*. Senescence phenotype **(N)** and chlorophyll *a* content **(O)** in *ecr2-1 ctr1-8* before (day 0) and after (day 5) the test. Data are mean ± SD for the seedling hypocotyl measurement and chlorophyll *a* content, and mean ± SE for gene expression.

Ethylene-insensitive etr1-1 and etr1-2 mediate receptor signaling by different mechanisms: the former does not require REVERSION-TO-ETHYLENE SENSITIVITY1 (RTE1), whereas the latter does [29,30]. Because of their dominant nature, we used *etr1-1* and *etr1-2* mutants to evaluate ETR1 receptor signaling. In the absence of ethylene treatment, etiolated *etr1-1* and *etr1-2* seedlings produced a long hypocotyl, and the *ctr1-10* mutation moderately reduced the seedling hypocotyl elongation in each allele; as expected, *etr1-1 ecr2-1 ctr1-10* and *etr1-2 ecr2-1 ctr1-10* seedlings were short, with a hypocotyl length similar to *ecr2-1 ctr1-10* seedlings (Figure 4A). Consistently, at the adult stage, the rosette was larger for *etr1-1 ctr1-10* and *etr1-2 ctr1-10* than *etr1-1 ecr2-1 ctr1-10* and *etr1-2 ecr2-1 ctr1-10* plants, respectively (Figure 4B). We scored the ethylene response in these genotypes by measuring *ERF1* expression: the *ERF1* level was greater in *etr1-1 ctr1-10* and *etr1-2 ctr1-10* than the wild type and lower than in *etr1-1 ecr2-1 ctr1-10* and *etr1-2 ecr2-1 ctr1-10* (Figure 4C). ETR1 receptor signaling may be prevented by *ecr2-1 ctr1-10* but little affected by *ctr1-10*.

ETR1 receptor signaling can be mediated via the N-terminal portion independent of CTR1 [14], so we evaluated whether ECR2 could act in the CTR1-independent pathway. With ethylene treatment, *ctr1-1* seedlings expressing the *ETR1p:etr1-1*^*1-349*^ transgene that encodes the etr1-1 N terminus (residues 1–349) were ethylene insensitive and produced a long hypocotyl [14] (Figure 4D and 4E). Interestingly, the phenotype of ethylene growth inhibition in *ecr2-1 ctr1-10* was rescued by the transgene, and *ETR1p:etr1-1*^*1-349*^*ecr2-1 ctr1-10* seedlings did not show the phenotype of ethylene-inhibited growth (Figure 4D and 4E). Consistently, *ETR1p:etr1-1*^*1-349*^ expression greatly rescued rosette growth in *ecr2-1 ctr1-10* and *ctr1-1* (Figure 4F). The expression of the transgene attenuated *ERF1* levels in *ecr2-1 ctr1-10* and *ctr1-1* (Figure 4G). Expression of *ETR1p:etr1-1*^*1-349*^ in the transformation lines was confirmed by qRT-PCR with *etr1-1–*specific primers, and *etr1-1* expression was detectable only in genotypes that carried the allele or transgene (Figure 4H).

These data suggest that the *ecr2-1* allele prevented the receptor signaling with the full-length etr1-1 and etr1-2 but not etr1-1 N terminus in *ctr1-10*. Therefore, ECR2 may be involved in CTR1-dependent but not -independent receptor signaling in *ctr1-10*. Given that the signaling of full-length ETR1 but not ETR1 N terminus is prevented by kinase-defective ctr1 isoforms [14,17], ECR2 could be involved in part in CTR1 kinase activity or protein stability.

ctr1-8 is not associated with the ethylene receptors, and the mutant shows a weak constitutive ethylene response. We previously hypothesized that mediation of ethylene receptor signaling in *ctr1-8* was independent of CTR1 [4,17]. The possibility that ctr1-8 could mediate in part the receptor signaling, for a weak mutant phenotype, should be considered. Given that ECR2 is involved in CTR1-dependent ethylene receptor signaling, we expected that *ecr2-1* would have little effect on *ctr1-8* if ctr1-8 did not mediate the ethylene receptor signaling.

We crossed *ecr2-1 ctr1-10* with *ctr1-8*, and the resulting F2 plants were genotyped for the *CTR1* allele. All F2 plants that showed the typical constitutive ethylene response phenotype were *ctr1-10* or *ctr1-8 ctr1-10*, so they all carried the homozygous *ecr2-1* allele and *ecr2-1* did not enhance the *ctr1-8* mutant phenotype. The mutant *ecr2-1 ctr1-8 ctr1-10* was selfed, and the resulting progenies with the homozygous *ctr1-8* allele were *ecr2-1 ctr1-8*.

The length of etiolated *ecr2-1 ctr1-8* seedlings was only 1.4±0.43 mm (99% confidence level) shorter than that of *ctr1-8* seedlings (Figure 4I and 4J), which is in line with the result showing that the *ecr2-1* allele conferred minor seedling growth inhibition (Figure 3E and 3F) but not increased ethylene sensitivity (Figure 3G). Light-grown seedlings of *ctr1-8* and *ecr2-1 ctr1-8* were phenotypically similar, with a shorter root and smaller cotyledons than the wild type (Col-0) (Figure 4K). Consistently, the rosette was larger for the wild type than *ctr1-8* and *ecr2 ctr1-8* plants (Figure 4L). Therefore, the *ecr2-1* allele may have little effect on the ethylene response phenotype in *ctr1-8*. We quantified the ethylene response for the two mutants. *ERF1* expression was identical in *ctr1-8* and *ecr2-1 ctr1-8* (Student’s *t* test, *P* = 0.363) and higher than in the wild type (Figure 4M). Both *ctr1-8* and *ecr2-1 ctr1-8* had the same senescence phenotype, and their chlorophyll *a* content was identical before (Student’s *t* test; *P* = 0.056) and after (Student’s *t* test; *P* =0.059) the test (Figure 4N and 4O).

The *ecr2-1* allele had little effect on *ctr1-8* ethylene response, which suggests that ethylene receptor signaling in *ctr1-8* was predominantly mediated by a pathway independent of CTR1.

### Genetic analysis of effect of *ein3-1* and *eil1-1* on the ethylene response in *ecr2-1 ctr1-10*

The present data suggested the involvement of ECR2 in ethylene receptor signaling mediated by CTR1. ETHYLENE INSENSITIVE2 (EIN2) acts downstream of CTR1, and *ein2* loss-of-function mutation may rescue the *ecr2-1 ctr1-10* mutant phenotype. Genetic analyses for the effects of *ein2* on *ecr2-1 ctr1-10* were not successful because *EIN2* is tightly linked with *CTR1* and *ECR2* remains to be cloned.

**Figure 5:**
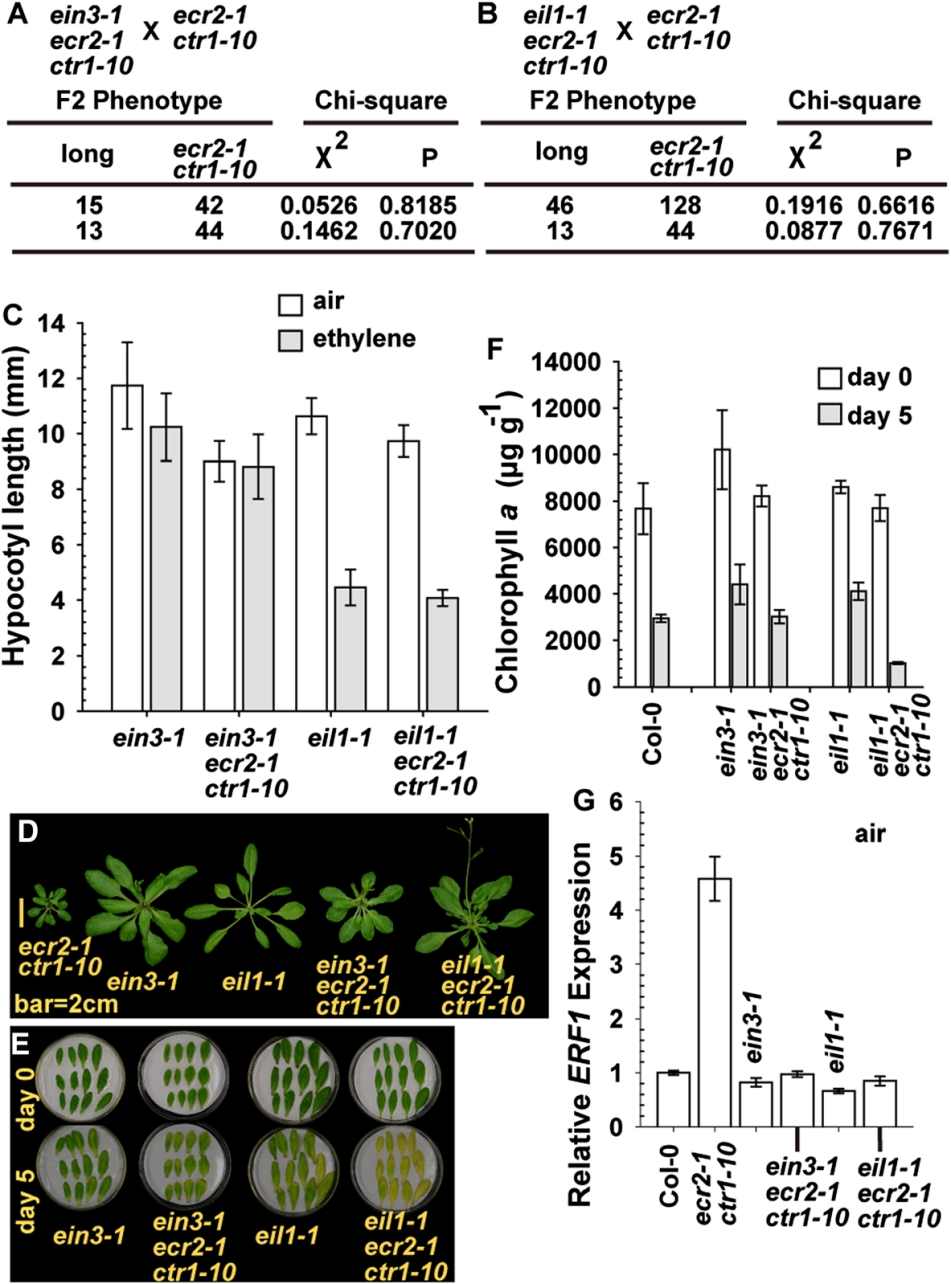
**Genetic analysis of the effect of *ethylene insensitive3-1 *(*ein3-1*) and *ein3-like 1 *(*eil1-1*) on *ecr2-1 ctr1-10*.** Segregation ratio and chi-square analysis of 2 independent genetic crossings of *ecr2-1 ctr1-10* with *ein3-1 ecr2-1 ctr1-10***(A)** and *eil1-1 ecr2-1 ctr1-10***(B)**. Etiolated seedling hypocotyl measurement **(C)**, rosette phenotype **(D)**, senescence test for detached leaves **(E)**, chlorophyll *a* content for the senescence test **(F)**, and *ERF1* expression **(G)** in *ein3-1 ecr2-1 ctr1-10* and *eil1-1 ecr2-1 ctr1-10*. Data are mean ± SD for seedling hypocotyl and chlorophyll *a* measurement, and mean ± SE for *ERF1* levels.

EIN3 and EIN3-LIKE1 (EIL1) are the transcription factors that direct the expression of ethylene-responsive genes, and *EIN3* loss-of-function mutations confer ethylene insensitivity [7,31]. We used genetic analyses to evaluate whether ECR2 acts upstream of EIN3/EIL1. To obtain *ein3-1 ecr2-1 ctr1-10* and *eil1-1 ecr2-1 ctr1-10* mutants, *ein3-1* and *eil1-1* were each genetically crossed with *ecr2-1 ctr1-10*, and the resulting F2 individuals carrying *ein3-1 ctr1-10* and *eil1-1 ctr1-10* were identified by genotyping. All F2 generations with the *ein3-1 ctr1-10* and *eil1-1 ctr1-10* genotypes showed relatively normal growth. The F2 individuals were each genetically crossed with *ecr2-1 ctr1-10* to test the presence of the *ecr2-1* allele, and the individuals that were *ein3-1 ecr2-1 ctr1-10* and *eil1-1 ecr2-1 ctr1-10* would give a 1:3 segregation (long seedlings: short seedlings) in the resulting F2 generation. Verified by chi-square test for 2 independent test crossings, we isolated *ein3-1 ecr2-1 ctr1-10* and *eil1-1 ecr2-1 ctr1-10* (Figure 5A and 5B).

Without ethylene treatment, *ein3-1 ecr2-1 ctr1-10* and *eil1-1 ecr2-1 ctr1-10* seedlings produced a long seedling hypocotyl (Figure 5C) as compared with *ecr2-1 ctr1-10* seedlings (Figure 2B). Of note, *ein3-1* but not *eil1-1* seedlings were ethylene-insensitive, and ethylene treatment inhibited *eil1-1* but not *ein3-1* seedling growth. Consistently, the *ein3-1* allele prevented ethylene-inhibited growth of seedling hypocotyls in *ecr2-1 ctr1-10* whereas *eil1-1* did not (Figure 5C). At the adult stage, *ein3-1* and *eil1-1* alleles each rescued *ecr2-1 ctr1-10* rosette growth to a great extent, and rosettes were larger for *ein3-1 ecr2-1 ctr1-10* and *eil1-1 ecr2-1 ctr1-10* than *ecr2-1 ctr1-10* plants (Figure 5D). The ethylene response in each triple mutant was quantified by leaf senescence and *ERF1* expression. The leaf senescence phenotype was weaker in *ein3-1*, *eil1-1* and *ein3-1 ecr2-1 ctr1-10* than *ecr2-1 ctr1-10* and *eil1-1 ecr2-1 ctr1-10* plants (Figures 2G and 5E), which was consistent with chlorophyll *a* content (Figures 2H and 5F). Without ethylene treatment, the *ERF1* level in *ecr2-1 ctr1-10* was highly reduced with the respective addition of *ein3-1* and *eil1-1* alleles, and *ein3-1*, *eil1-1*, *ein3-1 ecr2-1 ctr1-10* and *eil1-1 ecr2-1 ctr1-10* showed identical *ERF1* expression (Scheffe test, *P* = 0.06-0.995; Figure 5G).

The present data support that ECR2 acts in ethylene signal transduction upstream of the transcription factors EIN3 and EIL1. The *eil1-1* allele did not prevent the ethylene-inhibited seedling growth or leaf senescence in *ecr2-1 ctr1-10*, which indicates that functions of EIN3 and EIL1 differ, with a larger role for EIN3 than EIL1 in ethylene responses.

### *EBF1* and *EBF2* overexpression rescues *ecr2-1 ctr1-10* phenotype

EIN3-BINDING F-BOX1 (EBF1) and EBF2 are F-box proteins involved in the ubiquitination of EIN3 and EIL1 to mediate their degradation by the 26S proteosome [32,33]. Excess EBF1 and EBF2 reduces EIN3 and EIL1 levels and suppresses the ethylene response [34]. The argument that ECR2 acts in the ethylene signal transduction pathway upstream of the transcription factors EIN3 and EIL1 can be tested by examining whether overexpression of *EBF1* and *EBF2* rescues the *ecr2-1 ctr1-10* phenotype.

**Figure 6:**
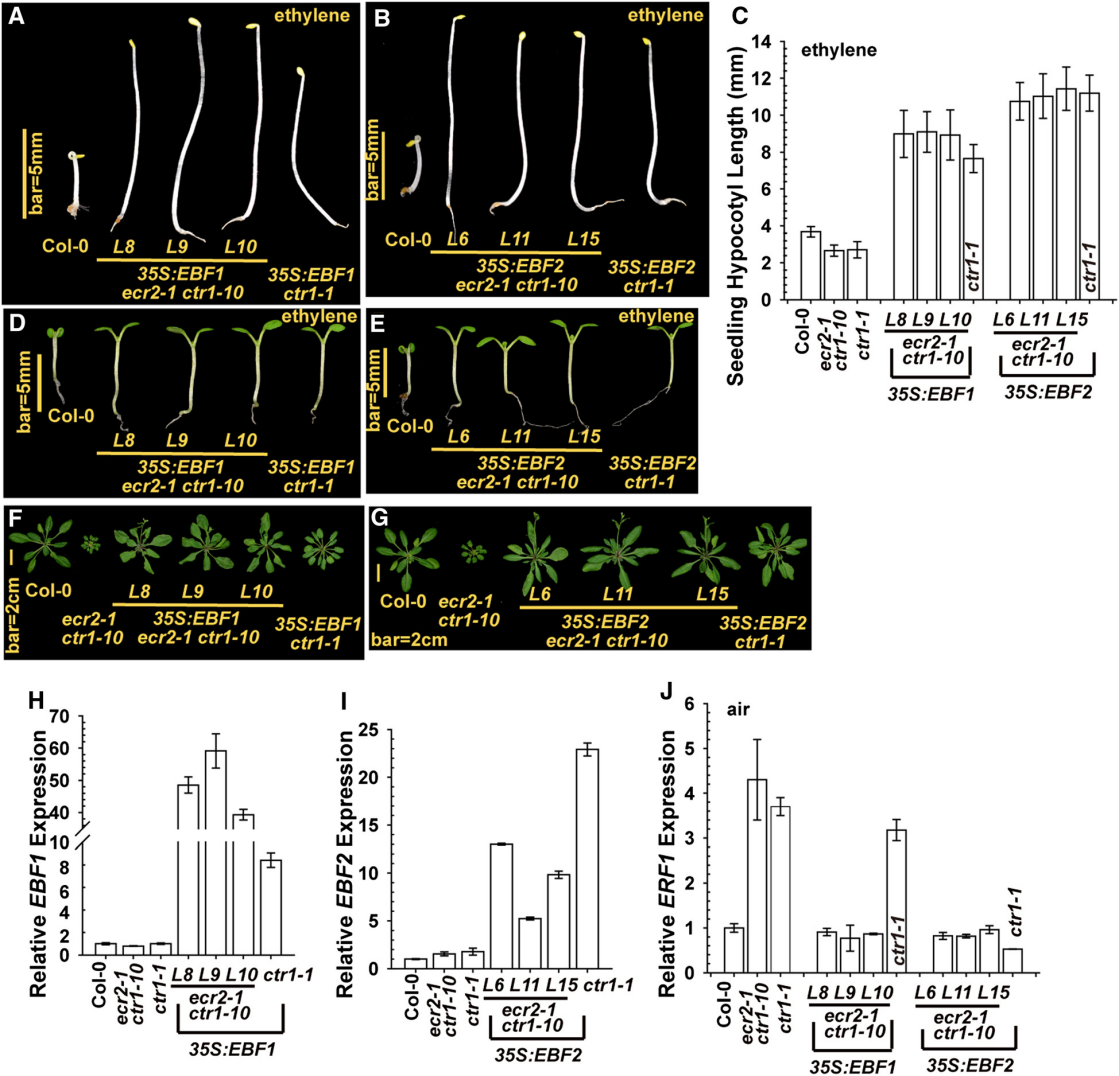
**Overexpression of *EIN3-BINDING F-BOX1 *(*EBF1*) and *EBF2 *rescues *ecr2-1 ctr1-10 *mutant phenotype and confers ethylene insensitivity.** Phenotype of etiolated, ethylene-treated *ecr2-1 ctr1-10* seedlings expressing *35S:EBF1***(A)** and *35S:EBF2***(B)** and their seedling hypocotyl measurement **(C)**. Phenotype of light-grown seedlings (with ethylene treatment) **(D)** and **(E)** and rosettes **(F)** and **(G)** for *ecr2-1 ctr1-10* expressing *35S:EBF1* and *35S:EBF2*, respectively. qRT-PCR analysis of mRNA levels of *EBF1***(H)**, *EBF2***(I)**, and *ERF1***(J)** in the transformation lines. Data are mean ± SD for hypocotyl measurement and mean ± SE for gene expression.

*EBF1* and *EBF2* were each expressed under the regulation of the constitutive *CAULIFLOWER MOSAIC VIRUS 35S* promoter in *ecr2-1 ctr1-10* and *ctr1-1*. As expected, with ethylene treatment, hypocotyls were longer for etiolated seedlings of *35S:EBF1 ecr2-1 ctr1-10* and *35S:EBF2 ecr2-1 ctr1-10*, than the wild type (Col-0), as well as longer for the strong allele *ctr1-1* expressing the transgene than *ctr1-1* (Figure 6A, 6B and 6C). Consistently, ethylene treatment inhibited the cotyledon expansion in the wild type (Col-0) but not the transformation lines in light-grown seedlings (Figure 6D and 6E). At the adult stage, the expression of each transgene largely rescued the growth inhibition phenotype in *ecr2-1 ctr1-10* and *ctr1-1*. Rosettes were slightly smaller for *35S:EBF1 ecr2-1 ctr1-10* lines than the wild type, but those for *35S:EBF2 ecr2-1 ctr1-10* lines and the wild type were similar in size (Figure 6F and 6G). Confirmation of the expression of the transgenes by qRT-PCR revealed lower *EBF1* level in *35S:EBF1 ctr1-1* than in *35S:EBF1 ecr2 ctr1-10* lines (Figure 6H). The lower *EBF1* expression could be associated with the stronger phenotype of rosette growth inhibition in *35S:EBF1 ctr1-1* than *35S:EBF1 ecr2-1 ctr1-10*. In contrast, *EBF2* level was higher in *35S:EBF2 ctr1-1* than *35S:EBF2 ecr2-1 ctr1-10* lines (Figure 6I).

We also examined other aspects of the ethylene response affected by the transgenes. *ERF1* expression was greater in *ecr2-1 ctr1-10* and *ctr1-1* than the wild type; the expression of each transgene largely reduced *ERF1* levels (Figure 6J). Of note, *ERF1* expression was greater in *35S:EBF1 ctr1-1* than *35S:EBF1 ecr2-1 ctr1-10* lines, likely because of its lower *EBF1* expression (Figure 6H).

Consistent with the suppression of *ecr2-1 ctr1-10* by *ein3-1* and *eil1-1*, the respective elevation in *EBF1* and *EBF2* levels in *ecr2-1 ctr1-10* rescued the inhibition in mutant growth and reduced *ERF1* levels, which supports that ECR2 acts upstream of the transcriptions factors EIN3 and EIL1 in the ethylene signal transduction pathway.

## Discussion

Arabidopsis ethylene signaling is negatively regulated by ethylene receptors and the Raf-like protein CTR1 [1-3,35]. The biochemical nature of receptor signaling is unknown, as are the underlying mechanisms by which CTR1 is activated [3,14,36]. A genetic screen for enhancers facilitates the isolation of genes or mutations that play a role in the same biological process as the known mutation. Given that the *ctr1-10* mutation does not disrupt the *CTR1* ORF and that *ctr1-10* is a weak allele, an enhancer screen for *ctr1-10* could lead to isolation of components involved in CTR1-mediated ethylene receptor signaling.

In this study, we reported the isolation of *ecr2-1* as an enhancer mutation for *ctr1-10*. With a relative weak constitutive ethylene response phenotype for *ctr1-10* and a strong phenotype for *ecr2-1 ctr1-10*, the mutations *ecr2-1* and *ctr1-10* synergistically facilitated ethylene signaling. The assumption that CTR1 level in *ctr1-10* could be reduced needs to be verified biochemically. Nevertheless, extensive studies suggest that for a common transcript, a long 5′-UTR can be translationally inhibitory as compared with a short one [22,37,38]. Conceivably, the T-DNA insertion that increases the 5′-UTR length may add structures of higher order and uORFs to inhibit the translation of *CTR1*, thus reducing CTR1 level, so that *ctr1-10* shows a weak constitutive ethylene response phenotype. We considered 3 scenarios explaining the synergistic effect of *ecr2-1* and *ctr1-10*: 1) ECR2 could be required in part for CTR1 activity or the protein stability, 2) ECR2 and CTR1 act in different pathways, or 3) ECR2 could be a signaling molecule acting with CTR1 to suppress the ethylene signaling. In the first scenario, CTR1 level, and possibly activity, in *ctr1-10* is presumably reduced to a level requiring ECR2 to suppress the ethylene signaling.

The nature of *ctr1-10* and *ctr1-8* mutations is distinct: CTR1 level could be reduced in *ctr1-10* and the ctr1-8 protein is not associated with the ethylene receptors [4,18]. The docking of kinase-defective ctr1 isoforms at the HK domain of ETR1 prevents receptor signaling to an alternative CTR1-independent pathway. In *ctr1-8*, the ethylene receptors are free from association with ctr1-8; thus, the receptor signaling is mediated to the alternative pathway and the mutant shows a weak phenotype [14,17]. Conceivably, in *ecr2-1 ctr1-10*, the activity of CTR1 docking at the ethylene receptors could be greatly reduced, and ethylene receptor signaling that is mediated by CTR1 as well as the alternative pathway was prevented. In contrast, the ctr1-8 protein does not dock at the ethylene receptors and the receptor signaling can be mediated by the alternative, CTR1-independent pathway; thus, *ecr2-1* had little effect on *ctr1-8* phenotype. In line with these results, our data showing that *ecr2-1* prevented etr1-1 and etr1-2 but not etr1-1^1-349^ receptor signaling in *ctr1-10* also support that ECR2 is involved in the ethylene receptor signaling that is dependent on CTR1.

An alternative explanation for the distinct effects of *ecr2-1* on *ctr1-10* and *ctr1-8* could arise from a role of ECR2 in CTR1 stability. In this scenario, very small amount of the ctr1-8 protein that could associate with the ethylene receptors could have a higher turnover rate in the absence of ECR2; meanwhile, ctr1-8 reserved in the soluble fraction could be continuously recruited to the receptors and mediate the receptor signaling. Contrarily, in *ctr1-10*, CTR1 level is highly reduced without ECR2 and the receptor signaling is prevented. However, this scenario is not consistent with the argument that the ethylene receptor signaling can be mediated to an alternative pathway that is independent of CTR1. In other words, if ECR2 were involved in CTR1 stability, the ethylene receptor signaling that was mediated independent of CTR1 still occurred and the *ecr2-1* allele would have little effect on the degree of *ctr1-10* ethylene responses.

## Conclusions

CTR1 is presumably activated by the ethylene receptors, and the mechanism is unknown [1]. Little is known about the presence of any other components that are involved in the ethylene receptor signaling to CTR1. The present data suggest that ECR2 is a component involved in part in CTR1-mediated ethylene receptor signaling, which indicates a regulatory mechanism for the receptor signaling. We favor the argument that CTR1 activity but not level was affected by *ecr2-1* in *ctr1-10*. With limited sequence information for the 598-kb region that contains *ECR2*, the cloning of *ECR2* is currently hampered. Complementation of *ecr2-1* by a large DNA fragment that contains *ECR2* is ongoing. However, with repeated sequences, transposons, retrotransposons, and sequence gaps in this region, cloning of a large DNA fragment is still highly challenging. Nevertheless, the cloning of *ECR2* will advance our knowledge of ethylene signaling that involves CTR1 activation.

## Methods

### Plant materials and growth conditions

*ctr1-10* and the PCR primers for genotyping were previously described [18]. For mutagenesis, following ethyl methanesulfonate treatment, *ctr1-10* seeds (M_0_) were washed with continuous flowing water and grown on soil as 380 pools (20–25 M_1_ plants in a pool). Seedlings at the M_2_ generation showing the constitutive ethylene response phenotype were candidates carrying the enhancer mutation and were characterized for other aspects of the ethylene response. We screened 162 pools, and *ecr2-1 ctr1-10* was isolated. For the growth of etiolated seedlings, seeds were stratified at 4°C for 72 hr and then moved to 22°C for germination (80 hr) in the dark. Seedling hypocotyl length was measured by use of Video tesT (Moscow) [29]. For measuring growth of seedlings and rosettes under light, stratified seeds were germinated and grown under 16-hr light/8-hr dark at 25°C; seedlings were phenotyped 7 days after germination and rosettes 4 weeks after germination. The isolation of *ecr2-1* and the genetic crossing of *ecr2-1* with *etr1-1*, *etr1-2*, *ctr1-8*, *ein3-1*, and *eil1-1* are described in the Results section and the segregation was verified by chi-square test (α=0.01). For ethylene treatment, the ethylene concentration was 20 μL L^-1^, or otherwise as indicated.

### Leaf senescence test

Detached leaves from plants were incubated in a Petri dish with wet filter paper in the dark for 5 days. Leaf senescence was quantified by measuring chlorophyll *a* content [39].

### Quantitative RT-PCR analysis

qRT-PCR of mRNA expression involved use of StepOne Plus (ABI). Each measurement was repeated 3 times with 3 independent biological materials (*n* = 3 × 3). The primer sequences were ERF1-F (5′-TTTCTCGATGAGAGGGTC-3′) and ERF1-R (5′- AAGCTCCTCAAGGTACTG-3′) for *ERF1*, etr1-1-NF (5′- GCTTTTATCGTTCTTTA-3′) and etr1-1-NR (5′-GCTTTATTTTTCAAGAAA-3′) for *etr1-1* and *etr1-1*^*1-349*^, EBF1-F (5′-GGAGATTGATGTTCCTTCCAAGA-3′) and EBF1-R (5′-CAATAGACCGAAGACCAAGATC-3′) for *EBF1*, EBF2-F (5′-CTTCAGATTTAGTGGTGATGAAG-3′) and EBF2*-R* (5′-CAAGCACTCCTCTCTTGTCCA-3′) for *EBF2*, and UBI-F (5′-ATGGAAAATCCCACCTACTAAATT-3′) and UBI-R (5′-TTGAACAACTCGTAGCAACTCATC-3′) for *ubiquitin* (the calibrator).

### Transgenes

The *ETR1p:etr1-1*^*1-349*^ transgene was previously described [14]. The *EBF1* cDNA fragment was released from a cDNA clone with the restriction enzymes *Bam*HI and *Sma*I and cloned to the binary vector *pCAMBIA1301* with the constitutive *35S* promoter. An *EBF2* genomic fragment was cloned by PCR with the primer sequences EBF2-F-BamHI (5′-TCGGATCCAAATGTCTGGAATCTTCAGATTTAG-3′) and EBF2-R-BamHI (5′-GCGGATCCTTAGTAGAGTATATCG-3′). The genomic *EBF2* clone was confirmed by sequencing and cloned to *pCAMBIA1301*. The transgenes were each transformed to *ecr2-1 ctr1-10* by *Agrobacterium*, with the floral-dip method [40,41], and phenotypes were scored in T3 and higher generations.

### Ethylene measurement

Ethylene evolution in light-grown seedlings was measured by use of the Ethylene Detector (ETD-300 by Sensor Sense) with the “stop-and-flow” measurement. In brief, 25–30 seedlings were grown in a vented vial for 5 days after germination. The vial was closed for 3.5 hr to let ethylene accumulate, and the ethylene amount was measured. We measured 3 independent biological samples for each genotype, and ethylene evolution is represented as mean (nL L^-1^ seedling^-1^ hr^-1^)±SD.

### Statistical analysis

Data are expressed as mean±SD or mean±SE for gene expression. Student’s *t* test (α = 0.01) was used for comparing 2 groups and Scheffe test (α = 0.01) for multiple groups. Chi-square test (α = 0.01) was used for testing segregation ratio.

## Competing interest

The authors declare that they have no competing interests.

## Authors’ contributions

AX conducted the experiments. WZ isolated the allele, and C-KW supervised the work and wrote the paper. All authors read and approved the final manuscript.

## Supplementary Material

Additional file 1 **BAC clones and sequences encompassing *ECR2.***
Click here for file
