# Supplementary material for: ENHANCING CTR1-10 ETHYLENE RESPONSE2 is a novel allele involved in CONSTITUTIVE TRIPLE-RESPONSE1-mediated ethylene receptor signaling in Arabidopsis
Source: BMC Plant Biol. 2014 Feb 15;14:48. doi: 10.1186/1471-2229-14-48 (PMC3933193; doi:10.1186/1471-2229-14-48)
Supplement: Additional file 1 — BAC clones and sequences encompassing ECR2. [file 1471-2229-14-48-S1.doc]

**Additional file 1. BAC clones and sequences encompass *ECR2*.** List of BAC clones and annotated genes we sequenced for *ecr2-1* in this study. Among those, T-DNA insertion mutants for At2g07981 and At2g08986 were each genetically crossed with *ctr1-10*, and the resulting double mutants did not show *ecr2-1 ctr1-10* phenotype. High-lightened sequences indicate *ecr2-1* DNA that we sequenced in this study.

| **BAC clones** | **annotated genes we sequenced** | **Annotated genes we did not sequence** |
| --- | --- | --- |
| **T12J2** | At2G07690 (Member of the minichromosome maintenance complex)  At2G07750 and a 2.3-Kb promoter [DEA(D/H)-box RNA helicase family protein]  At2G07760 and a 0.6-Kb promoter [Zinc knuckle (CCHC-type) family protein]  At2G07710 {transposable element gene; similar to unknown protein [Arabidopsis thaliana] (TAIR: At5G32410.1)}  At2G07791 (transposable element gene; pseudogene, hypothetical protein)  At2G07740 (transposable element gene; similar to nucleic acid binding / zinc ion binding [Arabidopsis thaliana] (TAIR: At2G01050.1); similar to Zinc finger, CCHC-type; Endonuclease/ exonuclease/ phosphatase [Medicago truncatula] (GB:ABE79649.1); contains InterPro domain Nucleotide-binding, alpha-beta plait) | At2G07789 [gypsy-like retrotransposon family (Athila)]  At2G07788 [gypsy-like retrotransposon family (Athila)]  At2G07790 [CACTA-like transposase family (Tnp2/En/Spm)]  At2G07720 [gypsy-like retrotransposon family (Athila)]  At2G07730 [non-LTR retrotransposon family (LINE)]  At2G07700 (gypsy-like retrotransposon family)  At2G07770 {transposable element gene; similar to unknown protein [Arabidopsis thaliana] (TAIR: At3G15600.1); similar to unknown protein [Arabidopsis thaliana] (TAIR: At3G43530.1); similar to hypothetical protein B14911_03489 [Bacillus sp. NRRL B-14911] (GB:ZP_01168655.1); contains InterPro domain Protein of unknown function DUF287}  At2G07780 (Mutator-like transposase family)  At2G07880 {transposable element gene; similar to unknown protein [Arabidopsis thaliana] (TAIR: At2G11090.1)} |
| **T6C20** | At2G09388(unknown protein) | At2G09790 (gypsy-like retrotransposon family)  At2G09589 [gypsy-like retrotransposon family (Athila)]  At2G09187 [gypsy-like retrotransposon family (Athila)] |
| **Tested by genetic crossing** | At2G07981(unknown protein)  At2g08986(unknown protein) |  |
| **T14C8** | At2G09795 (Potential natural antisense gene)  At2G09810 (transposable element gene; pseudogene, similar to putative helicase)  At2G09838 and a 1.1-Kb promoter (unknown protein)  At2G09840 [BEST Arabidopsis thaliana protein match is: nucleic acid binding; zinc ion binding (TAIR: At2G06904.1)] | At2G09820 [gypsy-like retrotransposon family (Athila)]  At2G09880 (transposable element gene; pseudogene, hypothetical protein, similar to hypothetical protein GB: AAC97247)  At2G09830 (copia-like retrotransposon family)  At2G09850 (gypsy-like retrotransposon family)  At2G09860 [non-LTR retrotransposon family (LINE)]  At2G09862 [CACTA-like transposase family (Tnp2/ En/ Spm)]  At2G09870 [gypsy-like retrotransposon family (Athila)]  At2G09865 {transposable element gene; similar to unknown protein [Arabidopsis thaliana] (TAIR:AT1G38380.1)}  At2G09800 (copia-like retrotransposon family)  At2G10080 [gypsy-like retrotransposon family (Athila)]  At2G10120 [gypsy-like retrotransposon family (Athila)]  At2G10100 [gypsy-like retrotransposon family (Athila)]  At2G10110 {transposable element gene; similar to unknown protein [Arabidopsis thaliana] (TAIR: T2G06914.1); similar to microfilarial sheath protein SHP3 precursor [Bru (GB:AAB86941.1)}  At2G10150 [non-LTR retrotransposon family (LINE)]  At2G10140 [CACTA-like transposase family (Tnp2/En/Spm)]  At2G09960 (transposable element gene; pseudogene of unknown protein)  At2G09920 (gypsy-like retrotransposon family)  At2G09953 (transposable element gene; pseudogene, hypothetical protein )  At2G09950 [gypsy-like retrotransposon family (Athila)]  At2G09910 {transposable element gene; similar to ASY2, DNA binding [Arabidopsis thaliana] (TAIR: At4G32200.1); similar to filament protein-related [Arabidopsis thaliana] (TAIR: At4G20730.1); similar to filament protein-related [Arabidopsis thaliana] (TAIR: At3G10100.1); similar to hypothetical protein 27.t00126 [Brassica oleracea] (GB:ABD65062.1); contains domain (Phosphotyrosine protein) phosphatases II (SSF52799)}  At2G10000 [CACTA-like transposase family (Tnp1/En/Spm)]  At2G10010 [CACTA-like transposase family (Tnp2/En/Spm)]  At2G09956 (transposable element gene; pseudogene, hypothetical protein)  At2G10040 [CACTA-like transposase family (En/Spm)]  At2G10030 [CACTA-like transposase family (Tnp1/En/Spm)]  At2G10070 {transposable element gene; similar to unknown protein [Arabidopsis thaliana] (TAIR: At1G40125.1)}  At2G09900 {transposable element gene; similar to unknown protein [Arabidopsis thaliana] (TAIR: At1G40115.1)}  At2G09890 (transposable element gene; pseudogene, similar to putative helicase, PIF1 family of mitochondrial helicases)  At2G10105 [transposable element gene; contains domain ATHILA RETROELEMENT ORF1 PROTEIN (PTHR10178: SF9); contains domain GAG/POL/ENV POLYPROTEIN (PTHR10178)] |
| **F7B19** | At2G09970 [Protein of unknown function (DUF1677))  At2G09990 (Ribosomal protein S5 domain 2-like superfamily protein)  At2G09992 (pseudogene of disease-resistance protein)  At2G09994 (pseudogene of U-box domain-containing protein/ armadillo/ beta-catenin repeat family protein)  At2G10020 and 2.1-Kb promoter (unknown protein)  At2G10014 and 2.1-Kb promoter (unknown protein)  At2G09930(transposable element gene; pseudogene, hypothetical protein)  At2G10050{transposable element gene; similar to pol polyprotein- like [Solanum tuberosum]}  At2G10090{transposable element gene; similar to unknown protein [Arabidopsis thaliana] (TAIR: AT2G04460.1); similar to pol polyprotein [Citrus x paradisi] (GB:AAK70407.1)}  At2G10130(transposable element gene; pseudogene, similar to putative AP endonuclease/ reverse transcriptase) |
|  |
|  |
| **T15D9** | At2G10260 and a 3.1-Kb promoter (unknown protein)  At2G10232 (transposable element gene; pseudogene, hypothetical protein)  At2G10226(transposable element gene; pseudogene, similar to putative reverse transcriptase)  At2G10237(transposable element gene; pseudogene, hypothetical protein)  At2G10234(transposable element gene; pseudogene, hypothetical protein)  At2G10270(transposable element gene; pseudogene, hypothetical protein)  At2G10253(transposable element gene; pseudogene, hypothetical protein) | At2G10160 [gypsy-like retrotransposon family (Athila)]  At2G10170 [gypsy-like retrotransposon family (Athila)]  At2G10175 (transposable element gene;similar to unknown protein [Arabidopsis thaliana] (TAIR: At3G30650.1)  At2G10180 [gypsy-like retrotransposon family (Athila),]  At2G10230 (transposable element gene; pseudogene, replication protein A1)  At2G10165 [gypsy-like retrotransposon family (Athila)]  At2G10265 (Mutator-like transposase family)  At2G10280 [gypsy-like retrotransposon family (Athila)]  At2G10210 (gypsy-like retrotransposon family,)  At2G10220 [gypsy-like retrotransposon family (Athila)]  At2G10205 (gypsy-like retrotransposon family)  At2G10223 (transposable element gene; pseudogene, hypothetical protein)  At2G10190 [gypsy-like retrotransposon family (Athila)]  At2G10240 (transposable element gene; pseudogene, similar to putative helicase, PIF1 family of mitochondrial helicases)  At2G10250 [gypsy-like retrotransposon family (Athila)]  At2G10200 [gypsy-like retrotransposon family (Athila)]  At2G10256 [hAT-like transposase family (hobo/Ac/Tam3)) |
| **F7K9** | At2G10300 (transposable element gene; pseudogene, hypothetical protein) | At2G10285 {transposable element gene; similar to unknown protein [Arabidopsis thaliana] (TAIR:AT5G31838.1)}  At2G10290 [gypsy-like retrotransposon family (Athila)]  At2G10310 [gypsy-like retrotransposon family (Athila)]  At2G10280 [gypsy-like retrotransposon family (Athila)] |
| **F12P23** | At2G10340 (unknown protein)  At2G10430 (pseudogene, similar to putative reverse transcriptase)  At2G10440 and 2.2-Kb promoter (unknown protein)  At2G10450 and a 2.2-Kb promoter (14-3-3 family protein)  At2G10390 {transposable element gene; similar to unknown protein [Arabidopsis thaliana] (TAIR:AT1G35100.1)} | At2G10330 (gypsy-like retrotransposon family)  At2G10470 {transposable element gene; similar to unknown protein [Arabidopsis thaliana] (TAIR: At2G10836.1)}  At2G10350 {transposable element gene; similar to Ulp1 protease family protein [Arabidopsis thaliana] (TAIR: At4G03970.1); similar to Ulp1 protease family [Arabidopsis thaliana] (TAIR: At3G42530.1); similar to Ulp1 protease family protein [Arabidopsis thaliana] (TAIR: At1G44880.1); similar to hypothetical protein 23.t00036 [Brassica oleracea] (GB:ABD65624.1); contains InterPro domain Peptidase C48, SUMO/Sentrin/Ubl1}  At2G10320 [gypsy-like retrotransposon family (Athila)]  At2G10370 {transposable element gene; similar to unknown protein [Arabidopsis thaliana] (TAIR: At1G44860.1); similar to hypothetical protein 31.t00082 [Brassica oleracea] (GB:ABD65101.1); contains InterPro domain Nucleic acid-binding, OB-fold, subgroup; (InterPro:IPR012340); contains InterPro domain Protein of unknown function DUF223, Arabidopsis thaliana; (InterPro:IPR003871); contains InterPro domain Nucleic acid-binding, OB-fold}  At2G10380 {transposable element gene; similar to unknown protein [Arabidopsis thaliana] (TAIR: At5G36070.1)}  At2G10360 {transposable element gene; similar to unknown protein [Arabidopsis thaliana] (TAIR: At1G44870.1)}  At2G10420 [CACTA-like transposase family (Tnp2/En/Spm)]  At2G10410 (transposable element gene; Member of Sadhu non-coding retrotransposon family)  At2G10400 (Mutator-like transposase family)  At2G10405 [hAT-like transposase family (hobo/Ac/Tam3)]  At2G10465 {transposable element gene; similar to replication protein-related [Arabidopsis thaliana] (TAIR: At5G35260.1); contains InterPro domain Nucleic acid-binding, OB-fold}  At2G10460 {transposable element gene;similar to unknown protein [Arabidopsis thaliana] (TAIR: At4G07450.1); similar to hypothetical protein 31.t00031 [Brassica oleracea] (GB:ABD65118.1); contains InterPro domain Nucleic acid-binding, OB-fold, subgroup; (InterPro:IPR012340); contains InterPro domain Protein of unknown function DUF223, Arabidopsis thaliana; (InterPro:IPR003871); contains InterPro domain Nucleic acid-binding, OB-fold} |
| **T4D8** | At2G10535 (Encodes a member of a family of small, secreted, cysteine rich protein)  At2G10537 (other RNA)  At2G10550 (Most likely an inactive paralog of the uracil DNA glycosylase AtUNG (At3g18630) generated by a gene duplication event and subsequently disrupted by transposon insertions)  At2G10557 (unknown protein)  At2G10560 and a 1.8-Kb promoter [unknown protein; BEST Arabidopsis thaliana protein match is: unknown protein (TAIR: At5G04860.1)]  At2G10589 (unknown pseudogene) | At2G10490 [CACTA-like transposase family (Ptta/En/Spm)]  At2G10480 [CACTA-like transposase family (En/Spm)]  At2G10500 {transposable element gene; similar to unknown protein [Arabidopsis thaliana] (TAIR: At4G07430.1); contains InterPro domain Nucleic acid-binding, OB-fold, subgroup; (InterPro:IPR012340); contains InterPro domain Protein of unknown function DUF223, Arabidopsis thaliana; (InterPro:IPR003871); contains InterPro domain Nucleic acid-binding, OB-fold}  At2G10540 (gypsy-like retrotransposon family)  At2G10530 (copia-like retrotransposon family)  At2G10520 (copia-like retrotransposon family)  At2G10510 (copia-like retrotransposon family)  At2G10555 {transposable element gene similar to replication protein-related [Arabidopsis thaliana] (TAIR: At5G35260.1); contains InterPro domain Nucleic acid-binding, OB-fold}  At2G10610 (copia-like retrotransposon family)  At2G10570 (copia-like retrotransposon family) |

**T12J2 sequence**

AAGCTTTGTCAGAGCTTCTCAGTGAAATGTTGTTAGAATCTGATCACTTGTAACTTCTGATCTCTGCTATAATTATCTTGTCATGGAAATTAAGAAAAACTAGTTTCAACTGTAGTAGCTCATTTATATGCTCAGTTTTCCATAATAAATGAGTCTTCATGGACTTGCCTAATCCAAATTCATAAATATCTATCTAATTCGATGGTGATTGAGAACTGAGAAGTAAACAATGGTACTCTTATTGTCTGTTTTTCTTCCAAGAAGTTAGTTCAGAAGGAATTGATTCTTCATTGCAACTTAAAACAGCTTTAGCCATGAGTTTCCCAAGCTATGCATGATAGAGTAAAGTAATTGGCCAAAAATATGTTAAATAGAGAAGCAATTATCTCTCAACAACACAAGAACAAAATGAAATTGAGAGATTCCGGGAGCTTCAGAACCAGTTCAAAATTCAACAACATACTAGTTTTGTAACCCGAACCAGTTTAAAAGAAGAACATTTTGAGTCAAGTTCAACCATGGTTATTTATTTCATTTTTTTTTCAAGGTAACAACATTCTACTAATAACAACATTAAGAAAATTTTAATTTACATATACGTTCAGATTACACTTTTACTTTATCAACTATTCTATCTGCATCTTTACTTTAACTATCGGCACTAGATTATACCATTCAGCAATCTTCAAACGCTTTCCTAACATTGGCGTGACATTTTAACTAAGGACAACAAAGGTGGAGAATACATTAGTTTGAAGCAAGCGTAAAAGTTTACTAAGAATCTTCATCAAATACACATGACATAAGCTTCTCTTTCTACTTTTTGCCTACAAATTAACTTGATAATATTAAATATTTTGTTTTGTTTGGTCATCGCTGCTACTAAAGAAGAGAGATTGCATGCCTAGACGATCAAGCTTTGCGGACAATAGAACGTCTCTCTCGCTGGTACTCAACTTCACCTCTCTGGTGCATTATCAACAAAGCTCTCCTCACCTGCCATTTTAACATGCACATTACCTCAAAAATCCCATTCACATTACTGAAAATTAAACAACAAAGATGTTAGCTTAGGTACCATTGAATCGTTCATTCCCATTCTAGCCAGATCCTCTATCAGTCTTCTCTCTGATAATCGAGCTCCGATCCCCATTCTTCTCTTTATCTGTGTTTCCGCTTGCTGTTTTTTCACACCAATACCAAATTTAGTTATTACATCTAGAGATACGATGATTCTTTCTTCCCCACAAGCAACTAACCTTGATTTCATTGGCCATCTCGCCTGTTATGTTTATCTGTTGATTTATCCCTGATCTTGCTGCATCCATTGTCGAGGTATCAAAAAGTTTAAAAGCTTTGTCTACATCATCTGGTGTGGCCTCGTGTGACCTGACATATTTAACAATGTCAATGGACGGCACATCCCATCAGGTTTCAGGAATCAGGAATCTCTATGTTAATAAGATTACTTACAGTCTCATTTTTGCAAGTGACTCACTCAGTCTGACAATAGCTTCGAGTTGTCTCACTGTGATAGGTATCGGAGCAGCCTCTCCTGTTTCATGTGCACGTCTCTTCATATCCTGTTTAGCCGAAAATTTTATACTGACACCAACTTTGTTTTTTAACCATCTTATTGTCAAATCTAATGTAAATGACAATTACCATTCTGATTGTGACATATTTCCGCTGCAGGTTCTCAGCAGCGTCTTTTGAGAGACGGGGATGACACCTTGCTCGACAGTATTGTATATACCTAACATAAAAAGAGCATTATATTTTAGAGTCATGATTTTGTACAGAAAACTGCGAGAACACATTGTTTTCTCGACGAATGAAAACACACCTTTTGAGCCAATTATCTTCTTTAGAATCTGTGTTTTCATCCGAAAATTTGTTTGCAGATGCATGAACCCTTATAATATGGCTGGCTATTTCCTGTGAAAAGATTATAAACATTAATCATGTCTACATAATAAGCAAGCAACGAAGCAAACTTTCCAATAACAGTGACCTCCATCATTAATCTCTGTTTTCCCTTTTAACTACATAAAATTTCTGAAAGAATTGTTCCTTTCCTTGCTTACCTTGTCTTGGGAATACTTCCTGATGTCCTTGACAATGAAGATAAGATCAAATCTAGAAAGAATTGTTGTCTGCAGATCGATATTGTCCTGAGCAGTCTGCAAATCCAAAGAAAAAAAAACACACCCATCTCTTAAGAATATTGCAAACAAGAATCGGGAAAACCATGATATTGCCAAAGGAGAGTACCAGATTTCAAACCTTAAGGTCATCATATCGACCAGATGGCGGGTTAGCAGCAGCAAGCACCGAGGTTCTAGAATTAAGGACAGTTGTTATCCCAGCTTTTGCAATGGAGATTGTCTGCTGCTCCATTGCCTCATGAATAGCAACCCTATAAAGAAAAAAAAAACATTCCATCAATTCTACTCATAGAGAAAAATTAGATTCGTCAACACTATGATTCAGATTTGAATTACCTATCTTCCGGTCTCATCTTGTCAAATTCATCAATACACACAACACCTCCATCAGCCAAAACCATAGCACCACCTTCAAGATAGAACTCTCGCTGCAATGAACATATGTATAAATAACTGAGAATCTGTCATAGCAACTACTTTTAAACAAATCATAGAAAAACACTTGAAGATAGGACATACAGTGCTGCTATCTCGAATCACCGAAGCTGTGAGCCCAGCAGCCGATGAACCCTTTCCAGATGTATAAACAGCAATTGGAGCCGTCTTCTCCACAAACTTAAGAAACTAGAGAGAAAAAAAATAAGCAATTAAGCTTTAAACATGTAAGCTACAGGTGTTGGAAATAACACAGACATTTTGTAAGGATTGTAATCTAACACTTGAACAGAAGGATGTAAGACGAATATCAAGCAAATAAGAGAGACGTAAGAAAATATAAAAAGAATAACACACTTATTATAAACCATTGATACTGAGACCACATTACAAACCTGTGATTTTGCAGTTGATGGGTCTCCCAAAAGCAAAACATTAATATCACCTCTTAGTTTCACTCCATCGGGCAGGCTCTGAATCAAAGATAGAAATGTATATAAATATATCAAAAACGTATACAAAGTGCACAGAAAATAAAAACTGCTATCACAGTTTCCTACCTTCCTGGATCCTCCAAAGAGAAGACACGCAGCTGCTCTCTTCACATCCTCATGGCCAAATATAGAAGGAGCGATCTTGGTGCAAATGTTCTTGTATACATCTTGACTATCAGCAAACTTTTTGAATTCTTCCTCCTAAAAGTAATATTATGGAAGACAAAACTGATAAATGGAATTGTACAACTAGGCTTCCAAGCAGGCCTAAATGTTTCATAGTAAACTAGTAGCACAGAGAATATTTCCAGAAAACACCATCACATTCATTTGGGAGTTACACAAAGAATTTCATAGAGCCAACAGCTACCTAAGGCATTTGGTTTCCAGTAACAATAATTCAGCCATGTTAACTTCAATAGTTCATTGTGAAACCATGGATGACATAGGCTATCAAATGAGAAAACAAAAAGAACCAAATGATGCGATACTAACTTCGTCAGGAGTGAAGTTGGCAGGGCCACGGGAACTGGCCTCATTTGTGTCTTCCAGTCCAACAACTCGGATATAAGGCTGGCGAATTGCAACTGCTCCTTTATGCCTACATCAGAAAGTAGGAAAAGATGTCCATCATTGTTGTGGTGTAACTCTCCCAGAAAACAATCTACACTCTCATTTGACATAAAACTACAAAGCTTACGAGTTAGATGATGACGAAGCTTGGAAAATGCTGTAGATACCCATTACTGTCAGTCTGGTTCCAGGTACAATTGTTTGTACAAGATGCCGATCAACTGAGAGAAGCATATTTCTTGGAAGCTCTCCAGTAGGAACATCCTTCAAAACTCAGAAGCATTTAATATATACAAGTGCAAAAAAAGCGAAAACATTACAATGTAAATCTATACCTCCGGATTCTCCTGTAGCTTTAAGGTCTGTTGATCAACATACTGGCTCCTATCAGGAACCACCATCCAAGGATCAAGAGGGCAAGGCTCCTCTCCAGGCTAAACAAATTCCAATTTGGTCTAATCAACACCAAGGATAACTACAAACTCAAATCACAAACGAATCAAAAGAACTCACCTGTGGAATATTATCACATGATCGAGGAACAATTGCCCCACCAAGACCCGGACGACATGGAACTTCCCTTGTCTTTTTACAATTCTTACATACCAAAAACACATAAGTCGCCTTTGCTTTCACTCTCGAAGCTGCAATACTAATCCCAGAGATTTTCACCAACTTTGAGATGTACTGTGCCTATCAAGAGTAAAGAGTACATTATAGACATACCCCAAGTAATCTCATTGATACTAAAAACAAAAAAACAGACATACCCCAAGTAATCTCATTGATACTGGATCCTCTCTTGAAGTGAGCAAAATCTGCACATCTCTAGTCAGAGGTTCCTCCATTACCCCTCCCTCATTAGCCTCCCTCATTTTCAATCCAGTCAACACTTCACCAGCTGCTTTCTCAAACTACAATTCAAAGATTGACTCACTCAATACAAAGCTTATATGAACCCTAATTTTTTTTGAAGTCATACCACAGGGAGATAATCGGCAGGAGCAGATCGGATTAAGGAGGGGAGATCGGAATCAAAGGAGAGGAGATCTTCGAGGTGGACAACAAGTCGCTTAGGGTTATCGAGGAGAGCTTCTCTGTAGGGGAAACAATTCTGTTCGATCTCGAAGTTTCGAATGAATTCCTTAAATTTGGTTAAGACGGCGTGGGGACTGATGGTTGCGGCGTCTCCGGCCTCCGGGAACTGCGGCTGGTCGCTGTAGTAAACTGCTCCTTCGTCCCATCCTGACATCGTTGCTTCGTCTCCGTTACGAATTTCTCTATCTCTTTCTCTCTTATCAAAAAGTTCTAAACGAAGAGAGAGAGTGGGGGCAAGGGCTTTATGTCGACTATGAGTTTTGGCGGGAACAATGTGGCGGGAAACTGATGGGCCGAACCCAAGACCTATGGACGGGTAAATGGATTTCGTTGGTTATTGGGTCTTTCTTTCTTTTTCTTTTTAGCTAAGGTTACATTTTTTATCCATTTTTTCTAATTTATTATAAAAAACCAAACAGATAAAAATATAATTATGGACTACATCTAGGAATCTTAAATTTATGAATTGCACATTTTAGAATTTCATATTTTTAAGCTACTTAATATCGTTGTAAGCATTTTCAATTCCATCGTAATTTTCTTCAATGTCACTTAAAATTTAAGGCTATATTAATATATATGTTTAAAAGAATAGTTTTTAGTTTTATAACATTAATAATTTGAAAATTAAGTAACTATAAAAAAGAAAAGAAAAATCAACCATATATATCCTTCTCATTTACGTTAATTACAGAAATGTCATTAACATGATAAATGTTTTTGAGAATATTAGGTAATTGGACACTTTTTTGAAACTCAATTACGCAATAAAGCACTTTAGAAAGAGTAAACACTTTTTAAAGTTTTTTTACTACTACACCATCAACCCAACCTAAGCAATCCAAACTTACCATAATTTACAACCATTACTTTTTCTCTCTTTTCATTCATTTTTTTCTCTACTCTTTACAAAATTAAACACACAAAACACTTAAAATATCTTAATGAAAAAAGGAAATGGTTTGAGACATTATCTCTCTTCTCTCTCATCAAAAACACCAAAAAGTAGATCTAGATTTTTCTTTTTTCTTTCTCTTCTTAGATCTTAAAAGTTGGAAATTTTCAAGATTTTTATTCAGTTTCTCATTCTTTAATCAACCTCATAAGGTTAAAATTTTCATTTTTAATTGCTAGGTTTCTTCTTCTCTTAGTGTATCCATCATTGTCTTGGTCAAATTTTTGTCTTTTTACAAATCTTAGATTTGGCCACCAAATATTTGTGGAAATTCCTCAAAGAGAGAGAGAGAGTTGAATTAAAGAACCCAAACATATATTCTTACTATAAATTTGTAACTTAAAGGTAGTTTGATGCTATTTTCATTAAAACACATGAGCAAATAATGAAAAAATGGAAAAAATACAAAACAAATCAAAGAAGAGTGATAGGCGATAATTTGAAATGACCACATGGTCAGAGTTTGGTGGATGTGTGTTGACGGTCGATAATTCAAAATGATTGTGTTTTGTTAATACTATTCATAAAACCAGTGTTACGTTATGTAAAAGATGTTGATAAAATGTGATGAAACAAGTTTTGTTGGTCATATCAACAAAAGTTTATTTATGAAAATGTTTATATTTATTTATCCACGATTACATATTAACAAAACATAAATACGAGTGTATATCGTAAGATTGTGTATGAACACATCGATCTCAATAATATTTTACGTTTTCAGGTTAAGAAGCGTGCTTTAGATTATCATGGATCAAAAGTGGTGGTTATAGATTATGTACATATAAAACTTAGGTTTTAGGGTTTAGGCTTTAAAGTCAAGGGCTAGGGTTTAATGTCTAGGGTTTAAGATTTAGTATCTAGTGTTTACATTTTAGGATTTAGGCTTTATTGTTTAGGATTTCGCGTTTAGTTTAGATGATTTAGGGTTTTGGGTTGAAGAATCAAAGTTGTCTACAATGATTTAGGGTTTATGGTTACATGTTTCTCATTGGAGATTATATATTCCATTGTTGTCCATATGGATTGGATAATATGGATTTTCTATCTCAATTTGTTTATATTTAGGGGATTTATGTTTAATATTTATGGACAACAACAAGAAGTTTTAAAGTGAAGATCTTAGAATTTAGGGTTTAGGGTTTAGGGTATACGTGATATGGTATATGTATATTGTTAAGGGTTTAGTCTTTATGGTTTAACAGGTTTAGGGTTTAATATCAAGGGTTTAGAGTTTAGGGTTTAGGGTTTAGGGTTTAGGGGTTTAGGGTTTAGGATTTAGGATTTAGGATTTAGGATTTAAGATTTAGGGTTTAGGGTTTCGGGTATAAATTTAGGGGTTAGGGTTTAATGTTTAGAGTTTAGTATCTAGTGTTTAGGGTTTTAGATTTAGTGTTAAGGAACTATGATTGTTTACAATGATTATAGAGTTTATTGTAGATGGTATAAGGTTTAGGGTTAATTGACCATGGTCATATACAATTATTTTTGGGTTTAGGGTTATATGCTTCTTGTTAGATTATATATTTCCATTTTGATCATATGAATTAAATCATATGAATTTTTTATGTTCTCAATTGGTTTATGTTTAGGGGATTTATGCTTAATATTTATAAGCAATAATAAGAAGGTTTAAAATTTAGGGTTTATATGGTAAATGGTATATGATATATTTCTATGGTTAAGGGCTTAGTGTCTATGGTTTAAAGTTTAGGGTTTATGGTTTAGGTGTTTAAGGTTTAGAGTTTAGGGTTTAGCGTTTAGGGTTTAAAGTTTAGGGTTTAGGGTTTAGGGTTTAGGGATAAGAGGTTTAAGCTTAATATTTATGGGCAACATTAAGAAGGTTTGGGATTTAGGGTTTATGGTATATGGTATATGGTATAGGGTATACGTATATGGTTAAGGGCTTAGTGTTTATGGGTTAAAGTTTAGGGTTTAGGGTTTAGATATAGAAGTTAGGGTTTAATGTATAGAGTTTAATATCTAATGTTTAGGGTTTAGAATTTAGTGTTAAGGAACTATGATTGTTTATAATGATTTTAGAGTTTATGGTTATATGGTTCTCATTAAAGATTATATATTTCATTGTTGTCCAAATGGATTAAATCATATGGATTTTTTGTTTTCTGAATCTGTTTATGTTTTGATGATACAATAAATAATAGTATGAATAAATATAGTAATATGGATTTCATGAAGGAAAAAATGTTATTATTATATATTTTATAAAAAATGATTTTTAATCAAATAGAGAATGTCTTAAATTAAATAAAAATTAAATAACAAAAGGAAGAGAGAGAAGAAATTGACAAGAAAGATAAGAAAGTGATAGATAGAGAAAATATGTTTTATTTATTTAAATTTGACGGTCAAGATTAAAAGAGATTAACTTTACCTTTCAATTCGATGGACGAGATGATTCCGTCAAAACCATTTCTTAACCATGCTACTTGATTTAAGGTGAAGAGAATAAGGGGTAAACTGGTATTGATTTTTTTAAAATAGTGAAAAAACTCCAAAAAAGTGTCTATTCTCTTCAATGTGCCCTAAATGCACTAAAAACTTGGAAAATGCCCCAATCATGAAAGAAGCCCAATTTTTTCTGACGTAATATGTGGCGACACAGTTCATAAACCGCTCGATAACACTGCTCACATGCGACCGAAACACTATTCATCAGATGATATAGTAAAACATCATATGATTTAAGCATTTATCTATTTGTTTTATATGCTAAAAAATATGATATCGCGATAAGTTTACTTTTAAACAAATCTTTTATCGACCTAGAATTGAAAATTAATATATTTTTATTTATGTAAATATCAGTTCAATTTAAAGAATTAGTTTATTTCTTACATAATTAATATAATAGCAGTTAAAACAACATGTTATATAAAATAATAACATGCTAACATATTTAATATCTAGATAATATTTGTATGAACGTGCCATCTCAATCCACGTTTTCATTCACGCCACCTCTTTTTTTGTCAACTCACCAATACTGATTTTAATATCTTTGTTTCGATTTGATTTTATGTTTCCATTATTATTCTTATAGTTTTGTATCGTCAGAAATTAAATAAAAGAGAAACAAAAGAAAGAAAAAAAGAAAAGAGAGAAAAGGGGTAAAAGAGTCAAAAAGAGGATGATAAAAAGAGGATAAACAAAAAAGAGGATGATAGTGACATGTGCACAGTGATCAAGTGGTCAACCTAATTAATGAGTCGTTCATGTATATCTATTGATATTACCCTAAAATTTAAAGATGTGATTCAACTAAATAAAATAAGTTTATTACAAAAATATAAACTTTTTATTACCTTCTAAATTCACAAAACCACGTAGTCAATTTCAAGTATAAAGCAAGGTTTTGACAATTTTTAGAATAAGACAATTTTTTTTACAAAGATTTAACCACTTACATCCAATACTAAACGATGATTTTGTTACTATTGTGGTGATTAAAATGCTAAACACATAAAGACGCTCATAACCTCCATGTACGTTAAAGACATCAAATCTAAATATTTTTCCAAATTTGATGTTGTGTTGTCAAAACCAAGCTGGATGCTCCTCTTAACATCAAAGAGATCATGTATGATATAGACATACTAAATTAAACAAGTATGATTATTATACTATGAACTTGAAATGTGAATTGATTTAAGAATTTACACCCACAACCTCATATCTATCCCATAACTAGAATGGTAATGTACTAATCCCGATAATTTAGCAAACTTGAATTAAATCTTCCTAGCCATCTCTTCTACTCTCAAATCACTAATAAGGTTCAAATTACCCTAGAACTACTCTCTCATATAAGAGATCAATTGCAGTATTTAAGGATCGAATTCACAAAGTTCTTTTGTTCAAACAATAAGTTGAATGTCGAGATTAAGCTAGAAGGTGATGATTGAAATAGTAATAAAACAAAGTAAGATAACAGCAGGTTGATTGTGTTGTAAAAAATTAGGAAAAGAGGTAGGAACAGAGTATTCTCAAGAAACTATTGGTTAGTAGATCTAATGAAAGCTAGGTTGTTATCGAACCATTCTTAAACTCAAACCCTAATTATGGAATAACCGGTGGCTTTAGTACCCCGCGCATTGACCCCAGGCAAGAAAGAAGGGCAAGAGAAGAAGAAGAGAGAATGGTGCGTGAAATGAAAGGAAGGGCCGAGTATTTATAATCCGCGGAAGCTAGAGTAGAGCCCAAGTCTTAAAACGTGGAGCCGAATCCATTCGGGCAAAGCCGACTTGGTGACCAAGTTTTGTCCTGAGCAATGCACGCCAACTTTAGCCAACTTGGTGAGCAAGTTTTGTCCCAAAGCCAACTTGGTGACCAAGTTTTGTCCCACTCCTCATATCCGAACCTTGAGCCTATAAATAGAACCTTACCCCCCCCCCCTTAGTTACACCTTAGAAGCTTTCGCACCAAAACTCTGCCCAAACCGAGAGGGAGAGAGAGAAATCATCCGAACCCTTAACCCGAGAGAGAGAGAGAGATCGTCCGAACCTTAAAACCCGAGAGAGAGAGAGAGAGTCTGAGCAAGAGATCGTCGAGCCTTAGGATAGTCCGAAAGAGAGATCCCGAGTAGAAACCATCGGTCGAGTTAGGAAGATTCATCGGTGATTGTGAGTTCCGGTTTTTGTTTTGCATGTTTGATTTTCCTTCTTGCATGTTCTCGATTCTCCCTTCTCTTAGTTTTGTTGTTGTTATGGTTGAGTTTGTTGAGGTTGTTAACCTAATAGCTAGGATAGTTGAGACCGAAGCACCTTGCCCTATAGGAGTGGTTGTGTTAGAATAGCACCATTCACTTTAGAGGGATGGTTGCATTGTCAAGACACTACTCGCTAGTATTCGGGAGACATTTAGAGAGAACTTTATGAACATGTGCGTTGAAAACGAACCGCATGTTTCATAGTGTTGATTGGAAGATCGTAGTGTGTGGAGCTTGAAGAGTATCTAAGTTAACTCCATAAGCATGCTACGGTCAAGTGGGATGTATCTGACCTTTCCTTGTGATTCTGTTCGTTCGTGGGGTGTGCTTGACGAGATGCAAGTGACCTCAAGGGGTTCAAAGGACCTATAGAGACTGGGTTAGCTACCCAAGGCCCGGTGTCGCTTACACGATGGTGTAGCCAAGGTGATGTTCCAGTTATATGCATTTGTGGTTATAACTAGGGTGTTCACGATGTGTTGAGGAAGGCAGTGCATTCGGGTCCTAATTAAAACTTTAAGAAACGTTATCTTCTTGTTGTGTTGTGTGTTGCTTACTTCCTTCTTTGTGTACTCTGATTATTTATGTTATGCATGTGGGATTATGGGTGGGGATTAAACTAATGTTTCCTTAGGGAACCCGAGCTCATTGAGTAATGCTAGATTACTCATGACTTTACTTGTTTTGCATGAAACCTTAGAGGGGATTAGAGGGAGTGGTGAACACTAGGGAACCCGGAGTAGTTTTTCAATGTCCTTTTGTAAAACTTAAAATATGTATTTTCGTATCGTGTACTTTTGGACCTCATGCCTTGGCCACAAAAACGATGTTTATGTTGTGGTTTTTCCTTGTATTTTCGTAACTCTAAAATAATGAAATCGGTATTTATTTTAGAGTATACGGGTTTCAAACTGATAAGACTTTGTCCGAGTCGACACAACATTTGGGTGAAGTGAGGGTTGGCAAGCCTAACACTACAAAAAAATTTGAATATTCTTACGGAAAAATTGCGACCATTTTTTCGTCAGAAAATTGTAACCATTTTCTTACAAAAATGTGACGTATTTATTTAGTCATAAAAACGTTACTATTTCGTTATAATATTATGTCTTAATTGAAAGTAACTAACGTTTGTCACTAATTATATTAAATTTGTTACGAATTATATAGTCATAAATATAAATACGGATTTGCGACCAAATATGGTAACTAAATTATACAATAATTGTGACTAAACTTATGTCACGTATAGTTACGAGAGTTTTGTAACTATCATGTCACAATTTGTCACTGTTTTAGTGACTTTTGCGTTTGGTCATAGATTGTGACATAATTATTACCAATTTTATCACAAAAATAAAAACATGTGAACTAAAAGTCAAATAAAAAAACAGCTTCAATTTTGGAAAGAAAAAAATCTGATAATACATAAAATTTTGAAAAGAGTATAAAAAGTTATATAAGAAAACAGTTTCAATTTCTTTGTTATTTTTTCTTTTTATTTCCAATGCTTTGTTTTAAAAAAAAAAATCCCACGATCTCTCTTTTGCAATGCATGATCTCCAAATTAACTTCCCAGTTTTATTAATTATTTTCATCAATTTCACTTTCTTTAAATCACCATATATATCCCACAATCGATTTATCTCAATTGTTCTTCCATTCATCCCTTCTGGAAAAAAAAACTGTAAATACTTTTACGGAATCGTCATTCAACACCACGAATTCATCTTCATTGTTCGCTAAATCCAACATTCCACGACTCCGATCGTCTTCTTCCTCCTTTCGTAAGGTGGTTTCAGTAACCCAAATCACCACAAAATCTAATATTTTAAGAGAAAATCTTTGTTTTGATTTTTTTGATGTATTTAGTTGCAGGCACTACTGTTTGCAACATGAAGTCAAAATCCCCTTCTTTTTATATCTCCAGAAATATGAACAGTTAATGCTAATTAAAAGGTGTGATTCTGCTTCTTCTAATGTGGATCTTTTTGTCTAATTTAGATACATTATATAATATCTACCCTATTTACATCCTATTCTCTGTCAGCTATCCACCTCTACGTCCACACTTTTGTTTTGTTGGAGCGGCATCAATCGTTGATTATATGTGATCTTCATTTAAGGACAAAAGTTGGAATATCATCATATGCTTCGATCATGTCAAAGAACCCAAATTAAAGAAGCACAATCAAATGATCAACTGCTTGAAGGAGACACCAAAAGTTTCGTCATCAAAGAAGAAGATAATTGAGTGACAAAGAAGAAGTCAGGTGATGAGAAGCAAGAGATGTTCAAAGCATCAAAAGGCCAACTCACGGTTTCAATAAAACATTACGAAAAGTAATGGTTTTTGTGGGGGGTTGACATTATTTGAATTCATTTTCACCCTTTTTATTTGTTCAAAGTTTAGGATCTAATATGTAGTATACAAAAGCAAAGAATCTTGATCATCAATATATAGACCTATTTTACGTTCTATTTTTTTAATTACAAAACTCCAATAAGAAAGGAGTAAATGAATAACGCCATGTCAAGTTGTTTTAATTTCTGGATATCACTAAATTTAGTTTTATAGATAATTTTAATATGTTAAAATGGCCATGTAACGGGACAGAGCATGACATTGTGTGACTATAATCGAGTCACTAATTGTGTTGGATTTGTGATTGTATTTTGGTCGCATAATATAACGGGACAATGACTTAAAAATATGAGTAGTAATAATCTGTGACAAGTATGTTACGATTTTATCGCCACTAGTTGTGGCATAATTGTGACAGTATCGTCCGCCACGAAATGTGTCCATTTTTGCGACTATATTAACGTCGTCACAATTAGTAACGAATTGTTACTATTGTTAATCGTCACATTTGTTTGTGACTTTTTTGAGACCTTCATGGATTTGTGACGATTTTATTGTGACATTTTAAATTTCGTTTCAGTTTCGTCATAAAAAAATTATTGTGACCAAATATTTTTTTATTGTGACGTTAATCATCGTATCAATATTCAGTTTTTATTGTAGTGTAAGTGGATCCCAAATGGGGAAATGGGTAACCTTGAGTGGGTAAAGGGTCGGAATGTGTTTTTGAACCTCAGCTACTCTATGGGAATTCATTTCCATTGTGGCGTCTCCGAGCCGTGCCGACTGGCCGAGTTCTAGCGCGGACCGGGGGCGTTAGAATGGGTGGTATCAGAGCATGGTTTTAGGGCTTTTGGGATCCGTTTTTTTTTTCAAACGTTTTATCGAGTAAATAGTCGCAAAAGGCACTTAGGATAATCCGAGTCGTTCACCACTATTAGGATAGGGGGGAAATATTAGATACCTAATATCGTTGTTGGATTGGATGCAGATGTCAAGGCAGGTGTCGGACGAGAACTAGTCGAGAGATGTGATGTCTAGTCGTGGAAACTTTGCGCAGTATCCGGAGTCGGATAACTAGATACCGAGTGAGAGTGGTGAGATAAATGGAGATTATATTCACTTGGAGCCGTATGGATTACCTGAATGGGAAACCCCCGATTCCACGCCCAGGCACTCGTCGCCCGTATCGATGTTCTTCGATACATCAAATGAGAGTTACTTTCGTTCTCGATCACATAACCCCGTACAATCCCCATCGATCGAGCAAAACCATGGAAACCCTATCCCTGAAACCAAGAACCAAGGAGCCCCGAAATGCAGCAAACCTTAGACATTATTCAGAGTCAATTGGGTCATATGATTGAAACACAACAACATCAACAACAAATTCAAGAAAGCCAGCGAGAGGAATCAACATCCGCAAAGTTCCTGAAGCTCGTGATGATGATGAGAGACTTAGGAATTCGCGTGTTTAAAGGAGAACATAACACGGTCGTGGCAGACAAGTGGCTACGAGACCTTGAGATGAATTTTGAGATTTCTCGATGTCCTGACAATTTCAAGAGACAGATCGCGGTTAACTTCTTGGACAAGGATGCACGAATTTGGCGGGAGAGCGTAACTGCCAGAAACCAAGGCCAAATGATATCATGGGAAGCTTTTAGAAGAGAATTCGAAAAGAAGTACTTTCCACCGGAGGCCCGTGATCGATTGGAGCAGCAATTCATGAAGTTAGCACAAGAGACAAGGAGTGTCTGAGCATACGAACAAGCTTTCACGCGACTAAGGAGGTACTTATATAACGGACAAGATGATGAAGCATTGATGGTTCGCAGGTTCTTGAGAGGACTCAGGCTAGAGATATGGGGACGACTACAAGCGGTCAAATATGACAGTGTCAATGAGCTAACCGAACGGGCAGATAATGTGGAGGAGGGTATTGAGATGGAGAAGAGTGTAACCAACAAGGAAAATGATTGATCGGGAGGGAGGAAGACTAATAACGCGTAGGCCAAAAACAACGCTCCTAAATTCACACCAAATCAGGAGAGAGACAATGATCGAGGAAGAGCAAGAGCGAACTTCACAAGGTGTTTCATGTGTCTCAATTAAAAAGACATGTACCCAAACCCGATGCTGTAATTTCAGAATCGATACCCGAATTGAAGACCAACCTCACATATCCGGAGGGGCCACTCGAGATCGGAGAACGGCGCATTCGAAAGTTTAAGAAGCGGTTAATTCCTCAAGTTCAAGTAATTTGGGAAAACAAAACCGACGGTTAATTACATTGGAGGGTGAAAAAAAGTTCCGGGCAAAATATCCTCAACTGTTCGACGTTGAAGAAGAAGATGACAATGAAGCCAGTCCATCCAATACTCAGGGAATTTGGGACAAATTCCTATAAGGAGGGCAAATGTAGTACCCCGCGCATTGACCCCGGGCAAGAAAGAAGGGCAAGAGAAGAAGAAGAGAGAATGGTGCGCGAAATGAAAGGAAGGACGGGTATTTATAATCTGCGGAAGCTAGAGTAGAGCCCAATTCTTAAAACTTGGAGCCAAATCCATTCGGGCAAAGCCGACTTGGTGACCAAGTTTTGTCCTGAGCCATGCACGCCAACTTCGAGCCCACGCCTTAGCCAACTTGGAGCCGAATCCACCTGAGCGAGCCAACTTGGTGACCAAGTTTCGTCCCAAAGCCGACTTGGTGACCAAGTTTCGTCCCACTCCTCACATCCGAACTTTGAGCCTATAAATAGAACCTTACCCCCCTTTAGTTAAACCTTAGAAGCTTTCGCACCAAAACTCTGCCCAAACCGAGAGAGAGAGAGAGATTGTCTGAACCCTTAACCCGAGAGAGAGATTGTCCAAACCTTAAAACCCGAGAGAGAGAGAGAGTCCAAGATAGAGATCGCCAAGCCTTAGGATAGTCCAAGAGAGAGATCCCGAGTAGAAACCATCGGTCGAGTTAGGAAGATTCATCAGGGATTGTGAGTTCCGGTTTTTGTTTTGCATATTTGATTTTCCTTTTTGCATGTTCTCGATTCTCCCTTCTCTTAGTTTTGTTGTTGTTATGGTTGAGTTTGTTGAGGTTGTTAACCGAATAGCTAGGATAGTTGAGACCGAAGCACCTTGCCCTATAGGAGTGGTTGTGTTAGAATAACACCATTCACTTGAGAAGGATGGTTGCGTTGTCAAGACACTACTCGCTAGTATTCAGGAGACATTTAGAGAGAACTTTATGAGCATGTGTGTTGAAAACGAACCGCATATTTCATAGTGTTGATTGGAAGATCATAGTGTGTGGAGCGTGGAGAGTATCCAATTTAACTCCATAAGCATGCTACGGTCAAGTGGGATGTATCTGACCTTGCCATGTGAGTCCGTTCTTTCGTGGGGAGTGCTTGACGAGATGCAAGTGACCTCAAGGGGTTCGAAGGACCCAGAGAGACTGGGTTAGGTACCCAAGGCCCGGTGTCGCTTACACGACAGTGTAGCCAAGGTGATGTTCCGGTTATATGCCTTTGTGGTTATAACTCGGGTGTTCACGATATGTTGAGGAAGGCAGTGCATTCGGGCCCTAATTAAAACTTTAAGAAACGTTATCTCCTTGTTGTGTTGTGTGTTGCTTACTTCCTTCTTTGTGTACTCTGATTATTTATGTTATGCATGTGGGATTATGGGTGGGGATTAGACTAACGTTTCCTTGGGAAACCCGAGCTCATTGAGTAATGCTAGATTACTCATGACTCTACTTGTTTTGCAGGTAACCTTAGAGGGGATTAGAGGGAATGGTGAACACTAGGGAACCCGGAGTAGTTTTTCAGTGTCCTTTTGTAAAACTTAAAGTATGTAATTTTGTATCGTGTACTTTTGGGCCTCATGCCTTGGCCATAAAAACGATGTTTATGTTGTGGTTTTTTCTTGTATTTTCGTAACTCTAAGATAATGAAATCGGTATTTATTTTAAAGTATACGGGTTTCAAACTGATAAGAATTTGTCTGAGTTGACACAACAATTGGGTGAAGTGAGGGTTGGCAAGCCTAAATGGATCCCAAATGGGGAAATGGGTAACCTTCAGTGGGCTAAGGGTCGGAATGTGTTTAAATATGCATTTTTAATGAGTTCGTTTGTTCACCTTAGTAGATAGGCCGATTCTTATTACACACCTAAAAACCAGACTCATCAAATAATAGATCCAACTACATATACTTATGGTGGGCATATCTATTGTCTGGATTCAAGATCTAGTTAGTTACTCTAGATCTAGCATTAAGCATAATTAAAGATGAAGAATTCTACAGATAACCTAGCAAAGGGGGCAATCTACTAAACTATATGAATCCCTAATGAGAAACCCTATTCCTAACAAGCAGATTACTCAGACATATTGATTGAAGGAAACAACATAATTGAGTATGAAAACATAAACACGGCAAATAGATTTAAGGGAAAGAAGGGATCTCTTCACTGTATTAGGAACTGAATCAATCTCTGAAAACACTCGATGAATAGCTTATGTCTCTCAGTAACAGGGTTTGCAAAAAGCTTGATAAAAAACTTGATAATGAAAACTTAGGTCTAAACAATGTATATACACCCTCTAAAAACGTCTAGGGACTAATAATGTAAATAGAAAAGTTTTCTAGGGCAAATTTCCTCTTCTGTAAACTTGAAAGCGTCTAGGACTTTGCTGGGCCGAAACTGGTGTCGATCGACACTAGGAGTGTGTCGATCGACACTCCTCTTGATTCGTGAAACCAAAGTCGTCCTTACCTTACTTTTTCTTAGCTTTTGCTCCAAAATGTCTCCTTATCTCCATTGTTGTCCCACTGCATAGAATACCTGAAAAGACACTAAAAAGACTCGAGAAATAACATAAAGACTCAAGATCCTATACCTAAAACATAGATAAAATCAGTTAAAATAAGGATATATCAATCACCACAATCTACATATTCAATTTATAGGAAGTTCAACATTTGTAGTAGGATCCTCACTCTCACCTTCTTTATCTTTCTCATTTGACTTATCTTGATTCTCTATTTCCATGTTGTCCTCAGCTGGTTCAAAAGTACCCAAATATTCTTGCTCCACATCCGTCTGGTCATTCTGCTTTCTCTTTTTGGGACGATCCATTTACTTCACCTGAAAAGTTCAATCAAACATAAATAATGAAGAAGCCAGTCATTAGCCATTTAAATATCATGAAGTAGAACAAGGTCAATTTTCTCTCTCTACAAAGGCAATTTATTAGAGGTTAGGTCTCGGCCGAAAGGGCCTTTGCCTGGGAACTCGAAGTTTGTCCGACTGGTAGCCAGTTCCCACCTTTCCGTCCACACTGGTTCTCACACTTGTTCTCATCCACATGTGGAAGTCCGATCTACTATTGGGAATCCCTCTTCGATTGCTGTTTTTGATTCTCTTCCGGTTGAGACGCTGGGAACCTCTTCCCCTATTCCTAGAATTGTTGAATTTCCCTTTAGTGACGAATCTTTAAAGACTCCTTTACTTGCCGTTATCCCCTTCGACGACAAGCTCTCTTCGATAAAACATTATGTTTCTGGCGAAAGCTCCTCCGATTTGGCAACAATGTCTGAGAACTCCTCCACTATCGTCAGAGAGTCTGATTTGGTCCCTAATTCTCGTTTGGTCGAGGCAAAGGGCTATTCCACCCTATCACCTAACGTGGGGGCGTCAATGCCTATCGATACCTCTGGCCTGAGCCACGCTGTACCTTCGTCCATACCCTCTGAGCCTACCACAATATCTGAGAATTCCTCTGCTGCGGTGGATACAACTCTCCTGAAAAATCCTGCTAACACTGGATCAAGCTTTCCCACTAATTTTGGTGCCACTGCCACTCCTCTTCCGGTTAACAATCCTCCTCACACTTCCGCTACCCTTCCATGGGCGAAGAAATTTAAGGCCTCGTTGCTAAACCTTAAGCAAATGTCCAGCCCAACCTTTCTCGATGATGGTACCCCTGTTGTTGTTGCGCCACCCTCTGTATTACTGAAGACAGCAGAGATGTGGAAAGGCCATCTTATAGCCCAATTTTGGGGCCTTTGTCCACCGGATTCAAATATCTTCAATGATCTTAATCTTATACGATGAATTTATGGCGCCATCACGGTTCGTATTATCTCGGAAACAGCTTCTTTGATCTACATCTCGTCTTTGGCAACGCGAGAATGGGTAGTGGATGTGGGGTCCTTGTACAGTGTACCCTTGGTATCCAGAAGGCCCTCTAGAACTCGAAGAATTACAAACGGCTCCTACTTGTACAATTCTCAAGAACGACCTTCCGTAGTTGTGTTTACTTGATGGAATAATTGTTATAGCAACTGGAATTGGCGAGCCCCTGCATAGAGAGCCTTTCTTCACATTTACTCGACCTCGTGGTCGAGTACCTCAGTCTAGGCCACTCGATGACACCACTCGACCACGAGGTCGAGTAACACCTTGCCATACCACTCGATCATCACACGAACTGTCACTCGATCATAACACTCGATCACAACACTCGACCCCGATCGATTAGATTCCTGGCCCATCCCTTATCGGTTTACTTTGTTTTGTATAAATAAGTCTTCAAGAATTTTGTCCCAAAACATTTAGGTTTTGCTTCACTTTTGATTTCTATTTACCTAGTTTATTCTATCATTGCGCCGTTTGCTCTACACACTTGTAATTCGAGATTTTCCATGTTATTCGGATTCTACATTTGATTTTGTTTAGCACCTAGTTTCTCTACTCTTTATCTCTTTATATATGCAGTCTACTTGTTTATTGGCAATTATGTTTTTGAGCATGTAATCTGAATAGTGACTTAGAATTTTTAAGGATAGGATAGAATGGTTGGTTGATCTGTAGTCTATAGGATGGTTAAGTTTTAGAATTGATTGAAACCCTTCTGGATTAGTTGTGTTTAAGGCTTATTTCTCTCTGATAAATTGGAATTCGATCCAAAGCACTCCCGCACCCAGAAGGTGTTCGATAAATGCTTGATCCACTAGATCTAGAGGTTTTCGTCTCTATCCCAAGGGATTGGCCGTTTAGAGCGTTTATTGACTGTAATAGGTTGAATCGTATTGCCTGCATAGTTAGATTCTGTTAATGGGAATTAGTTTGTGCTAGCTTTGCTTGAGGGTTTCTATCACGATAGTGAATTAATATGTTGTTGAACTTGTTATGTAGGGATAGCTTGATTGCGCTTTGTTAACCATATGAGCAGGCTAAGGAGACCACTCCATTTGATTACCTCATCCTTAAGAATTTCTCGTCCATTTGATTTCTTTATTTCATTGCTATTGTTTAACCATTTTTTTTTGTGTTGCTTAGTTTTCACATTTTCTACCTATTATTACCAACTTACTCGACCACACCCAATGTGTGGCAGTAGATTGTGCGGTCGATTATAGTTGTTATAATTTCTGTTTGTTATTCGAACAGTTACTGGACCTGTCCCTGCTACTGGCTTCAACCAGTCGTGGTCGAGTGTTTTGTGTAATTCTGTTAGAATTTCTATTTTCTACATGTTCACTTAGGACTGCTAAAAACTCCAAAACCTGCTATTGCTTGGCTTGACTTAGTGGCTTCCGATCACATCTCATCTGTTTGCATCACACCTAAAGTGGATTTACACCTAAAATACTACAACGACACGATAGTGTTTAAGGATTAATTGATCTTAAAAACCTATTATCAATTTTGTGTCGTTGCCATTTGGGTGCTTGTTTGCTATATTGGAGATTTCAGAATATTTAAGATCCAGTTCTTTTTCGTTTTTATTCAACGTTACTAACTCTGTGTTTTTTTGTTTGTCTGATTTGATCCAGGTTTTGCGAATCACTAGCTTTACTCAACTTCCTCTGGACCGTGCTAACGTATGGTTGTTGCTCAGTGCATGCAAACTCGATCGCAAGGGCATCAGAACCTCCTTTTTAACGATAACATGAACCGTATTGCTCTTGAATTAAGGGAACAATTAAACACAAACGCTATGGCTGATGACCAGAACCCAGAATTACCAACCAACATTGGTGCAGGCGATGCACCATGCAACCACAATCACCGTAATGGCATGGTTCATGGCAACAAGTTTCACGACCTCCCATGAAGGATCCACTCGACCACCTAGACGAGTTTGATAGGCTCTGCAGCCTAACAAAGATCAATGGAGGCAATGAGGATGGCTTCAAGCTTAGATTGTTTCCATTTTCTCTAGGAGACAAAGCTCATCAGTGGGAAAAGTCGATTCCTCAAGGCTCAAATGATTGCAAGAAAGCCTTCTTTGCTAAATTCTTCTCAAACTCAAGGACTACAAGACTCCGGTATGAGATTTTTGGTTTCACCCAGAAAAATGCTGAGACTTTCTGTGAAGCATGGGAGCGCTTCAAGGGATATCAGACGCAACGTCCTCATCATGGTTTCTCTAAGGCATCACACCTAAGTACTCTCTACAGAGGCGTTCTTCCTAAGATCAGGATGCTCCTTGATACAACATCCAACGGAAATTTCCTGAACAAAGGCGTTCTTCCTAAGATCAGGATGCTCCTTGAGAACTTAGCTTAGTCTGATGGCAACTATAATGAAGCACCGCATGGATATGAAAGCTAGAAATGACAAGCTGGATAAGCTAATCATTGTGCAATAGAAGCAAGTCCATTTTCTCAGTGAAGATGAGACATTTCAAGTCCAGGATGGGGAGACTCTCCAATAAGAAGAAGTTAGTTATGTGCAAAATCAGGGAGGCTACAACAAAGGTTTCAACAACTTCAAGTAGAACAATCCTAATATGTTTTACAGAAGTAGCAATGTTGCAAACTCGCAGGAACAAGTCTACCCTCCATAGCAGCGAATCAACCCAAACCATTTGTTCCATACAACTAGGGTCAAGGGTTTATTCCTAAGGAACTATAACAGGGAAGCTATCAACCGCAACTTCCACCACCTGGGCTCACAAAGCAGCAACAACAACATGCTTCCACAATTCGAGACTCAGACTTGAAGAACATGCTCTAGCAGATTTTCCAAGGACAAGCAACATGAGCAATGGATTTGGCCAAAAAGATGGCCAAAATACACAACAAGGTTGATTGCACTTTGAATGATCTCAACATCAAGATTGAAGCACTAACGTCTAAGGTCAGATACATGGAAGGCCAAACTGGGTCGACCTGTGCTCCCAAAGTTACAGGACTTCCAAGGAAGTCAATACAAACCCTAAAGAGTATGCGATCGTTCACGCCATCACTATCTGCCAAGATCGAGAGCTACCCATTCGTCATGCCTCTAACTCAATCACTGAGGAAAACGAAGCTCAGGAAGGGCAGGTTTCTAATCGGATTGAAGTCTTAGTTATTGAACTTGACCATTCAGCTGGATCTCGCCATCAAATTCTGTCTACCTTAGAAGAGAAAGTAGCCATCATTGAGAGAATGGTAAAACGATCCAAGCCAACTCCATTACCTCCACTTGTTCTTCCAAGGAAGTTCAGGAAAGCTTGGATAGAGAAGTATGAATCTCTTGTATAGGAACAACTCAATGGGATCAAAGCAGTGATGTCATTAATATAAGTTTTCGAGCTGATTCAATACCCTCACAAAGACGTGAGAAATTTAATACTAGAAAGGATCAAGAGGTATCACGATTCAGATGATGAATATGATGCCACTCTGTTTCAAGCTGCTCGTGAAAAAATTGTTCAAGAAAAGTTGGAAGATTCTGGATCCTTCACTCTGCCATGCTCTATCATGCATTTAGCTTTCAACAATTATCTATGTGATCTTGGAGTTTCAGTCAGCTTAATGCCACTCTCTATGGTAAAGAAGCTTGGATTCCTTCAGTATATGCCTTGTGACATAACCTTGATTTGCTGATAGGAGTTCAAGGAGACCTTGACACACTAAGTCAAATTCAAGGAGGAGATTAACCTCGACTCAACTTTGATGTCCCTTCAGTTTGGTTTGAAGTAGACTATCTACCAGATGAGGAAGCAGCCTACTTAGAGGAAAAATGAATTGAGGACTCAGTTGCGCAGCTATCAATGGAGGATACTGAGAATGATGATAAGCTCGATGAAGAAAAGGACTTCTTAAGTCCTTTCTGTCACACTTTCCCTGCCTAATAAGTGTGAGTACTCAAGCTAGAGATTTAAAACAAACTCACTTGGGAGGAAATCCTATGTCTATCAATGTATATATCTTTTATTTTTTCCTTGTTGTCTTTGATGCATTTTGTTAGTATTTTCAGGAGATAAGTATAAAGAGTTAAGTGAAGTGGGTTTTACCTCTGAGTGTGCAACAGAACACTCGACCACATCCTGCAAGGAGCAAGTGTATCTGTGTAATCGAGTATAAAGAGGGTTTCACTCTCAAGATTTTGAAGTTTCAACATGTCACTTTATCACCCGAAGCCACATGCTAATGAGGAAATGTGGTCGAGTATATGTGATGATTTTGGACCTAATGTTTTTAAAAATCATCACTATACTTGACCAACAGAAGCCATATGGTGACTAGAGGATGAATTTGAGTATGATGTTGTTTTGATCCAAAAATTCAACATTATACTCGACCAACAAAATCTATAGAGACTTACAGAGGCTTAATCAAGTTTACAGGGGATTGCGGAATGTTCTGAGACTTTTCTAGTCAACAGGGAACCGTGTTTAAAGACAAGAAAACAGACGTGGGGTTCACCACCAAAACTTAATGTTCCCCATGCCTCCCACCAAAAGATAAGAAATCCATCTCTTTTGCTTCTCCACCAGATGTCAGACGGGCCGCCCTGCGCCCAAACGGGCCGGCCTGCGCCCAAACGGGCCATCCGCAATGTGCCCGATTCTCTTCTGATTCGCGACGGACCTGCCGGCCGACGACCGCGGTCTTGAAAAACGAGCCCTAACCCGCCCCGCAAAGCCCAGCGGTTAGACAGGCCGGCCCGCAGGCATATATGAAAAAGGGAGTCAAATATATGCCAAGAGAATTGTCACCTTGACATCTATGGAAAGAAAATTGTCAAAACATATAGTTAGGGCCATCACAATTCTCTGCGTTTACATGTCCAACTTCATTTGAACCACGAGCATTTTGATCTATACTTAAAAGGCAGCATGTGTTAAAACATTGTACAGACCACAATAGTTAACTAGATGTCAACTCCAAGACAAATTCACGTTTGTGTTACTGGTAAAAATAAAATGCCTCGAATACTGATGATAAAAATATTTCAACTTCATCACTTGGTTGGTATAAAACTAGCCACATACCAGGTTCATGTGAGTTCTCTGTGATTTCTTCTATATGATAGTAGGTTTTTACGGTCAATTAATCCTAAAATACTATCCTGTCGTTGTAGTATTTTAGGTGTCAATCCAAAAGGGTGTGATGCAGACAGATGAGATATGATCAGAAGTTGTTAAATCAAGCCAAGCAGAATTTGATTGGTTATCTAACAGTCCTAGTGTTGATGCAAAAAACAGTAAAAAGTCACAGAAATGACTGATATACTCGATTGCAGGGTCAACTGCCAGAAGGGACCCTGTTAGAGGTGTCAGACGGGCCGGCCCGCGCCCATCTTGCCCGCCCCGCAGTGGGCTTTCTTTTTCCGGCCGCGGCGGTCGGCCCGTTTAGGACCGCGGGCAAAAATATCAGTGTCCAAGCCCGGCCTGCAAAGCCAGCGGATATACGGGTCTGCCCGCGGGCATTTCTATTCACTTGTTTAAAAGATGAATCATTGAATACAATAATGTCTAAAGAATTTTGATTCAAACCTTTTATTCATGTATAACAATGTACAAACTAATATGGTACAATGCCTTTTATGAGTTACCTTTTACAAGTTCTGAATTGTGAATCATTGATTAAAACATCTAGTTACATACTTACATTTGACATCTCCTTTAACTCAAACGATGACATTTATTTAAACTGAAACCAAACATAAGTACTTAAACATAGCTACAGTTAACTGAAACACTTTTTCATTTACTTAAACTGAAACCAAACATAAGTACTTAAGTATATAAGACTGAAACAAAACATAACTACATAACCAACTAAACAAAAATACTGAAGTGAAACTAAACTAATGCTAAAAAGAGAACCTGCAACTCATATTCAAATCTGAAATTCAAACTCAATACGTAGAACAAATATCAATCCTCAAACCAAACATAAGTACATAACTAGCTAAACTCAATTCAAAACTGAAACTAAACTCAAGCTAAGAATAGAACGTGCAACTCATATTTAAATCAAAACTGAATGCAAACTCAATTAAAGACATAAGTGAAACAGAACCAAACTGAAACATAAATTTTAAACATAAGTCTGAATAAAAGAACTTAAGTCGAAGAGAAAAACCTACAACATCTCAAAGACAAACAGAAATCTGAAGTAAAGTTTCAAACTCTAACAGAACCTTAACTTAAACTCAAATCCAAATATCCCAAAAGGAACAATGTCGTGTCTCATCTTGCAACTCATTTGTAAACCTTAATCTGAATTTAAACTCATATTCAAATCTTAAAATCAATGTAGACTTAATGAAAGATACGTAGAACAAAGGATCATAAAAACAGAACCAAACTAAAACAAAAGTCAAGACTGAAACATAATTGTAACAAGTTAAACTCAAATGTGAAACAGAAAATGAACTTAACAAAATAAATAAAAAGTATATAGAGGGATAGTCATGGGACTTCCTCCCAAGTGAGCTTGTTTAGAGTCTCTAGCTTGACTCCTCACACTAATCAAGTGGAGAAAATGTGACAGAGGGAACTTGAGAAGTCCTCCTCTTCACTGATATCATCATCATACTCAGCATCCTCCCTTGACAGTTGCACAGCTGAGTACTCAATTCTTCTTTCCTCATAGTAGGCTGCTTCCTCCTTTGGAGGATAGTCTTTCTCTTCTGACCAATCTCAGGTGATCTCCTTTCTTGAAATCGTGTCATGGTTGAGCTGGGGCTGAACCTCCTTTTGCATTCGACTTAGTTTGTCTCTAAGCTTTTCAACAGTGTAGCTCAGCTTCTGAATAGTTCTGTTCTGCAGATCAGTCTTCCTTTTGAGTTCCAGCCAATCAAGTGAGGAGGGTATACTCGATCAAGTAGTCGAGTTCGATGGCGGAGTTTGAACCAAAATTTGCTCCTTCGCTCTTTTGATCTCAAGGCCTTCACTTGGAACCGCTCTCTGGACCTCTAAGGTTTTTCCTTCTATTGGTGGCCTTGTCGGAAATTTGTTGATGTCAAACTGTAACTTGACATGCCTCCCAAGGTTGAGATCAATCTTACCATATCTAACATCTATTATTGCTCCCACTGAGGCTAAGAAAGGTCTTCCTAAGATCAGTGGATCCTTAGACTCTTCATCCATTTCAAGCACGACAAAATCAGTGGGTACCTCAACTCCATCTATCATTACTGGCACGTTCTCTAGAATGCTGAAAGGTCTCCTTGAAGTTCTATCAGCAAGGATCAAGGTGATGTCACATGGATTGTACTGAACGAATCCCAGCTTCCTTGCCACAGAGAGTGGAATTAAACTGACTGAGGCTCCTAGATCACATAGACAATTGTTGAAAGTCAATTGCCTAATGGAACATGGCAGAGTGAAAGATCCAGGATCTTCCAGCTTCTCTTGAATAATCCTCTCACCAGCGGCTTGATGCATAATGACATCGTCTTCGTCATCTGAATCCTGATATATGTTGAGCCTTTCCAGTATCAGGTTTCTCACATCTTTGTGAGGGTCTGAAATCAACTTGAGGACTTCTACTAAGGGCATCACAACTTCCATCTCATCAAGTTGTTTTTCTGCTAGAGATTCATATCTGCTTTTCCAAGCTTTCCTCAACGTCCATGGAAGAGCAAGTGAGGGTGATGGAGCTGGCTTGAATCGTTTTACCATCCTCTCGATTATAGCAGTCTTCTCCTCCAAGTCAGACTGAGCCTGATAGAAAGATTGAGATGAATAGTCGATCTCAGCAACAGAGACTTCAATCTAAGTGGAAGCCTCCCCTTCTTGAATTACACTGTCCTCAGTGTTTGATGTAGAGACATGTTGAGTTGGCAGCTTTCGCTCATGTCTGATAGTGATTGCGTGTGCAGTGGCGTACTCCTTAGGGTTTTTGATTGCTTTTCCTGGAAGCTGGCCTGGGTTGTTTGTAACAGGTGGTGAAGTAGTGATGCCGTCCAGATACCGTACCCTTGTGTTCAATGATTCCAACCTGCTACTCAGTTCATTGAACTGTCTGTCAACTTTGTTGTTTAAGTCAGCTAGCTTCTTAGCAGATTCCATTACCCCTGCGGCTTGACCTTGCATGATCTGTTGGAGCATATTCTTGATGTCTGATTCCTGGGGTGTAGAGGGCTGATGTTGTTGTGGTGTGAAACCAGGTGGTGGCTGTTGCTGTTGATACCCTCCTTGGAACTGTTGCTTAGGAACGAACCCTTGACTTTGGTTGTAAGTAACAAACGGTTTGGGTTGGTTCTGCTGCAGCTGCTGTTGAGGGTAAACTTGGTCCTGAGGATTGGCGACATTTGTGCTTCTGTATGACAGATTTGGGTTGGGTCTGTAATTGTTGTACCCTTTGTTGTAGCCGCCTTGGTTCTGAACATAACTGATTTCAGCGCCCTGATCATTCTCCCCCTCTTGGATTTGGAATTCTTCGTCTTCAGAGGCGAAGTGAACATGCTTCTGCTGCACTTTGAGGAGTTTGTCAATTTTGTCATGGAGAGCTTTAATCTCTCGGCGGTGCTTGTCATCAGATTCAGTACTGGTGCGGATGCTTCTGTCATAATCCTCGTTGTAATTGCAATCCGACTGAGCCAAGTTTTCAACTAGCTCCCATCCGTCTTCGACATCCTTGTTCAGGAAATTCCCATTGGAGGCGGTGTCAAGGAGCATGCGTATCTTAGGCAGGACGCCTCTGTAAAGTGTGCTGAGAAGAGATGCTTCCTTAAATCCGTGATGGGGGCATTTGGTTTGATAACCCTTAAACCGCTCCCAAGCTTCACAGAAGCTTTCATTTTGCTTCTGAGTGAATCCGGATATCTCGTTCCAGAGTCTTGCAGTCCTGAAGTTGGAGAAGAATTTTGCCAAGAAGGTCCTTTTGCAGTCATCCCAGGTCGTGATTGATCCCTGGGGTAGCGTCTTTTCCCACATATGGGCTTTATCTCCAAGTGAAAATGGAAACAAGCGAAGCTTGAAACCATCTTCACTAACTCCATTGATTTTAGTAAGGCCACAGAGCCTTTCAAACTCGTCAAGATGATCTAGCGGATCCTCCATTGGCAGGCCATGAAACTTGTTCCCCTGAACCATTGCAATGAGACCGCTTTTGATCTCAAAGTTGTTGTTCTGTATTGGAGATGGAACAATTCCATGACGCTGGTTGTGGTTGTGAGGGAAGTCACCAGCACCAATGTTGGTAGGTTGCTCTTGCTCATCTACAACGTCAGCCATTGTGTCGGTTTCTGTCTGTTCTCTTAGTTGGCGAGCAATACGGTAGATGTTATCGTTGAATAGGAGATTCTGATTTCCTTGTGACCGTGTATGCATGCAACAGATAGCAGCCGGGCTATAACAGACGAATCAGAGATGCGTCTGGAAAGCGGGTTGCAAGGTACCTGAAACACAAAGACAGAAAAGGCACAATGTTAGTAACTTGTGTAACAAAAACGAACCTGATCTTAATGTTTCTAATGATCTCAAATATAGCAAACAAACATCAAACTGGCAACGGCGCGAAATTGATAGTAGGATTTTTGTCAATTAATCCCTAAAACATTATCTTGTCGTTGTAGTATTTTAGGATGTCAATCTAATCCGGTGTGATGCAAAAAGATGAGATATGATCAGAAAGCTCTAAGTCAAGCCAAGCAGATGGTTGGTTGGTTCTAACAGTCCTAGTATGCAATAACAGAAATGAAATGAAACTAAAAGCAGGAATGGCAATAGCAGGCAAGTGAATGACAAAGCAGAAATGATAACAGGCAATCAAATGGATAAACGATTCATAAGGATGGGGTAATCGAATAGTATGGTCTAATTAATCTATTCAGGCGGTTGACAAAACTTCACAGAAGCTATCCCTAGACAACAAGTTCAACAACAGATTAATTCACTCCCGTGATAGAAATCCTCAAGCAAAGCTAAACACTGACCTAAATCCCTTTAGCGATCTCTAACTAAGCAGGCATTATCGAATCAACATGTTAAAGTCAATACCCTCTCTAAAGCCAATCCCTTGGGGTAGAGTCAGATATCTCTGGAATTAATAGGATCCAACGCCCATTAAACGTCTTCTAAGCGCTGGACGTCTGGACTCATACTCCACATTAGCCAGTGAAATAAGCAATAAGCGCAACTAATCCAGAAGGGATTCTAGTTTTCTAAACTCAACCACCTAAAACGACCAAGTAAACCACAACTATTCTATCCCATCCTCAGAAATACAGTTCACTACTCAGACATAATCAAAGACAATAACAAAGCAACAATTGAAAACATGATAACAGAAATGACGAAAGACTTAAACGAAAACTGAATATCAAATACTTGAAAACGAAACTACAAAGTCGCAGAAAAAACACTGCAGCTATCAAAGTTGCGAAAATGTAAAAGCGAGATAAAAACGGATAATAGCGGATAATAGCCGACGCCCTAGGGAAAACCCTAAGAGGCAGTTTATATAAGCGAGAAATAAAACAAATAAGGAAACTAAATATATAGCGAATCTTCACGTCATGATCCTGCGACCGAATATGTCATTGGCATCTAACGGGCAGAAATGAGCTTCCTAGTGATCGAATATGTCATTGATCAAACTGGGCTTCAGGGGCTCCGTCTTGCGAAATGTAGTGAAGTCGAGATTGAATGGTCCAACGACCTTGTGATCGAGTGCGATCGAGTGGATGTCATGAGGGTGTGATCGAGTGGTTTCTTCGGCTTCTCTTCTCTTCTTTGTTCTCAGCTTGTCTGGTTGAGTGCAGAATGAGCAGATAGGCTTCCTGGTGATAGAGTGGTGATCGAGTGGTTCTGATGGGGTGATTCCTCCGGCTTCGCGATTGAGTGAAACTGAGCTGCTTGCTTCTCCGGGGTTGCGATAGAGTGATTTGCCCTGGCAGGGCTCCGAGGCTGCGGATAGAGTACTTTTTTCTGAAGGCGTCATTGGGCATTCTGACTTCATGTTCTGTATAATTAACTTAATTAATACTAAGTATAATTAACTAACCTCCTAATTAACTTAATTAATATGCGGATAAACTCGGACGATCTTATCCATACGGAATAGATAGGCATGAACGAACTTATCCATATTCGTTCCTATCTACTTCTCGTAGAACCGTAACCATGACGGGTTCACCCAACGCATCAACACATTAATCTCAGTCACCCAAGTCTCCGTTCCACTCGACTCACCCATCCGGTTCCCCGACCACTTGCTCATGAATATCTCCAAAACTGCCAGACCGTTGGTCCGACAAACAATACTCTTCCCATAAGGACTTTTTCCGACCGTTCCGATTGTACGGCTTGCTAAATCATCGAGTAAAATTCCTCGGCCTAGTCCTAGTCCTGGCCAGGGGTATTACAATATCTATCCCGACCTTTGAAATCATATAGCCGCTTATAGAATACTTTCTCCTCAGGCTATGACAAAGAAAAAGGTGCAGGTGGAAGATAGATTATTTTACCTCTTTTAATAGGATGTAAATCTTTTCTAACGCAAAAAAGTTGTAAATCTTTGCGAGTCTTTAACCCATTCTTTGGTTTCCCACAGTGCAACAATGCTTGCACACACAATTCTCTTAACATGTATGACATTGAAACTGTGTTTAACTGCCAGATCTTGAAAAATAAAATAAAAGTAAATATAGATAAATGCTAAACTTTGTAAGCATCCCAGTTACAACAATACTATTCAAAGAGATCACTATAGAAACATTCAATAAGGCAACTTGAAAAAGATAGACCTTTTCTTCAACCTTGATGGTCTTCCTCGTCGTTTTCTTCCTCTTCCTCTTCCTCTTCATCTCATTCACTTTCTTCACTTTCATCACATGAGGCTGTCTCAATTACAGTTTCAGTCCTAGCTCTCTTCTTCTCTGTCTCTTTCCTATTCCCAAAATCAATTTTGAATTTTTTTTAAGAGGACATATATATTATGACTAGTTAATATTTTAACCTTACTCTCATGTTCAAATTTTCAATCAAACCATGTTTTCTTCCCCTGATAACTGTGATTTGGTGGTAGACCATTGCGATGACACATATCAACATGCTTTCTACCTTTACATAATCACATATTGTCAATTTTTCCTACGTAAAGGACAACCCATCTTACCTTTTACTTTGCATACTACAAGATTTCCATATAGAGGGAAATCATTGATAGTCCATAGGAGCATTGCTTTAGGGTTGAAAGTACTCTTACTGTAGGCATCATATGTTAACTTTCCGTTGCTCCACAACTGGATAAGATCCTCTTTCAGGGGTTATAACTAAATATCAATGATATTACCAGGTTAATGCAGCCAGAAATCAACAAGGTAAGCATTAGCAGCATTATGTTTTCTTTTTTCATACACAGGTCTCGAGTAATATTATAATTAACTAGTTATATGTTCTTTTTTAATGCAGCAAAAGTAGGGTATTTGGCATTTACTTGCTTCCGAGTCACATAATCTACAGGGTGAAAAAAATTTCCGTCTTATTACTAAAATGGCACCTTAGGTCATTGGCTTGTTGCTCAGACCTAAACATTCTCTTTAGACGTGGTATAATTGGAAAGTACCTTAGAATTTTTCTGTGAAACATTTTTCTTTATTTGACCAGTGTGCATGTTTATCTTCCATCTTCTATCTAGAAGCTATGCATTTAGGACATGCGTCAAGCTTCTTCAGCCTTTTCATGAATAGATAATGATTATTAATAAAAGCATGAATATCTTTTAATAACCTATATCAAAAGATTTCAGGAACTTCTTCACATCATACATTGATGTATATAGAACATTATCTTCTAGTAAAATGTTTGGTAAAGTCTCCTGAAGATCATTGAAGCTTTTGTCTGACCACCCATTTTGTGTCTTTATTTTATACAGTGACACAATTGTAGAAAGCTTGCTATGGTTTGCACAACTTGGGTATAAAGGAGTTTCACATTGATAAGTCTGCATTTTACTTGTCTTAGGCATCCATTTCCCATCACTTTAGCATCCTATCACCACTGTTTCATACCATTTCGCAACATTTGTCATCACATTGCATGTTAAGGATAGTTTTGCATGCATATTACATATTTGTGTTGTTTTCAGGTGAATTGGAGCTGATGGTGAACTAATTGGAAGAAGTGGACCTGAACCGATCAACATACTCGACCATGAGCTCGAGTAGTGTCATCATGGACTTTTTGAAGTACTCGCCACAACCTCTACAGCTGATCACTCGACCACGACACTCTATCCACCACTCGACCCTTAGGTCGAGTAGAAGACTCACCACTTCTCACAACCACTCGACCCCGAGGTCGAGTAGCAGCCATCACCATTTCTCACCATCACTCGATCCAGTGGCCGAGCATCACCATCATCAATCACTCGATCGCACACTCGACCTCGTGGTCGAGTCCCCTCATCATCACTCACTCGATCCATCACTCTGCCACCAAGTTGAGTTCCACCATCATCCACCACTCGACATCATACTCGACTGCCAGCTTCAGAGTCTTCTTGATTCCGCAATCAACAAGACACTCGAGCATAAGGAAGAGATGAAGACTTTGGCTCGTCACTCGACCCACTACTTGATCACCTGGGTCGAGTACCATTCTTAATCTGTTCCAATACTGTGTCGTTTTGAGTATTAGGGTTTCAGAATATTTTGCTATAAGTAGCATGTACTTTATTTTTTCAAAAACAAGTTTTTATTCCGCACACAGTTTGTGTGTTATTTGCTTTTGTAATCCGAATTTCTTTTAATCTATTCAGTATTCGCTTTTTGTTTATTTTACTAAGTTGTTCATCCTGTTATCATCATGTTATTACTCTTTTGCTATGATGTTTTCATTCATGTCATCATTTATGGTCTCTATAATGATGTCTGAGTAGTGACTAGGTTTCTGAGGATGGGTTATAGTAGTTTAGAATCCTCGGTCTGTTAGGTGGTTGAGTTTGATTTACAGATCCCTTCTGGATTAGTTGTTCTTAATGCATATTGCTTTCTGATCAACTGGAATTTGAGCCCAGATATTTCTGCACCCAAAAGGTATTCGACGAAATGTTTGAACCACTAATTCCAGAGATTTGTGGCTCTGTACCAAGGTATTGGTTACAGGGAGCGTTTTGGCTTTAACTTGATGATTCCTAATGCCTCTTAGGTTAGCTCTCGTCAATGGTGATTGAGTCTCAGACTAGTTTAACTTGAGGGAGTCTGTTGCGGTGGCACTTAGATTTGGTTATCGAACTTATTATCTAGGGATAGTTTATTGATCATCTCAATCACTTCTAGATTGAGGAAACTAACTACTCGATTACCCCATCCTCGGGAATTTCTTATCCAATTGATTTATTTGCTTACACTGCTATTGTTCTCTTGTTCTTATTACTCGTTGCTTAGTTTTTGCATTTCATACTTGTTATCACTCATATACCCGATCACACCCAGTTGTCTAGTAAGAGATTGTGCAGTCGAGTATCTTTGTTACAGTTTCTGCTTGTTACTCGACCTGTCACTCAACCACACCTAGTGCTTGGCAACAAGCAGTGTTGGTCGAGTGTTTTCTTTATCCTACTAGATTTTCTGTTTCCTGCATGTTCACTTAGGAGTACTAGAAACTCAAAAACCTGTTATTGCTTGGCTTGACTCTGTGACCTCTGATGGCATCTTGATTGTTAGCATCACACCCATTTGGATTGACAACCTTCAGTAGTACAACAACATGATAGTGTTTTAGGATTAATTGATGTTAAAAACCTATTATCAAACATCATCTAGCTTTGCAAGGAACTCATCTTCCATCTTGTCCTCTACCTCACCATCAAGGTTTATGAGTTTGTAATCCATTTTCTTGCTATTTTGTTACGAATTTGAGTTTGATTTTTTCTGTAGAGTTGTTTATCTTGCTTAGATCTACATCATACTCAGTTTACAATGATTTCTGGGTTTTGTCCTTTTTCTAAATTTTGTATGATGATGTTCTTGAGTGTTCTTGAGTAGTGTTCTCTAGAGTTCTTGAATATGGGTTAGGTTTAGGTGGACTCCTCTGTTTATTTAGTTAGCTAAGGTTATAATTGCATGATTCCCTTCACGATTAGGAGTTCTTAGTGTTATTTCCTTTCTGATCAACTGGAGATTGATTTTAGACTTTTCACGCCCAAAAGGTGTTTGATGAAATGTGTGAACCAACTTATTCCAGAGATTTGCATACTCTACCCCAAGAGATTGGATGTTTAGAATGCTTATTGACTTTAATAGATTGTTGCTTAATGTTTGCTTTGTTATGTTTCATCAATGAGAATTGTGATTAGGAAGTGGTAAAGTTATGAGGATCATTGTCACGAGAGTGGATTGATCTGGGTTAATCTATTGTCTAGGGATAGCTTGATTGAGTTTGTTAGTTAGCTGAACCAATTACTCCATTCTAAAGAGCTACGTTTTGATTAGATTGATTTAAACTTCGTGCTATTACTCTTTGCTTGTTGTGTTTAGTTATCTTGCTCGACTTATTTACTTGACCAAATTTAGTATTATGCTCGGCCGAGTGCCTTACTTTCTGTTTAATATAGTTTCATTTTGAATCTGATTTGCATGATAACATGTTGCTTGGTTTAGTGGAAATAAACCAAACCTATTCACACTTGGCTTGACTTAGTATTCTCTCATTGCATCATGATTGTTAGCGAGACACTCATTTGGATTGACACCTTAAATACTACAATGACATGATAGTATAAAGGAATTGATCTTTTGGAAACACAGTAAAAACCAGACTATCAATTTGACGTTATTGCCATATGGGTGTTTACTTGTTACATTTAGGATTTCTGAGTTATTAAGATCAAGTTCTATTTCATCTTTCTCGAAACTCACTTCTCTTTTCATTTGCTTGTTTTTATCTTGTAAGTACTGTCATACTCCACTAGCACTTCGAGTGTAAGGATCGAGGAACTTCACCCAATGTATGCAAACTCCATTCTTAAGGTTTTGACAATCTTCTGCGGTATAGAGACAACATCGACATGATTCAGCGTAACTGAGAGAACAACAAGCCACATCAGATCTGGTTGTGATGGCGACTGAAGACAATACTAATGAGTTGCCAGGCAACATTGGTGTAGGAGATGCACCCATAAACCATCATCGAAGAGCTGATATTGTCCCACCTCCTATTCAGATTAACGACTTTGAAATTAAGAGTGGTCTGATGTCCATGATACAAGGTAACAAGTTTCATGGTCTGCCTATAGAGGACCCATAAGATCATCTTGAAAGCTTTGATATAGTGTGTAGTTTAATCAAGATCAATGGGTTCGTGAAGACAGCTTCAAGCTCAAGCTATTCCCTTTCTCACTTGGAGATAAAGCTCATGTTTGGGAAAAGACTTTGCATGTTGAATCCATATGCACATGGGATGACTGTTAGAAAGTTTTCCTAGCTAAGTTCTTCTCTAACTCAAAAACAGTTAGATTGAGGAATGAGATCTCGGGTTTCACACAGAAGCAATCTGAATCCTTCTCTGAAGTATGGGAACACTACAAGGGATACACTACTCAATGCCCTCATACTTCACCTATTCGAAACATGACGGCTATGCTTCAACAAAACCTAAAAAGTCAAACTACTAGTTCTTTGGTCTTGGAAAAACATATTATTGAGATAAACACTGATTAGGTTCAAATTACCCTAAGAACTATTATCTCAAATATTTAAGGATCGAATTTCACAAAGTTTTTTTGTTCACACAATGAATTAAATATCGAGATTAAGCTAGTAGGGAATGATTGAAAATAAAGGAGAACAAAGTAAAAAGACAGCAGATTAATTGGTTGTAAACGATTAAATAAAGAGTTAGGAACATGGTATTCTCAGGAAACTATTGGTTAGTAGATCTAATGAAAGCTAGGTTGTTATCGAACCATTCTTAAACTCAAACTCTAATTATGCAATAACCGGTGGTGTTCCGCCAAACTCCCTATGCTTATAGCTAAGAATAACCGGAGAAGCCGAGAGATCTTTAACCTAAACATGCATTCTAAACGAGTTCAATTGTTCACCTTACTAGATAGACCGATTCTTATTACACACCTATAAACCAGGTTAATCAAATAATAAATCCAATTACAGATACCTATGGTGGGCATATCTATTGTCTGGCTTCAAGATCTAGTTAATTTCTGTAGATCTACCATTAAGTATAATCAAAGATGAAGAATTCTACAGATAACCTAGAAAAGGAGGCAATATACTAAACCATATGAATCCCCAATGAGAAACCCTATTCCTAACAAGCAAACTACTCAGACATATTGAATGAAACAAACAACATAATTGAGTAACAAAGTATAAACACAGCAAATAAGATAAGAGTAAGAAAGGATCTCTTCACTCTATTGAAAACTGAATAAATCTCTGAAATATTGAAAACTGAATGAATCTCTGAAAACAAATGATGAATAGCTTATGTCTCTGAAGATAGGGATTGCAAAGAACTTGATAAAAGGAACTTAGGTCTAAACAACGACCTCTAAAACTATATATAAATCCTATAAAACGTCCATGGACTAATAATGCAAATAGGGAAGTCTTTTGGGGCAAATTTCCACTTTTGTAAACTTGAAAGCATAATGGACTTTGCTAGGCCGAAACTGGTGTCGATCGTCACCAGAAGTGTGTTGATCGACACTCTGCCTGAATCTGGAATCCGAAATCGTCATTAGCTTCCTTTTCCTTAACTTTTGCTCCAAAATGTCTCCTCATCTCCATTGTCGTCCCACTGCATAGAATACCTGAAAAGACACTAAAAGACTCGAGAAATAACATAAACACTAAAAATCCTATACCTAAAACGTAGCTAAAATCAGTAAAAATAGAGTTATATCAACTCCCCCAGACTTAGGTTTTGTTTGCCCTCAAGCAAAACACAAAAGCCGAACCCGTGGAAGAGGTTTTGAAAACAAAGGAACTTCAACATTCTCTAGCCTATTGCCATGATCATCCAAACTAAATCCACATGCCTAACAAGTCTAATCAAATCTTAACCAACATGTATTTCTCTCCAAGCTTACAGAACTTTCTAGATTCCATACCTAAGTTCCACAACTATATCCACCAGGCTTTCATCGATGGGTCTCATCAAACAAGCTAACATTCAAGATCCTTCTAGACTCATTCCTGAACTATACCATGATAAATCGACCATTCTCGCATACTGGATCAACACCTTCGACAGTTTTCCAACCTTTGTAATTGATCATTCCAAATAATGTATAATAATGGGGTCATAGGAAACATTCCTACCCCGATACACTATCCGAAAATCATGTCAATCTCTCATTTTCTTTTTATTTTTATAAGGGGAGAGGAACACATTCTTTCTATCACATTGGTCTCAATATCAAGTATGAACAAGGAGTCAAGATCTATCAATACTAACCGTTTGAAGAGGTAAAAGGAAGAGCAGTTAGATTAGCTCCGGCCTTAGTGGTTGTGTTGGAAACTAGGATATCCTCAAGTCTGTTGGTATGCCATCTAGATTTTCCAATAGTCGAATTGAGTCGAACCTGCAGCACTCATCAATCAGGCAATAGTTCATAACTAAACTAAACAAAACGTTTTTTTTTTTTAAGAGTAAGAAAATGAGTCAATAAACTCAAAAACAAAAATGATAAGAATAAAAAATAAAAGCAATATTGGTAAGAACCTCCCCCACACTTAAACGACACTGTCCCCAGTGGCATTCAAATCCGAAGAAGGCGGAAAAAATAAATCAAAAATAAAAGAAAAGAAAAGAAAAGAAAAGAATAATCAAACCAGTGGGTTACCTCCCACTAAGTGCTTGTTTTGAGTCAATAGCTTGACTTGGCAGAGTTCAGGATGTGGGTAGATTGGTGGAGGATTGTTGTTGCCTCCGCCCCCTCCATGGCGGTGTGTAAACTGAACCGGTCTAGGCAGCTGCATGTGTCGATCGACACTCAATGGTGTGTCGATCGACACTCTTCTCGTCACCTGCAAGAACAACAAGAGAAACAAGCATTCGAGAAACTCTAGGCTTGTTAACATGTGAAGCAGACTTCATAGAATCTAAGGAATTAATTGAAGGTAAAGCAGGAGAAGAGATAGAAGAGAACTTATCACAGAGGGCCTCAAGTGGTGTGGGCTGAAGAGGATGTGCAGAATCCTCTGTCTGTGAATCTCGAGGGTGTCGATCGACAGGAGGGATGGTGTCGATCGACACTGATGCTCTGCAACTCTCATTGGCGAGCTGAGCTGATGGGAGTGTTATGTGTGGCTCTGTTTTGGGATTCTGAGCAGTCAGAGGAGGTGTGTCTGTGCTCTAAACGTAGTTAGTAAGTGCCTCCCCCAAACTTAAACGACATTGTCCCCAGTGCATTCAAATCTGAAGTAAGCAAGGAAATATAAGAAAAATGTAAATAAAAAGAAAAGAAAACAAAACTCATACTAGTGGGTTGTCTCCCACTAAGCGCTTCTTTATAGTCAATAGCTTGACTTTGTCGAAGTGCAGATGTCAAGTGGATTAGATAGTGGCAGTGATTCCAGCTTGAAACGTAGCTATTGTAGATAATTCTGCTTAAACAAGTGATGGGGGTCGATCTACACTAGTGGTGGTGTCGATCGACACCACCACTAGCAGTCTGAAATCTGTTCCTAGACAATAACAGTCTTCCTTATGTCTCCCTGATTTTGTGGAGCCTTGCAGTGCTCCTCCTAAATCTGGTGCATATACTGTTGAGGCATTGGACAAGGAATTTTATGCGTGTAGACTTGTTCAACAACAGGTGGAAAGTCAAGGCTGATTGGTGCAGTCTGCGTCCAATGTCGATCTTTATACTTTTTTGAGGCATCCGTTAGTTAACTGGACGCATACTGCACCAATCAGCCTTGACTTTCCAAAAGTAAAAGGGGGACTTGCATCCGTTAGGTAAGCAATGCAATCGAATTTATACTTTTTTGGGACTCTACGAACCATTCTCCGTGGGTCCCACCCGCTACGTAGGGAAACTCCATCTAGTCGTAACGAGGCTTGATACATGATAAAAATGCGAAAACAACGTCTAAACAAATAATTTAATTCCCGTAATATCGTATGGATCACAAAAAAGCCCAAAAAATTATATAAAGGTCTAAATTTGGAGTAAAAAAATTTAAAAATATACTTACCATTGCCTTGCTTCTTTCGTCGGAGGGCTCTCATTGTGATTTCCGAAAAGCTATCACGTACCAAATTTTCCCTCACGGTTTAAAAGTTATAGAGTCAAAACGTTTTAACTAAAAAAAAAACAAAAAGGGATGAAAAAAACACGAAGACTTTTCAGGACCCACCCGCTACGTACGGATACTCCATCTAGTTTAAATGAGGCTTGATACATTAAAAAAATGCGAAAACAACGTCCGAACAAATAATTTTGTTTCTTAATATAGCACGGATCACGAAAAGACCCGGAAAAGTATCTAAAGGTCAAAATTTGGAGTAAAAAAAAGAAGGGTACTTACCATTGTCTTGCTTCTTTCGTCGGAGGGCTCTCATTGTGCTTTCCGAAAAGCTATCACATGCCAACTTTGGCCTCACGGTTTAAAAGTTATGAGTCAAAAAGTTTTAACATAAAAAAAAAGGGATGCAACACGAGGACTTCTCAGGAGGTCACCCATCCTAGTATTACTCTCGCCAAAGCACGTTTAACTGTGGAGTTCTGATGGGATCCGTTGCATTCGTTTTGGTATGATCACATCCGTTAGTACATGCTATGCAATCGTATTTATACTTTTTTGGCCACTCTAGGAACCATTCGCTGTGGGTCCCACCCGCTACGTAGCGATACTCCATCTAGTTTTAACGAGGCTTGGTACATGATAAAAATGCGAAAAAACGTCCGAACAAATAATTTTGTTTCGTAATATAACACAGATCACGAAAAGACCCGAAATAGTACCTAAAGGTCAAAATTTGGAGTCAAAAACAAACAATGGTACTTATCATTGCCTTGCTTCTTTCGTCGGATGGCTCTCATTGTGATTTCCGAAAAGTTATGACATGCCAAGTTTGGCCTTATGGTTTAAAAGTTATAAAGTCAAAAAGTTTTAACTAAAAAAAAAACAAAAGGGATGCAACACGAAAACTTCTCATGAGGTCACCCTCTAATACTACTCTCGTCCAAGCACGCTTAACTGCAGTGTTCTGATGGGATCCGGTGCATTAGTGCTGGTATGATCGCATCTGTTAGTACATGCAATGCAATCGTATTTATACTTTTTTGGCCACTCTAGGAAGCATTCTCCATGGGTCTCACCCGCTATATACGGATACTCCATCTAGTTTAACGAGGCTTGATACGTAATAAAAATGCGAAAACAACGTCCGAACAAATAATTTTGTTTCATAATGTATGGATCACGAAAAAGCTCGAAAACGTACCTAAAGGTCAAAATTTGGAGTCGAAAAAAAAATGGTACTTACCATTGCTTTGCTTCTTCGGAGGGCTCTCATTGTGATTTTCGAAAAGGTATCATATACCAATTTTTACCTCACGGTTTAAAAGTTATAAAGTCAAAAGGTTTTAACTAAAAAAAAACAAAAGGGATGCAACACGAGAACTTCTCGGAAGGTCACCCTTCTAGTACTACTCTCGACCAATCACGCTTAACTACAGTGTTCTGATAGGATCCGGTGCATCTTGATGAAATTTAGTTATTTTTATCATCACAAAAATATTAGAAAAAGTATAATAATGTTACATTTACAAAATTTCATCATTAATTCGTATTATTAATAAAAATGGATTTTATAGTTAATTTTTAGTACAAAATGATAATTAAATCTTTGTTTGGTATCTTTTTTTACTATTTAAATATTTTTTTGTTATTTTATACCTTATTTTGCATTGAGCTTTGAAATTTTAACAAATACTAAAATACTTTAAAAACATTTAGTAAACAAATAGAGTTAGTTAGCATTTAATAGTTTTTAAACAAATAAAATAACCTAATACAATATTTCTAATAATAAAACATATGTTACATTTAAAAAAAAACGCGGTTTTGTTAAACAAATGTTTTTGTTTGCTATTGTAATATATACTTAAAAAATATTGAAAAATGATAATGTATAATATATGTTGTGTAGGCAGAATTTTAAAAGTTTATTTAAATATTATAATCATTTTCCCTGTATATTCCATAAATATCAGATTTTTCATCATTTAATTAATTTCTTAGTTTTGGAAAAATTTAGATTAATATCTTAATGAATTATTTCTATTTTCAATTCAAGATTAAAACAAAATCAACGGTCAAGATTTTTGTCTTTTTAGAATATCAATAAACTGTTGAGATTATAAAGTGAAACCGGGTCAAAATTCGCATCAAAAAGATGTGTGTGTTTTGAAACTGTTTTTTCCATGAAATAAGTTTTTTTAACCCACTTGTATTTTTGTGATTACTTCAAACTTTTTGCCCATCTCACTAAACAAACTGCACAAATAATAGTATATATATATATATATATATATATATATATGCTTTATACAAATCTCTGCTTTTCTAATCCAAATCAATTCAAGATGCATAAATCGTTTGATCAAATTTAAATGATAAGACGCTATATATGATAATGAAAAAATGATAAAACCAAACTGTTTATCATCGTATCAAAAACCAATGAATGAGACATTCAAAACTTTAATAACCAAACCTACCTACATGTATAAGGCTCACCTTTTGATCCTCATCGGGTTTCTGCAAATTGACAAAACAAAGACTAAAATCCAAAATCAAAAATTTTAAAAAGAATTACTCATATTCTACAAATATAGAGAGGTGGAAAATCTTAACATATTACTTTGCATAAAAGATTCTACTATATTATTTGGGAAGTACATTTTAAATGTAACCTCAATTTTTGTAATTAATTACATGAAATGCCATTAGAAAAAATTAATCAAAAACAAAATCTTTTAATGACGTCATTAAGGTTACCAAAATCATTTAACGACAATATTCATTTCTTAATTATATGGCTTATCAGATCTAAAGACGGGTCATCAAAGATTTCCTAAACTGAAGCAAAATATACCGAATATTCAAATATCTATCTGTTACCAAATTAGAAACAAATAAGTTAATAAGTCAATAACTATTTGGAGGGACGGGTTTTTGAATCAACATTAATAAAAAAGTAAAATATAATTAATCCACCGTTTCAATACGGGTAAAATCTTTAATTTATTATTTTGTAAGACCACTAATATTAAACATATCAAATCAATCTAATTTATAAAAGGTTATATAAAACAAAAAATGTTATGTGGTATGTATAATGTTACTATATATAAAATTAAACTATAAAATATAAATGTATTAGGGAATGATACAATTTGCAAAACTTTTATATATAATAAATAATTCTTAAAATTTTTAAAAATACTACTTTAAAAACAAATCACAAGACGGGTAAAGAAATTACAGAACGGGTTTTATTTTGGAATTGAGTTATATGGTGGATGTATTTGAATCAATATTTATAAAATTTTAAGATATTATTAATATGCTGTTTTAATAAGGGTTAAAACTTCAGTTTTTAACAATTGTCTCAATAAAGAAATTACATAACAGGTTTTATTTTGGAATTGGGTTATATGGTGGATGTATTTGAATCAATATTTATAAAATTTTAAAATATTATTAATATACTGTTTTAATAAGGGTTAAAACTTCAGTTTTTTAACAATTGTCTCATGGATTCGTGGTATAGCGTTACTTAATAACAATTATAAACTGTAAAATAGAAATATTTTATAAAAATAAAATTTGTAAGTTTTAATATATATTACCTTTAAAAAATAAATCGTTTCGCGTGGGGGCAGCCTAAGCCTGATAGAGGCAGAAGAATTCCAACAATTTCTCCCGCTCCTCAAAAGTGAGTTGCTCCTCACGTACGCATGTTCTAATAGTCGAGTTGCTGTTGCTAAAGCACCTATGGTTCCATCCCTGGTAACTTCAACCGGTATACTGCGGGTTAAAATCTAGTAAATAATTAGAAAGGAAGACATACTTTAACAAAATCATTTGTGAATCTACATCATCATCTTCCACACTACCTGCATGAATATTAACAAAAAAGTATGTCAGAGAATATATGAAATTAAAATTTTTATAAAATATAAACAATATAAAAATAAATTACAGATTTAAAACGAAATTAAGGTTTGTAGTAGAATGCGCTATTTCACCTAGTGGAATGCATGGTCTTATATAGTACTGAAGAAGGAAGGCTCATTTCAGGGTTTTGAAAAGGAATATTCTGTTTAGTTTGTAGAAAAAAATTTTGATTTAAATTATTATAAATTTTTGAAACTTGATGAATTATAGAAATAATTAATGAGTTTCTTAAACATAAAGATTTAAAATCCCTAAATTGATGTGAAGTAATTTTTCAAATGTGTTGTAATTATTTTTCAAAATATGCTTAGGATTTTTCAAATTTAAACAATACATAATATTAGGAATGTTAACTAATAGAATCAATAAGTTCAAGATTTGTTAATGATTTTTTTGATGGGCTGAGGGGAGACAATTGTCAACCCCATTTTATTAAAAGAACTACAAAGAATTACATCAAAACTGCTCTAGGGAACGATGTCCCGTTAGCATCCTCAACCAAAAATAAAAGAACGTCCTCAGGACATATCTCAAAACAATGAAATCCTAGGGGTAAGAAGAAAGCATAGTTTGCTAACCCGTCAGCTAGACGGTTAGCTTCCCTATACACGTGTGACACTCGGACTAGCCAGTCCCTTGTGAAAAAGCCTTGGCACAACCGTACCAGGAAGGACAGCGGATGAGCCTTGCTGATCCCTGTGCTTAGAAACCCCACCACCAATTCTGAATCCAAATTTAACTCCACCCGTCGAAAGCCCTTATCCCAAGCAATGAGCAACCCATAGTAGGCACCCCATAACTCAGCTAACGGCGCATCACAAGACCCGATATTAAGTGCAAACCCACCGAGCCATTCCCCCTGCAGATTAAGGATTGCCCCACTTGCCGCAGCTAATCCCTGGTGTCCCCTCGACGCTCCATCTGTCGTCAGTTTGACCCACCTGTCGCTTGGTGCCTTCCATCTTATCATCCTTTCTACCCGAGCTCTCTTCACATGGTTATTTAACGTTCCCACATGAGCTTTCCGAACCTCCTCTGCTATATCCTTGATGAACTTCAATCTATCCCGACATAACTTGCGTTCCCCAAATACATCGCCACATCGCCACTTCCAAGCCCACCATATCCCCATACTAAAGAGCGTAGGCCAATCTCCCTTCGCCGGATCCAGATTTGTAAACAACCATTCAAATTGACTGAAAAACTCGTTCTGCCTTCTTTGTGGCAGCAGCCTTTGCCAAATAGGTGTCATCGCCGGACAGTCCCTCAACACATGTAAGATCGATTCATCAGCTCCATTACAAACCGAACAAGTTGCAATGTCACTTAAGTGTCTCCTCACACGCTCCACATTAGTCATAATAACCATATGACTCACCAACCAGATGAAAACTCGTACCCTCTCCGGCGCTACCAGCTTCCAAATTTGCTTTAAAAAACTCCCTATGAGTGGCCTCTCCTCAGCCTCAGGTTTCAGCAGCTCATAAGCGGACCGAACCGTGAAGTCCCCATTTTGCGTTCCCTTCCAAGATAACTCATCACTTGTCCCTGGGAAATCATGCACCACAATAGAAAATAATTTTCGCAAGATATTCTCCAGCAGGTACAGAGTTAGAGCTCCCATGTCCCACCCCGAGCCTGCAATCTAGTAATCTGCTGCTACCTTAGGAGCGCATCCGCCGATATTGATTCTTTGGCCACCGCCGAGAGTTGTTCCTGCAACAGCCAACGATCCGACCAAAAACTTATAGTTTTCCCATCTCCAGGGACCCATCTGACTCCCTTAGCCACCACTTCTCTAAGTCCAACTGTAACACTTCGCCACGTGGAGCTCCAGCTACTTTTAGGTTTCAGCCAGTTTGAATTTTGCATTTCTCCCACCTTGTACTTCTTGCTAAGGACTCTTGCCCAAAGTGTCTCCTTATCATGGAGAAGACGCCACCCTACTTTAGCAACCAACGCCTACTTCATCTTTGGGTTGACATATCTTATTCCAGGCTAAGAGGTGCTTCTTCTTCTTCTCCAAAGTACTCCCCCAAAGAAAATTATGTGACAAACAATCCAAGCTATCTAAAGTAGCAACAGGAAGTATTATTGCACTCATTACATGGACCGGTATGGTTGACAAAACCGCTTTTGTAAGCGTTATTCTCCATGCCAGACTCAAAGACCGACTCTTCCATCCCGCCAAGCTAGACGAAACGTGTTCCAAGACTTCCCCAGAAGTCTCTTTGTTCATTCTCTTCTGTAAGATAGGCATTCCCAAATACTTCCCCAACTCTCTAGTACAGCCAATCCCACTCTCTCCACTTATCAACCTTTCCATATCCCTACTCACATTATTGGAGAAAACGATCTTTGACTTCTCCAGGCTGACGTTTTGCCCTGAAGCCTCACAAAACTGTTCCAACACTCGTCGTATGATAAGAATTTGGGCTACAGACGCTTCTGCACATAGAATCAAAGTCGTATGCAAAACAAATGTGTGATAGTTTCTACCCACCACACGAGAGTGCCATCGGTTTCCAGTCCCATGTTCCCACTGATGCCTCTATCAAGTGACAAAGCCTCTCAAGGCAAAGAACGAACAAATATGGTGACAAAGGATCCCCTTGTCTCAAACCCCTAGCAGGAACAAAAGAATCAGTTTTCCCTCCATTCCATAACACACTCATCGATGGGTCTGTTACCCCAGCCATTATTCTGTGAGTCCAGACCTCCGACAGTCCAGTAGCCACCAAAGTTTCCTGCAGAAAATTCCATCGAATTCGGTCATAGGCCTTTTCAAGATCCAATTTTAACAACATCCACCCTTTCCTTCCTTTCTTCCTCCGCATAGAGTGAACCGCTTCTTGCACTAACACAATATTATCAATGCTTAATCGCCCTGGGATAAAATTCGCTTGAGCCGGCCCAATCAGCTTCAAAATAACCTTCTTTAGCCGGTTAACTATCATCTTAGTAATAATCTTAAACAAGACATTACATAGACTCATTGGTCTAAACTGTGTAATCCGCTCTGGCTTAGCCACTTTGGCTATCAACACAAGCAAAGCATCATTAGTAGCCGCTGATAGCACCCCAGTTTCAAAGAAATCCAGTACAAAGCGTATCACCGACGGTCCCACAGTCTCCTAACATTGCTGATAAAAAACAGGCTGAAAACCATACGGGCCTGATGCCTTAAAACGTCCCATACCTTTTACTGCAACGACGACTTCCTCAGAAGTAAAAGGTTTGAGCAACGCAGCCTTTTCCCCGTCCGTTAGTGTTGCAAACCCGCCTATTGGCAACATGTTCCATACTGCGCTAACATCCTCCAAAGAGTATAGCCTCGTATAGTAGTTCAACGCCATTGTTTCCAACTCCACCTTATCCGTCATCCATCTGTTATCGCCATCTTTCGGCGATTCTATTCGATTCCTTTTTCTGCGGATAATTGTGGAAGTGTTGTATTTTTGTCTCCCAACTCAATATACTTTTCTCTTGACTTCTGAAACCACAGCGTCTCCTCTTGTTCCAGAACCAAGTCCAAGTCTTTCAATAATATCTCTTCTTGAGCAAGCAACTCATCGGAGTGCATCACATCCAACAAATCCTGTACTGCCTTAATGTCAGCCACCAACTTCTCTTTTTTGACATGAATGTCTCCAAAGACCTCCCTGTTCCATTTCTTTAACTTTCGTTGCAGCCCAAGTAACGCCTCTGTCGTCGACAAAGCTGTATCCCAAGACGCTGTCAACAACTCTTTGAATCCCCCATGACTCAACCAGGTTGCCTCAAATCTGAATGCTCGTCATCGTGGATCTAACTTCTGATACGGCTCCAACTGAACATATAGTGGAGCATGATCCGAGGACATGAAAGGTAAGTGAGAGACCACAGCCTCTTGCCACTTTTGCCTAGCATGGGCACAGCCCAAAACTCTATCTAGCCGTTTAGCCACCATCGTGCTCTCTTGTCTACCCCTCCTCCATGTAAACTTATTACCTCTGAATCCGAAATCAATCAAAGACAACTAATTGATCCAGTCTCCAAAAGCTAAAAAATCTGGTGAAAGCCTCCCATTTCCACCCATTCTCTCATCCACTCTCAAGATGGTGATAAAGTCTCCTCCAATTATCAACAGCCCTACCAAACCAGATACAACTTCCTTCAACTCTCCCCATAAACCACTCCGCCGACTCATAGATGGCGCCGCATAAACCACTATTAGATTCATCGGATCCCCATTACAGACCACCTTAGCGTGGATATATTAGCTTGTTGACACCACAATATCCACTTCCCCCACACTCGACTTCCATAGCACCCACAAGCCCCCACTCTGACCTGTTGCATCCACCCTATATGCATTCTCAAACCCCAGCTTCTGACAAATTCTACCTGCTTTTTCACCCCCCCCCCCCCCCCCGCGTGAGTCTCAAAAAGCGCTAGTACCTCCGTATCAAATTTCTTTAGCATATACCGTATCGATCTTCTAAAGATGGATTTATTCGCCCCCCAACAATTCCAAAGCATACAATTCATGATGACTTCACATAAACTCATAAGAACTACCGGGGAGTACCATTACTTCCCTCCGCTACTGATACTATGGCTGCATGCCCCTTCTTTCGGGCCAAGATCCATCGACGCACTTCCACTAGCTGGTGATCTGACCTCCTCTCCATCTCTCTGATTCCCAACTGGCAGCTGTGCATTTCCTAAATTCCTGCTACTCCCATTAGAAAACGCACCTCCAGGTCTGCCTACATTCTCATGCTCCACCCGCAGCCTCTTCCTAGGTGACACTTCTGTTTGCTCTCCTCTGATCGTTCCAAACACCAAACCTCTGGTAGCGATCGTATTCCTTCCCCCTTTACCTTTCCGTCCCCCTCCTTTCATTGGTTTATAGTGACCAGCTCTCCTATCTGTTATTACCCCAAGCCTTCCTTTTCCTTCCACTAACTTCCCATCAGGCCCACCTAACCTCACAACGCTGGTTAACGCAGCCCCATCCCCCTCATTCTCTTTATTTTCTTCCCCTGAGCTATCTGGCACCAGCATCCCTTGTTCCTCTTCTCTCTTTCCACACTCTTTCCTGATCTCTTCTCCGCCGATCCCCCTGCTGGAAACGTCATTCCCTTTCCTTGGACTGGCCCCCGCCTGGACAGTATACGTACCTCCGTAAACCCATCACTGGGTTGGTCCCCACCACTTGGACTAGCACCACTCTCAGTACTGGTGACCTGCACCACCGGACCCGGACTCCTCTTAGGACAAGCTCCAGCCAAATGCCCAAAAACTCCACACAAAGAGCAGATAACATTCAAACCCTCATAAGAAGCATAGTAGCATTCCCCATTCACCACCACTGTGCCTTTCAAAGGTTGCTTCAGGTTGACCTTAACACAGACCCGCGCAAAATGTGCTCTCTCCTTCCTCAGAATAGTTAAATTCACCTTAATCGGTTTCCCTAAGCCCTCTACTATCGCGGTCAAAATTGTCTCATGGTAGTAATTGACAGGCAGGTTTGAGATGCGAACCCACACCAAAGTGGTCACGATCTCGTCACGGATTGGATTGAAGTCCGGCGACCACGCCCGAATCATTAATATACTGCCTAGTACCCTCCACGGATCCCCAATAGCCGCTGCTAAATAATCCTCCTCCAAATCAAATTTCACCATGAAGAAGCTTCTAGGGAGATTCAACACTGACATCCCCCCATTTGGCTTCCATAGCTCTCTCAATCGCCGATTTAGGTCTGCAATCGTGATGTGGCGTCCTAAGACTTTGACAACCAGACACTGTAGGTACAAATCGTTCATCGCGTCGAGGACTTCCTGTCCAATCGTGATGATCGCTTCACCTTCTGCCCCAGTCAGAAACTCGACATTCATCCTCGAAGCCACAAACTCTGCACTCAATCTCGTCTCAGGAATTGGAATTCCTCCACAGTAGCAACTTTGAACCTTATCGACCCACTACGGCGACCCCGTTAAACCCCCTCCCGGCGGCCGGCCGTTCTCCTCCATGTGCTCATCGAGATCTCCTGGATTCACGACCCTAGCGGCGCTAGGGTTTTCTATCGACATCGCCTTTTCAATCTTTTCGATTGAATTATGGAAACAGCTTTCTAAAAAAGATTTTAAGATTTGTTAATGATTGAGTTATAACTACAGAGCTAATTCGGTAGAGTCTTAAAGAAAATACTGTTATTTTCTTTCTTTATTTAATCAATTTTTACACCAACTTTCCTAATTAAAACCATTTGTTTCTTTTTAAAATCAATTTCTTTGAATGATTGTTTAACGATTTGATTTAAAACCAAAGTTTATGATAATTAATTATTTTATGATTGTAATATTTAAAAATATTTCCTAAATATCTCAAAGAAATCATAAATGCTAAATCAGTTTTGATAATATTTATCTATTTTAATTATACAAAAATATTTAAGAAATATAATAATATTATATTATTACTGTAATCATCGTTTGTTATCTCTTTATTACTATTTAAATATTAGTTGTTATTTTATAATTTATTTACTATTTAGTTTTGAAACTTTGACAAATACTAAAAAACTAAATGAAAACTTCGTGAACAAATTGAATTAGTTAGCATTTAAGGTTTTTTGAAAAAACAATAAGAATAATCCGGTACAATATTTCTAATTAAAAACATATGTTACATTTAAAAAACAACATATCTGGTTTTTGTTAAGTAAAGTTTTTGTTTGATATTTTAATATCTACATAAAAAATATTTATTGAAAGGTGATAATGTGATTGGATAAGTAATATTTTAAAATATAATAAGTATTATAAATCCCTATAGTATAGGAGTATAATTAGTTTTCTAATTTGAAACCCACGTAATTATGAATGGTATTGAATAAGAAAATAAATTAATTAAAAAAAAAAGAAAGCTTTTTCTTCCCAAAATCTGGCTGTATAATTTGATTTTTACTGAAAAAGTCAATGGCTACTATGAAATGTAATGGATGACTAAGATTAGAAGTTATGACCCATTATTTTAAAATCCGGGTCAAAAGGATCCGCGCGTTTTGAAACGAGTTTTTTTCATTACAGAAATTTTTTAACTCAATGGCAAACTTGTTATTTCTTCTTGATAGTCTTGTTATTTGTAACTCCAAATTAAGATAATCAAATTAAATTCATCGATTGAATAATAAATTATGTACTTTATTTTGAGAGGAGTTAAAAAATTAATATATAAATAATGTGGACTTTTATGTTCCCATCCATTATCCATTTATATATGTTTTCTTTTGTGTGTTTTACACTTATCACAAAAAAGGGTGTATTGAACTTAGTATTTTGTAAGATTTTGTGGATTTTAGATTTGGAGAGATTTTAAAAGACGGCTAGTGATTTGGTGGATTTTTAGAAGAAATAAAACAAAATTTAAGTGGTCATTGAAAAAAAAGTTAGTGATCATTTAGTGATATTTGATGTCAAATTCCATAAGATTTTGAAGTATTTTGTGTGATAGTCCAAAATTTTTTTTGCTAACTATTTAAAAACTCATAAACGTTTCTACGTGTGAATGAAAAATTCAAAATTTGGAGTTGCACATTGGCGAATTCCATTAATTACAAAAAAGTATCAGAATTATAAAGTTTCATTCACTAATCCATTTTTTATTTAACATTCCATTTGCTAGTCCATCCATTTCGAAATTTTGCAATAATACGGGGGTATATTCGTCATTGTATGTTAGTATTTTTTAGAGAATTGATAAGTTTATAATTTGCATGTTTTACACATCCTTTTATCACCATTTTGAGTCTAGTTTCATCCATCTTTCATAGCATTCACCATCATTTTTGTACATTTTGCATCTTTAGTCGAGTCTAGGACTAGTCTTGCATACATTCTGCATTTTGGAGTTGTTTCAGGTGTTTTGGAGCTATTGGTGCTCAGAAAGAGGGAAAACAGAACAGAAAGTCACACTTCCACCAAACATACTCGATCACCAGTAGCAGAGAGGGAAGCAGTCAGCTCAGCAGTCCATCTAGACACTTCTACTCGATCATAGACGAGACCAGGCAGTCCATTCTGCCAGATATACTCGACCGCAGAGGAGAGGAGGCAGTCCATTCTGCAATTCTACTCTATCGCAGAGGCGACCAGGCAGTCCACTTTGCCAGTCCACCAGTTATACTCGATCGCAGAGGAGAAGAACCAGTCCATCCTGACAGCTGACACACTTACTCGATCACAGAGGGCAAGAAGCAGCCCGGACCATTCTGTCAACCAACCTACTCGACCACCAGCTCGACCCTTTGATCAATCCGCACTCAACCAGACAACCTGAGCATCGGAAAGAGAAGAGAAGAGAAGGCCCATTCGAAGCCCATTCGAAGCCCATCAGGAAGCCCACTTTGAGCCGACACTCTTGATCACACTGTCCATGGGCCGTAAGTGGGCTCGAAGCCCGCTATCTTATCTTAGTATAAATAAGTCTCTAAGGGTTTCGCCCTGAGACATGCACTTTAGAGACACGAATTTCGCACCATTATTCAGCAGCCGCTTTTCATTCCTACAACTTTGTATTTCGATTTTTCTTCTAAGTTATTCAGAATTTGCTTTCATTTTGCTTGTTCATCTTTCGTTTATATGTTATTGTCTTTAAGCATGTTGTCTGAGTAGTGATTAGGATTTCTGAGGATGGGATAGAGTAGTTGTGGAACCCCTGGTCGAATCTGTTGGTTAAGTGTAGATTTTTTGAATCCCTTGTGGATTAGTTGTGTTTACTGCTTATTCCTTTCTGATCAACTGGAATCCGATCCCAGACGTTCCAGCGCCCAGAAGGCGTTTAATGGAACGTTGGATCTACTAGTTCCAGTGATAATCGTCTCTATCCCAAGGGATTGGCCGTTTAGAGCGGTTATTGGCTTTAACAGTCGGTTCTTATTGTCTGCTTAGTTAGAGATCGCTAATGGGAATTAGGCCTGTGTCTAGCTATGCTTGAGGATTTCTATCACGGGAGTAAATTAATCTGTTGTTGAACCTGTTGTCTAGGGATAGCTTGATTGAGATTTGTTAACCAACGGAATTGATCAGGGAGACCACACTATCCGATTATCCCATCCTTAGGGATTTCTCTGTTTACTTACCTGCTTCATTCACTTGCTATTGGATTTTAGTATTTTTTCTATTTTCAACCATTCTACTCGATCACACCAGTCTACTAACAGTGAGACTGCGTGATAGAGTAAACTGGTCCATTTCTGCATATTCACTCTCTGTCATCGTGTTGTACTCGATCACAGGGTCCCTTCTGGCAGTCGACCCTGCGATCGAGTATATCAGTCATTTCTGTCATTTTTTACTGTTTTCTGCATCAACACTAGGACTGTTAGAAAACCAACCAAATTCTGCCTGGCTTGACTTAGCAACTTCTGATCATATCTCATCTGTCTGCATCACACCCATTTGGATTGACATCTAAAATACTACAACGACAGGATAGTGTTTTAGGATTAATTGACTAAAAAACCTACTATCAAAAATATATACAAACTAACCATGATAATGAAAAGAATCAATAAATTAACTATGATATACAATGTTTTGTCAGAGGGTAGATGAAACGACCATTAAACCTTTCCTTTCAATTTATATGTTTTATCGTCATCAACAACATCTTATTTCTCACCGTCATCAACACAACATCCACCACCATCCTCAACATCACCACCAACAGTCAAGATTTAATTGCCCACCACCCTCACTCCAACCAAATCAATATTCCTTCTATTGTCATTAACATAACCTCCACCACCAGGGACGGCCCAAACCATTTTTCATGCCTAAAGCAAACAATACTTTAAGCCTACTAATAAACAAAACTTAAATGAAAAAATCCAAAAAAAAAAGAAAAAAGGAACTCGAACCAGTCATCTCATGGACGTTTTAGTACAGTTACACGACTATGCCACAAGTTAAATTTGAAATGATGACCTCAAAACTTTTACAATATATTTGTTATGCCTAAAGCACCAGATTCATGAGCTTTAGCCTTGGACCGGGCCTATCCACCATCATCCTCGACACCACCGCCACCATGCTCTTGACCACTGCCACCACCATCCTCGACACCACCGCCACAATGCTTTAATAACTAATATAGAGATATTTATCAATATTTCATGCTATTCAATAAAATCGAATAAATTCTTGCAATTATGTTCTATATATTTCATGCTATTCAACAATATCACATGAAATCTTGTAATTATGTTCTATATAATTTCATGAGATTCAACAAAATTACGTGAATTTTCACAATAATGTCCAAATTTCTCAATATACCATGCTATTCAATAAAATTGAGTGAAATCTTTCACAACAATGTTCATATTTGGCAATATATCATATTATTCACCATAATCATATTCATTTTTGCAATAATGCCTCCTCAACCCTCGTACATTTTCTCTGCTACCTATTGTTCTCTAATTCTGAAAAAATATAGAAATGATTATTTGGTAAATGTCCCTCTTCTAAAAAATCAGTCTATGGTTAATTTCTGATAGAAAACAAGTCAAACATTATTTTCTTAACTATTTTACAGTTAAAAGGTTATTCAGTAATATTACCCTATTTTTTATTTTCTTAGGTCATCTTCTCAGTCCTTAAGTAAATTACTCATTTTCGCATCAAATCGATTTTGTTTGTTCTCTGCTTTTTGGTTTAGGGTTTTAGCTTTTCAAAGTTAAAACCTTTTTTCCCCCCGAAGAAGAATCAATACGACCGATCAAATCAATCCATTGAATCTATGGCGATGGCTATGAGACTTCCAGCGATATCTAGAGCTGTGACGGAAGTAGCTTCGTCTCCGGTTGGTTTGAGACGACTGTTCTGTTCAAATGCTTCAAGGTTCTCTTTTCTGTCTCCACCGGCTCGTCGTCAGGCTGAACCTAGCACCAATCTCTTCCACTCCGGTAACAAACCCTTTTCATCTCTCAGTTAATTTCTGGTTCTTTCGAGGGTTTATCGTTGTTCGAGTATATGTTTGATGCTTTTTAAAGATCCTTCCTTTATGAATTCAGATTTAGATTATCAGGTTCGTCTAAGATGAACTATATGTGTTTAAGAATTTGATAAGTTCAATTATTCATTTTTGGTTCTGATTTCCAGAAAAATGTCTGCTTTCGATTGCTTAACTCTAGAAATGTATTGTAAGAGTTAAAGAGAGTTTCTTTGCACTCAATTACTTCAGAATTCGGTTTGTTCGGTTAAAATTTGGATGCTTACGTGATGATATGTGTACCGGATCAATGTCTTGTTTCAAGGGTCTATGTGGGCTGTTTCTAGCATCTTCAAGGCTTCTGTTTTTGTAAACTACTTCCTAGTATCAATTAGTTTTTGAAATACTGCTTAACATTGGGCTTTGATGCAAGCAATTTTGATTAGGCTCTTACTTTTGTTCCTTCATAGCCTCTGTTTCTCACTTTTATCACATGGCCATTATCACATACATACATCTAAGGAATGTTATGTGTTCGATACTGTGTCTCAGTTTCGTGAAAGTGTCTTTTTGTCATGTTATCTTTATCTTTGTGTCACTGGATATTTCAGGGCTTAGTAAGCGCATCACTTCTGAAAGGTCCTTATGGAACCGAATATTCTCTCGGAACATGGGTGGTGGTCCGAGAACTTTCCCTGGAGGACTAAACAAGTGGCAATGGAAACGTATGCACGAGAAGAAAGCAAGAGAGAAAGAGAACAAGCTCTTGGATCAAGAGAAACAGCTTTACGAAGCTCGAATTCGGACCGAGATCCGAGCTAAAATGTGGGGTCATCCAGATTCCGGTGAGAAAACGGCGAAATTGAAGCAGAGTCATGGACCAATGAGCCCGAAAGAACATATCAAAACCTTGGCTGATCGGTTTATGAAAGCTGGTGCAGATGATTTGTGGAACGATAACGATGGTCCGGTGAAGAAATTTGATCAAGGGTCGAGATCATGTTCGGATTCAATTGATTCTACACCTATTGATGTGAGGAGATTAGTTTCTGCGACATGTGATTCAATGGGTAAACATCGTGTTTTGGATAGTAGTAGGAGAGGTTTTTCGAGTATGAGTAGATTTAAGAGAAATGAGAGTTCTTGTGATGAAGGAGATGACGTTGATGCTAAAAAGTTAGATACTTTGAGTCCTTTTTCTCCTAAATTTTCTGGTACCAAGGAGAAAGTGAAGAGCTCGACGAGTGTTGTTGGAGTTATAAGGAACAAAGGTTTGTTTGGAAGAAGGAAGTTTAGGAAGAACGATAGTTCAACTGAAGAGGATTCAGATGAAGAAGGCAATGAGGGAAAGATGATAGGTTGGATGGATTTGAGGAAAACAGGAAGCAGTGCGTCGTTAGGAAACCACGATATCAAGCTGACGAAAAGAGTGAATCGTAATGTCACAGACGAAGAGCTTTATCCTCCTTTGGATATTAATAGAGTTAGAGAAGATCTCAGTAAAAAACAATCTGTGGATAATGTAATGGAGGAGAAGCAAGAGCCTCATGACTCGATTTACAGTGCAAAAAGGTATTTTAAACTCGCTGTGTAAAGGATTTTGATTTGTCAAGTTTGAATCTTGATGAGCTCTGTTAATGGTTGCAGATTTGATGAGTCTTGTATATCTCCTTTGACGCTTAAGGCGCTTTCTGCGTCTGGCATTGTTAAGATGACTAGAGTGCAGGATGCCACTCTCTCTGAGTGCCTAGACGGTCTGTGTTCTTCTAGTTCTTTTGGCTCAAATGGTTTGCATTTGACAACGTATTATCCTATATGTAACGCAGGTAAAGATGCATTAGTCAAAGCCAAAACCGGGACGGGAAAGAGCATGGCGTTTTTGGTATTTTACAATCCTTTCTCCTGAGATTATGCCTTAGCTTTTGTTGTTTCTTAAGATAACGTTGTAAGACTGAAAAAACAGCTTCCTGCAATTGAAACAGTTCTGAAAGCTATGAACAGCGGTAAGGGCGTTCACAAAGTTGCTCCGATTTTTGTTCTTATCCTCTGTCCTACAAGAGAACTTGCAAGCCAGATCGCTGCAGAGGGCAAGGCTCTGCTCAAAAACCATGACGGGATCGGTGTTCAGACTCTAATTGGTGGTACGCGCTTCAGACTTGATCAACAACGCCTAGAATCTGAGCCATGCCAGGTTTGACCAACCGTGATTCCCTGTTTAACAGGAATTGTTTATATTTTCTAGTTGTTGTGGTGAATCTTCTTTTGTTTCCGTACCTTGTTGCAGATACTAATAGCAACTCCGGGAAGACTCTTGGATCATATAGAGAATAAGTCTGGCTTAACATCGCGTTTGATGGCCCTTAAATTGTTTATAGTTGACGAGGCTGATCTATTGCTAGACTTAGGATTCAAGAGAGATGTTGAAAAGATCATAGATTGTTTGCCTCGTCAGAGACAATCTCTTCTCTTTTCCGCAACCATTCCAAAAGAGGTACAATTGCTATATAGCTAACTTCATATATTCTTACCTTTATCACAAAATCCAATGCGGACACAAACGTCTAAAATGTAGTTTAAATTCAGGTCCGTCGAGTCTCACAACTTGTTTTAAAAAGGGATCACTCTTATATCGACACAATCGGGCTCGGTTGTGTAGAAACTCATGATAAGGTAAACTCTTAAGCTCTTCTTTTTGTCTCAAAAGCTTGTTACTTGAATCTGGTTATTGAGTTTCTTGCTTTGGTTTTAGGTGAAGCAATCTTGTATTGTTGCTCCACACGAATCTCATTTCCACTTAGTACCTCATCTTCTAAAAGAGCACATCAACAACATGCCTGATTATAAGGTAAAACAAATCCTCTTAAACGATTGCTTTTTTATTTCTGACCTTGGTAATTCTCGTAACTATCCAATTTCATTCTCGCAGATAATAGTCTTTTGCTCAACCGGTATGGTAACATCACTGATGTACACTCTGCTCCGGGAAATGAAATTGAACGTTAGGGAGATTCATGCTAGAAAACCTCAGCTCCATAGAACATGTGTATCAGACGAGTTTAAGGAATCAAATAGACTTATTCTTGTCACATCTGATGTATCTGCTCGTGGAATGAACTATCCAGATGTTACTTTGGTTATTCAGGTAAAGCACAAAGCGAACATTTTCATGCCAAAAACAACCTTTTATGGATATTTAACGGATTAGACTTTGTTTTACGTTTTTGGTAGGTCGGTATACCGAGTGACAGAGAACAGTATATACATCGGTTAGGTAGAACTGGAAGAGAAGGAAAAGGAGGAAAAGGATTGCTTTTGATTGCGCCATGGGAGAGATATTTTCTTGATGAGCTTAAAGATTTACCTCTTGAACCAATTCCAGCTCCAGATCTTGATTCAAGAGTTAAACATCAGGTTTGTTCGCCTTCACTGTCAATACCCATAAGTGGAGTTTTATTGCCAAGTGACATTGAGGACAATTGATTTTTGACTAGGTTGATCAGTCAATGGCTAAGATTGATACAAGTATCAAAGAAGCAGCGTACCATGCATGGCTCGGTTATTACAACTCGGTTCGAGAAACCGGTAGAGACAAAACCACGTTGGCCGAGTTAGCTAATCGGTTTTGCCATTCTATCGGTTTAGAGAAACCTCCTGCTTTGTTTCGAAGAACCGCGGTTAAGATGGGTCTTAAAGGTATTTCTGGTATTCCAATCCGAAAATAAACCGACCCGTGTTGGTACTTAGATGTATCGTCGTAGAGAGAAATGTATGAACACATTTATGGTCAACATCATGTTTAAATAATTTTGTTATTTATTTATGAATATATGATTAACTTTTTAATTACAGGAATTTTTGTTTGTCTAAGTCATGTGTTTATATATTTGACTACTAGTGTAATGGCCTAAATGGAGATTAATTAATTTTAATATGTACATTACAGATCCTTAAGCAAAGTAAAAACATACATTATTATTTTACTAAAAAATGTCCCACGCGATGCGTGGGTGAAATGAATGTAGTGAAATATATGTCTATAACTAATATATATTAATATAATATTAAATTATTTTATAATACATTCAATAAAAAATATCATAAATATTGTGATTATGATATTTTAGTTTTAAACTAAACATTTAGTTTATGAATTATAATCTAAAATTATTTTGGGTATTTATAAATTAAAAATTATCGAATTGATAAAAAAGAAAAATTATAATATTGATATACAAGATAAGGCTTGTGTTTTATTTAAATATATGAAAATGATATGCAATATTTCTCTGAGTAAAAGATTTCATAATTGTATATATCATATTGATTATAGTATGTGTGTAAATTGTAAAGTAGTTAATAGATTTGAAGTTGTGAGATTGATAAAGTCTTGATTAATGAGTAAGATTAACATATATGAATATTGATATATTAATACTAATTGATATCAAAAGAACTCTAAATACTTTATACAATTAATATTCAAATTATTTACTATTTCTTCCATCTTTGAACAATATGGTAAGAAAGTATTGAATTTATTTACTAATGATTTAGTATATACACATGATTTTTAATAAAGAAGTTTAAAGTTATTTCGATGCACATAATCAAATACTAAAGAAAATATACCATAATTCGGTTGAAAACTAAATAAATTTAAAATTTAAAGTACAATGTAACAAGTAACAACATACAAAATAATTTAAATTAACAGAATTTCCAACCAATGATATCAATGAAGATTATATATTTTGTATAGTTTCATCATTCATCAAATCTCGTAGGTTCTATATTATCTTTATCCATATAATTTCTTTCTATATTACCTCATTTGCCAACAAATGCATCAAAAGACGAATTATTATGGTCACTATAAAACTTAAGAAACGAAATATAGTGTAAATTTAGCATGATAAGTAAAAGATAAACATTTTTTCAATTACTATGATTAGTAAAATGAGTTATAAGTTACTGTTTAGTACATAAGATCAATAATAAATCAAATGTGTACAAATAGGAATAATGTTTGTATATTAAACTATAAACTAAATGAAAAAGACGAAAATTAAAGAAGTAGCCACACATTAAAATAAAAAGTGAATATATTAAGCAGGACCAACCAGGAAATATCATATGGATCCAATAAACATTTGGCTATATATGTTTATCATTTGGTTGAGTATTCACTGTTGTTATTTTTTATTGAAAAACTAAAATAATTTTAAAGATTAATATGAATTTTAGCAACTTGACATAAATATTTTATAAACAACATTGGTCAACTTCAAAACATGAAGAATAAGTAGAAAATGAAGGTAAAAGATACTTTGTTTCTCATGTAAAAGTGAGAAGGTACCTTAGACATTTTTCATCAAGTCATTTCTAAAGGAAATCCACCTTTCACATATTCACTTATGCATATACTATAAAATTTGACTCAACCTCTTTATTTCAAAACCTACGAAAGTCACATAAAATTTTGTTTTAAAAGATATTTAGTTAAATATATATATATGTATATCTATTAATACTATGTTTATATCCTCTATTAAAATATCGGACATGTTCATAAAATAGCAGGTTGAAGACACCGGGTTCCCATGTCGAACCACTAAAGTGAAGCTAAAGGCCTTTGACACCATTTACTAAACTTTGAAACTTTGACTATGGTTGTGACTCGAATAGTTAAAACACAAATAGCTGGAGAAAAAATTAGAACCATGACATTAATAATTAAATAAAATATACATCACAGATGAGCATCGAGCAACACATATATGATTTGCCTACCTATTACCTTCATCATGAACAGAAATTCTCAGTGTTATGATTGCTAAGAAATTCTTAGTGTAATAATTTTTAAGCCACTTTGTATCTACCATTGCACCTATAAAAGGATGTATTAGCAAAAACACATGAAACATGTTATAAAGAAGAGAAGTAACAAAGTGTGAATCAGTATCACCACATTTGGACGATACAAAGGCTACACAAAACAAATATTAAAATTATAAAAATGAGAGAGAGAAACTTGATTTTAGTTCATTTCTTTGGTGAAAATGAGAAAAATAAAAAACAACGTAATACAGAGGACGAAGCAAACCTTTGAGATTACCATTCTTTGGTTTCTCCAATCCGTCATACTCATATGGTTCGTGCTTTGTCTTTTTTCTCTTTACTTTCAATTTAATTATGTGGAAAGACTTAGCAGACCACAAAATATTATTTCGATGTTTAAACAATCCCGAAAGCAACACAGAATGATAGAAGCTTAAAAGGAGTTGTAAGAAACAAACCTTGAGGAACCGATGTTGATGTTGTTCCTATGAAGAATCCTACCAGGAGCAGCGTCACCACCATCGATCAACTCTCTAAGCCCCCAATTCTCCACCTCCTCTTCTTTCACCTCCTTCTTCTTCTCCTTCTCCACATGGCGGTTATTAGGAAGCTTCACAGGCAACCTCTCGAAGTAATTCCCGATACTTACGATGCACGTTTTTATTATCTTCGTATAGAAAGAATGAAGCTTCAACCTCAAGCGTTAACAAATGCAGAGAAACCAACTTAAAAAAACTGACATGCAGCGTCTTGCATTTTATAAGGTGAAGGGTTACACTAAAAATGATATTAAATATGATACTAAATATTGAAATCATGGTCATAATTACATAAATGATAATTATGTTAGTGTAACGTAACTGCTTTGATTTATGAAATAGAACAGTTTAGTTGACTAAAAAAAAACTGCAATAGTTAGACATAATAAATAAAAGAAAACGTGATATCACATGTGTCATTAAATAGAATATTAACCTCACAACATAACTGAAACGTATTATGAGTAATTCATTGTTTTTTCATTCAATTTATTATATTGTAAATGATATTTATGACTTAAGTTATATGGTCAGTTATGTTGTTTATTTTGTTTCAAGATATACAAATAAACATTTAATCGATTATGTGAGAGGCCAAATTTTTTTAAAACAAATAAACAAATGATCATATTGTCAACAAACACATACCATAATCTACTCATACATTTTTTATATTTTGAAGGTATTTTAGAGAAAAAATATTTGATCTTTGGTTTGAAAGATAAAACTAATATTTTATTCTTTAATTCTTGAGAAATTATTTTGTTGAGAAATTCTAATGTTATGGTAATTATATAAAAATATTGATAAATGATACATTTTTAGGAAAAATAATTATTTAGAAAAAGTGCATTGCAAAAATGTTATAAGTTTTTTTTATTATAAAACTGTAATTAAACTCAAAGAATATGGAAAATAGTATATTTAAATTTGAGAAGAATGACACATGTCGAATGAATAGAGCGCCACTTGTCAGATTTTTAGAGTACAAGTTTGAGGAAACCTTACTTTATTATATAAGATGTGGTTGGTTACTTGTTCGTCTTCTTAATTTTTCGACAATTATTAAATAAAATTGTGATGATTGAAAACTATATAATAAAGACAAAGTTTGGGAAAATAAAAATAAAATAAAGAAACGTGCGAGAGAGTATAGGTGGTAGATTCTTTTTCAGCTTGTTCACTTGAGTATAATTTTTTTTTTTTTTTTTTTAAACATTATATTAATAACCGATACAAATGTTATGACCAAAAGTCACACAGTTTTTTGAAGTTCGATACACAATATAGCCATAAACTAAAAGTATATAGAGATAATAACCAAATCATCCGCTTCGGTTACCTTTTGAGATCAGCATAATAGAACCATTAGCCACGTCCACGGTTCCCGCGTCCACGATTTCCGCGTCCACGGTTCCCACGCCCACGGTTACCTCTTCCTCGAGTAGTAGAGGACGCTTGTATCTCCTTAGCTTTGATGGATGGTTTAACCGGTCTTTCGCGCAAAAGACGTTGTCCCGATGGAGTCATAAGTTCCATCAATTCTGACCCACTGTCTGACTCAACTGAAACAATTGAGTCTTCATCTGAATGAAGAGAATCCCCATCAGGAAACGAAGAACCCAAACGACTAAACCTGTTTTCAGCAAGGACGTGTCTTATCTCACAATCACTAAGAATCACTTATCTTTTGTGCCTGCAATTTCGAAAACTGATTCAACTGTGCAAGTTTCCTCAGTTACAACTTCAGCTTCAACATTTGTAAAGCAATCTGCCACTCCGGTGCAATTATCTTTGTCTTGGATTGGGCCAGCCTCAATATCGAAGACTGTATCGATTTGGATTTTTGAATTTGTGATTGGGACATCAATTGGGGTCTTGGTTTGAAGAGTGATTGGCGATACCAAATTTGTGGCACTAGCTAAACTTACAATAGCAGGAGTAATGATCTCCTCTGAGACAATTTCTGTTACTTTTTCATGTGCCAAATGGGGTTTCATACAGCGCGAAGCCTTATGCCCTAACTGCCCACATTTTCCACATTTAGCTGGGATCCAAGCATATTCAACATTAACCATCGATATATTGCCTTTCTTATCAACTGCGGCAATTCTTGGAGGGAAAGCTTTACTCAACTCCACTTCCACCAAGATATTGGCCTCACTCATTAGGGAAGGGTCAAGTCTAGGTTTGTAAGTAGCCATAGGAGCACCTAAACCTGATGCAATATGGCTAATTCCTAGAATTGAGTACAACCGGTTTGGGATATTTTTAAGGGTTACCCAGACAGGAATAGTTGATATTTCAGGAAGATCAAATGTATTTACAGTAGACCAAGGCGCTACAAACATAAGACAATCATCAACATGCCATAAGCCCCTCTGAAGAACCCAAGCTCTAGTTGAAGCATCCGGGATGTGAAAAAGAAATTTTGAGTCACCTAATTTACGGATAAAGATTCTACACTTTCTACCCTAAATTTTATTGACTACCGCATGAATCAAACCACCAGGAGGAGGACTACATCTATAGAACTGGCCTAAGACATATTCCTTTTGATTCACAAGACCTTGCAAGAAGACATGACTTGGAATCGTTACCTTTGGAATTCCGTCTTCCATGAATTCCGGCGAGGTTGCCCTGTAAAGGTTCCTTGCCGCTGGATCCATTTTTGCAGCCCAAGGGAAGCGAACATTATCCTCCTTAGTTGCTTCAAGATGAGATTGCATCGGTGCTCCACCAACATCTTGCTGAGAGGCCGATGACGAGGTGAACTTTAGCTGCTTTGCCCAAGCTCCTTTTAGAGTAGGGGAGACCAACTTAACAGGAGCAATGGACTCGTCGAGAGTAGCAGACACTATTGGCGGTAAGGCGGAAGTAGGAAGAGACACTTCTGCGGAATAAGACAATGATGTAATACTCACCACATGTTCAATTGGAGGCTCGACAGTGACCGGTAAGGTCGAATCCAGCGAAGAAGTAAGATTCACGAAGTCAAGAGAAGAAGAGCGAGCAATTTTCGGCGAGGATCCAATTGTCAAGGGTTTCACCAGTGAGATAGAAGGAGAAATCACTGATGGAGATGGAGAAGGAGAATTCTGCGTCCACCGAGTCAAGTACGGCCACCGTGGATCAGGGACAGAGTTGACATTGGAAGAGTCTACGACTACAACTTTTGGTCCGACCTCTGTTTCCTTGCCTTGAGTTAAAACTGAGACAGAATTCATCGAGTGGGATACTGTGAAGTGATTGATTGGAAGTAGGCTCTCCTAAGAAGATTAAGAGAACAGCCGGCGACGTTTCGCGGTGGTTACAGCGAAACAAATTCTCAGGGGCTCCTCTTGGCTGAAGAGTAATCTCATACACTCTTCTTATAAAGAGCATTTTCTTTTTGATTTTTTTTATTCCGTAAAAAAAGTACTCAGAAAACTCTAAGAATATTGATTAATTACGACCCAACCAGGGCAAATGGATCCCAAAATAACCCAAGCCCACTGTTTTCGGGTCAAATCCAAATAAAACCCTAATTCCCCTAATTCTCTGCGGTAAAATTGATTCACATCTCTTTCTTCCCTTTGTTTTTTGTTTCTTCCTTATTTCGTCACTGTAAAATCCAGTAAAACCATTCTCAATTCTCGTCAACGACAACAACATAACTGTTCGTCGATCTTGGTATATCTACAGGAAACTAAGAAATTGGGTAAGTGATTTAATTGTTCGTCGATCCGAGTTGTTTCAGATTGGGTTTTGTGATCGAAATTGACTGAAGAGTAGTGCTAATCCAAATGATTTTGTTTGTAGGTTTGGAGAAATGGCTCGAACAAAGAACGCAGGTGTGCCATTGGCGGCAGAGGTGGTGGTTTCACAGACAATCAATGACGAAGTAGTAGTAGCATCGGCTGCTGAAGTGGATTCTGAAGCAACAAATTCAATCTCTAACGACGATAGAGCGATATGAACTTTAAACTTTACACAATTAGAAAAGTATAACTATGTATGAATTTGGTATATCTTTATTGATATTGTAACTTAGAAATTTTAAAAAGTATATCTATGTATAAATTAGTATATCTATGCATAAATTGAGTATATCTTTTTTGAATCTAACTTAGAAATTTTCGAAACTAAAAACTAGGTATATCTATGTTCGAATATAACTTAGATATTTTAGAGACTAAACAATATATCTATGTATAAATTAGGTATATCTATGTGGAGAAGTTCTTCCTTAGGGCTGTGGCTGATCTAGAGTTGTGCAAAACATTTCCGTGGGGAAGATTTGCATTTGAAGAAAATGTGAAAGACATTTTCTATTTGATTAAAAAGTGCAATGAAGTAGTTGGTCCACAAAAGGTGTTTCCTAGTTTTGTTATGCCTTTGGAGGTATGTCATTTCATTATGCATTTATGAATTGTTTTAAGAAAGTTAAACTATGTACAACTGTGTTTTTATTAGCAGTTATACTTATTAGTTTTTGAAAAATCTTGTATTGTTTTTTTGTAGTATCTAGCATTTGAGGCGATCCCAGTTCTCAGAAAAATTTTTTGCGAAGATATTGAATCCGCAGATCCTCAGTGTTCCCGGATGTGTAAAAGGAAGTTTAAATCAAGCACTATGAAAGGGTTTCCAATGTCAGATGTGTATGACAAACTCGGTACAACTAAGGTTAGTGATGACATATTTATTAGTTATCTATCATGTATCATGTATGTTCTATCATGTATGTTATATTAATTGGACTTTCTTATCATGCACAGGACATTCAGAGTATCTTGGCTTCAACTCCTGATGAGAAGCTACTTTTGGAAAGAATTATGGATAAGGAGTGTGAAGTGAATGATGTCGATGATCTAATAGCTGATGGCTGGAAGAAACGTCTAGTTGATGAGGAGAGGACTATATGTTTTGAACCGTTGCTCAATGAAGATGTAGCACACCGGAGCTTTGTGGCTAATAAAGCTTTGTCTACTGTGGTAAAAGCTCCAAGAAAAGCCGCAGTGGAAAAGAAAGGCAAGGGAAAGGCTGCTGCAGCTCTGACTAGTCCAAGTGATGGAGGTTTGACTGAGGTTGTAAACGAAATGAAGAATTTGATGGAGAATGGATTTAAGTCCATGAACAAGAGGATGAAAGATTTCTGTAAGAAGTATGAAGAGCAAGATAAAAGACTGAAACTGATGGAAGCTGCAATCAAATCAATTCAATCAGGCATTGGAACTGAAGACGCTTGTGGTTCAAAGGAGATTGATGATAAAGAAAACGAGCTGGAGGAAGGGAGTGATGCAGAGACTGAGATAGACAAAGAGGTGGCTCAAGGTGATAAGGAGAGAGAGGTTGGAGAAACAGAGACTCAGATAGACAAAGAGGTGGCTCAAGGTGACAGTGATAAGGAGGTGGCTGAAAGTGAGAAGGACAAAGTGGTGGCTGAAAGTGAGAAGGAGAAAGAGGTGGCTGAAAGTGAGATTGGGGTGGCTGAAAGTGAGAAGGACAAAGAGGTGCCTCAAGATGATGAGATGGATGGAGGTAAGGTGGCTGAGAGTGATGGAGAGATGGATGGAGAGAAGGACAAAGAGGTGCCTCAAGATGATGAGATGGATGGAGAGAAGGAGAAAGAGGTGGCTGAGCCAAGTGAGATTGGGGTGCCTGAAAGTGAGAAGGACATAGAGGTGGCTGATAGTGAGAAGGAGAAAGAGGTGCCTCAAGATGCTAAGGTGGCTGAGCCAAGTAAGAAGCGAGGAAAGGCACATGAGGATGGAGATGATCCTAGTAAAGAGGGAGTTAAAAAGCCAAAGGTGGTCAAGAAGCTGGCAGAGTCGAGGACGGATGCTAAGCCGGTGTATCGGAGTCCTATACAGACGAGGTATAGGAGAAAAAAGACAAAGAAGAATGTTTAAACAATTTCAACTCTTGGTTTGTTTTAGGTATAACTATGTTGGTTGTTGAAAACTTGGTACATCTGTTCGAACTTGGTACTTGGTTTTTTTTTGTATTTCTCCTTATGCTTTTGAAAACTTGGTACAACTGTTGAACATGTTTATTAGGTTTTTTGTATATCTACTTATGTTAATTTCAAAACTTGGTATAACTGTTTCATGTTCTATTGTTTTATTTTGGTATATCTACTTATGTTTTGTAACACTAGGTACAATTATATGTTTTTGTAATTGATAAGAAAAGAATTTTAACCGAAATAACTAGATTCAACACAACACTTAGTCTTTATAATTAGATTCATATCCATTCATCACATCAAATTCAACACTTAGTCTACATAATTAAACATTACAACACTTACTCTTCATAATTAAACATTACACCACTTAGGCTTGAATTTCTACGACAAACACAACATGCCAACAATTGCCATGGTGATTAAACATCCACAACTAAAAACGATTACCATCTTAAAGTCCTTTCCCTTTTTATTCTTTTCCATCGGATCTCTTTCCATCGTCTCTTGGTTCCTATCCATCTTCATCACTCTTACTTCTTCTTCAAGAACACCGAGTTGAAATTCCAACTGTTCAATCTCATGTGTAAAAGCCTCATCTACCCACTTAAACACATGTTTGTCGTTCATAAGCTACATTTTGAAATTGTAAAAATTGTTAAATCACATGTATATCTATGTATAAACTAGGTATATCTATTAAGTATATCTATTAGGTATATCTATTAGGTATATCTATTAGGTATATCTAGGTATTTTAGTGACTAGTCAAATTATTGTCAATTGCATATCAGAAATCTATAAGTGAAACGTGAATTGAATGAAAAATGTACCCAGAAAAACGAAATGCTTACCCAAATAATTCCACTCTTCTCTCTTTTTCTCTCTTCTTCTCTCTTTTGAGTCTTCTCTCTTCTTGTCTCTTCTTGTTCTTTTTCGATTTGATTTTTTTTATTTTTGTTAATAATAGGGTTTTAAAAAATTTCTGCGAATTGGGGAGAAATGAAAATTTAGGGGAAATTTTTTTTGATAGTGTGAAAAGGAATTGATAGTGTGAACACGTTTCTTATAGTGTGAAAAGGAATTAACCGTGGGACCCGCGTACGAGTTGTTCAGAACAAAAAGGGTGGGGCCCGCAAACTCTTTGCACACAGATATTCCAAGTTAACAGGTTGTACCTGTGTATACACAGTTTGACTTTTGTTTTTTTTTAATTTTATTAGATATACTTAAATTTAAACTAAACAAATTTAGTTATACCAAAGAGTTTTGATGTTTTATAAACATTCCACATATTTTCATAGAAAAATATGTCTGGAAAATTCAATTTTGTGTGTTTTGCAATGTTATGATGATAATAATTATAAAGTTATCTTCAAAGTGTAAGGTGTTTCTTTTATAACAAATGAATTTGGTAAAGACTAATTTTTTGAAGTTTCATTAAATTATGATTTTATTAATCAGTAGTTTTAATACTCAACATAAGGGGTATAATTTGTATAAGAAATAACATCACATCAAATTTTGTTGCAAATTAAGCACATTTGTTACATTTGTCATAGTTGTACCTTTACAAAACCAAGTTTAATCAAGTTTTACAAAACCAGAGAGTACAAAAAACACGAATTTAGAGACATACATAGTTCTACCATTTAAAAATTAAGTTTAACCGAGTTTTACAAAATCTGAAAGTACTTAAAAACATGAATTTAGAGCCATACATAGTTCTACCATTAGAGAATAAAGTTTAACCGAGTTTTACAAAATCTGAAAGTACTTAAAAACACGAGTACATTAATTTCCGCTTGTGTATTGGCCACTCCCCTGGCTATCACTTTCGTGGTTTGGAAAAAACCGGCTCTCCAAGTTTCTGCCTTTCACACGCTTATTCTTGGGGCGACGTTCTCCAACTGATGGGTGTCTAGTTTCTTGAACTCTTCCCTTTTTAACAACATGAACCGGAGGATATGCAAACAATTCTTGGATTTCACTTGGAATAACTCACTCTGACTTATGCGGCACCGGATAAATGGTTCTATCATATGCCATAACCCACATCTCAACAAAGTAATATTTCGTGATCAAATCATATAAACTTTTTACCTCCTCGGATCTATCTTTTTGTTTCAAGTGAGTCATCACAGCCGCTATTGCATGTACACAAGGATACTTGTCCAAGTCAAAACAACGACAATAACAACTTTTATTCCTCAAGTCCACCACATAGGTCTTACCATCGCGACCAATGACACTATATTGAAGCTCGTAGGTGTTAAGCTCTATCACCGGTAATTTTTGTGCAACTTCACATGTTTCATGTATCTCGTTCTCCACAAAAGGAACAAGTCTCTGTAAATTTGATCCACTTAAGGCTTCCTTCCTATTATTGCAGCTCCATTCCGCAAATTTCTCAATAATTGCATCTATCATAGGTAATAGCGCATACCCTCTCACATCCCTAAACACACCATTCATAGACTCAACCGCATTACTAGTGTCAACGTTGTACTTTACTCCCGGAAAATGACATCTTGCCCATTTTTCCAAATCAGTGTTTTCATCAAGATAGGTGGCTACCTTAGGGTGCCTTCTTCTAAACTCTGCATATTCATGTTCAAACTCAGACACTGAACAAATGGATGCAATTCTTCTGAACGTTTTAGCAACCTCTTCTTTGTTGTATGCAACTTTTGTTCTCACATTCTGTGACAAATGATACACACAGTTTCCATGGTGAGAGCTCGGGTACACCTCAGCCACTGCCTTGATGAGGCTAGAATTTCTGTCAGACATAAAGACCAATTCCGAACTGTCTGGTATAACGGTTTTGAGAGTTTTGAAAAACCACTTCCAAGTAACATCTTTTTCGCCATCAGCTACAGCAAACGCTAAGGGATAATGGTGATGATTCGGGTCTTGAGCTGTGGCAAAAATGAGCACACCTTTATATACAGTCTTCAAAAATGTTGCATCCACAATAATCACTTTTCTCATGACTTTAAATCCTTCAATGGCAGCCCCCAAAGCAACAAATAGGTACTTGAACCTTTTTTCTTCATCTAATACCACACTAGTCTTTGTGCCAGGATTCACCTTCTCTAACAAATACAAATATTGATAAACCATCTTGAAACTCTCCTCGGGACTAGCACGAATATCACTAACAGCTAGTTTTTCCCCCTATTAGTAGTCGAGTATGACACAGTTGTACCATGTTTTCCCTGAACAAGGCCAATGAGACTTTTAGGAGTTGGGGTCTTGTACTGACATAGATAATCTTCATGCAATACGGTTGCAACTAAACGTGGTGTACCTCTCCTTTTAATGCCTGTAGCAGTATTCGACCGTACACATGTATGGAACTTCCTATATGTTCGAACCGAGAAGCGAGTTGAATCCTTAGCCTTCGAAAATCGTATGTACCATTCACACCCTTATTCAGTACATTTTACCACATACCGCTTCTTATCCGAGTTGAGTAATGTTAGTCCAAAACAATTCCTATTTGCTCCTGTCACATCATCGCTATTTTCAAATTCTTGACCTATTACTAGATCAAGACCATCATCCCATGGACCTTTAAACTTAGAATGTTGGCTGTCACGTGGTTCCTCAACATATTCACGAATACCATCATTCTCCTTACTCATACTCATATTACTATTAACACCATTATCCACATCTCTAAGAGGCTCCAAAGGCTCTAAAGGCTCCAATGTCTCCAAAGGCTCAGCAAGTTGTTCTCCTAGTGGCTCATTTGCGTAAAGAGTTATAGCACCAACATTAGCTCTATCATCATTATCTAGACCACCAAAATTCTCCTCTTCATAATTCACACCAACTGAACTCTTTCCTTGTACTCTCGATTGTTGCTCGTTTAAGTGAACCATTTCCAACTCATACCGACTTCTACATGCAAGACACTTCTACTTTTCTCTTCATCCAACGTGGTTAGATAAACATATACATCATCATCATCCCTAATATATAAATATGTAGGAGGTTTAACACCCGTTGGAATGTAACTCAATTTCAGCTTCATCGTAGATTCATCTATCTTCATCTTTGTGCATATCTTATCCACTAACCACGAATAAGTAATCTCCTCCATTGATGATGTACTAAACAATATACCATACAAACCAACTACTTTTGGATTCCATTGATATGCTTCTCCGGTTTTTGAATAATATCCATCATAATCAAAATATATGATTATGCCCATTCTCCTGGAAAAAACAAAAAAAAAAACGTTAGCTATACTTAGATTTTCCTGTTCTAGCAAATTGTACTTCGAACAGCTATACCACGCTCATTTTCAGAAATACCAGTTATACTATTCAAACAGCTATACCACGCTCATTTTCAAAAATACCAGTTATACCATCAATTGTACTTCAAACAGCTATACCACGCTCATTTTCAGAAATACCAGTTATACCATAAATAGTGCTTCACACAGCTATACCACGTTTCTTAGGTATACCTAGATTATTCTGTTCTAGCAAAGATTTTCGGACCAAAAACAGTAATCTGCCTAAGATTAACTACGAATCAACCATTACAGTACATTAAAATGTCTTAATAGACCCAGAATATATAGTTACCAACATAATGTCTATTAATTTCCAACACCAATTTCAAATCTATCAAATCTAATTGAAATCTACAAAATTCAAACACAACAACACAAGACTTGATGAATTAAGATGTCCAAAGTATAAGAAGAGAATGGTAAAGCTCACTTTGAATAACAGAAGCAAAACGACGGCGAGCTTGGTCGATTGGATTCAACGTCGGAACAAATCGACGTTGGCTGCAGTCGTCGATTTAGATTTTGTGAGGGAGGAAAAGAAATTCTGGGAATTTCTGATAGGTTAATATTTTGTGACTTAAATGGTATTGGTTTGGAGACTTAACCGTAATTGGTTGGTTTGGTTCGTCCGTCCGGTTCCGGTTTGTTGGGTAAGGTAAACAAGGATTTTTAAAATTCATTAAGAGTATAGGAGTATGTTAGATTATATGAGAGTATTTAGACTCATTGTCCGATTACAGCTCCGTCGATTTGATCTGACCGGAGACTAGGTTTCGCCTTCGAGAGAAAAATTAGGGGCGCGTGTCACACACCATCTTTCCTTTCCTCTCAACCGCTATCAAAGATTGTTCATCCTGAAATTTGGTTGCTTTCTTTCATCAACTTCTACTTTTTTTCTAATCAGCTTCTCGTCCCTCAATTCCACCGCCAACAAATCATGTTCTTTTTTCTCTGAGATAGTTGAATCATCCACAAAAATTTTTGATTTCTTATCTGATGGTTGAGATTTAATATCAGAGTGGACATCCATCTACTGCAAGTTTACCCACAAGGCAGCATCAATGAGTGAATCCCTGAGTAAAAAAAGAAGTGGAGGAAATATTAGATCTTATAAACTAATGGTTAAAACGAAAAAAATTTACACATGAACTAAAGGTGAAAATAAAAATTACCCAAGATCTTTAAACTAAAGATTAAGCAATAGTGATGATCTCCTTAACTTTATTCATCCCAATAATCTCTCTAATAATGTTTGCAAATTAGAAAATTGCATAAAGAAATAACTTACGTTTGTATGAAGAGAAAAAACTGTAGATAATAAAACAAATTATAGCGATGGCAATGATGTACCCATAATATAATATCCTACTTATATCCTACTTTTTACTTTTTACAGAAGCTTGTATCCTACTTCCATGTTTCAAAATAAAATATAAACCTTGTCACTAACAGTTTACATCAAGTTATAAAAGAATACATTGATTTAGACAAATATCCAACTAAAATAAATTTAATTTTATAAAAAAAAAATATCCACAAGGTCGTTTGATTCTAATCCAAGTCATTAGTAGTTTGTAACGACAAAGATTAGAGAGGCGGTTTAGTATATGTGATACTGTTTCATATAGCAAAGAATGAAATATAGAAAATTGAATCAAACGAACCTCTTATAATACCAAACTTTTAAAGATGGTAAAAACTATAATGTAGTAGTACAACAGAGCTCTACATTAGTCTCATCAGTTCTCCTTCTTCTAAGTAAATCTTAACATAGGTCATGATATAATAGATTTTAGTGAGACTTCAGAAAAATCCACAAAATTGTACCTTATCATCTACAAGAAGAAGATTCAATCCAGTGAATTCCCTTATTTATCCTTTTTTTTGGGGAAATCCCCAAGGGCACTTCTCACCTTGATTTTACATCGATCCGTTACTAGAGTTATATGAGAGATAGGTGTAATATTGAGTGACATAATTGGAGATTGGAATTTGGAATATGAACCTACAAATAAAAAGTTGTTTTTCTTAAATAGAGAATTTTATGAGCAAGAGAAACTTATGCAATTTAGATAAAGAGAATAGACCATGTGGGTCAGTGGTTCGACACAGTAATGCCAAAAATTCTAAAATTTGCAGACACAATAACACCAAATCACTTGAGAGAAGATAATTGAACTATAACAAAATTTACAAACCTGAGAAATACGAAAACTTGTAAACTTGCATAGAGGAAGAGACATGTGATTGATGATAAACCCTAAAAGTAAATCATGTAACGACATCATACCCAAAGATGAATTGGAAACCAAGAAGGGATGATGTGAAGAGGAGAGGCAAAGTGAGAGGCGGAAGAAATAATGATACTTTTTCGGTTAAAGAGGAAAGGTTGAAAGTGAGGAATAATAGGGCCATGTTTGTTTGATCATTTGGAATTTGCATCCAAATGATCCATCCAAATAATCCATTTGAATGATTCATCTTAATGATGTTTGTTTGATTATTTGCTAATCATTTGCTAATTCATCTAAATGCATCATTTAGATGGAGCTTTGTTTGTTTGATCATTTGGATGAATCATTCACATTCTCATATATATATATATAAATGACTAAAATGCCCTCTGTTAAGTAACAAATAATCAATAACTACATTCTCATATTAATCAAAGTAGAGTCTTAAAAAGAGAGTTCTCTGTTTCCATTTTCTAAGTTCCTTCTTATGCTCTGCAGTTCCCTAGCCCGAATGAGTACTCAGCATTTATGGGAGACTTCTCAACCTGCACTGGTGTTGCAACATTCACAATGATGCTACAATATCAATGATAAAGAAGACACAGTAAATGGTTGTTAGTAATAGTGATTGATACTATTCAATGATTGATAGTACTAGTGATTGATACTATTCAATGAAAGGAATACATGAGCTTAGAGACCAACCAATATTCACGTGATTCAATTATGACAAACCAAGCTTAAAGACGTGATCATGTGAATACTATTATGAATCTTTTACAGAAATACGTAGATACAGAGACATGTGCAAGTGAATGAAAGATGCAGAACTTGTAGGCCCTTTGATGTTCTGAGAGAAGTTAAAACCATAAACCTGAAGAAATTTCAATAGATTCCTAGTGTCTAGATTAGTTAGGGAATAAATTGGGAATGAGAAATAAAACTAAAACGTCTAAAACCTTTTTTTCATGAGAATCTAACAAAAGATGTTGCAGAGACTTGCCCTTATATTCATCAAGTTATTCATTGTGTTGTACTATATTAGTAGTGTTACTTTGTTGTTCTTATGGTTGCAAAGAACATTGTAAACGCATCTAGATTGAAATATAGTTTCAACTTACGTAGAAAGCGATGGATCAAACGCCCTAACTATCTGAACATGGCACATTACATGATCTAAAGATTTAGGATCTAAAGAAGTGGGAAGGCTTTGCTAAAACGAAGGTTAGCTAACTCCTTAAACTCAGACACTTAACAGTTACATATCATCACCATTTAAACTCCTTAAAACAATAACACACTAAACAGTTCATAAACGAAAGAATATATGATGATGACTTACAGTGATTCTTCCTTCAATCCTTAAATCCTTCAAGCCAAATCTGGATCCTAGCTTTCTGCATCAAAAACACAAAAGACCTATAATCAAAGTAACACAACTAAAGCTAATAGAATAATCAAAGCAACACAAAGCAACATAAGCAAAACAAAGCCGTAAACTAAAGCTAATCGAAGAATCAAAAGCAACACAAAGCAAGCAAAGCAACACAAAGCAACATAAGCAAAACAAAGCTGTAAACTGTAGCTAATCGAAGAATCAAAAGCAACACAAAGCAAGCAAAGCAACACAAAGCAACATAAGCAAAACAAAGCTGTAAACTGTAGCTAATCGAAGAATCAAAAGCAACACAAAGCAAGCAAAGCAACACAAAGCAATATAAGGAACACAAAGCCGTAAATTAAAGCTAATCGAAGAATCAAAAGCAACACAAAGCAAAGTAAAGCAAACCCACAATTAAACAATGTAACCATGGTGGATCAAACCCAAGAACAAGTGATTGCAACAAGGATACAAAGAGAAACAACGAGAGAGACATGGAGCGAGAGAGAGGAAGCGAGAGAGAGAGAGAGAGCAACACAAACCTGTACACTCCGGCGATGGATCCGACGAGTCTCCGGTGAAGAGAATCGGCGACTGCGAGAAAAATCGGCGACAGCGAGATCACCTCGACGACAATGAATCTCGTTTTCAGTGTTTTGTGTCGTGGAGATGCTGAAGAAGAGATGTGGTTTTTGGGTGTAATTCCATATATTAGATTAGGGTATTATTGACATTTTACAATTTTGGCTGAACCAGCTTCAGCAGAATGAATAGATTAAACTCAGTCAGATTTTCTTTATTTTTTGGATGAGTTTAGACAAAAGCATTCAACTGAATCATTCAAATGATCTCTTAAAACATGAAAAACAAACATCAGTTTTAATCTTCATTGAGATGGTTTATCCAAATGGACAAACAAACAGGACCTAGATCGTGTGACTGAATGACCCGAGCAGTTGATTTTCTTTTTGTCGTAATTTTTTTTTTAACTTAACAATAAGGTGTTTCCTTTTACAAATTACAATCTTGAAAGGTGAAAAATGAGACTTATGACAGGTGTGAGAAGGAGAATGTGACAGTTTTTTGTAAATTAACTTGTGATTTATTAGATAAAGATTTGAGGAAGTTTGTTTATTATTTATAATCGTTACAATTGAAATCATATATATAAAAGCTTACCAACTCTCTCCATAAAATTGACACATAATCAATTTAGCATTTGCCATGTGTCTATTAAATAATTAATGTAAGCTCTTCAACTTCTAATTTATAGGTTAATGATATATTTATTATTTAATTTTATATACGATTTTATTTTATATATTTAATTGTACTATTACTAAATTTGTTTTAATATTACATGACCTTTCAATAAAACCATTTTAATAGTTTTCTCATACTCGGAGATTTTTTTTAATCCAAACTCAAAGTATGAGTGTATAATAGTCGTGGATTACTTAATGATTTAAGTTAGTTATTCTCTTAAATTGATATTTAGATTCTCTTATATTGATATTTATGTAATAAATCACTGAGGGTGAAGAATACGTAAGGAGCTCCATCATCAAATTCTTAGGTTTCTGGAATGCAACACTACCCTCTGTTAAATTGATTTAAGAGAAGCGTTGTTTATGAATTCTGTATACGATGCATTGTTGAGAATGTTGTTAAGAAAATATTTTAATACTAAAAGTAATTGCTTATTGCATGAGTGAATGATTAGATAAATAAAAATTATATTTAAATTAAACAATATAAACAAAATCCAAAAAAATGCGGCGTAGCGCGGGTGAACACCTAGTGTAAGTAAGGGAAAAATTGCTCAATAATAACATCATTGATCTCATTTCAAAGTTATTAACTAATAAATTAAGAATGAAAAATGGAGTTAATAATTTACTAACTAACCGCATTAATGAATAAAGATAATTATTTATTAGTATATGATCTTTTATATACTAAATACACATGCATCTAGTTCAAGCTAAGTGAAGGAACTATTTTCAGAGTGAAGCAACCTTTCCAAATTTATAACAAGAGAGTTCCAAGTACCAAATATCAATTTTTTCTCACTCTTAATTGTCACCATGCAAGTAACATCTTCAACTACACATACCCAAAAATTTGAAAGATTTCTTATTTTTTGTTTTATTTATAAAATTAATTGGCTCATACACGCACATATCGTCGAAGATAGAGATAGTTTACGGTGACATAGGCTGTACGAAGTTACTTTTAAGTCCATCGTACATCAAATTTTGGGTTTAAATGTTAGATATGCAATTTTTTATATGGGATATAGGATATCGATGATTTTTTTTTTTTTTTGAATAGAGTTGGTGTTATAATTTCATGTGTTGGATTATTCATTTAGGAGTAGTAGATTTTTCTTCTTTTTTTGAACAAATTAGGAGTAGTAGACTAGTAGTCAAGGTTTGTTCATTATCGAAAATAAAATTCATAATAACAGCAATAAAACTTATAAGTTATAACTCCAAATGACAAGGTGGTTCGTTGTATTTTGCTAGAAATATAAACTTGAAATTTTCAAATAATTAGTAAGTTTTAGACATTAGGTATTCTCACAAGGCAACATTCCGAAACAGTAGGAGCTACCACAAACAGCAATTTAATTTGTTGTCCGTATTTTTTCTGGTGACACGGACATCTCCATAAGTGATAATTTAAGTCGTCCTCAATAAATTTAGTTTTGTTTACACATAACATCCAAACTTTTGTTTTATAACCTTAAATTGAAAGTAAAAATATTTTGAACAGTAATTGTATTCCAAAGTTTTTTTTGTTTTTTTTTTTGCTTATAACCAGGAGTTTCAGGGCCGATGTCCTTATTCCCCCTCAGGATCCGGGTAAGACCAGGTGAAAGCCCCTGGTTAAGTGGAAATGACTAGCAATTTCGCGTTTGCGGGGAATCGATACCTGGTTTGGACCAACAGGGCAACCAACAGGGCAACGCTTCCTCAAATGAAACCACTAGAGCATGACCACTTGGTTTCTTTTCCACAATTGATAATAAATAATCAATAATAGACTGATTGGTACCATTTAAATAGGAATTTTATTTGTCATAATACTCAATCTATTTACTTTTGGAATTTTCATACCCCAACTTTTTGGTTAGAAGAAAAATACTTCATTTAAAATATCTTTGTCTAAAAATACTTCATGTTGAATTATTGGTGGATTCAAGTTTACGACTTAATAATTATTAACAAATATTACAAAATCATTTAACGACGTTAAATGTTAACCATGTGTATATAAATAGATATATAGACTTTTTATTCAAAATCTTACAAATATATTGGTGTGGTCTAGTAGTAACAAGTAGTTTATTTAATGTTACTGCGTTAATACGATTGATAAGCAGGTTTTTTACACCTACTTATATGTTGAATGTGTGTGTTCAGTTCAATTCCTCTAAAACACAAACATGTCGCTGCAGTTATAGAGGGTGTCAATCCAGTCCACTGTATTACTATCAATCAAGATGTGTACAAAAACAACTAAGTCAAGCCAAATTATAAAGATGGTGGGTTTCTAGTAACAATCCTAGATCAGGATTCAAACAAGTAAAAATGAGAACTTAACAGCTCTACTCGATCAATAGGTCGAGTAGAACCAACAGGTGACAAAATTAAGAACTAAAATGCAATCAACAGGGGATGCAAATAAAGGATACAAATAACAGGTAAATAAAAGTGCAATCAACAGGGGATACAAATAAACCTAATAGAAGTTCTAAGGATGGATTCATTGATAAGGGTAACTCTAGGGTTCATCAAATGGCTAATAGGCGAAAGGTAGATACCCATGACGATAGATTCAAAAACTGGGTCAATCCACTCTCGTGACAGAGACCCCCATAACTCTACCACTTCCTAATCTCAACTCTCGTTGATGAAACATAGCAGAGCAGGGTTTAAGAAACAATCTACCAAAGCCAATAGGCAGACAAGTCAATCCCTTGGTCGAGTCTACAGATCTCTGGAATTCGTAGGCAGACATCCATTTGAACGTCTTTTGGACGCTGGACGTCTATGATCGAATCTCCACATTAGCCACCGAAAACCGCATAGGGGCCAAGCGAATCCAGAAGGGAATAAACCTAAACACTAACCACCTAACTAACCTAGAGATTACCCTAATCTAATCCATCCTCAAGAACCCTAATCACTACTCATGCAACATTCTCAAGAACACAAATCCATAAATTACTAAAATCATACCAAAATGTAGATCTAAACAAGCTAAACAACTTTACTTAAACAATCAAATGCAAAAACGAAATAGATTAAGAATAATTAAGGTTTCAAGATTCATAAAAACAAAGAAAAACTGAAAGATAAAGCATTGATATTTTGGACAAACCCCAAAATGATCTATTTATAGTAAAATGTTGAAACCCTAGATTCACTAGAAGACAAGAGACTTGAAGCGATTTGGGCAAATACTCGATCTTGGGGATCGAGTAAAGAGGGTGCTGCAAAGTCTGCGCCTTTGTGTCTATGCTCCGAGTGATCAATTGGTCGAGTAGATGCTTCATCCTCTCCTATCCGTATGCTTAGATGTCTGGTTGAGTGGGGAATGAGGAGGATCCGAAGACGCTTTGATAGCCCAACAAGTCTTGTAGAGATGGTCACCCTAGGTCGAGTAAATGGATCGAGTAGTGGATCGAGTTGGAGGGGCGAGTAGATCAATCTGCTGCTTCATCTGAGGTTGCCTTGATCGAGTAGGAGGGTCGAGTAAACTGCAGCCTCCTCTGTGGCATCCTGGATCGAGTAAAGAGGTCAAGTAGAATGATGTCTCTTCTGTAGCTGCCTGGATCGAGTGAATGGATCTAGTAGAGTCCTTTTAAGCTCCTTTTGCACCTGAAAACACACCAAAACATGCAATGCGGATGCAAGATGATCCTAGATGCCTAAGTATGCAAAACAAGCTAAAATGGTGCAAAATGATATGATACAATGAGCAAATGCAAAGCTAAATGATGATTAACATACACCTAAAATGTGTAAAATACAGTGTTATCAACAATCAAAACTCCATCTTTCTCGAACCATTTCTTTTATCTCTCCACCTTTACCTTAATTCTTCTCCACTTCCGCAACTAACCTACTCCACGACCACAAAAATAACATATCCAGCACCGTCGTCATCTTTGTCTCAATCTCTCATGTGTTAAATGTATTTAAATTTAAATTTGTAGGAGTGAAGAAAATGATGTATTTAAATTTGTATGACGTCATTCATTATAGAAAATAAGACATCAAAAGAAGTTCTATGCAAGTTGAATAACAAACGTGTTACCACCAGATCAAGCAATATATTTGTAGAAGTTTGTAAAAATATTGTATTTATTTATTCTTACATGGAAAGTTAAAATTTAATGTAGTTTACTGATTCTAAAACGCCTCGTTAACGGCTGTTAAGGAGAGAGATTTGAATCTACTAACAATTTAAAAATAAAGTTTTTAAGACAAAAATATTTAAATGAGGTATTTTATCTTCTTTGGCTTTGGCTTTGGCTTTGTATCTTCTAGCAAGGAAACACTACTATAGCTTTTGGGATCTGGTTGCGGTTCTAGTTCTTATACTCATTCATATACATGACATCTAGTCATATTTGACTCCAAAACACTATACGTGCTTCTTCTTACTTCTCATTGTTTTGATAGTGTAGCCAAAATCCGTATGAATTTTCTGTTTTAGATCTTCTAAAAGGGAAACACTACTTTAGCTTTTGGGATCCAATTGCGGTTCTAGCTCTCATACTTAATCATACACTTGACATCTACTCATATTTGACTCCAAAACACTAACCAAGCGTTTTCTTGTTTCTCCATGCTGTGATGGTGTAGCCGAAGTCCATATGAGTTTTTTGTTTAAGATCTTCTAACAAGGAAACACTATTTAAGCTTTTTAGATCCCGTTGTTGTTCTAGTTCGTATACTCAATCATACACGTGACATCTAGTCATATTTGACTCCAAAACGCTAACAAAGCTTCTTATTGTTTTTCAATGCTTTGATGGTGTAGCCGAAGTCTGTATGAGTTTTTAGTTTTGGATCTTCTAATATGGAAACACTACTTTAACTTTTTGGATCTAGTTGCAGTTCTAGTCTTATACTTAATCATACACATGAAATCTATTCATATTTGACGCCTTTAGTTCTCAATGCTTAATGAGAAGCCCTATTCCTAACAAGCACACTACTCAGACAAATTGATTGAAGCAAACAACATAATTGAGTATGAAAGCTTAAATACAGCAATAAAGATGAGGGAAAGAAAGGATCTCTTCACTGAATTGAAACTGAATGAATCTCTGAAGAAAAGGGGATGAATAGCTTATGTCTCTCAAAATAGGGTTTGTAAAAAGCTTGATAAAAAGAAAATAGGTCTAAAACAATGTATATAAACACTATAAATCGTCCAGGGACTAATAATGCAAATAGGGAAGTTTTCTGGAGCAAATTTCCTCTTCTGTAAACTTGAAAGCGTCATGGACTTTGTTTGGCCGAAGCTAGTGTCGATCGACACCAGGAGTGTGTCGATTGACACTCCTCTTGATTCCTGAGTCCAAAAGCATCCTCAACTTCCTTTTCCTTAGCTTTTGCTCCAAAATGTCTCCTCATCTCCATTGTTGTCCCATTGCATGGAATACCTGAAAAAACACTAAAAAGACTCGAGAAATAACATAAAGACTCAAAAACCTATATCTAAAACATAGATAAAATCATTGAAAATAGGGTTCTATCAAAGATCCAAAACCAAAAACTCTAACGGACTTTTGCTACACCATCATAGCATTGAGAAGCAAATAAGCTTTGGTAGTGTTTTGGAGTCAAATATGACTAGATCTCATGTGTAAGATTGAATATAAGAACTAGACCGACAAACGGATATTGAATACTTAAGTAATGATTGCTTGTTAGAAGATACAAACCCAAAGACTCACACAAACTCTGGCTACACCCTCAAAGCTTTGAGAAGCAAGAAGAAGCTTGGTTAGTGTTTTGGAGTCAAATATAACTAGATGTCATGTGTATGATTAAGTATAAGAACTAGAAGCGCAACGTGATACCAAAAGCTAAAGTAGTGTTTCATTATAAGATGATCCAAAAACAAAAAATCATACAGACTTCGACTACACCATCAACGTATTGATAAGCAAGAATAAGCTTGGTTAGTGTTTTGGAGTCACATATGACTAGATCTAATGTGTATGATTGAGTATAAGAACTAGAACCGCAACCAGATCTTGAAAGATTAAAAGATTAAGTAGTGATTCCTTGTTAGAAAATAAAAACCCAAAGACTCATATGGACTTCGGCTACACCATCAAAGCTTTGAGAAGCAAGAAGAAGTTGTTGACGAGCTAATTGGAAGAAGCGGACCTGAAAATGTTAAACCACTCGACCCCCTGGTCGAGTAGATGCTTCACTACTTCAACAGACCACTTGACCCCGTGGTCGAGTAAAAGACCTCAGCACTCCACGTCACCACTCGACCACGTGGTCGAGTACCACCAGGACAACCACTCGATCACATCACTAGACCCCCTGGTCGAGTATCATCATGCCCACCACATCACCTTCACTCAATCACTCTACCAACAGGTCGAGTATCACTATTATCATACACTCGACCAATCACACTACCAACATGGTCGAGTATCACCATCATCATTCACTTGACCAATCACTCTACCAACAGGTCGAGTATCATCATCATCACTCACTCGACCAATCACTCTACCAACAGGTCGAGTATCACCATCATCATTCACTCGACCAATCACTCTACCAACATGGCCGAGTTCCACCATTATCATTCACTCGACCAATCACTCTACCACCAAGTCGAGTACTACCACCACCATCCACTCGACCAATCACTCTACCACATGGTCGAGTCCCTTATCATCATTCACTCGACGTCTTTACTCGACTGCCAGCTTCAGAGTCTTCTCCATTCCGCACTCAACCAGACACTCGAGCACAAGGAAGAAAAGAAGACTTTAGCTTATCACTCGACCACTCACTCGACCACCTGGGTCGAGTACTGTTCTTAATCCGTCCTAATACTGCGTCGTTTTGAGTATTAGGGTTTGAGAAATATTTCGCTATAAGTAGCATGCACTTAACATTTTCGACAAGAAGTTTTTTATCTAGTTTTATTTCCGCAGACTTAGATTTGGTTAATGAACTTGTTATCTAGGGTTAATTTATTGAGCATGCCAATCACCTGTAAACTGAGGAAACGAAACTACTCAATTACCCCATCCTTGGGAATTCTTTATCTGATTGAATTCTTTGTTTACTCTACTAATGTTTACTACATCTTGTCATCTGTTGCTTAGTTTCTGCAATTCTTTACTGTTACTCGACCTAATACTCGACCACCCAGTTGTCTGGCAACACACTGTGCAGTCGAGTATCCCTGTTATACTTTCTGTCTGTTACTCGACCTATCACTCGACCTCACCCAGTCCTCTGCAAAGAGCTGTGTTGGTCGAGTGTTTTATTGTTTTTGCTTGAATTTCTGTTTTCTGCATGTTCACTTAGAACTTCTAGAACACACCAAGACCTGTTATTGCTTGGCTTGACTTAGTGACTTCTGATCACATCTTGATTGTTAGCCTCACACCCATTTGGATTGACAACCTTCAGTACTACAACGACATGATAGTGTTTTAGGATTAATTGATTTTAAAAACCTATTATCACTTCTAACAAGGAAACACTACTTAGGCTTTTAAGATCGGGTTGCGGTTTAAGTTTTTATACTCAATCATACACATGACATCAAGTCATATTCGACTCCAAAACACTAACCAAGCTTCTTCTTGCTTCTCAAAGCTTTCATGGTGTAGCCAAAGTCCGTATGAGTCTTTGGCTTTGTGTCTTCTAACAAGGATACAATTCTTACGGCTTTAAGATCCGATTGCGGTTTAAGTTCTTATACTCAATCATACACATGACATCAAGTCATATTCTACTCCAAAACACTAACCAAGCCTCTTCTAGCTTCTCCAAGCTTTCATGGAGTAGCCAAAGTCCGTACGAGTCTTTGGCTTTGTATCTTCTAACAAGGAAACACTACTTAGGCTTTTAAGATCGGGTTGCGGTTTAAGTTTTTATACTCAATCATACACATGACATAAAGTCATATTCGATTCCAAAACACTAACCAAGCTTCTTCTAGCTTCTCAAAGCTTTCATGGTGTAGCCAAAGTCCGTATGAGTCTTTGGCTTTGTATCTTCTAACAAGGAAACACTACTTAGGCTTTTAAGATCGAGTTGCGGTTTAAGTTCTTATACTCAATCATACACATTACATCAAGTCATATTTGACTCCAAAACACTAACTAAGCCTCTTCTAGCTTCTCCAAGCTTTCATGGTTTAGCCAAATTCTGTATGAGTTTTTGGCATTGTATCTTCTAACAAGGAAACACTACGTAGGCTTTTAAGATTGGGTTGCGGTTTAAGTTCTTATACTTAATCATACACATGACATATAGTCATATTTGACTTCAAAACACTAACCAAGCTTCTTCTAGCTTCTCAAAGCTTTCATGGTGTAGCCAAAGTCCGTATGAGTCTTTGGCTTTGTATCTTCTAACAAGGAAACACTACTAAGGCTTTTAAGATCGGGTTGCGATTTAAGTTTTTATACTCAATCATACACATGACATCAAGTCATATTCGACTCCAAAACACTAACCAACCTTCTTCTTGCTTCTCAAAGCTTTCATGGTGTAGCCAAAGTCCGTATGAGTCTTTGGCTTTGTGTCTTCTAACAAGGATACAATTCTTACCGCTTTAAGATACCATTATGGTTTAAGTTCTTATACTCAATCATACACATGACATCTAGTCATATTCTACTCCAAAACACTAACCAAGCTTCTTCTAGCTTCTCAAAGCTTTCATGGTGTAGCCAAAGTCCGTATGACTCTTTGGCTTTGTATCTTCTAACAAGGAAACACTACTTAGGCTTTTAAGATCGAGTTGCGGTGTAAGTTCTTATAATCAATCATACACATGACCTCAAGTCATATTCGACTCCAAAACACTAACCAAGCCTCTTCTAGCTTTTCCAAGCTTTCATGGTGTAGCCAAAGTCCGTATGAGTCTTTGGCTTTGTATCTTCTAACAAGGAAACACTACTTAGGCTTTTAAGATCGGGTTGCGGTTTAAGTTCTTATACTCAATCATACACATGACATAAAGTCATATTCGACTCCAAAACACTAACCAAGCTTCTTCTAGCTTCTCAAAGCTTTCATGGTGTAGCCAAAGTCCGTATGAGTCTTTGGCTTTGTATCTTCTAACAAGGAAACACTACTTAGGCTTTTAAGATCGAGTTGCGGTTTAAGTTCTTATACTCAATCATACACATTACATCAAGTCATATTTGACTCCAAAACACTAACTAAGCCTCTTCTAGCTTCTCCAAGCTTTCATGGTTTAGCCAAATTCTGTATGAGTTTTTGGCATTGTATCTTCTAACAAGGAAACACTACGTAGGCTTTTAAGATTGGGTTGCGGTTTAAGTTCTTATACTTAATCATACACATGACATATAGTCATATTTGACTCCAAAACACTAACCAAGCTTCTTCTAGCTTCTCAAAGCTTTCATTGTGTAGCCAAAGTCCGTATGAGTCTTTGGCTTTGTATCTTCTAACAAGGAAACACTACTAAGGCTTTTAAGATCGGGTTGCGATTTAAGTTTTTATACTCAATCATACACATGACATCAAGTCATATTCGACTCCAAAACACTAACCAACCTTCTTCTTGCTTCTCAAAGCTTTCATGGTGTAGCCAAAGTCCGTATGAGTCTTTGGCTTTGTGTCTTCTAACAAGGATACAATTCTTACCGCTTTAAGATACCATTATGGTTTAAGTTCTTATACTCAATCATACACATGACATCTAGTCATATTCTACTCCAAAACACTAACCAAGCTT

**T6C20sequence**

ctacctctcagaaaaataaatttatttcggtgaaatctgaagtgttgagttcgcaattca

caagaatagatttcatccctcaaggatttttccaagtttcacctcttggaaaaatatatt

catttcggtgaaatctaagtgttgagttcgctcttcacaagactagatttcatcactcaa

ggatttttccaagttcctccttttggaaaaatatattcatttcggttaaatctaagtgtt

gagctcgctcttcacaagactagatttcatccttcaaggatttttccaagttacaccttt

tagaaaaatatattcatttcggtgaaatggaagtgttgaattcgctcttcataaaactag

atttcatccctcaaggatttttccaagttgcacttcttggaaaaatatattcatttcggt

gaaatctaagtgttgagtttgcgcttcacaagactagatttcatccgtcaaggatttttc

caagttccacctcttggaaaaatatattcatttcggtgaaatctaagtgttgagttcact

cttcacaagactagatttcatccctcaaggatttttccaagttccacctcttggaaaaat

atattcatttcggtgagatctaagtgttgagctcgcttttcaaaatactagattttatcc

ctcaaagatttttccaatttgcatttcttgaaaaaatatattcatttcggtgaaatctaa

gagttgagttcgcgtttcacaagactagatttcattcctcaagaatatttccaagttgca

cttcttggaaaaatgtattcatttcggtgaaatctaagtgttgagtttgcgcttcacaag

actagatttaatccgtcaaggattttttcaagttccacctcttggaaaaatatattcatt

ttagtgaaatctaagtgttgagttcgctcttcacaaaattagattttaaatctcaaggat

ttttccaagttccacctctcggaaaaatatattcatttaagtgaaatcttaattgttgag

ttcgcacttcacaagactaaaattcatccctcaaggatttttccaagttccacctcttgc

aaaaatatattcatttcggtgaaatctaagtgtggagttcgctctttacaagactagatt

tcatccctcaaggatttttccaagttcyacctcttggaaaaatatattcatttcggtgaa

atctaagtgttgagctcgctcttcacaagactagattgkatccttcaaggatttttccaa

gttacacctttgaaaaaaatatattcatttcggtgaaatgkaagtgttgagttcgctctt

cacaagactagatttcatccctcaaggatttttccaagttgcacttcttggaaaaatata

ttcatttcagggaaatctaagtgttgagtttgctcttcacaagactagatttcatccctt

aaggatttttccaagttacacctcttggaaaaatatattcattccggtgaaatctaagtg

ttgagttcgctcttcacaagactagatttcatccctcaaggatttttccaagttctacct

cttggaaaagtatagtcatttcggtgaaatttaagtgttgagttcgcttttcaccagacc

agatttcatccctcaagaatttttccaagttccacctctttggaaaaatatattcatttt

ggttaaatctacgtgttaaatttactattcacaaaactagatttcatccatcaaaaagtt

ttccaagttccacctctcggaaaaatatatccatttcggtaaaatctaagtgttgagttc

cctcttcacaaaactatatttcatccctcaagaatttttccaagtttcacctcttgaaaa

aatatattcatttcagcraaatctaagtgttgagctcgctcttcacaagactagatttaa

tccctcaaagatttttccaagttccacctcttggaaaaatatattcatttcggtgaaatc

taattgttgagttagctcttcacaaaactagatttcatccctcaaggatttttccaagtt

ccacctctccgaaaaatatattcattttggagaaatctaagtgttgaactcgcttatcac

aagactagatttcacccttcaaggatttttcctagttccacctcttaaaaaaatataatc

atttcggtgaaatccaagtgttgagttcgctcttcacaagattagatttcatccctcaag

gatttttcaaagttccacctcttgaaaaaatgtattcattttggtgaaatgtaagtgttg

agttcgctcttcagaagactagatttcatccctcaaggatttttccaagttccacctctt

ggaaaaatatattcatttcggtgaaatctaagtgttgagctcgctcttcacaagactaga

ttgcattcttcaaggatttttccaagttacaccttttggaaaaatatattcatttcggtg

aaatccaagtgttgaatatgctcttcataagactagatttcattcctcaaggatttttcc

aagttgcatttcttggaaaaatatattcatttcagtgaaatctaaatgttgagttcgcgt

ttcacaagactagatttcatccctcaaggatttttccaagttccacctcttggaaaaata

tattcattttggtgaaatctaagtgttgagtttgctattcacaaaactagatttcatccc

tcttaaagttttccaagttccacctctcggaaaaatatattcatttcggtgaaatattaa

gtgttgagttcgcaattcacaagactagatttgatccctcaaagatttttcatagttcca

cctctcggaaaaaaatatattcatttcggtgaaatctaagtgttgagcttgctcttcaca

agactagatttcatccctcaaaagttttt

**[gapbp]**[**ExpandNs**](http://www.ncbi.nlm.nih.gov/nuccore/14192923?fmt_mask=65536)

ttggaaaaatatattcatttcggtgaaatctaagtgttgagtttgc

gcttcacaagactagatttcatccgtcaaggatttttccaagtttcacctcttgaaaaaa

tatatttattttggtgaaatctaagtgttgagtttgctcttcacaaaactagattttatc

tctcaaggatttttccaagttccacctctcggaaaaatatattcatttaagtaaaatatt

aagtgttgatttcgcacttcacaagactagaattcatccctctaggatttttccaagttc

cacctcttggaaaaatatattcatttcggtgaaatctaagtgttgtgttcactcatcaca

agagtagaattcatccctcaaggacttttccaagttccacctcttcgaaaaatatattca

tttcggtgaaatctaagtgttgagctcgctcttcacaagactagatttcatccgtcaagg

atttttccaagttgcatttcttgaaaaaatatattcatttcagtgaaatctaagtgttga

gttcgcgcttcacaagactagatttcatccctcaaggatttttccaagttgcatttcttg

gaaaaatatattcatttagatgaaatctaagtgttcagttcgcgcttcacaagactagat

ttcatccctcaaggatttttccaagttccacctcttggaaaaatatattcatttttggtg

aaatctaagtgttgagtttgctcttcacaaaactagattttatctctcaaggatttttcc

aagttctacctctcagaaaaatatatttatttcggtgaaatctgaagtgttgagttcgca

attcacaagactagatttcatccctcaaggatttttccaagtttcacctcttggaaaaat

atattcatttcggtgaaatctaagtgttgagttcgctcttcacaagactagatttcatcc

ctcaaggacttttccaagttccaccatttggaaaaatatattcatttcggtgaaatctaa

gtgttgagctcgctcttcacaagactagatttcatccttcaaggatttttccaagttaca

ccttttaaaaaaatatattcatttcggtgaaatggaagtgttgagttcgctattcacaag

actagatttcatccctcaaggatttttccaaattgcacttcttggaaaaatatattcatt

tcggtgaaatctaactgttgagtttgcgcttcacaagactagatttcatccgtcaaggat

ttttccaagttccacctcttggaaaaatatattcattttggtgaaatctaagtgttgagt

tttctcttcacaaaactagattttatctctcaaggattttttcaagttccacctctcgga

aaaatatattcatttaagtgaaattttaagtgttgagttcgcactttacaagactagaat

tcatccctcaaggatttttccaagttctacctcttggaaaaatatattcatttcggtgaa

atataagtgttgagttcgctcttcacaagactagatttcatcactcaaagatttttccaa

gttccacctcttggaaaaaaatatattcatttcggtgaaatttaagtgttgagcttgctc

ttcacaagactagatttcatccctcaaggatttttcccagttacaccttttggaaaaata

tattcattttggtgaaatgtaagtgttgagttcgctcttcacaagactagatttcatccc

tcaaggatttttccaagttgcactttttggaaaaatatattcatttcggtgaaatctaag

tgttgagtttgcgcttcacaagactagatttcatccgtcaaggatttttccaagtttcac

ctcttaaaaaaatatatttattttggtgaaatctaagtgttgagtttgctcttcacaaaa

ctagattttatctctcaaggatttttccaagttccacctctcggaaaaatatattcattt

aagtaaaatattaagtgttgatttcgcacttcacaagactagaattcatccctctaggat

ttttccaagttccacctcttggaaaaatatattcatttcggtgaaatctaagtgttgagt

tcgctcttcacaagactagatttcatccctcaaggacttttccaagttccgccttttgga

aaaatatattcatttcggtgaaatctaagtgttgagctcgctcttcacaagactagattt

catccttcaaggatttttccaagttacaccttttaaaaaaatatattcatttcggtgaaa

tggaagtgttgagtttgctcttcacaagactagatttcatccctgaaggatttttccaaa

ttgcacttcttcgaaaaatatattcatttcggtgaaatctaagtgttgagtttgcgcttc

acaaaactagattttatccctcaaggatttttccaagttccacctcttggaaaaatatat

tcatttcggtgaaatctaagtgttgagctcgctcttcacaagactagatttcatccgtca

aggatttttccaagttgcatttcttgaaaaaatatattcatttcagtgaaatctaagtgt

tgagttcgtgcttcacaagactagatttcatccctcaaggatttttccaagttgcatttc

ttggaaaaatatattcatttaagtaaaatattaagtgttgagttcgcacttcacaagact

agaattcatccctctaggatttttccaagttccacctcttggaaaaatatattcatttcg

gtgaaatctaagtgttgagttcgctcttcacaagactagatttcatccctcaaggacttt

tccaagttccgccttttggaaaaatatattcatttcggtgaaatctaagtgttgagctcg

ctcttcacaagactagatttcatccttcaaggatttttccaagttacaccttttaaaaaa

atatattcatttcggtgaaatggaagtgttgagtttgctcttcacaagactagatttcat

ccnnnnnnnnnnnnnnnnnnnnnnnnnnnnnnnnnnnnnnnnnnnnnaaaccgcaagctg

atcttaaaagcctaagttgtgtttccttgttagaagacacaaagccaaagactcatatgg

actttggctacaccatgaaagctttgagaagcaagaagaaggttggttagtgttttggag

tcgaatatgacttgatgtcatgtgtatgattgagtataagaacttaaaccgcaacccaat

cttataagcctaagtagtgtttccttgttagaagatacaaagccaaagactcatacggac

tttgactacaccatgaaagctttgagaagcaagaagaaggttagttagtgttttggagtc

gaatacgacttgatgtcatgtgtatgattgagtataacaacttaaaccgcaaccctatct

taaaagcctaagtagtgtttccttgttaaaagacacaaaggcaaagactcatatggactt

tggctacaccgtgaaagctttgagaagcaacaagaaggttggttagtgttttagagtgaa

tatgacttgatgccatgtgtaagattcattataacaacttaaaccgcaacctgatcttaa

aagcctaagtagtgttaccgtgttagaagacacaaagccaaagactcatatagactttgg

ctacaccatgaaagctttcagaaaggttggttacatatttggagtcgaataggacttgat

gtcatgtgtatgattgagtataacaacttaaaccgcaatctgatcttaaaagcctaagta

gtgtttccttgttagaagacacaaagccaaagactcatatggactttgcctacaccatga

aagctttaaaaagaaagaagaatgttggttagcgttttggagtcgaatatgacttgatgt

catgtgaatgattgagtataagaacttaaaccgcaacccgatcttaaaagcctaagtagt

gtttccttgttaaaagatacaaagccaaagactcatacggactttgactacaccatgaaa

gctttgagaagcaagaagaagtttggttagtgttttggagttgaatatgacttgatgtcg

tgtgtatgattgagtataagaacttaaaccgcaactggatcttaaaggcgtaagaattgt

atccttgttagaagacacaaagccaaagactcatatggaatttggctacaccatgaaagc

tttgagaagcaagaagaaggttggttagttgtttggagtcgaatatgacttgatgtcatg

tgtatgattgagtataagaacttaaaccacaacccgatcttataagcctaagtagtgttt

ccttgttagaagatacaaagccaaagattcatacggattttgactacaccatgaaagcct

tgagaagcaagaagaaggttggttagtgttttggagtcgaatatgacttgatgtcatgtg

tatgattgagaaaaayaacttaaaccgcaacccgatcttaagagcctaagtagtgtttcc

ttgttagaagacacaaagccaaagactcatatggactttggctacaccatgaaagctttg

agaagcaagaagaaggttggcttagtgttttggagtgaatatgacttgatgtcatgtgta

tgattgagtataagaatttttaccgcaacccgatcttaaaagcctaagtagtgtttcctt

gttagaagacacaaagccaaagactcatatggactttggttacaccatgaaagctttgag

aagcaagaagaaggttggttagtgttttggagtcgaatatgacttgatgtcatgtgtatg

attgagtataagaacttaaaccgcaaccggatcttaaaggcgtaagaattgaatccttat

tagaagacacaaagccaaagactcatatcgactttggctacaccatgaaagctttgagaa

gcaagaagaaggttggttagtgttttggagtcgaatatgacttgttgtcatgtgtatgat

tgagtataacaacttaaaccgcaagttgatcttaaaagcctaagtagtgtttccttgtta

gaagacacaaagcctaagactcatatggactttggctacaacatgaaagctttgagaagc

aagaagaaggttggttagtgtttaggagtcgaatatgacttgatgtcatgtgtatgattg

agtataagaaattaaaccgcaacccgatcttataagcctaagtagtgtttccttgttaga

agatacaaagccaaagactcataaggactttgactacaccatgaaagctttgagaagcaa

caagaaggttagttagtgttttggagtcgaatatgacttgatgtcatgtgtatgattgag

tataagaacttaaaccgcaaccctatcttaaaagcctaagtagtatttccttgttagaag

acacaaagccgaagactcatatggactttggctacaccgtggaagctttgagaagcaaca

agaaggttggttagtgttttagagtgaatatgacttgatgtcatgtgtatgattgagtat

aagaacttaaactacaacccgatcttaaatgcctaagtagtgtttccttgttagaagaca

caaagccaaagactcatatggactttggttacaccatgaaagctttgagaagcaagaaga

aggttggttagtgttttggagtcaaatatgacttgttgtcatgtgtaagattcattataa

caacttaaactgcaacctgatcttaaaagcctaagtagtgtttccgtgttagaagacaca

aagccaaagactcatatagactttggccacaccatgaaagctttgagaagcaagaagaag

gttggttacatatttggagtcgaataggacttgatgtcatgtgtatgattgagtataaca

acttaaaccgcaacctgatcttaaaagcctaagtagtgtttccttgttagaagacacaaa

gccatagactcatatggactttgcctacaccatgaaagcttgagtattctatagtgtcac

ctaaatagcttggcgtaatcatggtcatagctgtttcctgtgtgaaattgttatccgctc

acaattccacacaacatacgagccggaagcataaagtgtaaagcctggggtgcctaatga

gtgagctaactcacattaattgcgttgcgctcactgcccgctttccagtcgggaaacctg

tcgtgccagctgcattaatgaatcggccaacgcgaaccccttgcggccgcccgggccgtc

gaccaattctcatgtttgacagcttatcatcgaatttctgccattcatccgcttattatc

acttattcaggcgtagcaaccaggcgtttaagggcaccaataactgccttaaaaaaatta

cgccccgccctgccactcatcgcagtactgttgtaattcattaagcattctgccgacatg

gaagccatcacaaacggcatgatgaacctgaatcgccagcggcatcagcaccttgtcgcc

ttgcgtataatatttgcccatggtgaaaacgggggcgaagaagttgtccatattggccac

gtttaaatcaaaactggtgaaactcacccagggattggctgagacgaaaaacatattctc

aataaaccctttagggaaataggccaggttttcaccgtaacacgccacatcttgcgaata

tatgtgtagaaactgccggaaatcgtcgtggtattcactccagagcgatgaaaacgtttc

agtttgctcatggaaaacggtgtaacaagggtgaacactatcccatatcaccagctcacc

gtctttcattgccatacggaattccggatgagcattcatcaggcgggcaagaatgtgaat

aaaggccggataaaacttgtgcttatttttctttacggtctttaaaaaggccgtaatatc

cagctgaacggtctggttataggtacattgagcaactgactgaaatgcctcaaaatgttc

tttacgatgccattgggatatatcaacggtggtatatccagtgatttttttctccatttt

agcttccttagctcctgaaaatctcgataactcaaaaaatacgcccggtagtgatcttat

ttcattatggtgaaagttggaacctcttacgtgccgatcaacgtctcattttcgccaaaa

gttggcccagggcttcccggtatcaacagggacaccaggatttatttattctgcgaagtg

atcttccgtcacaggtatttattcgcgataagctcatggagcggcgtaaccgtcgcacag

gaaggacagagaaagcgcggatctgggaagtgacggacagaacggtcaggacctggattg

gggaggcggttgccgccgctgctgctgacggtgtgacgttctctgttccggtcacaccac

atacgttccgccattcctatgcgatgcacatgctgtatgccggtataccgctgaaagttc

tgcaaagcctgatgggacataagtccatcagttcaacggaagtctacacgaaggtttttg

cgctggatgtggctgcccggcaccgggtgcagtttgcgatgccggagtctgatgcggttg

cgatgctgaaacaattatcctgagaataaatgccttggcctttatatggaaatgtggaac

tgagtggatatgctgtttttgtctgttaaacagagaagctggctgttatccactgagaag

cgaacgaaacagtcgggaaaatctcccattatcgtagagatccgcattattaatctcagg

agcctgtgtagcgtttataggaagtagtgttctgtcatgatgcctgcaagcggtaacgaa

aacgatttgaatatgccttcaggaacaatagaaatcttcgtgcggtgttacgttgaagtg

gagcggattatgtcagcaatggacagaacaacctaatgaacacagaaccatgatgtggtc

tgtccttttacagccagtagtgctcgccgcagtcgagcgacagggcgaagccctcgagtg

agcgaggaagcaccagggaacagcacttatatattctgcttacacacgatgcctgaaaaa

acttcccttggggttatccacttatccacggggatatttttataattattttttttatag

tttttagatcttcttttttagagcgccttgtaggcctttatccatgctggttctagagaa

ggtgttgtgacaaattgccctttcagtgtgacaaatcaccctcaaatgacagtcctgtct

gtgacaaattgcccttaaccctgtgacaaattgccctcagaagaagctgttttttcacaa

agttatccctgcttattgactcttttttatttagtgtgacaatctaaaaacttgtcacac

ttcacatggatctgtcatggcggaaacagcggttatcaatcacaagaaacgtaaaaatag

cccgcgaatcgtccagtcaaacgacctcactgaggcggcatatagtctctcccgggatca

aaaacgtatgctgtatctgttcgttgaccagatcagaaaatctgatggcaccctacagga

acatgacggtatctgcgagatccatgttgctaaatatgctgaaatattcggattgacctc

tgcggaagccagtaaggatatacggcaggcattgaagagtttcgcggggaaggaagtggt

tttttatcgccctgaagaggatgccggcgatgaaaaaggctatgaatcttttccttggtt

tatcaaacgtgcgcacagtccatccagagggctttacagtgtacatatcaacccatatct

cattcccttctttatcgggttacagaaccggtttacgcagtttcggcttagtgaaacaaa

agaaatcaccaatccgtatgccatgcgtttatacgaatccctgtgtcagtatcgtaagcc

ggatggctcaggcatcgtctctctgaaaatcgactggatcatagagcgttaccagctgcc

tcaaagttaccagcgtatgcctgacttccgccgccgcttcctgcaggtctgtgttaatga

gatcaacagcagaactccaatgcgcctctcatacattgagaaaaagaaaggccgccagac

gactcatatcgtattttccttccgcgatatcacttccatgacgacaggatagtctgaggg

ttatctgtcacagatttgagggtggttcgtcacatttgttctgacctactgagggtaatt

tgtcacagttttgctgtttccttcagcctgcatggattttctcatactttttgaactgta

atttttaaggaagccaaatttgagggcagtttgtcacagttgatttccttctctttccct

tcgtcatgtgacctgatatcgggggttagttcgtcatcattgatgagggttgattatcac

agtttattactctgaattggctatccgcgtgtgtacctctacctggagtttttcccacgg

tggatatttcttcttgcgctgagcgtaagagctatctgacagaacagttcttctttgctt

cctcgccagttcgctcgctatgctcggttacacggctgcggcgagcgctagtgataataa

gtgactgaggtatgtgctcttcttatctccttttgtagtgttgctcttattttaaacaac

tttgcggttttttgatgactttgcgattttgttgttgctttgcagtaaattgcaagattt

aataaaaaaacgcaaagcaatgattaaaggatgttcagaatgaaactcatggaaacactt

aaccagtgcataaacgctggtcatgaaatgacgaaggctatcgccattgcacagtttaat

gatgacagcccggaagcgaggaaaataacccggcgctggagaataggtgaagcagcggat

ttagttggggtttcttctcaggctatcagagatgccgagaaagcagggcgactaccgcac

ccggatatggaaattcgaggacgggttgagcaacgtgttggttatacaattgaacaaatt

aatcatatgcgtgatgtgtttggtacgcgattgcgacgtgctgaagacgtatttccaccg

gtgatcggggttgctgcccataaaggtggcgtttacaaaacctcagtttctgttcatctt

gctcaggatctggctctgaaggggctacgtgttttgctcgtggaaggtaacgacccccag

ggaacagcctcaatgtatcacggatgggtaccagatcttcatattcatgcagaagacact

ctcctgcctttctatcttggggaaaaggacgatgtcacttatgcaataaagcccacttgc

tggccggggcttgacattattccttcctgtctggctctgcaccgtattgaaactgagtta

atgggcaaatttgatgaaggtaaactgcccaccgatccacacctgatgctccgactggcc

attgaaactgttgctcatgactatgatgtcatagttattgacagcgcgcctaacctgggt

atcggcacgattaatgtcgtatgtgctgctgatgtgctgattgttcccacgcctgctgag

ttgtttgactacacctccgcactgcagtttttcgatatgcttcgtgatctgctcaagaac

gttgatcttaaagggttcgagcctgatgtacgtattttgcttaccaaatacagcaatagt

aatggctctcagtccccgtggatggaggagcaaattcgggatgcctggggaagcatggtt

ctaaaaaatgttgtacgtgaaacggatgaagttggtaaaggtcagatccggatgagaact

gtttttgaacaggccattgatcaacgctcttcaactggtgcctggagaaatgctctttct

atttgggaacctgtctgcaatgaaattttcgatcgtctgattaaaccacgctgggagatt

agataatgaagcgtgcgcctgttattccaaaacatacgctcaatactcaaccggttgaag

atacttcgttatcgacaccagctgccccgatggtggattcgttaattgcgcgcgtaggag

taatggctcgcggtaatgccattactttgcctgtatgtggtcgggatgtgaagtttactc

ttgaagtgctccggggtgatagtgttgagaagacctctcgggtatggtcaggtaatgaac

gtgaccaggagctgcttactgaggacgcactggatgatctcatcccttcttttctactga

ctggtcaacagacaccggcgttcggtcgaagagtatctggtgtcatagaaattgccgatg

ggagtcgccgtcgtaaagctgctgcacttaccgaaagtgattatcgtgttctggttggcg

agctggatgatgagcagatggctgcattatccagattgggtaacgattatcgcccaacaa

gtgcttatgaacgtggtcagcgttatgcaagccgattgcagaatgaatttgctggaaata

tttctgcgctggctgatgcggaaaatatttcacgtaagattattacccgctgtatcaaca

ccgccaaattgcctaaatcagttgttgctcttttttctcaccccggtgaactatctgccc

ggtcaggtgatgcacttcaaaaagcctttacagataaagaggaattacttaagcagcagg

catctaaccttcatgagcagaaaaaagctggggtgatatttgaagctgaagaagttatca

ctcttttaacttctgtgcttaaaacgtcatctgcatcaagaactagtttaagctcacgac

atcagtttgctcctggagcgacagtattgtataagggcgataaaatggtgcttaacctgg

acaggtctcgtgttccaactgagtgtatagagaaaattgaggccattcttaaggaacttg

aaaagccagcaccctgatgcgaccacgttttagtctacgtttatctgtctttacttaatg

tcctttgttacaggccagaaagcataactggcctgaatattctctctgggcccactgttc

cacttgtatcgtcggtctgataatcagactgggaccacggtcccactcgtatcgtcggtc

tgattattagtctgggaccacggtcccactcgtatcgtcggtctgattattagtctggga

ccacggtcccactcgtatcgtcggtctgataatcagactgggaccacggtcccactcgta

tcgtcggtctgattattagtctgggaccatggtcccactcgtatcgtcggtctgattatt

agtctgggaccacggtcccactcgtatcgtcggtctgattattagtctggaaccacggtc

ccactcgtatcgtcggtctgattattagtctgggaccacggtcccactcgtatcgtcggt

ctgattattagtctgggaccacgatcccactcgtgttgtcggtctgattatcggtctggg

accacggtcccacttgtattgtcgatcagactatcagcgtgagactacgattccatcaat

gcctgtcaagggcaagtattgacatgtcgtcgtaacctgtagaacggagtaacctcggtg

tgcggttgtatgcctgctgtggattgctgctgtgtcctgcttatccacaacattttgcgc

acggttatgtggacaaaatacctggttacccaggccgtgccggcacgttaaccgggctgc

atccgatgcaagtgtgtcgctgtcgacgagctcgcgagctcggacatgaggttgccccgt

attcagtgtcgctgatttgtattgtctgaagttgtttttacgttaagttgatgcagatca

attaatacgatacctgcgtcataattgattatttgacgtggtttgatggcctccacgcac

gttgtgatatgtagatgataatcattatcactttacgggtcctttccggtgatccgacag

gttacggggcggcgacctcgcgggttttcgctatttatgaaaattttccggtttaaggcg

tttccgttcttcttcgtcataacttaatgtttttatttaaaataccctctgaaaagaaag

gaaacgacaggtgctgaaagcgagctttttggcctctgtcgtttcctttctctgtttttg

tccgtggaatgaacaatggaagtccgagctcatcgctaataacttcgtatagcatacatt

atacgaagttatattcgatgcggccgcaaggggttc

[gapbp][ExpandNs](http://www.ncbi.nlm.nih.gov/nuccore/14192923?fmt_mask=65536)

cgaatatgacttgatgtcatgtgtatgattgagtataag

aacttaaacagcaacccgatcttaaaagcctaagtagtgtttccttgtgagaagacacaa

aaccaaagactcatatggactttggctacaccatgaaagctttgagaagcaagaagaagg

ttggttagtgttttggagtcgaatatgacttgatgtcatgtgtatgattgagtataagaa

cttaaaccgcaacccgatcttataagcctaagtagtgttgccttgttagaagacacaaag

ccaaatctcatatggactttggctacaccatgaaagctttgagaaggaagaagaaggttt

gttattgttttggagtcgaatatgacttgatgtgatgtgtataattgagtataagaactt

aaaccgcaacccgatcttataagcctcagtagtgttgccttgttagaagacacaaagcca

aagactcatatggactttggctacaccatgaaagctttgagaagcaagaagaaggttggt

tagtgttttggagtcgaatatgacttgttgtcatgtgtatgattgagtataacaacttaa

accgtaacccgatcttaaaagcctaaataatgtttccttgttagaagacacaaagccaaa

gactcatatgggctttggctacaccatgaaagctttgagaagcaaaaagaaggtttgtta

gtgttttggagtcgaatatgacttgatgtcatgtgtatgattgagtaaaagaacttaaac

cgcaaccggatcttaaaggagtaagaattgtatccttgttagaagacacaaagacaaaga

ctcatatggaatttggctacaccatgaaagctttgagaagcaagaagaaggttggttagt

gttttggagtgaatatgacttaatgtcatgtgtatgattgagtattagaacttaaaccgc

aacccgatcttaaaagccgtaagaattgtatccttgttagaagacacaaagccaaagaat

catacggactttggctacaccatgaaagctttgagaagcaagaagaaggttggttagtgt

tttggagtcgaatatgacttgatgtcatgtgtatgattgagtataaaaacttaaaccgca

acccgatcttaaaagcctaagtagtgtttccttgttagaagatacaaagccaaagactca

tacggactttggctacaccatgaaagcttggataaggtagaagaggcttggttagtgttt

tggagtcgaatatgacttgatgtcatgtgtatgattgagtataagaacttaaaccgcaac

tcgatcttaaaagcctaagtagtgtttccttgttagaagatacaaagccaaagactcata

cggacattggctacaccatgaaagctttgagaagctaaaagaagcttggttagtgttttg

gagtcgaatatgactttatgtcatgcgtatgattgagtataaaaacttaaaccgcaaccc

gatcttaaaagcctaagtagtgtttccttgttagaagatacaaagccaaagactcatacg

gactttggatacaccatgaaagctttgagaagctagaagaagcttggttagtgttttgga

gtcgaatatgacttgatgtcatgtgtatgattgagtataagaacttaaaccgcaactcga

tcttaaaagcctaagtagtgtttccttgttagaagacacaaagccaaggactcatatgga

ctttgggctacaccatgaaagctttgagaagcaacaagaaggttggttagtgttttggag

tcgaatataacttgatgtcatgtgtatgattcagtataagaacttaaaccgcaacccgat

cttataagcctaagtagtgtttccttgttagaagatacaaagccaaagactcatacggac

tttgactacaccatgaaagctttgagaagcaagaagaaggttggttagtgttttggagtc

gaatatgacttgatgtcgtgtgtatgattgagtataagaacttaaaccgcaaccggatct

taaaggcgtaagaattgtatccttgttagaagacacaaagccaaagactcatatggactt

tggctacaccatgaaagctttgagaagcaagaagaaggttggttagtggtttagagtcga

atatgacttgatgtcatgtgtatgattgagtataagaacttaaaccgcaaatcttataag

cctaagtagtgtttccttgttagaagatacaaagccaaagattcatacggattttgacta

caccgtgaaagccttgagaagcaagaagaaggttggttagtgtttttgagtcgaatatta

cttgatgtcatatgtatgattgagaaaaacaacttaaaccgcaacccgatcttaagagcc

taagtagtgtttccttgttagaagacacaaagccaaagactcatatggactttggctaca

ccatgaaagctttgagaagcaagaagacggttggttagtgttttggagtgaatatgactt

gatgtcatgtgtatgattgagtataagaattttaaccgcaacccgatcttaaaagcctaa

gtagtgtttccttgttagaagacataaagccaaagactcatatggactttggttacacca

tgaaatcattgagaagaaagaagaaggttggttagtgtttgtgagtcgaatatgacttga

tgtcatgtgtatgattgaatataagaacttaaacagcaaccggatcttaaaggcgtaaga

attgtatccttattagaagacacaaagccaaagactcatatggactttggctacaccatg

aaagctttgataagcaagaagaaggttggttagtgttttggagtcgaatatgacttgttg

tcatgtgtgtgattgagtataacaactcaaaccgcaagctgatcttaaaaacctaagtaa

tgtttccttgttagaagacacaaagccaaagactcatatggactttggctacaccatgaa

agctttgagaagcaagaagaaggttggttagtgttttggagtcgaatatgacttgatgtc

atgtgtatgattgagtataagaacttaaaccccaacctaatcttataagcctaagtagtg

tttccttgttagaagatacaaagccaaagactcatacggactttgactacaccatgaaag

ctttgagaagcaagaagaaggttagttagtgttttggagtcgaatatgacttgatgtcat

gtgtatgattgagtataacaacttaaaccgcaacccgatcttaaaagcctaagtagtgtt

tccttgttagaagacacaaagccaaagactcatatggactttggctacaccgtgaaagct

ttgagaagcaagaagaaggttggttagtgttttggagtgaatatgacttgatgccatgtg

tatgattgagtataagaacttaaactgcaacccaatcttaaatgcctaagtagtgtttcc

ttgttagaaaacacaaagccaaagactcatatggactttggttacaccatgaaagctttg

agaagcaagaagaaggtgggttagtggtttggagtcgaatatgacttgatgtcatgtgta

tgattgagtataagaacttaaaccgcaaccggatcttaaaggcgtaagaattgtatcctt

gttagaagacacaaagccaaagactcatatggaatttggctataccatgaaagcttttag

aagcaagaagaaggttggttagtgttttggagtcgaatatgacttgttgtcatgtgtatg

attgagtataagaacttaaactgcaacctgatcttaaaagcctaagtagtgtttccttgt

taaaagacacaaagccaaagactcatatggactttggcgacacaatgaaagctttaaaaa

gcaagaagaatgttggttagtgttttggagtcgaatatgacttgatgtcatgtgtatgat

tgagtataagaacttaaaccgcaacccgatcttaaaagcctaagtagtgattccttgtta

gaagatacaaagccaaagactcatacggactttgactacaccatgaaagctttgagaagc

aagaagaaggttggttagtgttttggagtcgaatatgacttgatgtcgtgtgtatgattg

agtataagaacttaaaccgcatctggatcttaaaggcgtaagaattgtatccttgttaga

agacacaaagccaaagactcatatggacttgggctacaccatgaaagctttgagaagcaa

gaagaaggttggttagttgtttggagtcgaatatgacttaatgtcatgtgtatgattgag

tataagaacttaaaccgcaacccgatcttataagcctaagtagtgtttccttcttagaag

atacaaagccaaagattcatacggattttggctacaccatgaaagccttgagaagcaaga

tgaaggttggttagtgttttggagtcgaatatgacttgatgtcatgtgtatgattgagaa

aaacaacttaaaccgcaacccgatcttaagagcctaagtagtgtttccttgttagaagac

acaaagccaaagactcatatagactttggctacaccatgaaagctttgagaagcaagaag

aaggttggttagtgttttggagtgaatatgacttgatgtcatgtgtatgattgagtataa

gaacttaaaccgcaacccgatcttaaaagcctaagtagtgtttccttgttagaagacaca

aagccaaagactcatatggactttggttacaccatgaawgctttgagaagcaagaagaag

gttggttagtgttttggagtcaaatatgacttgttgtcatgtgtaagattcattataaca

acttaaaccgcaacctgatcttaaaagcctaagtagtgtttccgagttagaagacacaaa

gccaaagacacatatagactttggctacaccatgaaagctttcagaagcaagaagaaggt

tggttacatatttggagtcgaataggacttgatgtcatgtgtatgattgagtataacaac

ttaaaccgcaacctgatcttaaatgcctaagtagtgtttccttgttagaagacacaaagc

caaagactcatatggactttgcctacaccatgaaagctttaaaaagaaagaagaatgttg

gttagcgttttggagtcgaatatgacttgatgtcatgtgtatgattgagtataagaactt

aaaccgcaacccgatcttaacagcctaagtagtgtttccttgttagaagatacaaagcca

aagactcatacggactttgactacaccatgaaagctttgagaagcaagaagaaggttggt

tagtgttttggagtcgaatatgacttgatgtcgtgtgtatgattgagtataacaacttaa

accgcaacccgatcttaagagcctaagtagtgtttccttgttagaagacacaaagccaaa

gactcatatggactttggttacaccatgaaagctttgagaagcaagaagaaggttggtta

gtgttttggagtcgaatatgacttgatgtcatgtgtatgattgagtataagaacttaaac

tgcaacccgatcttataagcctaagtagtgtttccttgttagaagatacaaagccaaaga

ctcatacgaactttgactacaccatgaaagctttgagaagcaagaagaaggttagttagt

gttttggagtcgaatatgacttgatgtcatgtgtatgattgagtataasaacttaaaccg

caaccctatcttaaaagcctaagtagtgtttccttgttaaaagacacaaagccaaagact

catatggactttggctacaccgtgaaagctttgagaagcaacaagaaggttggttagtgt

tttagagtgaatatgacttgatgtcatgtgtatgattgagtataagaacttaaactgcaa

cccgatcttaaatgcctaagtagtgtttccttgttagaagacacaaagccaaagactcat

atggactttggttacaccatgaaagctttgagaagcaagaagaaggttggttagtgtttt

ggagtcaaatatgacttgttgtcatgtgtaagattcattataacaacttaaaccgcaacc

taatcttaaaagcctaagtagtgtttccgtgttagaagacaccaagccaaagactcatat

agactttggctacaccatgaaagctttcagatgcaagaagaaggttggttacatatttgg

agtcgaataggacttgatgtcatgtgtatgattgagtataacaacttaaacagcaacctg

atcttaaaagcctaagtagtgtttccttgttagaagacacaaagccaaagactcatatgg

actttgcctacaccatgaaagctttaaaaagaaagaagaatgttggttagcgttttggag

tcgaatatgacttgatgtcatgtgtatgattgagtataagaacttaaaccgcatcccgat

cttaaaagcctaagtagtgtttccttgttagaagatacaaagccaaagactcggactttg

actacaccatgaaagctttgagaagcaagaagaaggttggttagtgttttggagtcgaat

atgacttgatgtcgtgtgtatgattgagtataagaacttaaaccgcaactggatcttaaa

ggcgtaagaattgtatccttgttagaagacacaaagccaaagacttatatggamtttggc

tacaccatgaaagctttgagaagcaagaagaaggttggttagtgttttggagtcaaatat

gacttgttgtcatgtgtaagattcattataacaacttaaaccgcaacctaatcttaaaag

cctaagtagtgtttccgtgttagaagacaccaagccaaagactcatatagactttggcta

caccatgaaagctttcagatgcaagaagaaggttggttacatatttggagtcgaatagga

cttgatgtcatgtgtatgattgagtataacaacttaaacagcaacctgatcttaaaagcc

taagtagtgtttccttgttagaagacacaaagccaaagactcatatggactttgcctaca

ccatgaaagctttaaaaagaaagaagaatgttggttagcgttttggagtcgaatatgact

tgatgtcatgtgtatgattgagtataagaacttaaaccgcatcccgatcttaaaagccta

agtagtgtttccttgttagaagatacaaagccaaagactcggactttgactacaccatga

aagctttgagaagcaagaagaaggttggttagtgttttggagtcgaatatgacttgatgt

cgtgtgtatgattgagtataagaacttaaaccgcaactggatcttaaaggcgtaagaatt

gtatccttgttagaagacacaaagccaaagactcatatggactttggctacaccatgaaa

gctttgagaagcaagaagaaggtttgttagttgtttggcgtcgaatatgacttgatgtca

tgtgtatgattgagtataagaacttaaaccgcaacccgatcttataagcctaagtagtgt

ttccttgttagaagatacaaagccaaagattcatacggattttgactacaccatgaaata

catgagaagcaagaagaaggttggttagtgttttggagtcgaatatgacttgatgtcatg

tgtatgattgagaaaaacaacttaaaccgcaacccgatcttaagagcctaagtagtgttt

ccttgttagaagacacaaagccaaagactcatatggactttggctacaccatgaacgctt

tgagaagcaagaagaaggttggttagtgttttggagtgaatatgacttgatctcatgtgt

atgattgagtataagaattttaaccgcaacccgatcttaaaagcctaagtagtgtttcct

tgttagaagacacaaagccaaagactcatatggactttggttacaccatgaaagctttga

gaagcaagaagaaggttggttagtgttttggagtcgaatatgacttgatgtcatgtgtat

gattgagtataagaacttaaaccgcaaccggatcttaaaggcgtaagaattgaatcctta

ttagaagacacaaagccaaagactcatatggacttaggctacaccatgaaagctttgaga

agcaagaagaaggttggttagtgttttggagtcgaatatgacttgttgtcatgtgtatga

ttgagtataacaacttaaaccgcaagctgatcttaaaagcctaagtagtgtttccttgtt

agaagacacaaagccaaagactcatatggactttggctacaccatgaaagctttgagaag

caagaagaaggttggttagtgttttggagtcgaatatgacttgatgtcatgtgtatgatt

gagtataagaacttaaaccgcaacccaatcttataagcctaagtagtgtttccttgttag

aagacacaaagccaaagactcatatgggctttggctacaccatgaaagctttgagaagca

aaaagaaggtttgttagtgttttggagtcgaatatgacttgatgtcatgtgtatgattga

gtaaaagaacttaaaccgcaaccggatcttaaaggagtaagaa

[gapbp][ExpandNs](http://www.ncbi.nlm.nih.gov/nuccore/14192923?fmt_mask=65536)

ttaagtcatattcactccaaaacactaaccaa

ccttcttcttgcttctcaaagctttccagcacaattccatatgagtctttgtctttgtgt

cttctaacaaggatacaattcttactcctttaagatccggttgcggtttaagttctttta

ctcaatcatacacatgacatcaagtcatattcgactccaaaacactaacaaaccttcttt

ttgcttctcaaagctttcatggtgtagccaaagcccatatgagtctttggctttgtgtct

tctaacaaggaaacattatttaggcttttaagatcgggttacggtttaagttgttatact

caatcatacacatgacaacaagtcatattcgactccaaaacactaaccaaccttcttctt

gcttctcaaagctttcatggtgtagccaaagtccatatgagtctttggctttgtgtcttc

taacaaggcaacactactgaggcttataagatcgggttgcggtttaagttcttatactca

attatacacatcacatcaagtcatattcgactccaaaacaataacaaaccttcttcttcc

ttctcaaagctttcatggtgtagccaaagtccatatgagatttggctttgtgtcttctaa

caaggcaacactacttaggcttataagatcgggttgcggtttaagttcttatactcaatc

atacacatgacatcaagtcatattcgactccaaaacactaaccaaccttcttcttgcttc

tcaaagctttcatggtgtagccaaagtccatatgagtctttggttttgtgtcttctcaca

aggaaacactacttaggcttttaagatcgggttgctgtttaagttcttatactcaatcat

acacatgacatcaagtcatattcgactccaaaacactaaccaacattcttcttgcttctc

aaagctatgatggtgtagaggaagtccatatgagtctttgtcgttgtatcttctaacaag

gaaacactacttaggcttttaggataaagttgcggtttaagttcttatacgcaatcatac

tcatgacatcaagtcatatttgactccaaaacactaaccaagcttctccttgcttctcaa

agctttgatggtgtagccgaagtctttatgagtctttgtctttgtatcttctaacaatga

aactttactttggcttttaagatccggttgcggtttaagttcttatactcaatcatacac

atgacatcaagtcatattcgactccaaaacactaaccaagcttcttcttgcttctcaaag

ctttgatggtgtagccaaagtctgtatgagtctttgtatttgtatcttccaacaaggaaa

cactacttaggcttttaaggataaagttgtggtttaagttcttatactcaatcatacaca

tgacatcaagtcatattcgactccaaaacactaaccaagctttttcttgcttctcaaagc

tttgatggtgtagccgaagtccgtatgagtctttctctttgtatctacgaacaatgaaac

actacttagtccgtatggtgaaatctaagtgttgagttcgctcttcacaagactatattt

catccctcaaggattttttcaagttccacctcttgaaaaaatatattcattttcgtgaaa

tctaagtgttgtgtttgttattcacaagacaagatttcatccctcaaaaagttttccaag

ttccacctctcggaaaaatatattcatttcggtgaaatctaagtgttgagttcgctcttc

acaagactagatttcatcccttaaggatttttccaagttccacctcttggaaaaatatat

tcatttagctcgctcttcacaagactagattttatccttaaaggattttttcaagttcca

cctcttgaaaaatatattcattttggtaaaatctaaatgctgagtttgctattcacaaga

caagatttcatccctcaaaaagttttcgaagttccacctctcggaaaaataaattcattt

cgctaaagtctaactgttgagttggcttttcacaagattagactgcatccctcaagaatt

tttccaagctccatttttggaaaaatatattaatttcggagaaatctaagtgttgagttt

gcgcttcacaacactagatttcatccgtcaatgatttttccaagttccacctcttggaaa

atatattcacttggatgaaatctaagcgttgagtttgctcttcacaaaactagatttcat

ctctcaaggatttttccaagttccacctctcggaaaaatatattcatttaagtaaaatat

taagtgttaagttcgcacttcacaagactagaattcatccctcaagtatttttccaagtt

ccacctcttggaaaaatatattcatttcggtgaaatctaagtgttgagtttgctctttac

aagactagatttcatccctcaaggatttttccaagtttcacctcttgaaaaaatatattt

attttggtgaaatctaagtgttgagtttgctctttacaaaactagattttatctctcaag

gatttttccaagttccacctctcggaaaaatatattcatttaagtaaaatattaagtgtt

gatttcgcacttcacaagactagaattcatccctctaggatttttccaagttccacctct

tggaaaaatatattcatttcggtgaaatttaagtgttgaactcgctcttcacaagactag

attgcatccttcaaggatttttccaagttacaccttttggaaaaatatatttatttcggt

gaaatcgaagtgttgagttcgctcttcacaagaatagatttcatccctcaaggaattttc

caagttgcacttcttggaaaaatatattcatttcggtgaaatctaagtgttgagtttgcg

cttcacaagactagatttcatccgtcaaggatttttccaagtttcacctcttgaaaaaat

atatttattttggtgaaatctaagtgttgagtttgctcttcacaaaactagattttatct

ctcaaggatttttccaagttccacctctcggaaaaatatattcatttaagtaaaatatta

agtgttgatttcgcacttcacaagactagaattcatccctctaggatttttccaagttcc

acctcttggaaaaatatattcatttcggtgaaatttaagtgttgaactcgctcttcacaa

gactagattgcatcnnnnnnnnnnnnnnnnnnnnnnnnnnnnnnnnnnnnnnnnnnnnna

aaaatattttcatttcggtgaaatctaagtgttgagttcgctattcacaagactagattt

catccctcaaggatttttccaagttccacattttggaaaaatatattcatttcggtgaaa

tctaagtgttgagctcgctcttcacaagactagatttcatccttcaaggatttttccaag

ttacaccttttggaaaaatatattcattttggtgaaatggaagtgttgagttcgctcttc

acaagactagatttcatccctcaaggatttttccaagttgcaccttttggaaaaatatat

tcattttggtgaaatggaagtgttgagttcgctcttcacaagactagatttcatccctca

aggatttttccaagttgcattttttggaaaaatatattcatttcagtgaaatctaagtgt

tgagtttgctcttcacaagactagatttcatccgtcaaggatttttccaagttccacctc

ttggaaaaatatattcatttcggtgaaatctaagtgttgagttcactcttcacaagacta

gatttcatccctcacggatttttccaagttccacctcttggaaaaatatattcatttcgg

tgaaatctaaatgttgagctcgcttttcacaagactagatttcatccctcaagaattttt

ccaatttgcatttcttgaaaaaatatattcatttcggtgaaatctaattgttgagttcgc

gcttcacaagactagatttcatccctcaaggatttttccaagttgcatttcttggaaaaa

tatattcatttcggtgaaatctaagtgttgagttcgcgcttcacaaggctagatttcatt

cctcaaggatttttccaagttccacatcttggaaaaatatattcatttttggtgaaatct

aagtgttgagtttgctcttcacaaaactagattttatctctcaaggatttttccaagttc

tacctctcagaaaaatatatttatttcggtgaaatctgaatttttgatttcgcaattcac

aagaatagatttcttccctcaaggatttttccaagtttcacctcttggaaaaatatattc

atttcgatgaaatctaagtgttgagttcactcttcacaagactagatttcatccctcaag

gatttttcctagttccacctcttggaaaaatatattcattttggtgaaatctaagtgttg

agctcgctcttcacaaaactagatttcatccctcaaggatttttccaatttgcatttctt

gaaaaaatatattcatttcggtgaaatttaattgttgagttcgcgcttcacaagaataga

tttcatccctcaaggatttttccaagttgcatttcttggaaaaatatattcatttcggtg

aaatctaagttttaagttcacgcttcacaagactagatttcatctctcaaggatttttcc

gagttccaccttttggattaatatattcatttcggtgaaatataagggttgagttcgctc

ttcacaagactagatttcatcactcaaagatttatccaagtttcacctctcggaaaaaaa

tatattcattttggtgaaatttaagtgttgagcttgctcttcacaagactagatttcatc

cctcaaggatttttccaagtttcacctcttggaaaaatatattcatttcggtgaaatcta

agtgttgagttcgctattcacaagactagatttcatcactcagggatttttccaagttcc

acattttggaaaaatatattcatttcggtgaaatctaagtcttgagctcgctcttcacaa

gactagatttcatccttcaaggatttttccaagttacaccttttggaaaaatatattcat

tttggtgaaatggaagtgttgagttcgctcttcacaagactagatttcatccctcaagga

tttttccaagttgcatttcttggaaaaatatattcatttcggtgaaatctaagtgttgag

tttgcgcttcacaagactagatttcatccgtcaaggatttttccaagttccacttcttgg

aaaaatatattcatttcggtgaaatctaagtgttgagttcactcttcacaaaactagatt

tcatccctcaaggatttttccaagttccacctcttggaaaaatatattcatttcggtgaa

atctaagtgttgagctctctttttacaagactagatttcatccctcaaggatttttccaa

tttgcatttctagaaaaaatatatttattttgttgaaatctaagtgttgagttcgcgctt

cacaagactagatttcatccctcaaggatttttccaagttgcatttcttggaaaaatata

ttcatttcggtgaattctaagtgttgagtttgcgcttcacaaggctagatttcatctctc

aaggatttttccaagttacacctcttggaaaaatatattcatttttggtgaaatctaagt

gttgagtttgctcttcacaaaactagattttatctctcaaggatttttccatgttctacc

tctcagaaaaataaatttatttcggtgaaatctgaagtgttgagttcgcaattcacaaga

atagatttcatccctcaaggatttttccaagtttcacctcttggaaaaatatattcattt

cggtgaaatctaagtgttgagttcgctcttcacaagactagatttcatcactcaaggatt

tttccaagttcctccttttggaaaaatatattcatttcggttaaatctaagtgttgagct

cgctcttcacaagactagatttcatccttcaaggatttttccaagttacaccttttagaa

aaatatattcatttcggtgaaatggaagtgttgaattcgctcttcataaaactagatttc

atccctcaaggatttttccaagttgcacttcttggaaaaatatattcatttcggtgaaat

ctaagtgttgagtttgcgcttcacaagactagatttcatccgtcaaggatttttccaagt

tccacctcttggaaaaatatattcatttcggtgaaatctaagtgttgagttcactcttca

caagactagatttcatccctcacggatttttccaagttccacctcttggaaaaatatatt

catttcggtgaaatctaaatgttgagctcgcttttcacaagactagatttcatccctcaa

gaatttttccaatttgcatttcttgaaaaaatatattcatttcggtgaaatctaattgtt

gagttcgcgcttcacaagactagatttcatccctcaaggatttttccaagttgcatttct

tggaaaaatatattcatttcggtgaaatctaagtgttgagttcgcgcttcacaaggctag

atttcattcctcaaggatttttccaagttccacatcttggaaaaatatattcatttttgg

tgaaatctaagtgttgagtttgctcttcacagaactagattgtatctctcaaggattttt

ccaaggtctacctctcagaaaaatatatttatttc

[gapbp][ExpandNs](http://www.ncbi.nlm.nih.gov/nuccore/14192923?fmt_mask=65536)

atatattcatttcggtgaaatctaagtgttgagtttgctc

tttacaagactagatttcatccctcaaggatttttccaagtttcacctcttgaaaaaata

tatkymttttggtgaaatttaaatgttgagctcgctcttcacaagactagatttcatccc

tcaaggatttttcaaagttccacctctcggaaaaatatatttatttcgatgaaatctaag

tgtttaacttgatcatcacaagactagatttcacccctcaaagagttttccaagttccac

ctcttgaaaaaatataatcatttcggtgaaatccatgtgttgagttcgctcttcacaaga

cttgatttcatccctcaaggatttttccaagttccacctttttgaaaaatatattcattt

cagcaaaatctaagtgttgagttcgctcttcacaagactagatttcatccatcaaagatt

tttgcaagttccacctctttggaaaaatatattcattttggttaaatctacgtgttaaat

ttactattcacaaaactagatttcatccatcaaaaagttttccaagttccacctctcgga

aaaatatatccatttcggtaaaatctaagtgttgagttccctcttcacaaaactatattt

catccctcaagaatttttccaagtttcacctcttgaaaaaatatattcatttcagcgaaa

tctaagtgttgagctcgctcttcacaagactagatttaatccctcaaagatttttccaag

ttccacctcttggaaaaatatattcatttcggtgaaatctaattgttgagttagctcttc

acaaaactagatttcatccctcaaggatttttccaagttccacctctccgaaaaatatat

tcattttggagaaatctaagtgttgaactcgcttatcacaagactagatttcacccttca

aggatttttcctagttccacctcttaaaaaaatataatcatttcggtgaaatccaagtgt

tgagttcgctcttcacaagattagatttcatccctcaaggatttttcaaagttccacctc

ttgaaaaaatgtattcattttggtgaaatgtaagtgttgagttcgctcttcagaagacta

gatttcatccctcaaggatttttccaagttccacctcttggaaaaatatattcatttcgg

tgaaatctaagtgttgagctcgctcttcacaagactagattgcattcttcaaggattttt

ccaagttacaccttttggaaaaatatattcatttcggtgaaatccaagtgttgaatwygc

tcttcataagactagatttcattcctcaaggatttttccaagttgcatttcttggaaaaa

tatattcatttcrgtgaaatctaaatgttgagttcgcgtttcacaagactagatttcatc

cctcaaggatttttccaagttccacctcttggaaaaatatattcattttggtgaaatcta

agtgttgagtttgctattcacaaaackagatttcatccctcwwaaagttttccaagttcc

acctctcggaaaaatatattcatttcggtgaaatattaagtgttgagttcgcaattcaca

agactagatttgacccctcaaagatttttcatagttccacctctcggaaaaaaatatatt

catttcggtgaaatctaagtgttgagcttgctcttcacaagactagatttcatccctcaa

aagtttttccaagttccacattttggaaaattatattcaattcggtgaaactaagtgttg

agctcgctgttcacaaaaatagatttcatccttcaaggacttttccaagttccaaatctt

ttaaaaatatatttcattttggtgaaatctaagtgttgagttcgctcttcgcaagactag

atttcaaccctcaaggattttttcaagttccacctctcggaaaaatatatctatttcggt

gaaatctaagtgttgaattcgctattcaygagactagatttcatccctcaaagatttttc

caacttccacctcttgaaaaaatatattccttttggtgaaatttaaatgttgagctcgct

cttcacaagactagatttcatccctcaaggatttttcaaagttccacctctcggaaaaat

atatttatttcgatgaaatctaagtgtttaacttgatcatcacaagactagatttcaccc

ctcaaagagttttccaagttccacctcttgaaaaaatataatcatttcggtgaaatccat

gtgttgagttcgctcttcacaagacttgatttcatccctcaaggatttttccaagttcca

cctttttgaaaaatatattcatttcagcaaaatctaagtgttgagttcgctcttcacaag

actagatttcatccatcaaggatttttgcaagttccacctctcgaaaaaaatataatcat

tacgatgaaatataagtgttgagctcgctgttcacaagacaagattacatccgtcaagaa

tttttccgagtcccacctcttggaaaaatatattcattttggtgaaatctatgtgttgag

ttcgctcttcacaagactagatttcatccctcaaggattttttcaagttacacctcttgg

aaaaatatattcattttcgtgaaatctaaatgttgtgtttgctattcacaagacaagatt

tcatccgtcaagaattttttcgagtcccacctcttggaaaaatatattcattttggtgaa

atctaagtgttgagttcgctcatcacaagactagatttcatccctcaaggattttttcaa

gttacacctcttggaaaaatatattcattttcgtgaaatctaaatgttgtgtttgctatt

cacaagacaagatttcatccctcaaaaagtttccaagttccacatcttggaaaaatattt

tatttcggtgaaatctaagtgttaagtgataataggtttttagtcaattatccaaaaaca

ccaatcatgtcgttgtagtattttaggttgtcaatccaaataggtgtgatgcaaacagat

gagatgtgatcagaagtcactaagtcaagccaagcaataacagttttggggtttctaaca

gtcctaagcgaacatgcagaaaacagaaacaataacagaattactaaaatcactcaacca

caacaggctgatgccagaagtaagatatggtcgagtaacagacaggaaatatgacaactg

tactcgactgcatagtctgttgccagacactgagtgtggtcgagtaagttgatcgagtaa

ttgcaaaaacgaagagacatgcaatgagaacaattaagcgataacgataaaacagagaaa

tcaaatagacgagaaattcctaaggatggggtaatcgaataaagtggtatcctagcctat

ccgaatggttaacaagcgcaatcaagctatccctagacaacatgttcaacaatagatcaa

ttcactcccgtgatagaaatcctcaagcaaagctagcccagactaattcccattaacgga

atctaactatgcaggcaataagaacagactgataaagtcaataaacgctctaaacggcca

atcccttgggatagagacgaatatctcaggaactagtggatcaagcattccatcgaacac

cttctggttgcgggaatgcttgggatcgaattccagttgatcagaaagaaataagcagta

aacacaactagtcctgaagggattcaatcaattctaaaacttaaccattctatagactac

ggattccacaactactctatcccatccttaagaattctaagtcactactcagacaatatg

ctcaaagacataaacgcagataaatgataatattggataagtaaagagataaagagtaga

gaaacaagataacaaatgaaatcaaatgcggaatctgaataacttggaaaaatctcgaat

tacaggttgtagaagcaaaagcggcgcagccagagtaaaacagaaaataaaactgaagca

aaaacccaatgtcgactagatgtctcaggacagaacccaaagacatctatttatgcaaaa

cgaaataaaatgataggagacaggccaataacctcatcgcccgacccatagagtgataat

gggccgagatgggcttcgtgatccgatggaggtccaggccgcttccaaacacttcgtctt

ctcctcttctcttccttgtgctccgcttgtctggttgagtgcggatttgagtagactaag

aagatgtgatcgagtgcccggtcgagtagtggtgttggtggtgagtgatgatactcgacc

cactggtcgagtaacgtggtagagtgaacggccgagtgtggtcaggtggtttggcgaagt

gatactcgaccagggggtcgagtggcgtcatcgagtggcctgagctgaggttactcgatc

tgggggtccagtagatgggaagaatggctccaaagtcactcgaccagggggtcgagtatg

gcgctggatgcacacgacatggtggtcgagtatatcttctggttctggctcgtttcttct

aatttgctcgcaaacagctccaaatcacctgaaaacaactcagaaatgcaatatgtatgc

aaagctatcctaatcatgcaaagtatgcaagaatgatgagaaatgatataaaacagtgat

gaatgatacaaaacttgactcgaaatgatgatgaatggacactgaaaacatgcaaattat

agacatatcaactccccaaaacttagtctttgcttgccctcaagcaaataagaagacata

gttgaaggagaggtttgaaggtggggactcagaaccaaagcatcaggtagagcaaataaa

atcaatgtccaagttggtagttctaagatgcgatatgattaaattttactcaaaaacgtt

agccatgccttgttatcaatcaatctgtctcttatactcgacctatacatgctttcaaat

ctaccaatccctttaacattcattagttctagaacgtgaatcaagcaatgcatcatcaat

gaactcatttggctaaggtgaaggtcaagagacaaagatggtcccttacagaataggctt

aagtaacagggaatctctcaagaatgattgagctttagtagaatgctaaaggtaagtccc

aagctatgcatacagagataagatcccatctacccatagtataacaatgaagctataatg

gttatcccaactctagtcccaatacaagaacttaactctaccctttgcactcatgaatct

ttcaaactctttttcatatcattcttttcttttctcttaactctaggagaagctttctca

acactttgatacatctagcggatttggatttctttaacaactaaggttcctgtttttaat

tttttaaacaaaaggcttttaacccttcttctttaactaacaagaacctcattcttttct

ttaacattcccaaaacctttactagattttttttctaacttttttagcttacttccccta

gaattctttccctttccttatcaacaatgaattcacccttttcttttaaacacatcccaa

cccatgatctaagcgcccacctagtcctatccagtccgaaaaacgcgaactagaactaca

tgagacactatctctgtcgttacgaacctaacactttctaccaagctcaatcgaaataag

acctcacaaacactcacaagtgatatagggcttgaaagaaagtttaggtttgggtttggg

ttaactgctataataaggagagtcagctacttggattaacaagagttgttaaaatgagta

agaaaatccaagtattttagggtatccatgagttcaaattcgatgccaagacatgataat

tctaaatcagattcaagttcaaattgatagggaatggtatcagatcctaacagtcgtgtg

ctcgagtagagcaatctaggcatggtgtagttttacttcaatttcattgactatgcatca

gacaactaacaacgagggcactgagtttatctggtgaaccaaaatgcttacaaggctatc

tcatcattgtcccctatgcatatgcaatgatcctaaatgaaacactctagactcgatcct

aaatgcaatgcaactatatgaacactctgtttttgtgaaatcattttctgaaatttttca

attttttttttggttttttctatgccttagatgaatgcagactcaagattatatacaacg

caacacacacttcctgagccctcccccaaacttaaatcacacagtccactgtgtgaccaa

atttggaagagagtactcaggacaaaacacatcacagaaacaaaataaaaacaggagatg

gtatattacaatggtggagtgaggtgcttacctcagtggaagaaggagcggagttggtca

tcaacagccgcctgtgaatgctcccaggccatcgaagaatctctctgttgtgtctcagct

tccccctgggggtactcaacctctgctccttagtcgcggccagcaccgctctgatgatac

tcgatctctctttttctgctgcggccagcaccgcgatcgcgtaagctacgggactggaga

agtcgtgtactcctgctgccagaccgagcgagtgcagccttcttctttctcccgagctcc

cgaggctcgaatgatgaatgccacgcagggacttgtgagactactcggtcactaggctca

ggcatgtggtgacttggctcaggcctgtggcgagttggctctggcgcgtcatatctcctc

gagggcatgtcctgaggaggctctccctgcggaatcgctgtggtagaagaggagcagctt

agcttgtctgtcagaaacttgatggccttgaagcacttgatgagtagcctatcttgtttc

ttgcaccacctctgcaacttactgttgtttctgtgagcttcgctcaagctcttgctctcc

ctcgctggaggtacatgctcactgaaatgatacatgcgcgtatcgtactcctcctcccta

tcctcatctgtctcagcaatctcagcttgggtagcttctttgtagggacgttgtcatcag

tcggtggagcgctctcaaagtagaggtaatcacgcgcaggcttgaagtcaatgttttcgc

cctgaagtatggttgtggcctcggtgcagggaagcagaatgttggctgttcgggtcaagg

agtgctcaaatttgtagcgatagaaatcgccaaccatgtcgtgctccagaaactcacaac

gacgcaagtgatctaaatccatcatcctcggatcaaaccctggagacgtgaaaggtacac

cacatgtaatcagaattggtgtcacaacaccacctacacaaagggctcctcgcgccctct

tcttcccgttggtgtgcgcccacttcctgtatccacacaggtggatcaacagaagcataa

ctggtggtgcatcattgaggtcgcccttcaggaccttcttcccctttgttctacggagaa

tgcccttgagtgcagaattaatcatctccatgtctgtgttagacacggtacctgtagatt

ccctagagtacagaacattcgctatagagcgttgatagtagcagatcacagggcttcgaa

tctggttgctcttggacctcgcagagttgagcgccatatcgttcccaatagttaaccaca

aatccttcaactcttccctctctaacttgggtttagttccctttccactgggtaagccaa

acaatccttccaagctcctgatagatagctggtaacactgctcgctcacggaaaaagtca

agaaccccaacccttcactctccagctcatctgctgtaagtccctgatacatctccactt

gcagagtggagagaaactctcttatttccttcttgtaagcgacgtacgggtaagacatca

gtgtctccagatgacacttctcgaataggtgttgtacgtcctccggtagccccagctggg

ctaaagtctgataataggggtacttcgttccccagaagtcgttcagctcgaaaagtctga

tgtactcctccactgggacttcattaggcctgttcaacagcttcgtctctctatgccatg

actcaggctcatactcgacgtcaatatcatcgtcgatcaactcatatctgctggtcattg

ctctctttcctctagcaatctcagctctcctctcattgcgtcaagttactgagcgttggg

tctccgctctgaagctctcataagcttgtcgctctctctctggtccagttgaccaagatt

cagcttcatcgatgttgtaatccgcatccatggaggattcgccactgtagttactcatat

tgatttcctgaaaaagaatgtaactcaaagccaaacgtcaatatgttagtccaaataaat

gcaaacactaaaccggtcaagtacacggtcgagtattacggtcgagtacagtgaaattat

ggtttcgagtttagcggtagagtgaagacgaatgagagacggtaaatgcgtggtcgagtg

aacggcgaatggagagaagacagtggcggtttgaggagcggtcgagtgaagagataaacg

gaggcgcaatgaatggcgaggtcgagtgaacggtggatagttgagagatggtgaagccgg

tccggcggtcgagtaaaagagagggaacggagatgaggtcgagtagcggttgagtatgga

gaggtgagaaaaagaaatcggagtagaggttgaggtgctcgtgcggtcgagtgaagagga

ggcgagagcggtgagagatgatgaaaccggtcccgtcgttgagtggaagagagggagaag

gcagtggcgtgagcgatgaaagctgaattttggtcgagtgagaaagttgacgaagtggtg

agggatggtgaagttgggacggcgaggtcgagtgaagaggaggcgacgttgagacggtga

aggtccggcggtagagtaggcggagaatagagaggagaggcgtttcgatcgagtgaaaga

gaggcggtgagagagatgtcgagtgatgtttggtcgagtgagagacgtgacggagaatgt

gtctgcgaggtcgagtgaatggagaggaaaagagatgatgtcggtgggaaaggatagggt

gagatcgagtggtggagaagtagtcttgggatttcgttatctctaaaaacgcatgtggga

acaaaggtgagaggtggtgggccacactctgtctgtttgtcctgaagcactgttctctgt

tgactaggaatgtcttagaaccttctgtaatcctctgtaaacttgataaactctctgtaa

gtctttgtagcttctgttggtcgagtataaatatgatttcaaaattttgggtttttaaaa

tcatcactaatactcgaccaaactactcgatctcatggtctctgattgcaacagaacttt

tttcaaattcttgaatttctttacactgacatgctcgatcccctgttgctctgtagagta

aaattttaaaatttttactctacctccacgctgtttctattttcctgagaatcacaaaca

cagccaaaaacaaatcaaaaataacaagaaaataaaaattttatttatagaaacagtctt

gggaattcctcgcaagtgagcttgtttaaagtctctagcttgactcctcacacttattaa

gaagaaaatgggtgatagagaggatctgcatagtcctctctaatctcatcatcatattca

gcatcctctcttgagagatgcgtagcagaatacttaattcttcttttctcgaaataggcc

tctttctcttctggtggataatctatctcctcagaccatcttgaagtaaacttttttctc

gggatagtatcaatcccgcattttgattgagctttctcttgcatctgatccagtttactc

ttaagttcctcaactgtctgagctagcttctctatggtttcttcttgctcatcagatttc

tttttgagatctcgcaagtcaggtgtagaggttgtgcttggtctggtgattgaatccgaa

ggttgaggctgagccctgatcttttcttctacagctgtcctttgcgaagtttcgttgatg

tcaaactgcagcttgatgtgcttcccaaggttgagacttattctcttgtctttgacatct

atcatcgctcccacggaggctaagaatggtcttcctaggattagaggatccttaggttct

ggtttcatctcaagcacaacgaaatctgtaggtacttccactccattaatcattactggc

agatcttgtagcaggccaaagggtttccttgaagacctatcaggaaggatcaaagtcagg

tcgcaaggcttgtactgagtgaattccagcttccttgccatgaagagtggcattaagctt

actgaagctcccaaatcacatagacaattgctgaaaaccaattgaccaatggaacatggt

agagtgaaagttccaggatcttccagtttttcttgaacactcctcttaacagtggcccta

gatggattagcctcacaatcatcttctgaatcttgataaatcttgatcctttccagaatt

gaatttctcacatctttgtgaggatccgggatcaggttgagaacttctattagaggcatc

accgcttccatctcatcaagctgcttctctgcaagagaattgtatctttctatccatgct

ttcctgaatttccatggaagagcacgtgaaggtaatggtgctggcttgaatcgttttacc

atcctctcaatgatggctgctttctcgtctaggttggactgagtttgaaaacgggatcca

gctaaatggtcgagtccaacaactgaaatttcaatctgagtagaagccttcccgtcctga

acatcactgtcctcggtgattgatgtggagacatgtcgaataggcaactctcgatcatga

cagatggtgatagcgtgagcggtggcgtaatccttcgggttctgtatgaactttcctgga

agtcctcttactttgggagcagaggtcgacgcagtttgtccttccatgtatctgaccttt

gaggtgagtgcctcaagtttaatgttcagatcgttgaaagtgcaatcgaccttgttatgg

atttctgccatcttgttggcgagatccattgcccctgctgcttgtccctggagtatttgc

tgtaacatgttcttcaagtttgaatgtggagttgttgaagctggttgttgttgctgctgt

gtgaacccaggtggtggaagttgcggcggatagttgccctgatactgctgcttaggaaca

tacccctgaccttggttgtatggaacaaagggcttgggtttattcagctgctgagagggg

tagacttggtcctgtgggtttgcaacatttgtacttctgtaagacagattgggatggttc

tgcttgaagttgttgaaatctttgttgtaacctccttggttctgcacatagctgacctct

tctgactacagagtatccccatcctggacttggaacgtctcatcatcacccagaaaatga

atgtgcttctgttgcacaaggagtagcttgtccagtttgtcatttatagctttcatttcc

ctgtggtgcttctcatcagaatcagagctgtggcggatgcttctatcgtaatcttcattg

tagttgccatccgactgtgccaagttctctaccagctcccatccttcttcaacgtctttg

ttgaggaagttcccgttagaagcggtatcaagaagcatcctgatcttgggaaggacacct

ctgtagagagtgctgagaagtgaagctttggagaaaccatgatgaggacattgcgtctgg

taacctttgaagcgctcccatgcttcacagaaagtctcattatttgtttgtgtgaaaccg

gagatatcattccttagtctcgcagtccaagagtttgagaaaatatattcatttcggtga

aatcgaagtgttgagttcgctcttcacaagactggatttcatccctcaaggatttttcca

agttgcccttcttggaaaaatatattcatttcggtgaaatctaagtattgagttcgcact

tcacaagactagatttcatccgtcaatgatttttccaagttccacctctcggaaaaaaat

ataatcatttcggtgaaatctaagtgttgagttcgctcttcacaagactagatttcatcc

ctcaagaatttttccaagtttcacctcttggaaaatatattcatttcggtgaaatctaag

tgttgcgttcgctcttcacaagactagatttcatccctcaaagatttttccaagtttcac

ctctttaaaaaatatattcatttcagcgaaatctaagtgttgagttcgctcttcagaaaa

ctagatttcatccctcaaggatattttcaagttccacctctcgaaaaaataaattcattt

agctcgctcttcacatgactagatttcatccttcaaggattttttcaagttccacctctt

ggaaaaatatattcatttttggttaaatctaaatgttgagtttgctattcacaagacaag

atttcatccctcaaaaagtttttcaagttccacctcttggaaaaatatattcatttcggt

gaaatctaagtgttgagttggcttttcacaagactagattgcatccctcaaagatttttc

caagttccatttcttgaaaaaatatattaattttggaaaaatctaagtgttgagtttgcg

cttcacaagactagatttcatccgtcaaggatttttccaagttccacctcttggaaaaat

atattcattttgatgaaatttaagcgttgtgttcgctcttcaccagaccagatttcatcc

ctcaaggatttttccaagttccacctcttggaaaaatatattcattttgttgaaatctaa

gtgttgagtttgctcttcacaaaactaggtttcatctctcaaggatttttccaagttcca

cctctcggaaaaatatatccatttcggtaaaatttaagtgttgagttcgctcttcacaag

actatatttcatccctcaagaatttttccaagtttcacctcttgaaaaaatattatcatt

tcagcgaaatttaagtgttgagctcgctcttcacaagactagatttcatcgctcaaagat

ttttccaagttccacctcttggaaaaatatattcatttcggtgaaatctaaatgttgagt

tagctcttcacaaaactagatttgatccgtcaaggatttatccaagttccacctctcgga

aaaatatattcatttcggtgaaatctaagtgttgaactcgcttatcacaagactagattt

cacccctcaaggatttttcctagttccacctcttagataaatataatcatttcggtgaaa

tccaagtgttgagttcgcttttcacaagattagatttcatccctcaaggatttttccaag

ttccacctcttgaaaaaatgtattcatttttgtgaaatgttagtgttgagttcgctattc

acaagactagatttcatccctcaaagatttttccaagttccacctcttggaaaaatatat

tcatttcggtgaaatttaaatgttgaatttgctcttcacaagactagatttcatccctca

agtatttttcaaagttccacctctcggaaaaatatatttatttcggtgaaatataagtgt

tgaacttgctcatcacaagactaaatttcacccctcaaggatttttccaagttcaacctc

ttgaaaaaatatatgcatttcggtgaaatcccagtgttgagttcgctcttctcaagagta

gatttcatccctcaagtatttttccaagttccacctttttgaaaaatgtattcattttgg

tgaaatctaagtgttgagctctttcttcacaagactagatttcatccctcaaggattttt

ccaagttccacctctcggaaaaaaatatattcattacggtgaaagataagtgttgagctc

gttgtttacaagacaagatttcatccctcaaaacattttctgagtcccacctcttggaaa

aatatattcattttggagaaatctaagtgttgagttcgctcttcacaagactagatttca

tccctcaacgattttttcaagttctacctcttggaaaaatatattcatttttgtgaaatt

aagtgttgagtttgctcttcacaagacaagatttcatccttcaaggattttttcgagttc

cacctttttgaaaaatatatttatttcggtgaactctaaatgtgaagctcgctcttcaca

ggactaaatttcatctctcaaggatttttccaagttccacctctttgaaaaatataatca

tttcggtgaaatccaagtgttgagttcgctcttcacaagattagatttcattcctcaagg

atttttccaagttccacctcttggaaaaatgtattcatttcagtgaaatctaagtgttga

gttcatacttcgcaagactagattatatccctcaaggatatttccaagtttcacctcttg

gaaaaatatattcatttagctcgctcttcacaagactagatttcatccttcaaggatttt

ttcaaattccacctcttggaaaaatatattcattttggtgaaatctaagtgttgagtttg

ctattcaaaagacaagatttcatccctcaaaaagttttccaagttccaccgctcggaaaa

atatattcattttggtgaaatctaagtgttgagttcgctcttcacaagactagatttcat

ccctcaaggatttttccaagtttcacctcttgaaaaaatgtattcattttagcgaaatct

aagtgttgagttcgctcttcacaagactatatttcatccctcaaagatttttccaagttc

cacctcttggaaaaatatattcatttcggtgaaatctaagtgttgagttcgcgcttcaca

agactagatttcatccgtcaaggatttttccaagttccacttcttgaaaattatattcat

tttggtgaaatctaagtgttgaggttgctcttcacattgctagattttcatccctcaaag

atgcttttgatgtccgaccaccggaagaatgtgctcatttcgatggaatttatgtatcga

tttaactcgtcatggactcaaattccagttaaagcggtggcgattcactttttctcggaa

gcaagaaactcgtaaaagcaaacaaaaaaaaaaagaggccatcaacgtctcatgatgttc

cgagatttcccattagctacacaaacttcaccaaagaactcctggacctcaacaagacta

cagctcgtggtggcgaaacaacaaccttgtcacctccaaagtaggagcacaagtctaggt

agaagcaaggttgagtctactaagatcaactcaatcgatcaagccatgacacaatgatac

agagaagttttcatggcgtttcttcggaaatcattagcgaatgacttcaacaattacagc

catgacaggacaagatcatattggctcaaaacatttggatcagatcatgtatgaagcatc

aaagataagttcaactatggtgtacacgtttactccttaacaggaatgtctccatcttaa

ggtacacaaaatgttatgacgaaaagcatagatgtctaagaccaaacaggtgggggcatc

ttccaaataatataaagcaaagtcaagcctctttggaggagaattatacagcattatcac

ttaacgtgattgacaaatcattgctacaagtttggagtccatatttatatcatcactcaa

aattcaaggagtcaaaatgcgacatttgtcaacaagaagagattcaagtttgaaaaagac

aacttcgatgctataccacaaccttccaatgttattcaccttcatggagatgtgaatttc

aattggtggaatctaagtgttgagtttgctcttcacgaaattagattccatcgagaagag

atttctccaagttctaactcttggaaaaatgtgttcattcgatggaatttaagagttttt

tccatagtttacctgctttattccgagcaatcaatcggataaaagtggaagacgagaaga

ttagcttcacttcgcaagaaaaagaaagccgactatgccaattgttactgtcccttccag

gaaaccaaagttaaagggcaacaaagagatcaaaccacttggattaaacccatttaacaa

taatgtggagacaccatctgtaagacccctataccgctatataaatataaacgaaatagg

agcgtcacgttagacgcccgcagtcggccaatccaggtttccaaaatgaattcgttcgcc

tgagtcgtccagccgcaagagtctagcctcccgcccgtgacctaaggcttttcaacccta

tcgcggccgtgagttgtgatagtacgaacaagactaatatcagtcagtataaccatataa

attttagaaatccgagtcttatatatatatatatatcatacgagaaatggttaccaaaac

gtatatagtttccggatcctctttgaaaaccatacataatatacttagtaagaatttaca

aaaaaacatacataatatacttagtaaaacctacattagttcgtcctaagtcatcagcac

cctctacaaggttggtcaactaagatttcctgaaaaagataagtaacgtcataagtaatc

tagtattacttagtgaaccccggatctagacaatgttatcctaatacccttccttacccc

ctaagcagcaacaacacgtaacaataatactacctaacaagcaagacataaacaaagata

acacgcaacatacaacacaagcaaaatataatgcaccatgcagaatgcatgtagactcga

atttccttatattcaacggataactaatgcaagcatgcttctatcaacctaggtaaggct

ttgtacgaatccatgatctcccaaggacatgactccatactcttagacacaaagtctata

agagacatagcattcctttatactacatcgtcgtgtagcgcccggtcggtagcaattcag

cctcgagccttcgtatgccataacggacaacatacggacgggatcctcatggttatgcta

atgctagtcatctaattaaagactaactaacatgacaacagactcacatactatcatgca

ttcatcctatatgcaaaactctactaatcaaacaactaatcacacttaattattactaat

agcggttagtcccctaatcatggtttaactaaccctaatcatccttaggaaatactaatt

gtaattaatgatcactattcctaattaacaattaataaaccaaattaactatcctaatca

aggattaagcgaataattaagctacgtaatcataattaacctcctaagctatattaagga

aattaatgaagaaactatcctattcacgtgctacttcacgtaactcaagactcacttgga

tttctgagcggtttggcttccgaatgttttctcccgtgaacttagctcttctaggcgatt

tttaggtctaaggaatcaaatgggcggagggggtatttatatggtccaaggtcggtgatt

aggtcggccaaggcaagcaggaggtgtcctggattaatcaaaacgtgaaaaatgtcgacc

aacggccgctccaagacactaatcggcctccgattggttccttccatttttcggataaac

atcatccaaaaatggataagcacgaccctaaaaagataaggatgaccaaaatatggataa

gcttgagcctttacggataaacatgaggcacggttcaatcctggccgtccatttcttcat

tccggggaaacctacgatctgtcaccagaccgcaagaacattaatacggcacattccgtt

ccaatgtctcttgagggtccatgacgcaaattaaggtttccttaggactataacacgttc

catgccataattaaccgagtaagccagctatcgtcacttatccgcattctccgatcaatc

tcgcgaccattcacgacccatacacatatctcactgtccaaatggattcccctcgacctt

ctaatgatttgtcgcattgtagctaaacttgatccgtctcatggtgagtcattcctttac

ccatggacttgatccaattcaatgcttcttacccatgacttgccacactaaatggtcgaa

ctgatcttgcatttttttggcttgatctcgcattctactcgatagactgtacgtccggcc

acatagtcaaggtaaggggcattacaccatctcttggaaaagagaagatcgagacaacca

tctctgacaacatggttgcgatgaaaaggcatacgacaacatccatagagtaaataatta

tactacaaaagactgtaccgatatcaagaggcatcttgctgagctacggacaaagaggtg

acctcgcaaaacctcgacgtcggagagttcgtgaaaatttaccacgaagaaaaggcggaa

gtcaaaagttcatcactagctaagaaaatacctaaaacatctaatcttttggtaataaga

agaagattgaagtaatcatgaacgggtctaagttttgtagagattcgaccactacaatca

aaaagtttaggggaatgttctattaaaggcgagcacaagcgacaaactcgaactcgctag

agcaatcacaacattcaaggaagaagaaattcgtcaccttgcagaacctgatgacgttga

ttttgtcctcaccttagacgtggctgacgtcggggtttcaaagattgtgattgatgttgg

aagtttgatagatctactattcttgagtacgcttggagagaatggaaagtagccaggctg

acatagttggagccccatggatttatcggatcaaggttatcccatcaatctattgtcaat

acgttaaatatttaatcccgaatggtgtgaaaattttacaaggagatcaagaatcttcaa

aatcttgttatttaacgactcatcggatggttatccaattaacaaatctcgtgaagaaag

aagtgattttaggaatatcaagcgggcaagtagtggatgacgtcataatcgaagtggcca

ttgatgagaaagatcacgaacgaagaatttggaagaaagtagcatctagttacaaccaaa

taacgatgaatgatataagtcaaattaatcctaagaccagttcacaaagttctcggatca

cgcagtagagtaatggctaatcgaattaagctaagtaaaacagtaaagaaaataaaagaa

acaaaaagtaagcaataccaatcgattgtgagttgtgaaaacaagataataaaggcgtca

ggcttagagtattctcacgaaatagatgatagtagatctagaaatagctaggttattatc

aaaccgttctcgaactcaaactatgattttagagtaactggtggtgttccgcgaaactct

ctatacttagagctaagattaccggagaagccgagaaatcctatactaaagcatgcatta

ttatcaattttgattagttcacctagtatctaaacaagagcccttctatgagcctacctg

ttctttcttaaatgcctaggctcatctatgataggtcaaataacaagtacttatggtggc

atacctattatctaatatcaagttctagttagctactctagaactagcattaagaacaat

caagatgaagaatcctacaaataacgtagcaagggggctatctactaaatactctaaatc

cctaacgagaaaccctaaacctaacaagaagattactcaaacatgatcatagaagaacaa

aacatgattgaataagaaataattgaataaaacaagaggagaataaggtttagaaggatc

ttctctaaagggagtctcaacagggtttgctctcaaagtacagaatagaaaaagggaata

gcctcccaaagcttacggtctaaacattgacctaaaaactataaatatgtcttaagaacg

tgttgggccttcattaaacataggagaaagttctgggccgaattcggaaatcttcaaaac

atcaataagttacgtctttcaattgccgtcaggtattggtatcgctcgaaaccaggggtg

gtgtcgttcaataccagggatggtgtcgttcgacacctacgtgcattttcgtctccagat

tgcttctacagcttaagttgtatgttttgggccagaatgccccataatctcgaaatgcat

cccaaacatgtaaagacctgaaaaagactaaaaaagactctagaaataacaattagactc

taaaacctatacctaaaacatagttaaaatggtaaaaatagggatatatcaactccccca

gacttggcttttgcttaccctcaagcaaagcacaaaagtcgaacccatggaagaggtttg

aaaacacaggaactcccaatattctctaacctattgccatgatcattctaactaaatcca

tatggctagcaagtctaatcaaatcctaaccaacttgtacttctccgcaagcttacacaa

cattatagattccatacctaagttccacaactatatctatcaggctttcatcgctgagtc

tcatcaaacaagttaacattcaagatccttctagactcattcctgtgctataccgtgata

aatcgaccggtctcttatataatattttttctttggtgttctttctctcccaaacttatt

tttttagagagtaaacaaaggggaactcgcatactggatcgacacccttgactttttcca

acctttgtaattgatcattccaaataatgtataataataggatcataagaaacatttcta

cccctatacactatctgaaaatcctatcaatctctcattcatcattcattttttttttct

gggcagaggaacacattctttttatcacattggtctcaatatcaggtatgaaaaggagtc

aagatctatgaatactaaccgtttgaaaaggtaaaaggaagagcagtgagattagctcca

gccttggtggtggtgttggaaactaggatatcctcaagtctattggtttgccatctagat

tttccaatagtcggattgagtcaaacctgcagcattaatcaaccaggcaataatctaaag

aatatagatcataattcctaacaaaattttgactcaataaaacgacttaaaaagaaaaat

aagaataagaataagactcaaaaagaaaaacgtagttagtaactgcctcccccacactta

aacgtcattgtccccaatggcattcgaattcagagtaagcgagaaaaagaaaaaaaaaac

gtaaaagaaaagaacaagaaaaagaaaaccatactagtgggttgcctcgcactaagcgct

tgtttagagtcaatagcttgactttggtggagtttagggatcaggtggatcaaatggagg

aagtaaatactgcctagtagaaccccgcatcaaccaaggtggcgtattaacagtcggtgt

gtgcggtctaccaaggaagtgaggaatgtcagctgggcaggacagaggtatgggtggtgg

actccttgtacctgcacaatcatcaagagaaagaacaagtgctcgacgagaaactcgtgg

cttgcttaaccgtaaaacagttttttgaaattgtaaactcttgttgatgaaatcaggagg

tttaggaggaggagatgagtacctttgccgtttacgaggtagatgaaggagtagaggtga

tgtgggtgacgtggcaacatgtcctcaaatttcagcagtgatctgaacttgcaaagcagc

ttcattatacaattctggtttagcctgtttgggatgtcgatcgacacaggtgatggtgtc

gatcaacaccatctctacagattgattctcagcaacgaatctgaaattggcctcatgtgg

acgcttgctgttactgagtacctgttatgacacaaccattatctttgtcttgtcatcttg

cttctctggagcatcatctcgtatcatatcactttgaagagagggtgacaacttctctat

atcaactggaggcaactgaagtagaatctcatccatgatacccatgcaatgcatctcagg

aatctccttcttggactgtcgaggtagtagacaaggaacttgtggtgtgtagacttgtat

agtaacaggtggagatgcaaggcatattggggcagtttaagtcttgtgtcgatcgccact

aggtgagtgtcgatcgacactactgtgtggcatcgatcctgctctgtcaaacgtcagcac

tgggttgcctatgtggtattggtgtcgatcgacatgaggtgggtgtcgttcgacaccatc

gggaagaagttcctcctcctctgtgaaagtatcgtatccacacgataaaggaaatacaga

gccgtcaagatcaattggtgtcgatcgacaccctgatgtatcaatcgacattgaggcttt

ttctgcaactgggatagatgaggtctgagctgaacgttcacactaaagaattgggtttag

ttgctttccacttcgtagtgtgactgcagtgataaatgctaatgtaggagaaggctaaga

tatatgagatctcagattgtcgatcttcccattaagatctgtgtagagagaatcaaactt

gccagtaaactcaaccacgatctttcctaggtggagaagcaatgcgtgtagatccttgtt

cctctctgcgattgtcggaagcctcagcaaattttcttcgatcatagtctatttcctttg

tggagagacttgtgacagcgttgttgataagaactgatgcttcggttggagtctttgtca

tgaaattgccattacttgcaccaccaaaacataaccgatactgcttatcgattccacgga

agaatatgttgattagtttgctttcctagaaaccatgttgtggacaatccctctgatagg

atttgaatctgatccaagctaccttcaaaccttctgttggagcttgagaaaacaaagaga

tcttctcccttagtaattttgtcactgaatcatcaaggaagtggatcatgaatgcgttct

tcgtgtcactccaattggttagtgaaccgggttctagttgtcgcagccatgatgttcctc

tgccagcgagagagtgtgggaagagtttgcaaagatgataatcagcttgtaccccatctg

ctttgattgaggacactaagtcttcgaagacctctagatgatctagtagtgattcatcaa

aatgacctcgaaactttcctcgtgaaacaagataatagtaggcagggtgaatagcaaatt

cttctcttgcaataggaggtagctggatcgctgatcgattggaatagaaaagctcagctc

tgttgaagtcgcctatggtagctggtttgttctgcagatggtgtagaacgacaccaacag

atggtgtcgaacgacaccaacagatggtgttgaacgacaccaacagggtgtcgatcgaca

ctgtcgttgaaatctgccagctgtttgttgcgttccatcttgtagtttccctgtcatagg

aaaaagaagaaagaaaacgtgtgagaaacaaaagaaagaaaaataaaaacttagtctaaa

ttctacacctagtctaatggtgaatcacagagaacttcggcaaccgcggcaaatttgata

taattcaatttaatcctaagaccactctctcaaataaagaggtcagttgcagcacttagg

gatcgaattcacaaagttctcggatcacgcaggagactaatggctaatcaaattaagcta

agtaaaatagtaaagaaaataaaagaaacaaaaagtaagcaataacattatgagttgtga

aaacaagataataaaggcgttaggcttagggtattctcagaaaatagatgatagtagatc

tagaaatagctagattattatggaaccgttctcgaactcaaactatgattttagagtaac

cggtagtgttccgcaaaactctctatacttagagctaagattaccggaaaagccgagaaa

tcctatactaaagcattcattattatcaagtttgattagttcacctagtatctaaaccac

agcctttctatgagcctacctgttctttcttaaatgcttaggctcatctatgataggtca

aataacaagtacctatggtgggcatacctattatctaatatcaaattctagttaggtact

ctagaactagcattaagaacaatgaagatgaagaatcctacagataacttagcaaggggg

caatctactaaatcatctaaatccctaatgagaaaccctaaacctaacaagaagattact

caaaaatgatcatagaaaaacaaaacatgattgaataagaaatcaatgaataaaacaaga

ggagaataaggtttagaaggatcttctctacagggagtctccatagggttttgctcccaa

agtacagaattgaaaaagggaatagcctcccaaagcttagggtctaaacattaacctcaa

agctatataaatatgtcttaagaacgtgttgggccttcattaaacataggagaaagtttt

gggccgaattcaaaaaatcttcaaaacaacaataagttgcgtctttcaattgccgtcagg

tattggtgtcgcacgacaccaggggtggtgtcgttcgacacctacgtgcattttcgtctc

tggattgcttcttcagcttaaattgtatgttttactccagaatgctccataatcttcaaa

tgcatctaaaacatgtaaagacctgaaaaagactagaaaaaactgtataaataacaatta

gactctaaagcctatacctaaaacatacttaaaaacggtaaaaatagggatatatcaatg

aatcctaacgattaagaaaaataattcattcagcaccaaccgaggtatatactattttac

gatcatgccgtgtgaactaaacatgtaggtgtgacataccaacgataatcatcttgaaaa

acggtggaggtctatatagatgacatgctcgtgaaatcgtcaaaagagagcggtcacatt

taagacttgggagaatgctttaatatcttgaacaagcttcagatgaagttgaatcttgcc

aagtagacattctgagtcccatccggagagtttctcggttacttagcaacaaagagagaa

accgaagcaaacccaagacagattaacgctcttttcgaacatgctagccccaaggaacat

caaagatgtctaaagactctttggtaggattactgcgttaaaccggttaacatctcagtc

agccgataaatgcctttctttctatcaaatactaaaagggaacaaagagttgagttggat

gaaagatgtgaagatgctttcggtcaactaaagacatacttaagtgttgctctaattctc

tgaagcctgaagccgatgaaaaattgtacttatacatctcattttcaaatcatgccgtta

atggagtaataatttctgaagacagaagagagtgaaagccgatttgttacatgagcaagt

catcaaccagtccaaaattaagttacactataatggagaaacttgcgattgatgtagtca

cttcaacttggaagctgcgactttatctatgtctcaccagatcatagttgaaactcttgg

gaatattggttaatttcggcggggttcaagtgtccaattcgctcttcacgaaactagatc

tcatcccaagaaggtttttccaagtttcaactcttggaaaaataagattattttggtgga

ctataagtgttgagttcgctcttcactgaactatctcgtgaagggtttttccaagttcca

actcttggaaaaataaatctctgtgaaatctaagtgttgagatcgctcttcacaaattta

gataccacctcacatcaacaagcatttatgacgaagtctttgagtacaagctcggaagaa

gatcttcagtttctctctagattgctgctcaataacctgcctttgtattctataagcaga

atacactttctaaactcaagcttatagcttcgattcgaaagtcgagttcacttcgcaaca

acttaactcgaggtcagctcccatgcataactcgagtttaattctgataccaaaactcca

tggttgaactgacgagtttgagtcaataaaatggatttaatcctcttggacaaggggtca

actagcaaaacaaagatctttacggtgtttagtcgatctacctaatacttcttaggcgga

gcacaattctcgcaatcaaagaaagtccaaagattgagtttgggggtttgatcaacctat

ttctctcttagccacatggcccaaatttctcttagccacatggcccaaatttttgaaaaa

gtacggactttgcaaatccggcaacttcgaagcaaatactttaagcaagtgtcattccaa

aacccctatggacaacgaaaaggaaaaaagttttaaaaacccctcttgggcacacaacca

acaaattttaaaaccccttgggcaaataaaaattccctttcgggaaagtcgtattcataa

aataaattgggacaatccccagattttatggtaatagccataaaagagttaagatgagct

gaagcctcatcaacactcggtgtaatctcgtcttggttcaaagcaggagtggccgtccct

tgatcgtgagtttcagtttcataattaggagctgcacatgagctggatgaagcatgagca

ttagttaggatacacattttggcgagttgtcattcaagtgtatgaattgattgtgaaagt

tattttatgaattagttctataattaatggcacattggaagaaggaaagaggctataaaa

gagaggtgatgactccaagataaggggggctagagattctatattctgaccacaaatctt

aaacttaaacattgttattttctcataaatacacaaaagattatttcttctctttttatg

taagatctttacataaggaatactataccattttctatgtacaattcttcatacatttct

atatactctcttatacttttcttatgcatatatgtcgtgtacttttgtatttgagagctt

tagcccacaaataatataaatacttgagtgtgtgaatccgacatccataaaagtctgtat

gagtatctggctatgtatcttctagcatggaaacacaactttagcttttaggatctggtt

gtgcttctacttcttatagtcaatcatagacatgacatctagtcatatttgactccaaaa

cactaannnnnnnnnnnnnnnnnnnnnnnnnnnnnnnnnnnnnnnnnnnnngctcgctct

tcacaagactagatttcatccctcaaggatttttccaatttgcatttcttgaaaaaatat

attcatttcggtgaaatctaagtgttgagttcgcacttcacaagactagatttcatccct

caaggatttttccaagttgtatttcttggaaaattatattcatttcggtgaaatctaagt

gttgagttcgcgcttcacaagactagatttcatccctcaaggatttttccaagttccacc

tcttggaaaaatatattcatttttggtgaaatctaaatgttgagtttgttcttcacaaaa

ttagattttatctctcaaggatttttccaagttctacctctcagaaaaatatatttattt

tggtgaaatctgaagtgttgagttcgcaattcacaaaaatagatttcatccctcaatgat

ttttccaagtttcacctcttggaaaaatatattcatttcggtgaaatctaagtgttgagt

tcgctcttcacaagactagatttcatccctcaaggatttttccaagttccacctttwcga

aaaatatatycatttcggttaaatctaagtgttgagctcgatcttcacaagactagattt

catccttcaaggatttttccaagttacaccttttggaaaaatatattcatttcggtgaaa

tggaartgttgagttcgctcttcataaaactagatttcatccctcaaggatttttccaag

ttgcacttcttggaaaaatatattcatttcggtraaatctaagtgttgagtttgcgcttc

acaagactagatttcatccgttaaggatttttccacgttccacctcttggaaaaatatat

tcatttcrgtgaaatctaagtgttgagttcactcttcacaagactagatttcatccctca

aggatttttccaagttccacctcttggaaaaatatatttatttcggtgaaatctaagtgt

tgagtttgctctttacaagactagatttcatccctcaaggatttttccaagttccacctc

ttggaaaaatatattmatttcggtgaaatctaagtgttgagctcgctcttcacaagacta

gattgtatccttcaaggatttttccaagttacaccttttggaaaatatattcatttcggt

waaatcgaagtgttgagttcgctcttcacaagawtagatttcatccctcwaggatttttc

caagttgcacttcttggaaaaatatattcatttcggtgaaatctaagtgttgagtttgct

cttcacaagaatagatttcatcccttaatgatttttccaagttacacctcttggaaaaat

atattaatttcggtaaaatctaaatgtgagttcgctcttcacatgactagatttcatccc

tcaaggatttttccaagttccacctcttggaaaaatatattcatttcggtgaaatttaag

tgttgagctcgctcttcacaagactagattgcatccttcaaggatttttccaagttacac

cttttggaaaaatatatttattttggttaaatcgaagtgttgagttcgctcttcacaaga

atagatttcatccctcaaggaattttcgaagttgcacttcttggaaaaatatattcattt

cggtgaaatctaagtgttgagttcgcgcttcacaagactagattttatccctcaaagatt

ttttcaagttccacctcttggaaaaatatattcattttggtgaaatctaagtgtttagtt

tgctattcacaaaactagatttcatccctcaaaaagttttccaagttccacctctcggaa

aaatatattcatttcggtgaaatcttaagtgttgagttcgcacttcacaagactagattt

catccctcaaggatttttccgagttccaccttttggaaaaatatattcatttcggtgaaa

tataagtgttgagttcgctcttcacaagmctagatttcatcactcaatgatttatccaag

tttcacctctcggaaaaaaatatwttcatttcggtgaaatttaagtgttgagcttgcttt

tcacaagactagatttcatccctcaaggatttttccaagtttcacctcttggaaaaatat

tttcatttcggtgaaatctaagtgttgagttcgctattcacaagactagatttcatccct

caaggatttttctaacttccacattttggaaaaatatattcatttcggtgaaatctaagt

gttgagctcgctcttcacaagactagatttcatccttcaaggatttttccaagttacacc

ttttggaaaaatatattcattttggtgaaatggaagtgttgagttcgctcttcacaagac

tagatttcatccctcaaggatttttccaagttgcacttcttggaaaaatatattcattta

ggtgaaatctaagtgttgagctcgctcttcacaagactagattgtatccttcaaggattt

ttccaagttacaccttttggaaaaaatattcatttctgtaraatcgaagtgttgagttcg

ctcttcacaagaatagatttcatcccttaatgatttttcctagttacgcc

[gapbp][ExpandNs](http://www.ncbi.nlm.nih.gov/nuccore/14192923?fmt_mask=65536)

acaagactagatttcatccttcaag

gatttttccaagttctacctctcagaaaaatatatttatttcggtgaaatctgaagtgtt

gagttcgcaattcacaagactagatttcatccctcaaggatttttccaagtttcacctct

tggaaaaatatattcatttcggtcaaatctaagtgttgagttcgctcttcacaagactag

atttcatccctcaaggacttttccaagttccaccgtttagaaaaatatattcatttcggt

gaaatctaagtgttgagctcgctcttcacaagactagatttcatccttcaaggatttttc

caagttacaccttttaaaaaaatatattcatttcggtgaaatggaagtgttgagttcgct

attcacaagactagatttcatccctcaaggatttttccaaattgcacttcttggaaaaat

atattcatttyggtgaaatctaagtgttgagtttgcgcttcacaagactagatttcatcc

gtcaaggatttttccaagttccacctcttggaaaaatatattcattttggtgaaatctaa

gtgttgagttttcccttcacaaaactagattttatctctcaaggatttttccaagttcca

cctctcggaaaaatatattcatttaagtgaaattttaagtgttgagttcgcattttacaa

gactagaattcatccctcaaggatttttccaagttccacctcttggaaaaatatattcat

ttcggtgaaatataagtgttgagttcgctcttcacaagactagatttcatcactcaaaaa

tttttccaagttccacctcttggaaaaaaatatattcatttcggtgaaatttaagtgttg

agcttgctcttcacaagactagatttcatccctcaaggatttttcccagttacacctttt

gggaaaatatattcattttggtgaaatggaagtgttgagttcgctcttcacaagactaga

tttcatccctcaaggatttttccaagttgcacttcttggaaaaatatattcatttcggtg

aaatctaagtgttgagtttgcgcttcacaagactagatttcatccgtcaaggatttttcc

aagtttcacctcttgaaaaaatatatttattttggtgaaatctaagtgttgagtttgctc

ttcacaaaactagattttatctctcaaggatttttccaagttccacctctcggaaaaata

tattcatttaagtaaaatattaagtgttgatttcgcacttcacaagactagaattcatcc

ctctaggatttttccaagttccacctcttggaaaaatatattcatttcggtgaaatttaa

gtgttgaactcgctcttcacaagactagattgcatccttcaaggatttttccaagttaca

ccttttggaaaaatatatttatttcggtgaaatcgaagtgttgagttcgctcttcacaag

aatagatttcatccctcaaggaattttccaagttgcatttcttggaaaaatatattcatt

tcggtgaaatctaagtgttgagtttgcgcttcacaagactagattttatccgtcaaggat

ttttccaagtttcacctcttgaaaaaatatatttattttggtgaaatctaagtgttgagt

ttgctcttcacaaaactagattttatctctcaaggatttttccaagttccacctctcgga

aaaatatattcatttaagtaaaatattaagtgttgatttcgcacttcacaagactagaat

tcatccctctaggatttttccaagttccacctcttggaaaaatatattcatttcggtgaa

atttaagtgttgaactcgctcttcacaagactagattgcatccttcaaggatttttccaa

gttacaccttttggaaaaatatatttatttcggtgaaatcgaagtgttgagttcgctctt

cacaagaatagatttcatccctcaaggaattttccaagttgcacttcttggaaaaatata

ttcattttggtgaaatctaagtgttgagtttgcgcttcacaagactagatttcatccgtc

aagtatttttccaagttccacctcttggaaaaatatattcattttggtgaaatctaggtg

ttgagtttgctcttcacaaaattaaattttatcactcaaggatttttccaagttccacct

ctcggaaaaatatattcatttaagtaaaatcttaagtgttgagttcgcacttcacaagaa

tagaattcatccctcaaggatttttccaagttccacctcttggaaaaatatattcatttc

ggtgaaatctaagtgttgtgttcactcatcacaagagtagaattcatccctcaaggactt

ttccaagttccacctcttcgaaaaatatattcatttcggtgaaatctaagtgttgagctc

gctcttcacaagactagatttcatccgtcaaggatttttccaagttgcatttcttgaaaa

aatatattcatttcagtgaaatctaagtgttgagttcgcgcttcacaagactagatttca

tccctcaaggatttttccaagttgcatttcttggaaaaatatattcatttagatgaaatc

taagtgttcagttcgcgcttcacaagactagatttcatccctcaaggatttttccaagtt

ccacctcttggaaaaatatattcatttttggtgaaatctaagtgttgagtttgctcttca

caaaactagatttatctctcaaggatttttccaagttccacctctcggaaaaatatatty

atttaagtaaaatattaagtgttgatttcgcacttcacaagactagaattcatccctcta

ggatttttccaagttccacctcttggaaaaatatattcatttcggtgaaatttaagtgtt

gaactcgctcttcacaagactagattgcatccctcaaggacttttccaagttccaccgtt

tggaaaaatatattcatttcggtgaaatctaagtgttgagctcgctcttcacaagactag

atttcatccttcaaggatttttccaagttacaccttttaaaaaaatatattcatttcggt

gaaatggaagtgttgagttcgctattcacaagactagatttcatccctcaaggatttttc

caaattgcacttcttggaaaaatatattcattttggtgaaatctaagtgttgagtttgcg

cttcacaagactagatttcatccgtcaaggatttttccaagttccacctcttggaaaaat

atattcattttggtgaaatctaagtgttgagttttcccttcacaaaactagattttatct

ctcaaggatttttccaagttccacctctcggaaaaatatattcatttaagtgaaatttta

agtgttgagttcgcattttacaagactagaattcatccctcaaggatttttccaagttcc

acctcttggaaaaatatattcatttcggtgaaatataagtgttgagttcgctcttcacaa

gagtagatttcatcactcaaaaaattttccaagttccacctcttggaaaaaaatatattc

atttcggtgaaatttaagtgttgagcttgctcttcacaagactagatttcatccctcaag

gatttttcccagttacaccttttggaaaaatatattcattttggtgaaatggaagtgttg

agttcgctcttcacaagactagatttcatccctcaaggatttttccaagttgcacttctt

ggaaaaatatattcatttcggtgaaatctaagtgttgagttcgctcttcacaagactaga

tttcatccctcaaggacttttccaagttccaccgtttggaaaaatatattcatttcggtg

aaatctaagtgttgagctcgctcttcacaagactagatttcatccttcaaggatttttcc

aagttacaccttttaaaaaaatatattcatttcggtgaaatggaagtgttgagttcgcta

ttcacaagactagatttcatccctcaaggatttttccaaattgcacttcttgaaaaaata

tattcatttaagtgaaatctaagtgttgagtttgcgcttcacaagactagatttcatccg

tcaaggatttttccaagttccacctcttggaaaaatatattcattttggtgaaatctagg

tgttgagtttgctcttcacaaaattagattttatcactcaaggatttttccaagttccac

ctctcggaaaaatatattcatttaagtaaaatcttaagtgttgagttcgcacttcacaag

actagaattcatccctcaaggatttttccaagttccacctcttggaaaaatatattcatt

tcggtgaaatctaagtgttgtgttcactcatcacaagagtagaattcatccctcaaggac

ttttccaagttccacctcttcgaaaaatatattcatttcggtgaaatctaagtgttgagc

tcgctcttcacaagactagatttcatccgtcaaggatttttctttccagcacagggga

[gapbp][ExpandNs](http://www.ncbi.nlm.nih.gov/nuccore/14192923?fmt_mask=65536)

accttttggaaaaatat

attcattttggtgaaatggaagtgttgagttcgctcttcacaagactagatttcatccct

caaggatttttccaagttgcacttcttggaaaaatatattcatttcggtgaaatctaagt

gttgagtttgcgcttcacaagactagatttcatccgtcaaggatttttccaagtttcacc

tcttgaaaaaatatatttattttggtgaaatctaagtgttgagtttgctcttcacaaaac

tagattttatctctcaaggatttttccaagttccacctctcggaaaaatatattcattta

agtaaaatattaagtgttgatttcgcacttcacaagactagaattcatccctctaggatt

tttccaagttccacctcttggaaaaatatattcatttcggtgaaatctaagtgttgtgtt

cactcatcacaagagtagaattcatccctcaaggacttttccaagttccacctcttcgaa

aaatatattcatttcggtgaaatctaagtgttgagctcgctcttcacaagactagatttc

atccgtcaaggatttttccaagttgcatttcttgaaaaaatatattcatttcagtgaaat

ctaagtgttgagttcgcgcttcacaagactagatttcatccctcaaggatttttccaagt

tgcatttcttggaaaaatatattcatttagatgaaatctaagtgttcagttcgcgcttca

caagactagatttcatccctcaaggatttttccaagttccacctcttggaaaaatatatt

catttttggtgaaatctaagtgttgagtttgctcttcacaaaactagatttatctctcaa

ggatttttccaagttctacctctcagaaaaatatatttatttcggtgaaatctgaagtgt

tgagttcgcaattcacaagactagatttcatccctcaaggatttttccaagtttcacctc

ttggaaaaatatattcatttcggtgaaatctaagtgttgagttcgctcttcacaagagta

gatttcatccctcaaggacttttccaagttccaccgtttggaaaaatatattcatttcgg

tgaaatctaagtgttgagctcgctcttcacaagactagatttcatccttcaaggattttt

ccaagttacaccttttaaaaaaatatattcatttcggtgaaatggaagtgttgagttcgc

tattcacaagactagatttcatccctcaaggatttttccaaattgcacttcttggaaaaa

tatattcatttcggtgaaatctaagtgttgagtttgcgcttcacaagactagatttcatc

cgtcaaggatttttccaagttccacctcttggaaaaatatattcattttggtgaaatcta

agtgttgagttttctcttcacaaaactagattttatctcttaaggatttttccaagttcc

acctctcggaaaaatatattcatttaagtgaaattttaagtgttgagttcgcattttaca

agactagaattcatccctcaaggatttttccaagttccacctcttggaaaaatatattca

tttcggtgaaatataagtgttgagttcgctcttcacaagactagatttcatcactcaaaa

aattttccaagttccacctcttggaaaaaaatatattcatttcggtgaaatttaagtgtt

gagcttgctcttcacaagactagatttcatccctcaaggatttttcccagttacaccttt

tggaaaaatatattcattttggtgaaatggaagtgttgagttcgctcttcacaagactag

atttcatccctcaaggatttttccaagttgcacttcttggaaaaatatattcatttcggt

gaaatctaagtgttgagttcgctcttcacaagactagatttcatccctcaaggacttttc

caagttccaccgtttggaaaaatatattcatttcggtgaaatctaagtgttgagctcgct

cttcacaagactagatttcatccttcaaggatttttccaagttacaccttttaaaaaaat

atattcatttcggtgaaatggaagtgttgagttcgctattcacaagactagatttcatcc

ctcaaggatttttccaaattgcacttcttgaaaaaatatattcatttaagtgaaatctaa

gtgttgagtttgcgcttcacaagactagatttcatccgtcaaggatttttccaagttcca

cctcttggaaaaatatattcattttggtgaaatctaggtgttgagtttgctcttcacaaa

attagattttatcactcaaggatttttccaagttccacctctcggaaaaatatattcatt

taagtaaaatcttaagtgttgagttcgcacttcacaagactagaattcatccctcwagga

tttttccaagttccacctcttggaaaaatatattcatttcggtgaaatctaagtgttgtg

ttcactcatcacaagagtagaattcatccctcaaggacttttccaagttccacctcttcg

aaaaatatattcatttcggtgaaatctaagtgttgagctcgctcttcacaagactagatt

tcatccgtcaaggatttttccaagttgcatttcttgaaaaaatatattcatttcagtgaa

atctaagtgttgagttcgcgcttcacaagactagatttcatccctcaaggatttttccaa

gttgcatttcttggaaaaatatattcatttagatgaaatctaagtgttcagttcgcgctt

cacaagactagatttcatccctcaaggatttttccaagttccacctcttggaaaaatata

ttcatttttggtgaaatctaagtgttgagtttgctcttcacaaaactagattttatctct

caaggatttttccaagttctacctctcagaaaaatatatttatttcggtgaaatctgaag

tgttgagttcgcaattcacaagactagatttcatccctcaaggatttttccaagtttcac

ctcttggaaaaatatattcatttcggtcaaatctaagtgttgagttcgctcttcacaaga

ctagatttcatccctcaaggacttttccaagttccaccgtttagaaaaatatattcattt

cggtgaaatctaagtgttgagctcgctcttcacaagactagatttcatccttcaaggatt

tttccaagttacaccttttaaaaaaatatattcatttcggtgaaatggaagtgttgagtt

cgctattcacaagactagatttcatccctcaaggatttttccaaattgcacttcttggaa

aaatatattcatttcggtgaaatctaagtgttgagtttgcgcttcacaagactagatttc

atccgtcaaggatttttccaagttccacctcttggaaaaatatattcattttggtgaaat

ctaagtgttgagttttctcttcacaaaactagattttatctctcaaggatttttccaagt

tccacctctcggaaaaatatattcatttaagtgaaattttaagtgttgagttcgcatttt

acaagactagaattcatccctcaaggatttttccaagttccacctcttggaaaaatatat

tcatttcggtgaaatataagtgttgagttcgctcttcacaagactagatttcatcactca

aagatttttccaagtaccacctcttggaaaaaaatatattcatttcggtgaaatttaagt

gttgagcttgctcttcacaagactagatttcatccctcaaggatttttcccagttacacc

ttttggaaaaatatattcattttggtgaaatggaagtgttgagttcgctcttcacaagac

tagatttcatccctcaaggatttttccaagttgcacttcttggaaaaatatattcatttc

ggtgaaatctaagtgttgagtttgcgcttcacaagactagatttcatccgtcaaggattt

ttccaagtttcacctcttgaaaaaatatatttattttggtgaaatctaagtgttgagttt

gctcttcacaaaactagattttatctctcaaggatttttccaagttccacctctcggaaa

aatatattcatttaagtaaaatattaagtgttgatttcgcacttcacaagactagaattc

atccctctaggatttttccaagttccacctcttggaaaaatatattcatttcggtgaaat

ctaagtgttgtgttcactcatcacaagagtagaattcatccctcaaggayttttccaagt

tccacctcttggaaaaatatatttatttcggtgaaatctaagtgtagagtttgctcttta

caagactagatttcatccctcaaggattatttcaagatccacctcttggaaaaatatatt

aatttcggtgaaatnnnnnnnnnnnnnnnnnnnnnnnnnnnnnnnnnnnnnnnnnnnnnc

tagattgtatccttcaaggatttttccaagttacaccttttggaaaaatatattcatttc

ggtaaaatcgaagtgttgagttcactcttcacaagaatagatttcatcccttaatgattt

ttccaagttacacctcttggaaaaatatattcatttcggtgaaatttaaatgtgagttcg

ctcttcacaagactagatttcatccctcaaggatttttcaagttccacctcttgaaaaaa

tatattcatttcggtgacatttaagtgttgagctcgctcttcacaagactagattgcatc

cttcaaggatttttccaagttacaccttttggaaaaatatatttatttcggtgaaatcga

agtgttgagttcgctcttcacaagaatagatttcatccctcaaggaattttccaagttgc

acttcttggaaaaatatattcatttcggtgaaatccaagtgttgagttcgcgcttcacaa

gactagattttatccgttaagcattttttcaagttccacctctcggaaaaatatacacat

ttcggtgaaatcttaagtgttgagttcgcacttcacaagactagatttcatccctcaagg

atttttccgagttccacctcttggaaaaatatattcatttcggtgaaatctaagtgttga

gtttgcgcttcacaagactagatttcatccgtcaaggatttttccaagttccacctcttg

gaaaaatatattcattttggtgaaatctaagtgttgggttttctcttcacaaaactagat

tttatctctcaaggatttttccaagttccacctctcggaaaaatatattcatttaagtga

aattttaagtgttgagttcgcacttcacaagactagaattcatccctcaaggatttttcc

aagttccacctcttggaaaaatatattcatttcggtgaaatctaagtgttgagtttgctc

tttacaagactagatttcatccctcaaggatttttccaagttccacctcttggaaaaata

tattcatttcggtgaaatctaagtgttgagctcgctcttcacaagactagattgtatcct

tcaaggatttttccaagttacaccttttggaaaaatatattcatttcggtaaaatcgaag

tgttgaattcgctcttcacaagaatagatttaatcccttaatgatttttccaagtaacac

ctcttggaaaaatatattcatttcggtgaaatctaaatgtgagttcgctcttcacaagac

tagatttcatccctcaacgatttttcaagttccacctcttggaaaaatatattcatttcg

gtgacatttaagtgttgagctcgctcttcacaagactagattgcatccttcaaggatttt

tccaagttacaccttttggaaaaatatatttatttcagtgaaatcgaagtgttgagttcg

ctcttcacaagaatagatttcatccctcaaggaattttccaagttccacctcttggaaaa

atatattcattttggtgaaatctaagtgtttagtttgctattcacaaaactaaatttctt

ccctcaaaaagttttccaagttccacctctcggaaaaatatattcatttcggtgaaattt

taagtgttgagttcgcacttcacaagactagatttcatccctgaaggatttttccgagtt

ccgccttttggaaaaatatattcatttcggtgaaatggaagtgttgagtttgctcttcac

aagactagatttcatcccttaaggatttttccaaattgcacttcttcgaaaatatattca

tttcggtgaaatctaagtgttgagtttgcgcttcacaaaactagattttatccctcaagg

atttttccaagttccacctcttggaaaaatatattcatttcggtgaaatctaagtgttga

gctcgctcttcacaagactagatttcatccgtcaaggatttttccaagttgcatttcttg

aaaaaatatattcatttcagtgaaatctaagtgttgagttcgcgcttcacaagactagat

ttcatccctcaaggatttttccaagttgcatttcttggaaaaatatattcatttaagtaa

aatattaagtgttgagttcgcacttcacaagactagaattcatccctctaggatttttcc

aagttccacctcttggaaaaatatattsatttcggtgaaatctaagtgttgagttcgctc

ttcacaagactagatttcatccctcaaggacttttccaagttccgccttttggaaaaata

tattcatttcggtgaaatctaagtgttgagctcgctcttcacaagactagatttcatcct

tcaaggatttttccaagttacaccttttaaaaaaatatattcatttcggtgaaatggaag

tgttgagtttgctcttcacaagactagatttcatccctgaaggatttttccaaattgcac

ttcttcgaaaaatatattcatttcggtgaaatctaagtgttgagtttgcgcttcacaaaa

ctagattttatccctcaaggatttttccaagttccacctcttggaaaaatatattcattt

cggtgaaatctaagtgttgagctcgctcttcacaagactagatttcatccgtcaaggatt

tttccaagttgcatttcttgaaaaaatatattcatttcagtgaaatctaagtgttgagtt

cgtgcttcacaagactagatttcatccctcaaggatttttccaagttgcatttcttggaa

aaatatattcatttaagtaaaatattaagtgttgagttcgcacttcacaagactagaatt

catccctctaggatttttccaagttccacctcttggaaaaatatattsatttcggtgaaa

tctaagtgttgagttcgctcttcacaagactagatttcatccctcaaggacttttccaag

ttccgccttttggaaaaatatattcatttcggtgaaatctaagtgttgagctcgctcttc

acaagactagatttcatccttcaaggatttttccaagttacaccttttaaaaaatatatt

catttcggtgaaatggaartgttgagttcgctcttcacaagactagatttcatccctgaa

ggatttttccaaattgcacttcttggaaaaatatattcatttcggtgaaatctaagtgtt

gagtttgcgcttcacaagactagatttcatccgtcaaggatttttccaagttccacctct

tggaaaaatatattcattttggtgaaatctaagtgttgagttttctcttcacaaaactaa

tttttatctctcaaggatttttctaagttgcacctctcggaaaaatatattcatttaagt

gaaattttaagtgttgagttcgcacttcacaagactagaattcatccctcaaggattktt

ccaagttctacctcttggaaaaatatattcatttcggtgaaatctaagtgttgagtttgc

tctttacaagactagatttcatccctcaagaatttttccaagttccacctcttggaaaat

atattcatttcggtgaaatctaagtgttgagctcgctcttcaaaagactagattgtatcc

ttcaaggatttttccaagttacaccttttggaaaaatatattcatttcggtaaaatcgaa

gtgttgaattcgctcttcacaagaatagatttcatcccttaatgatttttccaagtaaca

cctcttggtaaaatatatccgtttcggtaaaatctaagtgttgagttcgctcttcacaat

actatatatcatccttcaagaatttttccaagcttcacctcttgaaaaaatattttcatt

tcagcgaaatctaagtgttgagctcgctcttcacaagactagatttcatccctcaacgat

ttttccaagttacaccttttggaaaaatatatttatttaagtgaaatcgaagtgttgagt

tcgctcttcacaagaatagatttcatccctcaaggaattttccaagttccacctcttgga

aaaatatattcattatagtgaaatctaagtgtttagtttgctattcacaaaactaaattt

cttccctcaaaaagttttccaagttccacctctcggaaaaatatattcatttcggtgaaa

ttttaagtgttgagttcgcacttcacaagactagatttcatccctcaaggatttttccga

gttccacctcttggaaaaatatattcatttcggtgaaatataagtcttgagttcgctctt

cacaagactagatttcatcactcaaagatttttccaagttctacctctcggaaaaaaata

tattcatttcggtgaaatttaagtgttgagcttgctcttcacaagactagattttatccc

tcaaggatttttccaagttacaccttttggaaaaatatattcattttggtgaaatggaag

tgttgagttcgctcttcacaagactagatttcatccctcaaggatttttccaagttgcac

ttcttggaaaaatatattcatttcggtgaaatctaagtgttgagtttgcgcttcacaaga

ctagatttcatccgtcaaggatttttccaagtttcacctcttggaaaaatatatttattt

tggtgaaatctaagtgttgagtttgctcttcacaaaactagattttatctctcaaggatt

tttccaagttccacctctcggaaaaatatattcatttaagtaaaatattaagtgttaagt

tcgcacttcacaagactagaattcatccctcaagtatttttccaagttccacctcttgga

aaaatatattcgtttcggtgatatctaagtgttgagttcactcttcacaagactagattt

catcccttaaggatttttccaagttccacctcttggaaaaatatattcatttcggtgaaa

tctaagtgttgagctcgctcttcacaagactagatttcatcccacaaggatttttccaat

ttgcatttcttgaaaaaatatattaatttcggtaaaatctaagtgttgagttcgcgcttc

acaagactagatttcatccctcaaagatttttccaagttgcatttcttggaaaaatatat

tcatttaggtgaaatctaagtgttcagttcgcgcttcacaagattagatttcatccctca

aggatttttccaagttccacctcttggaaaaatatattcatttttggtgaaatctaagtg

ttgagtttgctcttcacaaaactagattttatctctcaaggatttttccaagttctacct

cttagaaatatatatttatttcggtgaaatctgaagtgttgagttcgcaattcacaagac

tagatttcatccctcaaggatttttccaagtttcacctcttggaaaaatatattcatttc

ggtgaaatctaagtgttgagtttgctcttcacaagactagatttcatccctcaaggactt

ttcmaagttccaccttttggaaaaatatattcatttcggtgaaatctaagtgttgagctc

gctcttcacaagactagatttcatccttcaaggatttttccaagttacaccttttaaaaa

aatatattcatttcggagaaatggacgtgttgagttcgctcttcacaagactagatttca

tccctcaaggatttttccaaattgcacttcttgaaaaaatatattcatttcggtgaaatc

taagtgttgagtttgcgcttcacaagactagatttcatccgtcaaggatttttctaagtt

ccacctcttggaaaaatatattcattttggtgaaatctaagtgttgagttttctcttcag

aaaactagattttatctctcaaggatttttctaagttccacctctcggaaaaaaatattc

atttaagtgaaattttaagtgttgagttcgcacttcacaagactagaattcatccctcaa

ggatttttccaagttccacctcttggaaaaatatattcatttcggtgaaatctaagtgtt

gagtttgctctttacaagactagatttcatccctcaaggatttttccaagttccacctct

tggaaaaatatattcatttcggtgaaatctaagtgttgagctcgctcttcacaaaactag

attgtatccttcaaggatttttccaagttacaccttttggaaaaatatattcatttcggt

aaaatcgaagtgttgaattcgctcttcacaagaatagatttcatcccttaatgatttttc

caagttacacctcttggaaaaatatattcatttcggtgaaatctaaaagtgagttcgctc

ttcacaagactagatttcatccctcaaggatttttcaagttccacctcttgaaaaaatat

attcatttcggtgacatttaagtgttgagctcgctcttcacaagactagattgcatcctt

caaggacttttccaagttacaccttttggaaaaatatatttatttcattgaaatcgaagt

gttgagttcactcttcacaagaatagattttgtccctcaaggaattttccaagttgcagt

tcttggaaaaatatattcatttcagtgaaatctaagtgttgagttcgcgtttcacaagac

tagattttatccctcaaggattttttcgagttccacctcttggaaaaatatattcatttt

ggtgaaatctaagtgtttagtttgctattcacaaaactaaatttcatccctcaaaaagtt

ttccaagtttcacctctcggaaaaatatattcatttcggtgaaatcttaagtgttgagtt

cgcacttcacaagactagatttcatccctcaaggatttttccgagttccacctcttggaa

aaatatattcatttcggtgaaatataagtgttgagttcgctcttcaaacgactagatttc

atcactcaaagatttttccaagttccacctctcggaaaaaaatatattcatttcggtgaa

atttaagtgttgagcttgctcttcacaagactagatttcatccctcaaggatttttccaa

gttccacctctcggaaaaatatattcatttaagtaaaatataaagtgttaagttcgcact

tcacaagactagaattcatccctcaaggatttttccaagttccacctcttggaaaaatat

attcatttcggtgaaatctaagtgttgagttcactcttcacaagactagatttcatccct

caaggatttttccaagttccacctcttggaaaaatatattcatttcggtgaaatctaagt

gttgagctcgctcttcacaagactagaattcatccctcaaggatttttccaatttgcatt

tcttgaaaaaatatattcatttcggtgaaatctaagtgttgagttcgcgcttcacaagac

tagatatcacccctcaaggattttttcaagttgcatttcttggaaaaatatattcatttc

ggtgaaatctaagtgttgagttcgcgcttcacaagactagatttcatccctcaaggattt

ttccatgttccacctcttggaaaaatatattcatttttggtgaaatctaagtgttgagtt

tgctcttcacaaaactagattttatctctcaaggatttttccaagttctacctctcagaa

aaatatatttatttcggtgaaatctgaagtgttgagttcgcaattcacaagactcgattt

catccctcaaggatttttccaagtttcacctcttggaaaaatatattcatttcggtgaaa

tctaagtgtttagttcgctcttcagaagactagatttcatccatcaaggacttttccaag

ttccgtcttttggaaaaatatattcatttcggtgaaatctaagtgttgagctcgctcttc

acaagactagatttcatccttcaaggatttttccaagttacaccttttaaaaaaatatat

tcatttcggtgaaatggaagtgttgagttcgctcttcacaagactagatttcatcactca

aggatttttccaaattgcacttcttggaaaaatatattcatttcggtgaaatctaagtgt

tgagtttgcgcttcacaagactagatttcatccgtcaaggatttttccaagttccacctc

ttggaaaaatatattcattttggtgaaatctaagtgttgagttttctcttcacaaaacta

gattttatctctcaaggatttttccaagttccacctctcggaaaaatatattcatttaag

tgaaattttaagtgttgtgttcgcacttcacaagactagaattcatccctcaaggatttt

tccaagttccacctcttggaaaaatatattcatttcggtgaaatctaagtgttgagtttg

ctctttacaagactagatttcatccctcaaggatttttccaagttccacctcttggaaaa

atatattcatttcggtgaaatctaagtgttgagctcgctcttcacaagactagattgtat

ccttcaaggatttttccaagttacaccttttgsaaaaatatattcatttcggtaaaatcg

aagtgctgagttcgctcttcacaagaatagatttcatcccttaatgatttttccaagtta

cacctcttggaaaaatatattcatttcggtgaaatctaaatgtgagttcgctcttcacaa

gactagatttcatccctcaaggatttttcaagttccacctcttggaaaaatatattcatt

tcggtgacatttaagtgttgagctcgctcttcacaagactagattgcatcgttcaaggat

tttttcaagttacaccttttggaaaaatatatttatttcggtgaaatcgaagtgttgagt

tcgctcttcacaagaatagatttcatccctcaaggaattttccaagttgtacttcttgga

aaaatatattcatttcggtgaaatccaagtgttgagttcgcgcatcacaagactagattt

tatccgtcaaggattttttcaagttctacctcttggaaaaatatattcattttggtgaaa

tctaagtgtttagtttgctattcacaaaactaaattttatccctcaaaaagttttccaag

ttccacctctgggaaaaatatatttatttcggtgaaatcttaagtgttgagttcgcactt

cacaagactagatttcatccttcaaggatttttccgagttccacctcttggaaaaatata

ttcatttcggtgaaatataagtgttgagttcgctcttcacaagactagatttcatcactc

aaagatttttccaagttccagctctcggaaaaaaatatattcatttcggtgaagtttaag

tgttgagcttgctcttcacaagactagatttcatccctcaaggatttttccaagttacac

cttttggaaaaatatattcatcttggtgaaatggaagtgttgagttcgctcttcacaaga

ctagatttcatccctcaaggatttttccaagtttcacttcttggaaaaatatattcattt

cggtgaaatctaagtgttgagtttgckcttcacaagactagatttcatccgtcaaggatt

tttycaagttccacctcttgaaaaaatatattcactttggtgaaatctaagtgttgagtt

ttctcttcacaaaactagattttatctctcaaggatttttccaagttccacctctcggaa

aaatatattcatttaagtgaaattttaagtgttgagttcgcacttcacaagactagaatt

catccctcaaggatttttccaagttccacctcttggaaaaatatattcatttcggtgaaa

tctaagtgttgagtttgctctttacaagactagatttcatccatcaaggatttttccaag

ttccacctcttggaaaaatatattcatttcggtgaaatctaagtgttgagctcgctcttc

acaagactagattgtatccttcaaggatttttccaagttacaccttttggaaaaatatat

tcatttcggtaaaatcgaagtgttgagttcactcttcacaagaatagatttcatccctta

atgatttttccaagttacacctcttggaaaaatatattcatttagctcgctcttcacaag

actagatttcatc

**T14C8 sequence**

AAGCTTCAGATGAAGTTGAATCTTGCCAAGTAGACATTCTGAGTCCCATCCGGAGAGTTTCTCGGTTACTTAGCAACAAAGAGAGAAACCGAAGCAAACCCAAGACAGATTAACGCTCTTTTCGAACATGCTAGCCCCAAGGAACATCAAAGATGTCTAAAGACTCTTTGGTAGGATTACTGCGTTAAACCGGTTAACATCTCAGTCAGCCGATAAATGCCTTTCTTTCTATCAAATACTAAAAGGGAACAAAGAGTTGAGTTGGATGAAAGATGTGAAGATGCTTTCGGTCAACTAAAGACATACTTAAGTGTTGCTCTAATTCTCTGAAGCCTGAAGCCGATGAAAAATTGTACTTATACATCTCATTTTCAAATCATGCCGTTAATGGAGTAATAATTTCTGAAGACAGAAGAGAGTGAAAGCCGATTTGTTACATGAGCAAGTCATCAACCAGTCCAAAATTAAGTTACACTATAATGGAGAAACTTGCGATTGATGTAGTCACTTCAACTTGGAAGCTGCGACTTTATCTATGTCTCACCAGATCATAGTTGAAACTCTTGGGAATATTGGTTAATTTCGGCGGGGTTCAAGTGTCCAATTCGCTCTTCACGAAACTAGATCTCATCCCAAGAAGGTTTTTCCAAGTTTCAACTCTTGGAAAAATAAGATTATTTTGGTGGACTATAAGTGTTGAGTTCGCTCTTCACTGAACTATCTCGTGAAGGGTTTTTCCAAGTTCCAACTCTTGGAAAAATAAATCTCTGTGAAATCTAAGTGTTGAGATCGCTCTTCACAAATTTAGATACCACCTCACATCAACAAGCATTTATGACGAAGTCTTTGAGTACAAGCTCGGAAGAAGATCTTCAGTTTCTCTCTAGATTGCTGCTCAATAACCTGCCTTTGTATTCTATAAGCAGAATACACTTTCTAAACTCAAGCTTATAGCTTCGATTCGAAAGTCGAGTTCACTTCGCAACAACTTAACTCGAGGTCAGCTCCCATGCATAACTCGAGTTTAATTCTGATACCAAAACTCCATGGTTGAACTGACGAGTTTGAGTCAATAAAATGGATTTAATCCTCTTGGACAAGGGGTCAACTAGCAAAACAAAGATCTTTACGGTGTTTAGTCGATCTACCTAATACTTCTTAGGCGGAGCACAATTCTCGCAATCAAAGAAAGTCCAAAGATTGAGTTTGGGGGTTTGATCAACCTATTTCTCTCTTAGCCACATGGCCCAAATTTCTCTTAGCCACATGGCCCAAATTTTTGAAAAAGTACGGACTTTGCAAATCCGGCAACTTCGAAGCAAATACTTTAAGCAAGTGTCATTCCAAAACCCCTATGGACAACGAAAAGGAAAAAAGTTTTAAAAACCCCTCTTGGGCACACAACCAACAAATTTTAAAACCCCTTGGGCAAATAAAAATTCCCTTTCGGGAAAGTCGTATTCATAAAATAAATTGGGACAATCCCCAGATTTTATGGTAATAGCCATAAAAGAGTTAAGATGAGCTGAAGCCTCATCAACACTCGGTGTAATCTCGTCTTGGTTCAAAGCAGGAGTGGCCGTCCCTTGATCGTGAGTTTCAGTTTCATAATTAGGAGCTGCACATGAGCTGGATGAAGCATGAGCATTAGTTAGGATACACATTTTGGCGAGTTGTCATTCAAGTGTATGAATTGATTGTGAAAGTTATTTTATGAATTAGTTCTATAATTAATGGCACATTGGAAGAAGGAAAGAGGCTATAAAAGAGAGGTGATGACTCCAAGATAAGGGGGGCTAGAGATTCTATATTCTGACCACAAATCTTAAACTTAAACATTGTTATTTTCTCATAAATACACAAAAGATTATTTCTTCTCTTTTTATGTAAGATCTTTACATAAGGAATACTATACCATTTTCTATGTACAATTCTTCATACATTTCTATATACTCTCTTATACTTTTCTTATGCATATATGTCGTGTACTTTTGTATTTGAGAGCTTTAGCCCACAAATAATATAAATACTTGAGTGTGTGAATCCGACATCCATAAAAGTCTGTATGAGTATCTGGCTATGTATCTTCTAGCATGGAAACACAACTTTAGCTTTTAGGATCTGGTTGTGCTTCTACTTCTTATAGTCAATCATAGACATGACATCTAGTCATATTTGACTCCAAAACACTAAACAAGCTTCTTCTTGCTTCTCAAAGCTTTGATAGTGACGTCGAAGTCCGTATGAGTCTTTGGCTTTGTATCTTCTAACAAGGAAACAATAATTAAGGTTTCAAGATCCGGTTGCGGTTCTAGTTCTTATACTCAATCATACACATGACATATAGTCATATTTAACTCCAAAACACTAACTAAGCTTCTTTTTGCTTCTCAAATCTTTGATGGTGAAGACAAAGTCCGTATGAGTCTTTGGCTTTCTATCTTCAAACAAGGAAACACTACTTAGGCTTTCAAGATCCGGTTGCGATTCTAGTTCTTATACTCAATCATACACATGACGTCTAGTCATATCTGACCCCTAAACACTAACCAAGCTTCTTCGTGCTTCTCACAGCTTTGATGGTGTCGTCGAAGTCCGTATGAGTCTTTGGATTTGTATCTTCTAACAAGGAAACATTACATAGGATTTTAAGATTAGTTTGCGGTTCTAGTTCTTATACTGGATCATAACCATGACATCTAAACAAATTTGAGTCCAAAACACTAACCAAGCTTCTTCTTACTTCCCAAAGCTTTGATGGTGTAGCCCAAGTCCGTATGAGTCTTTGGCTTTGTATCTCTACTAATAAGGAGACTCTACTTAGGCTTCTAAGATCTCGTTGAGGTTCTAGTTCTTATACTCAATCATACACATGACATCTAGTCATATTTGACTCCAAAACACTAATCAAGCTTCTTCTTGCTTTTCAAAGATTTGATACTGAAGCTGAAGTCTATATGAGTTTTTGGCTTTGTATCTTTTAACAAGGAAACAGTACTTAGGCTTTCAAGATCCAGTTATTGTTCTAGTTCTTTTGCTCACCCATACACATGAAATCTAGTCATATTTGACTCCATAACACTAACCAAACTTATTCTTGCTTCTCAAAGCTTTGATGGTGTAGCCAAAGTCCAGATAAGTCTTTGGCTTTGTATCTTCTAACAAGGAAACAATACTTAGGCTTTCAAGATCTGGTTGCGGTTATAGTTCTTATACTTAATCATACACATGACATATTGTCATATTTCAGTCCGAAACACTAACTAAGCTTTTTTTTGCTTCTCAACACTTTGATGGTGAAACCGAAGTCCGTATGAGTCTTTGGGTTTCTATCTTGTAATAAGGAAACATTATTTAGGCTTTCAAGATCCGGTTGCGATTCTAGTTCTGATACTCAATCATACACATGACATCTATTCATACTTGACTCCAAAACACTAACCAAGCTTCTTTTTACTTCTCAAAGCTTTGATGGTGTAGCTGAAGTCCGTATGATTCTTTGGCTTTGTATCTTTTAACAACGAAACATTACTTAGGCTTTTAAGATCCTGTTACGGTTCTAGTTATTATACTGAATCATACACATGACCTCTTGTCATATTTCACTCCAAATCACTAAGTAATCTTCTTATTGCTTCTCAAAGCTTTGATGGTGTAGCCGAAGTCTTTATGAGTCTTTGGATTTGTATCTTCTAACAAGGAAACACTACTTAGGCTTTTAAGATCCAGTTGTGGTGCTAGTTCTTATACACAATCATACACATGACATCTAGTCATATTTCATTCCAAAACACTAAACAAGCTTCTTCTTGCTTCTCAAAGCTTTGATGGTATAGTCGAAATCCGTATGAGTCTTTGGATTTGTATCTTCTAACAAGGAAACAGCTCTTAGGCTTTTAAGATTCGGTTGCGGTTCTAGTTCTTATACTCAATCATACACATGATATCTAATCATATTTGACTCCAAAACACTAACCAAGCTTCCTCTTGCTTCTGAAAGTTTTGATGGTGTAGGTGAAGTCCGTATGAGTGTTTGGTTTTGTATCTTCTAACAAGGAAACACAACTTAGGCTTTTAAGATCTGATTGCGGTTCTAGTTCTTATACTCAATCATAGACATGACATCTAGTCATATTTGACTGCAAAACAATAACCAAGCTACTTCTTGCTTCTCATAGTTTTGATGGTGTAGCCATAGTCCGTATGAGTGTTTGGTTTTGTATCTTCTAGCATGGAAACAAAACCTTAGCTTTTAGGATCTAGTTGTGGTTCTAGTTCTTATAATCAATCATACACATGACATCTAGTCATATTTGACTCTAAAAGACTAACAAGCTTCTTCTTGCTTTTCAAAGCTTTGATGGTGAAGCCAAAGTCCGTATGAGTCTTTGGCTTTGTATCTTCTAACAAGGAAACAATATTTAGGCTTTCAAGATCCGGTTGCGGTTCTAGTTCTTAGACTTAATCATAGACATGACATATAGTCATATTTATCTCCAAAACACTAACTAAGCTTTATTTTGCTTCTCAAAACTTTGATGGTGAAGCCGAAGTCCGTATGAGTCTTTTGCTTTCTATCTTCTAACAAGAAAACACTACTTAGGCTTTTAAGATCGGGTTGTGGTTTAAGTTGTTATACTCAATCATACACATGACATCAAGTCATATATGACTCCAAAACACTAACCAAGCTTCTTTGTGCTTCTCAAACCTTTGATGGGGTAGTCGAAGTCCTTATGACTCTTTGGATTTTTATCTTCTAACATGGAAACATCACATAGGATTTTAAGATTACTTTGTAGTTCTAGTTCTTATACTCAATCATACACATGACATCTAGTCATATTTGACTCCAAAACACTAACCCAGCTTCTTCTTATTTCTCAAAGCTTTGATAGTGGAGTTGAAGTCCGTATGAGTCTTTGGCTTTGTATCTTTGACAAGGAAACACTACATAGGCTTTTAAGATCCTGTTGCGGTTCTAGTTCTTATACTCAATCATACACATGACCTCTTGTCATATTTGACTCCAAAACACTACCCAAGCTTCTTCTTGCTTCTCAAAGCTTTGATAGTGTAGCTGAAGTCCGTATGAGTATTTGGCTTTGTATATTCTAACAAGGAAACATTACTTAGGCTTTTAAGATCCAGTTATTGTTCTAGTTCTTTTACTTAATCATACACATGAAACCTAGTCATATTTGACTCCAAAACACTAACAAAAGTTCTTCTTGCTTCTCAAAGCTTTGATGGTGTAGGCAAAGTCCGTATGAGTCTTTGGCTTTGTATCTTCTAACAAGGAAACACTACTTAGGCTTTCAAGATCGGGTTGCGGTTCTAGTTCTCATACTCATTCATACACATGACATCTACTCATATTTAACTCTAAAACACTAACCAAGCTTCTTCTTGCTTCCCAAAGCTTCGATGATGTAGCCGAAGTCCGTATGAGTCTTTGGCTTTATACCTTCTAGCATGGAAACACAACTTTAGCTTTTGGGATCCAGTTGTTGTTCTAGTTCTTATACGCAATCAAATACATGATATCTAGTCATATTTGACTCCAAAACTCTAACCAATATTCTTCTTGCTTCTCAAATCTTTGATGGTGTATCCGAAATCCGTATGAGTCTTTGGCTTTGTATCTTCTAACAAGGAAACACTACTTAGCTTTTAAGATCTGGTTGCGGTTCTAGTTCCGATACTCAATCATACACATGATATCAAGTCATATTCAACTCCAAAACACTAACCAAGCTTCTTCTTGCTTCTCAAAGCTTTGATGGTGTAGCCAAAGTCCGTATGAGTCTTTGGCTTTGTATCTTCTAACAAGGTAACACTACTTAGGCTTTTAAGATCAGGTTGCAGTTTAAGTTCTTATACTCAATCATACACATGACATCATGACATCAAGTCATATTCGACTCCAAAACACTAACCAAGCCTCTTCTTGCTTCTCAAAGCTTTGATGGTGAAGCCAAAGTTCGTATAAATATTTGGCTTTGTATCTTCGAACAAGGAAACACTCTTTAGGCTATTAAGATCAGTTGCGGTTCTAGTTCTTATACTCAATCATACACATGACATCTAGTCATATTTGACTCCAAATCACTAACCAAGCTTCTTCTTGCTTCTCAAAGCTTTGATGGTGTAGCCGAAATCCGTATGAGTCTTTGGCTTTGTATCTTCTAACAAGAAAACACTACTTAGGCTTTCAAGATCCGGTTGTGGTTCTAGTTCTTATACTCAATCATACACATGACATCTAGTCATATTTGACTCCAAATCACTAACCAAGCTTCTTCTTGCTTCTCGAGGCTTTGATGGTGAAGCTGAAGTCCGTATGAGTCTTTGCCTTTGTATCTTTGATGTAGGTCAAATTGCCTTAAGCCCACTCTTTCAAATAAGAGAACGATTGTAGCACTGAAGGGTCGAATTCCACAAAGCTCTCGGATCACACAATAGACCAAGGTTTTGAATCTACATATACATTTTTGGTAGGGTTTTTGGCCAAAAATCTTTCACCTATTTACTATTTGCAATATGAGTCCTTACAAATTTAATCTCAATCAAATACTTTATAACTAAAAACTTACAATTAGTTTAATTGCATTAATTAGTTGAATTAACTCTTTCCTAAATTTTTATTCTCAATTAAATCATTTTTATTGAACTATATTCGATTAAGGCTAATTTCCTAAAATATATATATCGACATTAAATTTAAATATCTAATTTTTTTGTCACTATATTTGTTTATATATATATATAATTTTGCTTTTAGTTTATATATAAAACACATATAGTTAATAAAATATTAGATTTTTTTTGCATCTGATGAAATTCAAATTTTTTCAAACAAACATATAATACTCCATCGATGAAAAAAAAAGTTTTAAAGCGACTAATAGTGAGTTATGAATAAAAAGCGACTAAATCCGGTAATCGTAAATCGATGCTAAAATAAATAGTCGTAAAAATAGTCGCCAAATAGCGAATATTGTACGACTAATATTCATAGTCGTAAGAGTAGTCATAAAATAGTCGTCAAATTTAGTGACTAATTGACTAATCTGCGATTAAATTTCACGAACATAAATTGTTTGTAGTGTTATAGTAGTAGAGTAGTCATTAAAAATTCGTTAAAATTTAGTGACTAATTTACAACTATATTGCGACTATGGTCGTATTTATGTACAAGAATACCATATTACAAAATAAAATTACAAACATTATAAAAAATAAGAATACATCATCCTCCTAAAAACATAACTAAAACAAAGTAGATAACAATCTTTAAGAAAGAACTTGGTTAAATTAATTCTTTTGATCTCAATTTTTCCTCCTTGTCTTACATCTTTCATGTTTTCTTCTTCTCCAAAAACTGCCTCATGGTTATTTCTACCAGAAGAAAATGCTGATATGCTATCATAGGTCTGGTCAAGATCATCTCCTTGCAAACAGAACACACCATGAGAAGGCAAGGACATAACATCTTAAGACGAACCTATCAACATGATTCTGGCCAAGCATTAAATCATGATTTGCCATTGCAGAAACAATAGCACACTGCTTAAAAAACAAACACCGTTAAAAAGACAAGTAGTTCTCACACTATAGCAATAAAAGAACAGTACATGTAACAAGAGAACATATCAACAAGTTCTATTTATCCAACTATAGAACACAAACCCACATAAGACCAAAGCATGAGAGACTATATCAATGTGAGCTTAAATTAAAAAAAAAAGTAAAGAACTTGGCGAGATAAGCCAAACAGTTCTTAAGGCACAATCATCATTTTCGCAACTTAATATCTTCGGACCTCACAGTTAAGAACTTGAATAGCTACACTATCATAAGAAACATATGTAAATTTCTTGGTCAGACAATATAGGCAACTTGGTTTTTAAAAGATCAGTTCAATTGATCTCAGCTGTTAAATAGTTTATCACTCTATAGCTAATACATTAAGAATGTTTAGAGAAAGATAAACACAGATTTGACTTTGTAGTGATCACTAACCTGAATTGGTCCCTTTTTTTTATGTTTTCTCTATGAAACAAGGTTTGACAAAAATTAACACCCCATTACTTTTTTAGAAAAACAAACTTCGACAATATCCAATCACAATGATAGCTTTGAAACCAGAAATTATAGACATATCAAGCTAAAATGATCAGCTCTTCTAATTATATTCAAAGAGAAGTGGATCAACATGAGAAAAAGCCATTATCAAAGTTCAGAAATCAAAACTCGTAAAAGACAAGACACCATTGATTATCAGATCTTTAAATTTAAAAGCAAAAACCATGACCCGTTACTTTGTCCAAAGTCTAAGTACATTCAAACTATGAGAGAAAGTAAACTCGTACCTTAAACCCAACTGAAACTGTTGGGACTTTTTTAAAACATCTCTTAGGCTGCGCTGGAAGAGGTCCATCCCTAGTCTACAAGAGAAATCGAGACAAAGTAAGCAAATATCAAAACTTACCAATCCAAGCAGTAGCAACAGATGCTAAAGAAGTTCCAACTGCATACACGACCATGTGCCTCTCGAACAAGTCCTAAGTGATTAAATATATTAGCGAGATATGCGGGCAGTTTGGAACAAGAAGTTGTTAAGATTGTTCCACAAGTTTTTGATAGTTGTAGATTTGATTTTTTCGGATCCTCTTAGAGATTCTTTTGTAATCTATTTAAAGCATTGTAAACACTTCTCTTGAATATATCAGAAAACTGAACATATTTTCCCAGTCACAATCTCTGAATTCGCATAGTAACAGAGCCTTTCTCTTAAAATTTTTGATTGGAAAATAGAGAAGTGATTCTCTACGACGATGGCAGCGATGAACCCATCAACGAACCCGACGACCACTGAAGTCCGACGAACCATATCCCCCTACGACAACCCCGGTGCTGTGATTTCCCACCCGTTGCTGCAAGCAACAAACTACGATGAGTGGGTCTGTAGCATGAAAACTGCATTGTGCTCTCGAAAGAAATTTGGATTTCTCGACGGCTCAATTGCACGACCTGCAGAAAACTCTCCCGATTTGGAGGATTGGTGGACCATTCAAGCGTTACTTGTCTCTTGGATAAAGATGTCTATTGATCCAAGTCTCCGATCTAACGTCTCACACCGTGATGTTGCGAAGGATCTGTGGGACCATCTAAAGAAACGATTCTCTGTCACTAATGGACTGAGAATTCAACAGATCAAAGCCGAGCTAGCATGCTGCAAACAAAGGGGTCTAGCCATTGAGACCTACTTTGGAAAGCTAACTCGCATTTGGGACAGTATGGCGAACTATCGTCCTTTGAGAGTTTGCAAGTGTCGTAAGTGTGAGTGTGATCTTGGCACACTTCAAGAGAAGGACATTGAAGAAGACAAGGTACATCAATTCCTTTTTGGGCTGGACGATACACATTTCCGAACAGTGAGGTCAAGCTTGGTTTTGAGAGTTCCGATCCAATCTATGGAGGAAGAATATAATATGGTACGACAAGAGGAAGATATGGTAAGAAATGGAATCAAAGGCGTTGAATAGAGCTCAGAAGTAAACGCATTTGCTGTCCACACCACTACACCATTTCAAAGAGATGAGAGAGATAAGTCAGTGTTTTGCAAACTCTGCAACAGAGCGGGTCATTCTAGCGACAGCTGCTATGGCGTGATAGGCTATCCAGAGTGGTGGGGAGATAGACCGAGATCTCGCTCTACGTCTAGCTGTGGTCGTGGACGTGGAGGCTCAAACAGAGGGGCTGGAAAGAGTCGTGGCAGCACCTCGTACGTCAACGCCGTCGGAGTTAATGAACCAACAGGTTATGCAGCCGCTAACTACGTCATCACCGACAATGACAGAGATGGAGTGACCGGTTTCTCCGATTCACAGTGGAAGGTGATTAAGAATCTGCTCAATGCTAACACTAGCAAAGTCTATGAATCCGAAAAACTGACGGGTACGTACTCTTTTCCCTCTTGGATAATGGATACAGGGGCTACACATCACTTGACGGGTCAGTTAGAAATCTTAACGGATGTTAAAGATATTCAACCAGTTGGAATTGTGTTAGCTGATGGGAGACAACGAGTGTCAGTTAAAGAAGGATCAGTGAGGCTTGGGCCGAATTGGACACTAAAATCAGTTTATTATGTTGATGGGTTTCAATCAAATTTGATTTCTCTTTGTCAATTGATGGATGAAAATCGTTGCGTAGTGCAGTTAGCTGATCATTTCCTTGTTGTTCAGGACCGCACTACGAGGACGGTGATTGGAGTTAGTAAGCAAAAGGGAGGGACCTTCCTTTTTTGGAGTTTGGAGTCTGTAACATCTGTTGAAGTTAGAGATGAGAAGTCATTCGAGTTGTGGCATCAACGGATGGGACATCCTTCAGCTAAAGTAGTTGGTCTTCTTCCTTTAATCTCAAATGTTATTTCTTCTGAAATTTTGAATAAAGCATGCGATATATGTCTCCGCGCTAAGCAAACCCGGTTGTCTTTTCCAATAAGTGAGAATAAAACGAAAAGGATTTTTGATCTTATTCACTGTGATTTATGGGGTCCATATCGAACTCCAACACACTCTGGTGCTCGTTATTTCTTAACTATCGTTGATGATTACTCTCGAGGAGTGTGGTTATATTTGCTTAACGAAAAATCTGAGGCACCAAATGTTTTGAAGAACTTCATAGCCATGACTGAACGACAATTCGAAACCCAAGTTTAAGTCATAAGAAGTGATAACGGCTCTGAATTTGTTAGTTTGAATGAGTTCTTCAAGAAACAAGAGATTGTTCATGAGACATCATGCGTCGGCACTCCTCAGCAGAATGGACGGGTTGAAAGAAAGCACAGACATATTCTAAACGTGGCCAGAGCTCTACAGTTTCAAGCTAACTTGCCCATTGAGTTTTGGGGGAATGTGTTCTCAGAGCGGCATATTTAATCAATCGAACACCGAGTATGGTCTGGAAAGGAATGACTCCTTATGAACGGATATACAATGCTAAACCGGTGTATGATCACATTCGAATTCTTGGGAACACGTGCTATGCTCACGACCAATCACATGGAGGCGATAAGTTCGCATCGCGAAGCCGTCGATGTGTATTCATGGGATATCCGTACGGGAAAAAGGGGTGGCAGTTATATGATCTAGAGAAGTGTCAATTCTTTGTCTCAAGAGATGTGGTGTTCTTTGAACATGAATTTCCATTTAAATCGGATGTTGCAGAAGAATTTGTGGAAGAAGCTGAAAATTTTAACATAGCTTCTTTGTACGTCGAGGAAGAAGAAACAGAGAGAAGATTGGGCCAGTTGGGCCAACTTAATGCTGAAAGCCCACTTGTTACAACTAATTCAGATGGGCTGCCCATAAGCCCAATTCGAAATAATGAAGAAGCTATTATTAGTGAAAATCAAAACGACGTTGCAGTTGAAACTTCACGTCACGTTCCTCTTCGTAACTTGCCATCATCTTCTTCCTCAGAGATTCCACGATCGACGACGTCACGACCTCCACCATTAACATCACCGCAAGTACAAACAAAGAAATCGTTTCCTACTGAACAATTGGGTCGAGGAAAGAGGCAGAGAACACATTCAGTAAAATTGAAGGAATTCGTGATTGAGACTGGAAATCCAAAAACAAAGAAGAACAAGTCCACATCTGAGTATCTGATTGCCAATTACGTTGACTGTGAGCGTTTCTCTGAGTCACATAAAGCATACATGGTTGCAATTACTCAGGGCGTAGAGGCAAAATCGTTTAAAAAGGCAATGGAAGACGATAGATTGAAAGGGGCAATGGGTTCATAAATTGATTCTCAGGAAAGAAATCATTCGTTGACCATAGAGGATTTGCCTAAAGGGAAAAAGGCAATAGGATGTAAATGGGTTTACAAGATCAAATACAAGTCTGATGGCACCTTGGAGAGACATAAAGCCAAACTGGTTGCTCTTGGAAACAAACAAGTTGAAGGAGTAGACTATGGATAAACTTTTGCGCCGGTAGCTAAAATGGGAACCGTGAGATTGTTCTTAGATACGGCAGTTAAGAACAAGTGGGATGTTCACCAGAAGGACGTCTACAATGTGTTTCTTCACGGCGACCTTGAAGAAGAAATTTATATGAAGCTACCATCAGGGTTTAAAGCAACCGATCCGAACAAGGTATGCAGACTTCGAAAGTCTATTTATGGATTGAAGCAGAGTCCGAGGTCCTGGTTTGCCAAATTGTCTACGGCGTTAAAAAATTTGAATTTGAGCAAAATCTCTCTGATTATTCATTGTTCACCAAGGATGCAAATGGAGAGTCACTTCACGTTTTAGTGTATGTCGACGATCTCACTTAAGGTCATGAATGATTTTAAAGATTACTTGTTGTCGTGCTTTCACATGAAGGACTTAGGCCAATTAAAATACTTCCTGGGAATTGAAGTAGCAAGAAACTCTACCGGTATGTATCTTTGTTAAAGAAAGTACGCATTGGACATTATCTCAGATGTTGGACTACTTGGTGCGAAACCTGTAGCGTTTCCTTTGGAAACAAACAATGACTTGTCATTGTCGAAGTCCGCGTTTTTAAACAACCCTGAACCTTATAGAAGATTGGTGGGTAGATTGATATACTTGGCTGTGACTCGACCCGATTTAGCGTTCTCTTTTCACTTTCTTGCTCTGTTTATGCAAAAGCCGAGAGAAGATCATTGGGGAGCAGCCTTGCGGGTGGTTCGATATTTGAAAAACAATCCTGGTCAGGGGATATTGTTACGAGCAGACACACCTTTTCAATTGTCCGGATGGTGTGATAGTGACTACGCAAGCTGCAGGCTATCAAGAAAATCCGTAACTGGATACTTCATATAGCTGGGAGGGTCACCTATCTCATGGAAGACGAAAAAGCAAAAGACAGTGAGTAGATCCTCGGAAGAAGCAGAATACCGAGCATTGGCATATTTGACTCAAGAATTGATTTGGTTAAAAACAATTCTTTCAGCGCTCGGAGTCAAACACAATCAACCGATGAATGTTCGCTGCGATAGCAAAGCCGCCATTCATATCGCAACAAACCCAGTGTTTCATGAGCGCACAAAACATGTGGAGGTCGATTGCCACTTTGTCCGAGAAGAGGTGATAACTAAAAACATTTGTCTCCTACATGTCAAGAGTGAGTCTCAGCTTGCTGATATTTTTACTAAACCATTGGGAAGACAAGAGTTTTATCGTTTTTGTTTCAAGTTGGGCATTACAGACCTCCATGCTCCAACTTGAGGGAGGGTATTAGCGAGATATGCAGGCAGTTTGGAACAAGAAGTTGTTAAGGTTGTTCCACAAGTTTTTTATAGTTGTAGATTTGATTTTATCGGATCCTCTTAAAGATTCTTTTGTAATCTATTTAAAGCATTGTAAACACTTCTCTTGAATATATCAGAAAACTGAACATATTTTCCCAGTCACGATCTCTGAATTCACAAAATAAAACTCCATTTAAGCAACTATACCCAAAATTTATAACATAACTCCTGATTCGCAAAACATTCCCAATCCCAATTCTTCAAAATACGATATAAATTGGAAAATTTGAAGTAAAAGACGCATTCAATACATACTCGTCTCCATACCAACCAACACATGCGATTCAAAACTCCCATTTAAACCCAATCACTGAGAGAAAAGTTTTAAAATTTAGAGATTACAAAAGGGTGGCCACTTAAGAGAAAAAAGTTAAGATTTAGAGAGCGTACCCAATCAAACATCCCTCCTCTGATGTTGGTGATGGCCAAATCCTTTACATTCCCTTTTCAAAACGGAAGAGGGCGCTAACAAGAGGATTGACGAACAAGCTTGAGATCGAGAATCTCCTTGACTTGAACCAATAAGATTGCAACTCCGCAGGACGAATCGAACATCAACAACAGAGAAAACCGATCTCTGTCCTTAAGAGGCTAGATCTGGGAACACGAATCAAGTGAGATAAACTCAGATCGACCCAAAGGGGATCAATAAATTGGAGCGCCATCATCCTTAAACATGAGCTAAGGAGATGTCTTGAGCTGCTGCTAATCGAGATCTCAAATCAGGAACTCTATATCCCTGCTAATCACCGCTAAAACAACAGATGATGGATTAAATAACTCCAAAGACACAAACGAAGAAGCTGCTAGGTTCAAATCCCCAACAAAGAACATAACATTGTTTAGAAGTGAAAGATTGAGAAGAGAAGAAGCGATAGAAATAGAGAGAGATTTGATAGTGTATTTCATTGATAAGCAACCCTAAATTTGAGAATCCGCTTAAGTCTCTGAGTTGCAATAGTAAACGACAACGTATTCAATGTAAAAGAGAAAGGACAAACCCTCTTTCTCAATTAGAGGTTTACTAATACTTCAAACGAATTTTCATAAGTTCGTTTTCAGTACAAGAAGAATCTTTTTCATTGCACAAAAAAAAGAAGAATCTTCTTCTTGCTTCATGTATATATTCTAAAGTAGCCACAAAGATTTAGAACATTTTATTGTCTATTATTGTTTCGCCCAAATTTTAGTTCTGACAATTTAATAGGGAGATGAAAAAAAATTAGATGCCAATTAAAATTTTTATGATTTAATTTTTAAGTTGAGCACAAAGTTAAAACTTTAGAATTTATAATATAGGGTTTTGGATTTAAAATCAGAAATTTGGAATTAGAATTCTAATTTTAAATATAATAATTGAATTTTTTTTAGGGATTTCAATTTATAAGTATAAAACGAATTAAAAAAACAATGGTAAAGTAAATATGTAAATAAATATAAATGTTTCTTTTATGTATATATAATGATTATGTTTTGGTTTATGAATTAATTTCTGTGATTCAGTGCTTAATCAAACCCGAGCCATGTTTGTAGTAGGTGACGGTCGTTGCCGTCTTCTTCGTTCCAAAATGTTGGACACACTCTTTCGCCGGTTGAGTCGCCATTGTTAGGATTTTATGATTTCCGTGCTTTTTCTGCAGCCAAGGAGATTATATGAGTAGAGAAAAAAAACATAATGCGTTAAGCTTTTATAGGTCAACGTGATGATGGACTGACTTTGAAGGCCCATAAATTAGGGTAACTATGACCTGTTACAATGTTTATGGGTACATAACTTAGATTAACCGACTACATCTTCTAATAAACCAACGGGTTTCGTCAATTTCGAAGATTCGCAAAAAACGGATGTATGTGGTCAACCACTAGATTTAGATTTCAAAGATGTGTTTTTTTTGTACCATTTTCGCTAGGAACTTGTAAGTATGCGAGCTAAACGACAACTTATGGTTAAAATGATCGGTTATATCTTTCATGTTTTTTGATCAAATGAGTTTCTAATTTTGTTACCAAAAATTTTGGTTTTTGATTGATATTCGGTTTATATGCGGTTAAATCGGATTGATTGCTGACAACCAAAAATAAATCAGATCGGTTAGATTTAATATAATTAACAAACAAACAAAAAAACAAAATAGGCCATTTGTGAAACAAAACCCAAATAAAAATTGGGCATTTTTTAATACAACTCAAACAAAGTTGGGCCTTTTTGCTTAGAGAAACAAAATCAAGGCCTCTTAATTTTTATTTATTTATTTATTTCAGATCTACATATATGTGTGTACCTTCTTCTTTGCGAGACATGTTAATAAAGTGAGGCTTACAGTTCCAGGGTAAAAAGTTTTAGAAATGTTCCGGGCAGCTTCAGCGATTCGATTTACATTTGTTGTGGTATTTAAAATTGATTTTGGTTTGGTTTACATTGGTTTAGAGAATGAAAACAGAAAAACAAAAAACTTGTGCGATGTGTTTTTGAGAGAGAAGTGCGTCGATTGAAAAAGGCATGATTTCGAAAGGGGTACACCCATAGAATTGCTCTAACTCGCTCAACGCGCACTTGGTGAGAGGGGGTGTGAAATAGAGCCTTCTGATTGGGTGAAAAAATCTAAGAAATTGAAATAGTAGACATGTTGCTAGCTCCTCTTCTATGTTATTTAAAAAGTCAATTTTAAAGTTTGCATTCAAATGGTTCTCATAATATAAATACATATATATATTTATATATATGTATATGTTTTAAAGAGTTTATCTACACTATTAAAATAAGAAACATTTAAAGAGTTAATGATATTTTTCGTTGCAAAATTGATAATATAATTTTATTAAAAACATTATTATTTTTATTATGAATTTAGTTTAGTTGCTAATAATTTTATATTTACATAATATATAAAGGAAAAAACAAATAAGATCTTGTATCTCTAAACATATTTCGATTTGAAAAAAAAAAAATATTGGATAGAATGTATGTCGCATATTTGTTTTGGCTATGTACTTAATCTGTTATAGTTTGTAAATGAGAAAATATCGGAATATATTTATAACATAATTATTATTACTTAGTTTTTATTATTAAAATATTTTGTATTTTTCAATTAATGGCATTTGATTTATGTTGATTGTTTTTTGTTTTATCTATAATAAATTGAAAAATACAAAATAGTTCGATTTATATTCGAAATAAATATATTGATGCTGAATATTTAGTTATTTAATATTTGATGTTTTGTTTAGTAGACAAATCATAGTAAAAATAGTGACGAAGCAAATCTCGGAATATATTGGATCATTTAATCAGGTTATTTGTTTTTGCAAAAAATTAATAAAGGATCCAAATCAAAAATATTTATTTAGTTGTATTAGTTGTTAATTTATCAGGTTATTTATTTATTTGTCAAAGATATCCTCACAGTTGTAGTTCACAAAATGTTTTTCAGTTAATAGTATAGATTTTGTGATAAAGAATCATTAGAGAGATAAGCATGGCCCTATCTTCAATGATTTTTGTTCATACATGTCTTTTCAATATGATAAATATCTTAAGAATCTCCCTTTTAATTATCTAACTAGGTCACCCGTGGGCATCTGTTACATCTCAATTCTGTGTATTTCACAAAGAATCTACTATATTTTCTTTATCTGTAAACATGTTGTATATTTAGTCTTGTCAAACCATATTTTGTTTAGTAAATATAGTTTTCTTGGTTTGCAGTATAAAAAATATATGTCTACAGTTGGTATGTACGTTTTGGAACGTAATTGTATATTTAGTATATGTAGGTGTACACATAGTATTTGTACAGTTTCGTGTATGTCATTAACATGTAAGCTAATTATAAAATTTGATAATTTAATACTTGGAAAATGTCTTTCTTCAAACTTCAAACTCATAATTAAAAAAAAAAAAGAATATTAATAAACTTCTCCACTAGTATTAAAAAAAGCTTGCATTAAGAAAATGGGAAAACGAAAAGCTTTTTAATAATTATTCCAACCTGCAAATTTAAAAACGACTAAATTAGCATGTTATATAATTTTGCAAAATGATATTTATTTTATAATAATCAAACTAAATAAATCCTGTAGAAATAATCTACTAAAGGTTTTGAAATATTTCTTTGAAAACAACATTTGCTGTTTCTTTCTTCTGTATTCCTTACCTTCTGTAATAAGAACCTTCAGCCCTGCCTTTGATTTCACTCTAGACATAGCGACATACAACTGTCCATGTGAGAAAACAGGCTTCGACAAATATATGCCAACATTGGCTAATGACTGGCCTTGGCTCTTATTGATCGTCACTGCAAATGTAACAGATAAAGGAAACTGCCTTCGTCGCATTTTAATGGGCAATCTACGATCCAAAGGAATTAGGGGCATCCTGGGAATAAGGACCGGTTTACCAACTCTAGTACCTTCTAATATACGTCCTTGCACCGGTTTATCTCCAAGTCTGATGACATAGCTCAAATTGCCTTAAGCTTACTCCCTCAAATAAGAGATCGTCGTTAGCACTTAAGGGTCGAATTCCACTGATCTCTCGATGTTCAAACAATAGACTTATGGTGTTATTAAATAAGCTAGACAAAGTAATTGAATGTAAAAGACAAATAAGCAAGTAAACGAAGTGTAGATTTAAGTGATTAAAGCGTCAGGTCTAAGGAATTATCTCGGGAGATAGATAAAGTGTAGATCTAGCTAATATAAGGATTGTTATCGCACTGTTCTCGAACTCAAACTATGATTCTAAAGTAACCGGTGGTGTTCCGCAAAACTCTCTATACTTAGAGCTAAGATAACTGAAGAAGCCGAGAAATCCTATACTCAAAACATACGAAAAAGATTAGAAAGACTCTAAAAACCATTCTGAAACTATGTTAGAAATCAATAAAAAAACCAGGACATATCAACTTCCCCAGAGTAAGCCTTTGCTTGCCCTCAAGTGATAACACTGTATTTTACTCATTTTTGGTGTGTGTTTATCATCATTTAGCTTTGCATTTGCTCATTGTATCATATCATTTTGCACCATTTTAGCTTATTTTGCACACTTAGGCATTTAGGATCATCTTGCATACATATTGCATGTTTTGGTGTGTTTTAAGGTGCAGAAGGAGCTTAAAAGCATTCTACTCAATCCATTCACTCGATCCAGGCAGCCATAGAGGAGGCAGCAATTCACTCGACCCTCTTACTCGATCAAGGCAACCTCAGAAGAAGCAGCAGATCAATCTACTCGCCCCTTCTACTCGATCCTCTATTCGACTCATTTACTCGACCAAGGGTTACCAACTCTACAAGACTTGTTGATCTATCAAAGCGTCTTCTTATCATCCTCATTCCCTACTCAACCAGACATTTGAGCATACGGATAGGAGAGGAAGAAGCATCTACTCGACCAATTGATCACTCGGAGCATAGACTCAAAGGCGCAAACTTTGCAGCACCCTCTTTACTCGATCCCCAAGATCGAGCACTCGCCCAAACCGCCTCAAGTCTCTTGTCTTCTAGTGAATATAGGGTTTTCAACATTTCACTATAAATAGATCACTTTGGGATTTGTCCAAAGGATCTAGCTTTTATCCTTTAGTTTTTCTTTGTGTTCTTGAATCTTCAAAACTTAATTACTCTTAATCTATTTTGTTTTTGCACTTAGTTCTTTTAGTAATGTTGTGTAGCTTGTTTAGATCTTCCTTTTTTATATGATTTTAGTAATTTCTGGGTTTATGTTCTTAGTTATGTTGCTTGAGTAGTGATTAGGGTTCTTGAGGATGGACTAGATTAGGGCAATCTCTAGGTTAGTCAGGTGGTTAAGATTAGGTTAGATCCTTTCTGGATTCGCTTGGCCCCTATGCGGTATTCGCTGGCTAGTGTGGAGATTCGATCATAGACGTCCAACGTCCAAAAGACGTTCAAATGGATATCTGATTTATGAATTCCAGAGATTTGTAAACTTGACCAAGACATTGGCTTGTCTTCTTATTGGCTTTGGTAGATTGTTGCTTAAACTCTGCTCTATTATGTTTCATAAACGATAGTTGAGATTAGGAAGTGGTAGAGTTATGGGGGTCTCTGTCACGACAGTGGATTGACCCGGTTTTTGAATCTATTCGTCTAGGGTATCTACCTTTCACCTGTTAGCCACCTGATGAATCCTAGAGTTGCCCTTATCAATTAATCCATCCTTAGGACTTCTATTCTTCTTAGATTTATTTGTATTCACCACCTGATTGCACTTTTATTTACCTGTTATTTGTATCCCATGTTGTTTGCATTTTAGTTCTTAATATTGTCACCTGTTGGTTCTACTCGACCCAATGATTGAGTAGGGCTCTTAAGTTCTCATTTTTACCTGTTTGAACCCTGACCTAGGATTGTTACTAGAAACCCACCATCATTATAATCTGGCTTGACTTAATTGCTTCTGTATACATCTTGATTGTTAACAATAATAGTGGACTGGATTGACACCCCCTATAGCTGCATCGACATGTCAGTGTTTAAGGGAAATTGAACTGAACCTCACATTCAACACATAAGTAGGCGTAAAAACCTACTTATCAACAAGCAAAACAAAAGATTGAATCTATGAAAGAGGTTTGAAAAACATACAAAACTCCCAATCTCTCTAGCTTATTTCCTTGATTATACAAACCAAACACATGCACTAAACAGTTCTAATCAGATCCTGACCACCATGTAATTCTTTGCTAGCCCACACAACATCCTAGATTCCATACCAAAGCACTCAACTATATCCACTAGGCTATCATCGCTGGGTCTCATCAATCAAGTTAATGTTTAAGAACCTTCTAGACTCATTCCTGTATTATACCATGACAAATCGACCATTCATTTTCTTCTTCTTTTTTTGGTATTCTTTCTCTCCCCTACACTTATTCTTTTAAGAGAGGAAACAAAGGGTAACTCGCATACTGGATTGACACCCTCGGCATTTTTCCAACCTTTGTAATTGATCATTCCAAATAATGTATAATAATGGGGTCATAAAAAACATTCCTAACCTTATACACTATTCGAAAATTCTATCAATCTCTCATATTTTTATTATATATTTTTTTATAAGGGGAGAGGAATACTTTCTTTTATCACATTAGCCGACTTATCAGGTATGAACAAGCAGTCAAGTCCTATGAACACTAACCGTTTTGATGAGGTAAAAGGAAGAGCAGTGAGATTAGCTCCAGCCTCTATGGATTATGTTGGGAATGGATGTCCTTAAGTTTGTTGATTGGCTTTCTGGATTTTCCATTAGTCGAATTGATTTGAATCTACAGTACTTAAACAATCCAGGCAATAAACTCGAGAGATAGATCATAATTTCAGACAAAAGTTTTAACTCAATAAACGACTATCTAAGTTAGAATGACACAACTGACTCATAAGAAGAGAAAAGATGAAGTTAGTAACTGACTCCCCTAGACATAGATAACACTGTCCCCAAAGTTATTAAGCTAGAGGAATCGAAAGAAAAATAATCAAAAGTTAAAAGTTAGAGAAACGTGAGTTGCCACCTGAGAGTGGTGTCGATCGATTCTAGGTTGGTGTCGATCGACACTCAGGTCATTTTTGCATCGCTTTGCGAATTCAGCTAATCGTTGTAGAGCTTTGGTTAGTGTTTTCACATGGATCCTCCAGTTAGACTGTTAAATCACTAAAACACACATCAATAGTTCCAATACAAATAACAAATAGTCTGAAAATAAAGCAAAAACAAAATCAGAAAACAAAGCAAGATAGGGAGGATGGCTAGTCCTCATGCTCCGAGCCGGAGGTGGGTGCGGGAGAGCGACGACAGTGACGAGTGGAAGCAACACAACCGCGACGAGAGAGTGTAGCTATGGCGTTCCAGACGCTCTTGAAGCTGTCGACGATGTACTGATGAAAGACCTATTGCTCTATCGGGATAGGAGGAGGAGAAGGCAGCTCATATGGTAGACCTAAAGAAGAAGATCCAACTCTCGGACCCCTAGTAGGGTCAATGAACTCATCCTCAGTCTGAGTCGGTGCGGATCTTGAAGCTGAACCTCTCTGTCTCCTGGAGCATGGCGCGGAATGCAGACGGGAAGGATCATGATGAAAACCAATCTCCTCATGCTCTCCTGTAATCTCTGTAAGTGCAGGGCAAGGTAACTGAATCATATGGTGTTGTGTCTCGTCTCTGAAACACCAAAGGAAGTTGCCTTTCAGCCAGTGAAAGTTCTTAAGATTACTCTCATCCATTGTGAATCTCGTGGTGTTGACCTCTTCTCCTTCAAAGCTGATGCAAAAATGCTCAAATATAGGAGTGAGAAGACTGCCAACTCTCTTAGATTTCTTCCCTTTACGTGTAAAGGGCTTGGTCTTGAGTGAGACCAAGTGATGAGCAAACACAGCGCCAAAGTTGACATCGTGGTCAACCTCCTCAACTCCAAAGCTGTCATGAAAGAAATCATGAACAGCATCGTTTCTTTGTTATTGTTTTTAAGCATGTTGTGTGAGTAGTGATTAGGATTTCTGAGGATGGGATAGAGTAGTTGTGGAACCCCTAGTCGAATATGTTGGTTAAGTGTAGATATCTTGAATCCCTTCTCGATTAGTTGTGTTTACTGCTTATTCGTTTCTGATCAACTGGAATCCGATCCCAAACGTTCCAGCGCCCAGAAGGCGTTTAATGAAACGTTGGATCTACTAATTCCAGTGATAATCGTCTCTATCCCAAGGGATTGGCCGTTTAGAGCGGTTATTAGCTTTAACAGTCGGTTCTTATTGCCTGTTTAGTTAGAGATTGCTAATGGGAATTAGGTCAGTGTCTAGCTATGCTTGAGGATTTCTATCACGAGAGTAAATTAATCTGTTGTTGAACCTCTTGTCTAGGGATAGCTTAATTGAGATTTGTTAACCAACTGAGTTGATTAGGGAGACCACACTATTCGATTACCCCATCCTTAGGAGTTTCTCATCCATTTGATTTCTCTGTTTACTTACCTGCTTCATTCACTTGCTATTGAATTTTAGTATTTTTCTGGTTTCAACCATTCTACTCGATCACACTAGTCTACTAACAGTGAGACTGCGTGATAGAGTAAAATGGTCAATTTCTGAATTTTCACTCTCTGTCATCCTGTTGTACACAATCACAGGGTCCCTTCTGGCAGTTGACCCTGCGATCGAGTATATCGGTCATTTCTGTGACTTTTTACTGTTTTCTGCAACAACACTAGGACTGTTAGAAAACCAACCAAATTCTGCTTGGCTTGACTTAGCAACTTCTGATCATATCTCATCTGTTTGCATCACACCCATTTGGATTGACACCTAAAATACTACAACGACAGGATAGTGTTTTAGGATTAATTGATCTGAAAAAACCTACTATCAGTTGTTGTGGAGGATACACCTTATCCTCAGGATTGGCAACGTTGGTGCTGCGGTAGGAGAGGTTGGAGTTGTTGGTTTTGAAGTTGTTGTATCCTTTGTAGCCACCTTGATTGTTGATGTAGCTGACTTCGTCTAACTGGTTCCCCTCCCCATCTTGGAATTGAGACTGCTCGTCATCAACAAGGAAGTGCACATGCTTCTTCTAACTAAGGAGAATTCTGTCCAGATTGTCATTCAGTGCTTTGATCTCCTTCCTGTGTTTGTCATCGGAGTCAGCTGTGCCTCTAACGGTCCTGTCGCAGTCCTCATTGTAATTACCATCTGATTGAGCCAGGTTCTCAACCAATTCCTAGCCTTCTTCAACATCCTTGTTCTGGAAATTCCCATTGCTGGCGGTATCCAGAAGCATCCTAATGCGTGGTAGAACTCCTCTATAAAGAGTGCTGAGCAGAGAGGCTTTCGCGAAGCCATGATGAGGGCATCGATTGGTGTAACCCTTGAAACCTTCCCATGCTTCACAGAAGCTTTCGCCAGTCTTCTGTGAAAAACCAGAAATCTGATTTCTGAGTCTTGCAGTTCTGGCGTTGGAGAAGAACTTTGATAGAAAAGCCTTCTTGCAATCATCCCAAGTGGTGATTGATTCATGGGGTAGATGCTTCTCCCAGATGTGTGTTTTGTCGCCCAAGGAGAATGGAAACAAACAGAGCTTCAATCCGTCTTCACAGACACCATTGATTTTTGTTAGGTTGCAGAGCCTATCGAATTCATCAAGGTGGTCGAGTGGATCCTCCATTGGCAGACCATGGAATTTGGTCCCCTTAATCATCGAGATGAGACCACTCTTAATCTCGAAGTTGTTGTTCTGGATAGCAGGAGGTGCAATTCCTTTCCTTTGACGATGGTTCCGTGGTGCATCTCTAATACCAATGTTGGCAGGGCCATTTTGTTGATTCTGTTCGTCGGCCATTTCGAATGGTTGTTGAGGTAAAAGGTTGACTGTGTTTCTCCTTTCTTTGATTTCGCTAGCTATGCGGTCGATGTTGTCGTTGAAAAGGAGGTTCTAATTGCCTGTTGATCGAGTTTGCATACAGCAAAGGTGTACCTGGATAAAGAAAACAACAAGAAAAACACACAGTTAGTAATCGAAAAGAAAATGGAAAAAGAACTAGATCTAAGCAAGTCTGAAATCTCAAATGTAACAAACAAACACCCAATTGGCAACGGCACCAAATTGATAATAGGTTTTTTAGTCAATTATCCTAAAACACTAATCATGTCGTTGTAGTATTTTAGGTTGTCAATCCAAATGGGTGTGATGCTAACAATCAAGATGTGATCAGAAGTCACTAAATCAAGCCAAGCAATAACAGGTTTTGGTGTGTTCTAGCAGTCCTAAGTGAACATGCAGAAAACAGAAAATCAAGTAGAAACAGTAAAACACTCGACCAACACAGCTCGTTGCAGAGCACTGGGTAAGGTCGAGTGACAGGTCGAGTAACAGGCAGTAAACGAAACAAAGACACTCGACTACACAGTCTGTTGCCAGACAACTGGGTGGTCGAGTATCAGGTCGAGTAACAGGAAAGAATTGTAGAAACTAAGCAACATATGACAAGATGCAGTAAACAACAGTAGAGTAAACAATAAAATCAATCAGATAAAGAATTCCCAAGATGGGGTAATTGAGTAGTTTCGTTTCCTCAGTTTACAAGTGATTGACATGCTCAGTAAATTATCCCTAGACAATAAGTTTATTAAGTAAATCTAAGTGCCACCGCAAAAGAGACCCTTAAGTTACCCTAGTCCCAGACTCAATCACCATTGACGAGAGCTAACCTGACAAGTATTACGAATCAACAAGTTAAAGCCAAAACGCTCCCTGCAACCAATACCTTGGTACAGGGCCACGAATCTCTGGAATTAGTGGTTCAGACATTTCATCGAACACCTTTTGGGCGCAGAAATGTCTGGGCTCAAATTCCTGTTGATTAGAAAGCAATAGGCATTAAGAACAACTAATCCAGAAGGGATCTATCAATCAAACTCAACCACTTAGCATACTGAGAATTCTAAACTACTCTAACCCATCCTCAGAAACCTATTCACTACTCAGACAACATTATAGAGAACATAAACGTTGAATTGAATGAAAACATGATAAACAACAGTGTAATAGCAAAATGATAACAGGATTAACAACAATAAACGAAATCAAGAACAAATATTGAATACTAAATAGATTAGAGAAATCCAGATTACAAAACATCAAGAACACAGTGTCTCCGGAATAAAACTTAGGTTAACGAAAATGTAAAGTACATGCTCCTTATAGCGAAATATTTCTGAAACCCCAATACTCAAAACGACGCAGTATTGGGAGGGATTAAGAACGGTACTCGACCCAGGTGGTCGAGTGAGTAGTGGAGTGATAAGCTGGAGTCTTCTTTTCTTCCTTGTGCTCGAGTGTCTAGTTGAGTGTGGAATGGAGAAGACTCTGAAGCTGGCCATCGAGTATGTAGTCGAGTGATGGTGATGGTGATACTCGACTTCCTGGTAGAGTGGTGTGATCGAGTGATGGTCAGGTGTGGAGGTGATGATACTCGACCAGGTGGTCGAGTGAAGTGATCGAGTGACTCTCATGGTGGTACTCGGCCAGGAGGTCGAGTGGTGAGGTGAATTGATGAAGTCTTCTACTCGACCAAGGGGTCGAGTGGTCTGTTGAGGTCGTGAAGCTTCTACTCGACCAGGGGGTCGAGTGGTTTGACATGATCATGTCCGCTTCTTCCAATTAGCTCGCCAACAGCTCCAAATCACCTGAAAAACACAAATATGCAACATGCATGCAAAACTATCCTAAACATGCAAAGTGATGAGAAATGATATATAACAGTGATGATATGATGCTAAAGTGATGACAAATGGATGCTCAAAACATGCAAAATACACACTTATCAGGTCGGCCCAGGAGGCAGGCGTATCCTTTGGTGGGTATCCTTTGGTGGCTTATCTTATAGCGGAATCCAGCATCCATGTTGAACCGTCTACAAAATCCACTAGGAAATCATACTCCTCCTTTTCTTCTATTAAAGCTATCTCCAGGGGATCCTCCAGAATGATTTCCCCTACACATTCTACCAGACGCCTCTTCTATCTCATCGAGATAGATAATCTGATCATGAGATGAAGCTTGCTTGAAAGTCTCATTCATATCAAACTTCATTATGAAATCGCCAAGGTGAAGGTCGATTTTTCAGTTCGGGACATCGATAATGGCGCTGGCGGTAGACAGGAATGGTCGGCCAAGGATCAGTGGTCCTTATGCTCTTTGTGAACTTCCAGGATAACAAAATTAATAGGGATTTGAGATCCCCCAATCATTATCGGAATATCAGCCAAAATCCCAACAGGATGGCGGATAGAACGATCTGCCAGTATTAGGGAGATTTTAGTTTGTTTGAAGGTGTAGTATCCAAGACGGTTAGTAACTGTGAGCGGCATCATGTTGATGCTTGATCCTCAATCACATAGGCATCACTCAAAAAGTTCATCATCAATAGCACAAGACAACACAAAACTCTCAGGATCACCTTTCTTTCATGTCGTCTGGTCTTGCAGGATGGCGCTACACTCTTGGGTCATCATCATTACCTCGTCTTCTAGGCTGATGTTGTTGGTTAAGATACTCTTCATTTATTTCCTAAGAGTGGGGATCATGGCACAAGCCTCAACAAAAGGTAGCCTTACCGTTAACTACCCGATAAGCTCTCGTTGCTTAGCTTTCTCCTTCTTGGTGCTCTTCCCAGCATTCGGGTATGGTACCTTAGGTCAGTAGATACGGTCCGAGGGAGAGATAAGATTGGTCGCAGTAGTGCTTTCGCGGCTTGGCGGTACTTCTTCCTCGGCTTCTTGGCTAGGAATGTGTATGGGAGGTTCTGGGTCTCTTCCACTATTCGGATCGTGACAGGTCTCACAGGCTTGCTTGGATTGGTTTTTCTTCCTCCTCCTGATCTTTCGGATACTCTGGTGTCTGTAAGCCATCCCACTCCTCAGTGTTATGGCTTGCAATGGTGCACCGATCAGTGGGTGCCCTTCTTCTGTTCTGAAGGATTTCTCGTATGACTCAATCAGGCAGGTAGTCTCATTTTCCTATTAAGACAAGTATTCGAACTTCCTCTAAAGGGAATTAATTTTGCTTTTTAAGTCCTCATGCATGATCTGGATTGAGGCGTCGACACTTTTTTGGCATTCTTGAAAACTAGCTTCGAGTCTCTTGATCAAAACCTCTAGGGTCGAGTTCTCATTTGCCGGTGTTGGAATAGGCAATTCTCACTGAGGTTCCTGAAACTCGCTAAGAATAGACCTTTGTTGAATATTACCTTGGTTTGAGATTTTCCCTCGGTTCCTAAAGCTCCGGTTTTGGGAGACTCTCGGGTGGCTCCTCATTGGCCCCATGTAATTTGCGGTTTCTTCTCGTCCGTAGAAATCATCAAGTTCCTCGGAGGAAGAGCTGAAAATCCCTTGAGAGTTCCGTACGGCGATACTCACAAGACTAGATTTCATCCTTGGAGGATTTTTCCAAGTTCCACATATTTTAAAAATATATTCATTTCGTTAAAATCTAAGTGTTGAGTTCGCTCTTCACAAGACTAGATTTCATCCCTCAAAGATTTTTTCAAGTTTCATCTCTTGGAAAAATATATTCATTTTGGTGAAATCTAAGTGTTGAGTTTGCTATTCACAAGACAAGATTTCATCCCTCAAAAAGTTTTCCAAGTTCCACCTCTTGGAAAAATATATTCATTTCAGTCAAATCTAAGTGTTGAGCTTGCTATTCACAAGACAAGATTTCATCCTTCAAGGATTTTTCCGAGTTCCACCTCTTTGAAAAATATATTCATTTCGGTGAACTCTATTTTTTGAGCTCGCTCTTCACAAGACTAGATTTCATCCCTCAAGGATTTTTCCAAGTTCCACCTCTTGGAAAAATATATTCATTTCGGTGAAATCTAAGTGTTGAGTTCGCTCTTCACAAGACTAGATTTCATCCCTCGAAGATTTTTCCAAGTTCCAGCTCTTGGAAAAATATATTCATTTCGATGAAATTTAAGTGTTGAGTTCGCTCTTCACAAGACTAGATTTCATGCCTCAAGGATTTTTCCAAGTTCCACCTCTCGGAAAAATATATATTATTTCGGTGAAATCTATGTGTTGAACTCGCTCATCACAAGACTAGATTTCACCCCTTAAGGATTTTTCCAAGTTCCACCTCTTGGAAAAATATAATCATTTCGGTGAAATCCAAGTGTTGAGTTCGCTCTTCACAAGATTAGATTTCATCCCTCAAGTAGTTTTCCAAGTTCCTTCTCTTAGAAAAATGTATTCATTTTGGTGAAATCTAAGTGTTCAGTTCGCTCTTCACAAGACTAGATTTCATCCCTCAAGGATTTTTCCAAGTTCCACCTCTTGGAAAAATATATTTACTGTGGTGAAATCTAAGTGTTGAGCTCGCTCTTCACATGACTAGATTTCATCCTTCAAGGATTTTTCCAAGTTATCCTTCAAGGATTTTTCGAAGTTACACTTTTTGGAAAAATATATTCATTTCTGTGAAATCTAAGTGTTGAGTTCGCTTTTCACAAGAGTAGATTTCATCCCTCAAGGATTTTTTCAAGTTCCACTTCTTGGAAAAATATTTTCATTACGATGAAATCTAAGTGTTGAGTTCGCTCTTCACAAGACTAGATTTCATCCCTCAAAGATTTTTTCAAGTTCCACCTCTTGGAAAATATATTCATTTCGGTGAAATCTAAGTGTTGAGCTCGCTCTCCACAATACTAGATTTCATCCCTCAAAATGTTTTCCAACTTCCACCTTTTGGAAAATTATATTTAATTCGGTGAAATCTAAGTGTTGAGCTCGCTCTTCACAAGTGTACGATTCAAGATGAATCGTCTTCAAGTTTAGCTCACTAAACACTCTAGACTCTCGTGTGTTTGTTTAAGAATGAAGAAACGACACCAAGCTTTAACTAGTTCAACTAACTGATGTGGTTAGTTTACGTCTCGCCGGAGATTCTCCGGAATCCACTAGATCGCTGATAACGTTCTCTCTCTACAAAGGATCTCTCTCTCTCTCTTATCTTGTTACAACAACTCAATGAACGATGAAACAGAATGAGCTAAAGATATAACGCTAATACTCCTTCGTTGACTTCTCACCTGTTAACCACACGTGTGATAATTAACCCCAAGACTTGAACCTTCAGTTTCTTTGAGAGACTCTCTGATACTTAGAGAATAAAGGCATTTGGGTCACAACTCTCAGTTCTCCACCTTGACCCCAATGCAGCGACCTAGCAGCCGAACTTGGACTCCAACTCCGTATACCTGACTAAACTCACTCAGCCTTCTAAGCCTTTTTGATTAAACCCACTAAGCTTCAGATCACGTGAGTAAACCCACTCAGCTTGTTGAGGAGCCTTATGGCTCCATTATGCTTCTGTAATCCTGAGTCGCTCTAACGCTTCTTGAAGCTTACCCACTGGTACTGTCTTAGTAAAGATATCTGCAGGATTACACTCTGTTGCAATCTTAACCAGATTTATAACCCCATCACTTACCAAATCTCTTATGAAATGATACTTGGTATCTATGTGTTTAGTCCTTTCATGGAACACTGTGTTCCTTGCCAAAGCAATCGCACTCTTAGAGTCACAGTGAATATTTACTGAATCCTGCTTAAACCCAAGTTCATTCATCAGTCCTAGTAACCAAACTGTTTCCTTAGCTGCCTCTGTTAGAGCCATGTATTCAGCCTCAGTAGACGATAATGCCACATCCTTTTGCAGACTTGATTTCCAGCTGATAACGTTGCCTCCTAAGTAAAAACTATACCTGATGTAGATCTTCTCCTGTCATGCAGTATAGTCTGAATCACAAAACCCTTCAATCACAAAATCTCCTTTGTTTTTATAGCAAAGCTTCTTCTTGATAGACCCACTGATATACCTTAGAATCCACTTAACTGCTTGCCAATGTTGCTTGAGTGGTTTACTCATGTACCTGCATACCAATCCCACAGGATATGCGAGATCTGGTCTGGTACCTATCATTGCATACATTAAGCTTCCAACTGCACTTTGATATGGCACACCCCTCATAGATTCAGACTGAATCCTTAATTCTTCATCTGTGGCAGCACGCAACTTGAAGTGTATTCCCATTGGTGTTCCTATTGGTTTGGATTGGTCCATCCCATAAGTTCCAAGGACCTTTAGGAGATATCCATCTTGAGATACAGTGAGAGTACCTTTCTGTCTATCTCTTGATATTTCCATTCCAAGGATCTTCTTAGCTTCTCCTAGATCTTTCATTTCAAATTCTGAGTTGAGAAGTGTTTTTAACTCATCTATATGATTCTTATCCTTTGAAGCTATTAAAATATCGTCGACATATAGCAGCATATAGACATAGTCTCCACTCTGTAATCTCTTAGAGTAAACGCAACTATCATATTGACTCCTTTCATATCCATGTGACTACACAAACTCGTTGAATCGATTATTCCATTGCCTTGGTGACTGCTTGAGACCATATAGCGACCTTTGTAGCAGACACACCTTCATTGGATATTTTTCATCTACATAACCTTCTGGCTGATCCATGTAAATGGTCTCTTATAAGTATCCATGCAAGTTAGCAGTCTTGACATCCATCTGTTGGAGTTCCATATCCAAATGAGCAATAATACTGAGCAGAAAACGAATTGACACATGTTTCACCACAGGTGAAAAAATCTCCTGATAGTCAACCCCTTCTTTCTGTGAGTAACCTTTAGCTACTACTCTTGCTTTAAATCGAGGACCTTCCACACCCACTATTCCAGGTTTTCTCTTGAAGACCCACTTGCAGCCTATAGGCTTCTGTTTTTCATCTCTGTCTACTAGTGACCAAGTTTTGTTCTTCATGAGAGACTCTATCTCTTCATCTGCAGCTGTGAACCACTTGTCACTATTAGCATCTCTTAATGCTTCCTGAAAACTGGAGGGCTCAGACTTGCCACCGTCTTCTGTTACGAAGTATGCGTATCCGGCTATCTCATCTAATTCTGCTTCTTCAGTGTATTAGTCTTCTAGTCTGGTTGGTCTCATGATCTGTCTTCTTGGCCTGTGTTTAGCTATCTGATAGCTCCCTGACATTTGATTCTCAACTCTTGTTGACATTTCCACTGAACTTTGTCCTGAATTTATCACAGTATCTTGCTTTAAAGCTCCACCTTAAGAAATATCCTCTTCAGACCTGTTATTTCCTGCAATATCACAAGTAGCTTTATCAGATAACATTGGTAAAGGGTTAGAGATCATACATGACTGTTTCTGAGCCATGATATCCTTGTACAACACATCCTCTTTGAACACCACATTTCTGCTAATGGTGCACTTCCTGTCTTCTAACAACCAAACCCTGAAGCCCTTAACTCCTTCTGGATATCCGGTAAAGATTCTTTTCTTAGCCCTCAAATTTAGCTTACCATCATTTGAGTGAATGTAAGCGAGACATCTAAATCTTCGCAAACCTGATAAGTCTGGCACAGTTGAAGTCCACATCTCTTCTGGAATGTGAAAATCAATAGCAGATGATGGTGTTCTATTTATTAGGTACACTAATGTGGAAGCTGCCTCAACCCAAAACTGTTGTCCTAATCCGCTCTCACTGAGCATGCTTCTGACTTTGTTCATAATCGTTCTATTTAATCTCTCTACAATTCCATTTTGTTGAGGTGTATAGGCACAGGTTCTGTGTCTCACCATTCCTTCCTTCTTGCAGAAAGTATCAAATCTATTATTGCAGAACTCAAGACCATTATCCGTACGAAGCTTCTTTACTTTCCTTTCACTTTGTGTTTCAACCATCTTCTTCCATTGAACAAACATGTCAAATGCTTCATCCTTTGTCCTCAAGAAATAGATCCAGACCTTCCTGGACCAATCATCAGTGAAGGAGATGAAGTATTGACACCTTCCAAGACTCAATGGGACGTTTGGAGAGCCCCATAAATTTGAATGAACATAGTCTAGTTTCTCTTTAGTGACGTGTTGTGCTGAGCTGAAGCTAACTTTGTGTGTTTTTCCAAACACACAATCCTCACAGAACTTAATACTTGATACTTTGTCTTTTCCAAAACACCCTTTCTCTGCTAGGCCATCCATCCCTTTCTGTCCCACATGCCTAAGACGATTGTGCCAAAGTTTTGTTAGATCTTCTTCTGCAGGTTTCTCTTCAAGTGCCAGACACTCTGACTTCTTAGCTCCTGCTTGTAAAATGTATTAAGACTCTCTTCTTGTGCCTTTCATGTACGTGATATCCCCTTTCATAACCTTTAACACACCGTTTTTGCCTTCGAACTCACATCCTTTAGAGTCCAGAGTTCCCATGGAGATCAGATTCCTTGACATCCCTGGCATATATCTGACATCGTGAAGCATAAAGGTGGTACCATCCGAGTTCACAAACCTCACTTTGCCAATACCATTGACCTCTGTGAATAAGTTGTTTGCCATTCTAACCATCCCTGTTCCAATTTCCTGAAACTCAATGAGCACATCTCTTCTTGGAGTCATGTGAAACGAGCAATTGTGTCCATTATCCACTCATCTTGATCTGCATTCTCAGTGAGATTCACTTCAGATGCGACTAGTCCAATCAGATCTTGTGCATCATCTTTAACCAGAGCTGATTCTCCTTGTTGAGAATTAGCCTTGTTCCTCTCAATCCACTTATAGCACTGTCTTTTAAAATGTCCCTCTTTACCGCAGATCCAACAGATTTTCTTGCCCTCTTTCCCAAACATTTGATCATTACCCTTTGCATGTGGTCTTCCTCACACATACAAGCCTTCACCAACCGGTCTTGAATCACCTAAGCTATCCCTCAGCTCCAGCTCCTTTGACTTAGCAACACTTATTACATCATCCAGCCTTATACCTTCTCTACCATATTTCGATGTTTTTTTCAACATATCATACTTCTCTGGAAGAGCACTCAGGATCAAGATTGCTTGAACTTCATTTGGGACTTGAATCTGAAGATTATTCAGATCTAAAATCATCTTTAAGAACTCATCAAAGTTTTCCTCAAGAGTCTTCGATTCTTGCATCATGTAGTTGTAAACCTTTAGTTGAAGATATACACGATTTGGTAAAATCTTTACCAAATACAATCTCTCAAGCGTTCCCCAAGCTTCCGCTGCAGATTTTGAATGTTTTCTTGATCTTAGAACCTTATCTGCGACATTGATGAATATAATGTCCATCGCCTTCTCATCTCGCTCTATTCTAGCCTCTTCCTCCTCGATCCACTTCTTCTTCTTTGCAGGATCTCCTTCTTCTTCTTCCGTGAGAGGTTTATGTGGTGGTTGCTCCACCAGAGCAGTCTTTAGCCCCAACACTCTGAGGTGAGCGAACATCCGAGTTCTCCAGAGGGAGAAGTCACCCTATCCATCGAATTGAGCAATCTTTACTTTCGAATCCGTCATTCTTATAACCGATCTTCAGATTATTGATGAACCAGAGCTCTGATACCAATTTGTATGATTCAAGATGAATCGTCTTCAAGTTTAGCTCACTAAACACTCTCGACTCTCGTGTTTTTGTTTAAGAATGAAGAAACGACACCAAGCTTTAACGAGTTCAACTAACCGATGTGGTTAGTCTACGTCTCGTCGGAGATTCTCCGGAATCCATTAGATCGCCGATAACGTTCTCTCTCTACAAAGGATCTCTCTCTCTCTCTCTCTCTCTTATCTTATTACAACAACTCTATGAACGATGAAACAGAATGAGCTAAAGATATAACGCTAATACTCCTGTGTTGACTTCTCACGTGTTAACCACACATGTGACAATTAACACGAAGACTTGAACCTTCTGTTTCTTCGAGAGACTCTCTGATACTTAGAGAATAAAGGCATTTGGGTCACAACACTCAACAAGACTAGATTTCATCATTCAAGGATTTTTCCAAATTCTAAATCTTTTAAAAATATATTCATTTCGGTGAAATCTAAGTGTTGAGTTTGCTCTTCACAAGACTAGATTTCATACCTCAAGATTTTTTTCAAGTTCCACCTCTTGGAAAAATATATCCATTTTGGTGAAATCTAACTGTTGAGTTTGCTATTCAGAAGACAAGAATTCTTCCCCAAAAAGTTTTCCAAGTTCCACCTCTTGGAAAAATATATTCACTTCATTTCGGTGAAATCTAAGCGTTGAGCTTGCTATTCACAAGACAAGATTTCATCCTTCAAGGATTTTTCCGAGTTCCACCTCTTTGAAAAATATATTCATTTCGGTGAACTCTAAGCGTTGAGCTCGCTCTTCACAAGACTAAATTTCATCACTTAAGGATTTTTCCAAGTTCCACCTCTCGAAAAATATATTTATTTCGGTGAAATCTAAGTGTTGAGTTCGCTGTTCACAAGACTATATTTCATCCCTCAAGGATTTTTCCAAGTTCCACCTCTTGGAAAAATATAATCATTTCGGTGAAATCCAATTATTGAGTTCGCTCTTCACAAGATTAGATTTCATCCCTCAAAGATTTTTCCAAGTTCCACCTTTTTGAAAAATGTATTATTTTGGTGAAATCTAAGTGTTGAGCTTGCACTTCACAAGACTAGATTTCATCCCTCAAAGATTTTTCCAAGTTCCACCTCTCGAAAAAATATATTCATTACGGTGAAATATAAGTGTTGAGCTCACTCTTCACAAGACAAGATTTCATCCCTCAAGAATTTTTCCGAGTCCCACCTCTTGGAAAAATATATTCATTTTGGTAAAATCTAAGTGTTGAGTTCGCTATTCACAAAATTATATTTCATCCCTCAAGGATTTTTTCAAGTTCCATCTCTTAGAAAAATGTATTCATTTCGGTGAAATCTAAGTGTTGAGCTCGCTCATCAAAAGACTAGATTTCACCCCTCAAGGATTTTTCCAAGTTCCACCTGTTGGAAAAATATATTTATTTCGGTAAAATCCAAGTGTTGAGTTCCGTCTTCACAAGATTAGATTTCATACCTCAAGGATGTTTCCAAGTTCCACCTCTTGGAAAAATGTATTCATTTTGGTGAAATCTAAGTGTTGAGCTCGCTCTTCACAAGACTAGATTTCATCCCTCAAGGATTTTTTCAAGTTACACCTTTTGGAAAAATATATTCATTTCGGTGAAATCTAAGTATTGAGTTGGCTTTTCACAAGACTAGATTGCATCCCTCAAGGATTTTTTCAAGTTCCATTTCTTGGAAAAATATATTAATTTCGGAGAAATCTAAATGTTGAGTTCGCGCAGTCACAAGACTAGATTTCTTCCGACAAGGATTTTTCCAAGTTCCACCTCTTGGAAAAGTATATTCATTTTGGTGAAATCTAAGCGTTGAGTTCCTTCTTCACCACACCATATTTCATCCCTCAAGGATTTTCCAAGTTCCACCTCTTGGAAAAATATATTCATTTTGTTGAAATCTAAGTGTTGTGTTTGCTCTTCACAAAACTAGATTTCATCCCTCAAGGATTTTTCCAAGTTCCACCTCTGGGAAAAATATATCCATTTCAGTAAAATCTAAGTGTTGAGTTCCCTCTTTACAAGACTATATTTCATCCCTCAAAGATTTTTCCAAGTTCCACCTCTTGGAAAAATATATTCATTTCGGTGAAATCTAAGGGTTGAGCTCGCTCTTCACAAGACTAGATTTCATCCCTGAAAGATTTTTCCAAGTTCCACCTTTTGAGAAAATATATTCATTTCGGTGAAATCTAATTGTTGAGTTAGCTCTTCACAAAACTAGATTTCATCCCTCAAGGTTTTTCCAAGTTCCACCTCTTGGAGAAATATATTCATGTCGGTGAAAAATAAGTGTTGAGCTCGCTCTTAACAAGACTAGATTTCATCCTTCAAGGATTTTTTCAAGTTCCACATCTTTTAAAAGTATATTCATTTCAGTGAAATCTAAGTGTTGAGCTCGGTCTTCACAAGACTAGATTTTTTCCCTCAAGGATTTTTTTAAGTTCCACCTCTTGAAAAAATATATTCATTTTGGTGAAATCTAAGTGTTGAGTTCCCTCTTCACAAGATTAGATTTCATACATCAAGGATTTTTCCAAGTTCTACCTCTTGGAAAAATATATTTATTTTGGTGAAATCTAAGTTTTGAGTTTGCTCTTCACAAGAATATATTTCATCCCTCAAAGATTTTTTCAAGTTCCACCTCTTGGAAAAATTTATTCAGTTCGGTGAAATCTAAGTGTTGAGTTCGCTCTTCACAAGATTAGATTTCATCCCTCAAGGATTTTTCAAAGTTCCAGCTCTCGGAAAAATATATTGTTTTTGATCAAATCTAAGTGTTGAACTCGCTTATAACAAGACTAGATTTCACCCCTCAATGATTTTTCCTAGTTCCACCTCTTAGAAAAATATAATCGTTTCGGTGAAATCCAAGTATTGAGTGCGCACTTCACAAGATTAGATTTCATCCCTCAAGGATTTTTCCAAGTTCCACCTTTTTGAAAAATGTATTTATTTTGGTGAAATCTAAGTTTTGAGCTTGCTCTTCACCAGACTAGATTTCATCCCTCAAGGATTTTTCCAAGTTCCACCTCTCGAAAAAATATATTCATTACGGTGAAATATAAGTGTTGAGCTCGCTCTTCACAAGACAAGATTTCATCCCTCAAGAATTTTTCCGAGTCCCACCTCTTGGAAAAATATATTCATTTTGGTAAAATCTAAGTGTTGAGTTCGCTATTCACAAAATTATATTTCATCCCTCAAGGATTTTTCCAAATTCTATCTCTCAGAAAAATGTATTCATTTCGGTGAAATCTAAGTGTTGAGCTCGCTCATCACAAGACTACATTTCACCCCTCAAGGATTTTTCCAAGTTCCACCTCTTGGAAAAATCTATTCATTTCGGTGAAATCCAAGTGTTGAGTTCCCTCTTCACAAGATTAGATTTCATACCTCAAGGATTTTTCCAAGTTCCACCTCTTGGAAAAATATATTCATTTTGGTGAAATCTAAGTGTTCAGCTCGCACTTCACAAGACTAGATTTCATCCTTCAAGGATTTTTCCAAGTTCCACATCTTTTAAAAATATATTCATTTTGGTGAAATCTAAGTGTTGAGTTGGCTTTTCACAAGACTAGATTTCATCCTTTAAGGATTTTTTCAAGTTCCACCTCTTGGAAAAATATATTCATTTTTGTGAAATCTAAGTGTTGAGTTTGCTATTCACAAGACAAGATTTCATCCCTCAAAAAGTTTTTCAAGTTCCACCTCTCGGAAAAATATATTCATTTCGGTGAAATCTAAGCGTTGAGTTCGCTCTTCACAAGACTACATTTCATCCCTCAACGATTTTTCCAATTTCACCTCTTAAAAAAATATATTCATTTCAGCGAAATCTAAGTGTTGAGTTCGCTCTTCACAAGACTATATTTCATCCCTCAAAGATTTTTCCAAGTTCCACCTCTTGGAAAAATATATTCATTTCGGTGAAATCTAACTGTTAAGTTCGCTTTTCAGAAGACTAGATTTCATCCCTCAAGAATTTTTCCAAGTTCCACCTCTTGGAAAATATATTTATTTCGGTGAAATCTAAGTGTTGAACTCGCTTATCACAAGACTAGATTTTACCGCTTCAGGATTTTTCCAAGTTCCACCTCTTGGAAAAATATAATCATTTCGGTGAAATCCAAGTATTGAGTGCGCACTTCACAAGATTAGATTTCATCCCTCAAAGATTTTTCCAAGTTCCACCTTTTTGAAAAATGTATTTATTTTGGTGAAATCTAAGTGTTGAGCTTGCTCTTCACAAGACTAGATTTCATCCCTCAAGGATTTTTCCAACTTCCACCTCTCGAAAAAATATATTCATTACGGTGAAATATAAGTGTTGAGCTCGCTCTTCACAAGACAAGATTTCATCCCTCAAGAATTTTTTCGAGTCCCACCTCTTGGAAAAATATATTCATTTTGGTAAAATCTAAGTGTTGAGTTCGCTATTCACAAAATTATATTTCATCCCTCAAGGATTTTTCCAAATTCTATCTCTCAGAAAAATGTATTCATTTCGGTGAAATCTAAGTGTTGAGCTCGCTCATCACAAGACTACATTTCACCCCTCAAGGATTTTTCCAAGTTCCACCTCTTGGAAAAATCTATTCATTTCGGTGAAATCCAAGTGTTGAGTTCCCTCTTCACAAGATTAGATTTCATACCTCAAGGATTTTTCCAAGTTCCACCTCTTGGAAAAATATATTCATTTTGGTGAAATCTAAGTGTTCAGCTCGCACTTCACAAGACTAGATTTCATCCTTCAAGGATTTTCCAAGTTCCACATCTTTTTAAAATATATTCATTTTGGTGAAATCTAAGTGTTGAGTTGGCTTTTCACAAGACTAGATTTCATCCTTTAAGGATTTTTTCAAGTTCCACCTCTTGGAAAAATATATTCATTTTTGTGAAATCTAAGTGTTGAGTTTGCTATTCACAAGACAAGATTTCATCCCTCAAAAAGTTTTTCAAGTTCCACCTCTCGGAAAAATATATTCATTTCGGTGAAATCTAAGCGTTGAGTTCGCTCTTCACAAGACTACATTTCATCCCTCAACGATTTTTCCAATTTCACCTCTTAAAAAAATATATTCATTTCAGCGAAATCTAAGTGTTGAGTTCGCTCTTCACAAGACTATATTTCATCCCTCAAAGATTTTTCCAAGTTCCACCTCTTGGAAAAATATATTCATTTCGGTGAAATCTAACTGTTAAGTTCGCTTTTCAGAAGACTAGATTTCATCCCTCAAGAATTTTTCCAAGTTCCACCTCTTGGAAAATATATTTATTTCGGTGAAATCTAAATGTTGAACTCGCTTATCACAAGACTAGATTTTACCGCTTCAGGATTTTTCCAAGTTCCACCTCTTGGAAAAATATAATCATTTCGGTGAAATCCAAGTATTGAGTGCGCACTTCACAAGATTAGATTTCATCCCTCAAAGATTTTTCCAAGTTCCACCTTTTTGAAAAATGTATTTATTTTGGTGAAATCTAAGTGTTGAGCTTGCTCTTCACATGACTAGATTTCATCCCTCAAAGATTTTTCCAAGTTCCACCTCTCGAAAAAATATATTCATTATGGTGAAATATAAGTGTTGAGCTCGCTCTTCACAAGACAAGATTTCATACCTCAAGAATTTTTCCGAGTCCCACCTCTTGGAAAAATATATTCATTTTGGTGAAATCTAAGTGTTGAGTTCGCTATTCACAAAATTATATTTCATCCCTCAAGGATTTTTCCAAGTTCCATCTGTCAGAAAAATGTATTCATTTAGGTGAAATCTAAGTGTTGAGCTCGCTCATCAAAAGACTAGATTTCACCCCTCAAGGATTTTTCCAAGTTCCACCTCTTGGAAAAATATATTCATTTCGGTGAAATCCAAGTGTTGAGTTCCCTCTTCACAAGATTAGATTTCATACCTCAAGGATTTTTTCAAGTTCCACCTCTTGGAAAAATGTATTCGTTTTGGTGAAATCTAAGTGTTGAGCTCGCTCTTCACAAGACTAGATTTCATCCCTCAAGGATTTTTCCAAGTTACACCTTTTGGAAAAATATATTCATTTAGGTGAAATCTAAGTGTTGAGTTGGCTTTTCACAAGACTAGATTGCATCCCTCAAGGATTTTTCCAAGTTTCATTTCTTGGAAAAATATATTAATTTCGGAGAAATCTAAATGTTGAGTTCGCGCTTCACAAGACTAGATTTCATCCGTCAAGGATTTTTCCAAGTTCCACTTCTTGGAAACATTTTGGTGAAATCTAAGCGTTGAGTTCCTTCTTCACCAGACCATATTTCATCCCTCAAGGATTTTCCAAGTTCCATCTATTGGAAAAATATATTCATTTTGTTGAAATCTAAGTGTTGTGTTTGCTCTTCACAAAACTAGATTCCATCCCTCAAGGATTTTTCCAAGTTCCACCTCTCGGAAAAATATATCCATTTCGGTAAAATCTAAGTGTTGAGTTCGCTCTTCACAAGACTATATTTCATCCCTCAAAGATTTTTCCAAGTTCAACCTCTTGGAAAAATATATTCATTTCGGTGAAATTTAAGTGTTGAGCTCGCTCTTCACAAGACTAGATTTCATCCCTCAAAGATTTTTCCAAGTTCCACCTTTTGGAAAAATATATTCATTTCGGTGAAATCTAATTGTTGAGTTAGCTCTTCACAAAACTAGATTTTATCCCTCAAGGATATTTCCAAGTTCCACCTCTTGGAGAAATATATTCATGTCGGTGAAATCTAAGTGTTGAGCTCGCTCTTAACAAGAGTAGATTTCATCCTTCAAGGATTTTTTCAATTTCCACATCTTTTTAAAGTATATTCATTTCGGTGAAATCTAAGTGTTGAGTTCGGTCTTCACAAGACTAGATTTCATCCCTCAAGGATTTTTTTAAGTTCCACCTCTTGAAAAAATATATTCATTTTGGTGAAATCTAAGTGTTGAGTTTGCTATTCACAAGACAAGATTTGATCCCTCGAAAAGTTTTCCAAGTTCCACCTTTCGGAAATATATATTCATTTCGGTGAAATCTAAGTGTTGAGTTCGCTCTTCACAAGACTAGATTTCATCCCTCAAGAATTTTTCCAAGTTTCACCTCTTGAAAAAATATATTCATTTCAGCGATATCTAAGTGTTGAGTTTGCTCTTCACAAGAATATATTTCGTCCCTCAAAGATTTTTTCAAGTTCCACCTCTTGGAAATGTTTTTTCATTTCGGTGAAATCTAAGTGTTGAGTTCGCTATTCACAAGATTAGATTTCATGCCTCAAGGATTTTTCCAAGTTCCAGCTCTCGGAAAAAATATATTCATTTCGATCAAATCTAAGTGTTGAACTCGCTTATCACAAGACTAGATTTCACCCCTCAATGATTTATCCTAGTTCCACCTCTTAGAAAAATATAATCATTTCGGTGAAATCCAAGTATTGAGTTCGCTCTTCACAAGATTAGATTTCATCCCTCAAAGATTTTTCCAAGTTCCACCTTTTTGAAAAATGTATTATTTTGGTGAAATCTAAGTGTTGAGCTTGCTCTTCACAAGACTAGATTTCATCCCTCAAGGATTTTTCCAAGTTCCACCTCTTGAAAAATTATATTCATTATGGTGAAATATATGTGTTGAGCTCGCTCTTCACAAGACAAGATTTCTATCCCTCAAGAATTTTTCCGAGTCCCACCTCTTGGAAAAATATATTCATTTTGGTGAAATCTAAGTGTTGAGTTTGTTATTCACAAAATTATATTTCATCCCTCAAGAATTTTTCCAAGTTCCATCTCTCAGAAAAATGAATTCATTTCGGTGAAATCTAAGTGTTGAGCTCGCTCATCAAAAGACTAGATTTCACCCCTCAAGGATTTTTCCAAGTTCCACCTCTTGGAAAAATATATTAATTTCGGTGAAATCCAAGTGTTGAGTTCCCTCTTCACAAGATTAGATTTCATACCTCAAGGATTTTTCCAAGTTCCACCTCTTGGAAAAGTGTATTCATTTTGGTGAAATCTAAGTGTTGAGCTCGCTCTTCACAAGACTAGATTTCATCCCTCAAGGATTTTTCCAAGTTACACCTTTTGGAAAAATATATTTATTTCGGTGAAATCTAAGTGTTGAGTTGGCTTTTCACAAGACTAGATTGCATCCCTTAAGGATTTTTCCAAGTTCCATTGCTTGGAAAAATATATTAATTTCGGAGAAATCTAAATGTTGAGTTCGCGCTTCACAAAACTAGATTTCATCCCTCAAGGATTTTTCCAAGTTCCACCTCTTGAAAAAGTATATTCATTTTGGTGAAATCTAAGCGTTGAGTTCCTTCTTCACCAGACCATATTTCATCCCTCAAGGATTTTCCAAGTTCCACCTCTTAGAAAAATATATTCATTTTATTAAAATCTAAGTGTTGTGTTTGCTCTTCACAAAACTAGATTTCATCCCTCAAGGATTTTTCCAAGTTCCACCTCTCGGAAAAATATATCTATTTCGGTAAAATCTAAGTGTTGAGTTCGCTCTTCACAAGACTATATTTCATCCCTCAAAGATTTTCCCAAGTTCCAGCTCTTGGAAAAATATATTCATTTCGGTGAAATCTAAGTGTTGAGCTCGCTCTTCACAAGACTAGATTTCATCCCTCAAAGATTTTTCCAAGTTCCACCTCTTGGAAAAACATATTCATTTCGGTGAAATCTAATTGTTGAGTTACCTCTTCACAAAAATAGATTTCATCCCTCAAGGATTTTTCCAAGTTCCACCTCTTGAAAAATATATTCATTTTGGTGAAATCTAATTGTTGAGTTTGCTATTCACAAGATAAGATTTCATCCCTCAAAAGGTTTTCCAAGTTCCACCTCTTGGAAAAATAGATTCATTTCGGTGATATTTAAGTGTTGAGCTTGCTCTTCACAAGACAAGATTTCAACCTTCAAGGATTTTTCCGAGTTCCACCTTTTTGAAAAATATATTCATTTCGGTGAACTCTAAGTGTTGAGCTCACTCTTCACAAGACTAAATTTCATCTCTCAAGGATTTTTCCAAGTTCCACCTCTTAGAAAAATATAATCATTTCGGTGAAATCCAAGTTTTGAGTTCGCTTTTCACAAGATTAGATTTCATCCCTCAAGGATTTTTCCAAGTTCCACCTCTTGAAAAAATGTATTCATTTTGGTGAAATGTAAGTGTTGAGTTCGCTCTTCACAAGACTAGATTTTATCCCTCAAGGATTTTTCCAAGTTCCACCTCTTGCAAAAATATATTGATTTCGGTAAAATCTAAGTGTTGAGCTCGCTCTTCACAAGACTAGATTTCATCTTTCAAGTATTTTTCCAAGTTACACCTTTTTGGAAAAATATATTCATTTCGGTGAAATCGAAGTGTTGAGTTCGCTCTTCACAAGACTAGATTTCATCCCTCAAGGATTTTTCCAAGTTGCACTTCTTGGAAAAATATATTCATTTCGGTGAAATCTAAGTGTTGAGTTCGCGCTTCACAAGACTAGATTTCATCCGTCAAGGATTTTTCCAAGTTCCACCTCTTGAAAATTATATTCATTTTGGTGAAATCTAAGTGTTGAGTTTGCTCTTCACATGGCTAGATTTCATCCCTCAAAGATGCTTTTGATGTCCGACCATCGGAAAAATGTGCTCATGTCGATGAAATTTATGTATCGATTTAACTCGTCATGGACTCAAATTCCAGGTAAAGCGGTGGCGATTCACTTTTTTCGGAAGCAAGAAACTCGTAAAAGCAAAAAAAAAAAAAAAAAAAAGAGGACATCAACGTCTCATGATGTTCTGAGATTTCCCATTAGCTACACAAACTTCACCAAAGAACTCCTGGACCTCAACAAGACTACAGCTCGTGGTGGCGAAACAACAACCTTATCACCTCCAAAGTAGGAGCACAAGTCTAGGTAGAAGCAAGGTTGAGTCTACTAAGGTCAACTCAATCGATCAAGCCATGACACAATGATACAGAGAAGTTTTCTTGGCGTTTCTTCAGAAATCATTAGCTGATGACTTCAACAATTACAGCCATGACAAGACAAGATCATATTGGCTCGAAACATTTGGATCACATCATGTATGAAGCATCAAAGATAAGTTCAACTATGGTGTACACGTTTACTCCTTAACAGGAATGTCTCCATCTTAAGTTATGCAAAATGTTATGACGAAAAGCATAGATGTCTAAGACCAAACAGGTGGGGGCGTGTTCCAAATAATATAAAGCAAAGTCAAGCCTCTTCGGAGGAGAATTATACAGTATTATCACTTAACGTGATTGACGAATCATTGCTACAAGTTTGGAGTCCATATTTATATCATCACTCAAAATTCAAGGAGTCAAAATGCGAGATTTGTCAACAAGAAGAGATTCAAGTTTGAAAAAGACAACTTCGATGCTATACCACAACCTTCCAATGCTATTCACCTTCATGGAGATGTGAATTCCAATTGGTGGAATCTAAGTGTTGAGTTTGCTCTTCACAAAATTAGATTCCATCGAGAAGAGATTTCTCCAAGTTCTAGCTCTTGGAAAAATGTGTTCATTCGATGGAATTTAAAAGTTTTTCCATAGTTTACCTGCTTTATTCCGAGCAATCAATCGGATAGAAGTGGAAGACGAGAAGATTAGCTTCACTTCGCAAAAAAAAGAAAGCCGGCTATGCCAATTGTTATTGTCCCTTCCAGGAAACCAAAGTTAAAGGGCAACAAAGAGATCAAACCACTTGGATTAAACCCGTTTAACAATAATATGGAGACATCATCTGTAACACCCCTATCCCGCTATAAAAACATAAACGAAATAGGAGTGTCACGTTGAACGCCCGTAGTCGGCCAACCCAGGTTTCCGAAACGAATTCGTTCGCCCGAGTCGTCCAACCGCAAGAGTCCAGCCTCCCGCCCGTGGCCTAAGGCTTTTCAACCCGTACCGCGGACGCGAGTGGTGATAGTCCGGAAAAGACAAATATCAGTCAGTATACCCATATAAATTTTAGAAATCCGAGTCTTATATATATATATATATATATATATCATACGAGAAATGGTTAGCAAAACGTATATAGTTTTCAGATCCTCTTTGAAAACCATATATAATATACTTAGTAAGAATTTACAAAAAGCCGTAAAACCTACATTGGTTCGTCCTAAGTCACGAGCACCCTCTACAAGGTTGGTCAACTAAGTTTTCCTGAAAAAGATAAGTAGCGTCATAAGTAATCTAGCATTACTTAGTGAACCCCGGATCTATATAGTGTTATCGTAATACCCTTCCTTACCCCCTAAGCAGCAACAACACGTAACAATAATACTACCTAACAAGCAAGACATAAACAAAGATAACACGCAACATACAACACAAGCAAAATATAATGCACCATGCAGAATGCATGCAGACTCGAATTTCCTAATATGCAGCGGACAACTAATGCAAGCATGCTTCTATCAACCTAGGTAAGGCCTTCTACGAATCCATGATCTCCCAAGGACATGCCTCCATACTCTTAGACACAAAGTCTATAAGAGACATAACATTCCTTTAGACTATATGGTCCAAGGTTGGTGATTAGGTCGGCCAAGGCAAGCACGAGGTGGCCCAGACCAATCAAAACGTTAAAATTGTCGACCAACAGCCGCTCCGGGACACTAATCGGCCTCCGATTGGTTCCTTCCCTTTTTCGAATAAACATCATCCAAAAATGGATAAGCACGACCCTAAAAGAATAAGGATGACCGGAATATGGATAAGGTTTAGCCTTAACGGATAAACATGAGCCACCGTTAAATCCTGGCCATCCGTTTCCTCATTCTAACCAAACATACGATCTGTCACTGGACCGCAAGAACATTAATACGGCACACTCTGTTCCAATGTCTCATGAGGGTCCATGATGCAAATCAAGGCTTCCTTAGGACTATAACACGTTCCATGCCAGAATTAACCGAGTAAGCCAGCTATCGTCACTTATCCGCATTCTCCAATCAACCCTCGACCATTCACGACCCATACATATCCTATTGTCCAAATGGATTCCCCTCGACTTTCTAATGTTTCCTAGACCGAAACTAGGTTTCCTTAAGCTATCCTAGTTCTTAGGGCTAACCTAATGGTTTCGGGGTTTACCGGTAGTCGCATCGTAACTAAGCTTGGTTCGTCTGATGGAGAGTGATCCCTTTACCATGGACTTGATCCTATTCAATGCTCCTTACCCATGACTTGCCACACTAAATGGCCGAACTGATCTTGCCTTTTTTTTGCTTGATCTCGCATTCTACTCGACAGACTGTACGTCCGGCCGCATAGTCAAGGTTATGGGCATTACATCCTTCCCCCCCTTAAAAGAATTTGTCCTCCAATTCTAAGGACTAAGGAAGTAACTAGCCTTCAAGGCAAACCGATTCCTTTGTTGGTTCCATGAAGCTTTGTGTAGAAAGGATAGGTTTGGTCTCCTCCATTTTTTTATTGCAATATTTTCCTCCATTCCTAGGAAGTTGATGCATGCCTAACGGACTCCGCCACTTTCTTTTCTTGTATGTGATTTTTGTCGTCACCACATACGGTTTAGCTAACGATTTAATTTATCGGCTTCCTAGTACTACTAGGCTTGGTCCACAAATCCTAAGGTTCCATCCTAATTTTTATGGTTCTCTCCTAACTCTTTTGAATGGGCTTCAGACTTCCTATACTTTCTAGTTCCTATGGTTTGGTACTTGGTTAAGTACGAAACCAAACACACACATAAATACATTAATAACTTGAAAGTAAATCAAGCAATCACACAAAAGGATATTAGTATAAACATAATGTAACATAACATTAGTTAACAGCCCTAAGGGGAAAGGAGGTGATTTTGACTATCGTACACACCTGCTTATTCAAGCCTATAGATATCATCAAAAAGATATCACAATGTAACCGAAGGTTACTATTGGCTCCCTTGTAGTCGTCGCTATACATCAAAAAATGGGGTTAGTTTCCTAATATTTACTAAGGTTTATGACTCCTAAGGGAATCAGAGTAACCCTAAGCTACACCTCGAACATGTACAGCCTAGAAAAAGATCCTCCTCCGTGTCGGGAATCGGTGGGGATGATGGTCCTGCTACGTGAGATGGGGAAGTACTAGTCGGTGCAGGAATCTGACCGTCATCTCTCATTATCATACAAGTTGCCGTGTAATGCCCATACTCTCCACTGTAAAGACAAGCAATGGCCATGGGCATATACTGCGTGTATAACTAACAGTCCTCGGGTAATAAACATCCTCACCAAAGATGAAGCAGAACGGTATCTGGATACCCGATGTGGAGCCCCCGTCAAATGTCAGAATAGCGTCAGCCCTCATTTCAGCCGTGAAACAAAGTATGGGGGACATGCCCTCCCATAACAGTGCAAAAGGGCTATCGGGAGAGTGATCGATAGCCTCCGGAACGTATGGAGAAACCGGTGGATCCTCATCCTCATCCGGATCTGACTCATAGGATATCGATGAATACGGGTTAAGTGCAAAGGATGGTGTGGCAGAACTGCTCGTGGGAACACTCACATAGACATGGACCATCACGATCTCATGTCCCTTCAGAGCCAGCTCCTGCTCCACCGTCCTCACTCCCGTGATCACACATTCATCCTCACAATCCGAATCGATCATCATCCCATCCCCTATATGCACATCCTCCTGCTCTGACAGAGCAACTATCCCTGCAAGACCAAAATCAGCAACCGTATCACTCGATCTAGTCTCCGATATAACCCGGGAACACTCGGCCACATCCCTTCTCACACACCTACCCATGACTCTCTCACGAAGGTCATATCGCCTGGACGACAGACCCAAGGACCGTCCCTCCTCCTCCACAGACTCCCACCACGTGATCTCTTTAGCGGGATACTATAATACAAACAAAGAGGTTAAGACACGAAAATACAAGCAAAAGGGTAAGCCACCTATAATACTAACAACAAGGGTAAGCCAACCTAAACAAAAAGGGTTTGTTCTATAGGTCAATCTACCTAAACTCTAGCAAGTGACTGAACAAATTTCCTAATAGCTTTTTACGACACATTTACTCGATAAAACATTGAAAACCATGGTTTCTCGTAGCGTCACAAACCATGCTCTGATACCACCTTGTAACACCTCTATCCCGCTATAAAAATATAAATAAAATAGGAGTGTCACGTTGAACGCCCGCAGTCAGCCAACCCAGGTTTTCGAAACGAATTCGTTCGCCCGAGTCGTCCAACCGCATGAGTCCAGCCTCCCGCCCGTGGCCTAAGGCTTTTCAACCCGTACCACGGACGCGAGTGGTGATAGTCCGGAAAAGACAAATATCAGTCACTATAACCATATAAATTTTAGAAATCCGAGTCTTATATATATATATATAACAAAACGTATATATATATATATATATATCATACGAGAAATGGTTAGCAAAACGTATATAGTTTTCAGATCCTCTTTGAAAACCATACATAATACACTTAGTAAGAATTTACAAAAAGCCGTTAGACCTACATTGGTTCGTCCTAAGTCACGAGCACCCTCTACAAGGTTGGTCAACTAAGTTTTCCTGAAAAAGATAAGTAGCGTCATAAGTAATCTAGCATTACTTAGTGAACCCCGGATCTATATAGTGTTATCGTAATACCCTTCCTTACCCCCTAAGCAGCAACAACACGTAACAATAATACTACCTAACAAGCAAGACATAAACAAAGATAACACGCAACATACAACACAAGCAAAATATAATGCACCATGCAGAATGCATGCAGACTCGAATTTCCTAATATGCAGCGGACAACTAATGCAAGCATGCTTCTATCAACCTAGGTAAGGCCTTCTAGGAATCCATGATCTCCCAAGGACATGCCTCCATACTTTTAGACACAAAGTCTATAAGAGACATAACATTCCTTTAGACTACATCGTCATGTAGCGCCCAGTCGGTAGCGATCCAGCCCTAAGGCCTTCGTATGCCATAACGGAACGGAGTTACACGTGATGCACAACATACAGACGGGATTCTCATGATTCGACTAATGCTAGACATCTAATTAAAGACTAACTAACATGAAAACAGACTCACATACTATCATGCATTCATCATATATATATGCAAAACTCTACTAATCAAACAACTAATCACACTTAATTATTACTAATCACGGTTAGTCCCCCTAATCATGGTTTAACTAACCCTAATCATGCTTAAGAAATACTTACCCTAATTAATGAACACTAATCCTAATTAACAATTACTAAACCTAATTAACTATCCTAATCAAGGATTAAGCGAATAATTAAGCTAAGTAATCATAATTAACCTCCTAAGCTATATTAAGGAAATAAATGAATAAACTATCCTATTCACGTGCTACTTCACGTAACTCAAGACTCACTTGGATTTCCGAGTGGTTTGGCTTCTGAATGTTTTCTCCCGCGAACTAAGCTGTTCTAAGCGATTTTTGGGTCTGAGGAATCAAATGGGCGGAGGGGGTATTTATATGGTCCAAGGTCGGTGATTAGGTCGGCCAAGGCAAGCACGAGGTGTTTGGACCAATCAAAATGTGAAAAATGTCGACCAACGGCCGCTCCAAGACACTAATCGGCCTCCGATTGGTTCCTTCCATTTTTTCGGATAAACATCATCCAAAAATGGATAAGCACGACCCTAAAAGGATAAGGATGACCGGAATATGTATAAGCTTGAGCCTTAAAGGATAAACATGAGGCACGATTCAATCTTGGCCGTCCATTTCCTCATTCCGGCGAAACCTACGATCTGTCACCAGACCGCAAGAACATTAATACGGCACACTCCGTTCCAATGTCTCCTGAGGGTCCATGACGCAAATCAAGGCTTCCTTAGGACTATAACACGTTCCATGCCAGAATTAACCGAGTAAGCCAGCTATCGTCACTTATCCGCACTCTCCGATCAATCCCGTGACCATTCACGACCCATACACATATCTCACTGTCCAAATGGATTTCCCTCGACCTTCTAAGGTTTCCTAGACCGAAACTAAGGTTTCTTGAAGCTATCCTAGTTCTTAGGGCTAGCCTAATGGTTTTGGGGTTTACCGATAGTCGCATCGTAGCTAAACTTGGTCCGTCTCATGGTGTGTCATTCCTTTACCCATGGACTTGATCCAATTCAATGCTCCTTACCCATGACTTGCCACACTAAAAGGCCGAACTGATCTTGCCTTTTTTTGGCTAGATCTCGCATTCTACTCGACAGACTGTACGTCCGGCCACATAGTCAAGGTCAGGGGCATTACACCATCTCTTGGGAAAGAGAAGATCGAGACAACCATCTCTAACAACATGGTTGCGATGAAAAGGCATACGACAACATCCATAGAGTAAATTATTATACTACAAAAGACTGTACCGATTTCAAGAGGCATCTTCCTGAGCTACGGACAATTGGTGACCTCGCAAAACCTTGACATCGGAGAGTTCGTGAAAACTTACCACGAAGAAAAGGCGGAAGTCAAAAATTCATCACTAGCTAAGAAAATACCTAAAACATCAAATCCCGGAGTTTCTTTTGGTTCTAAGAAGAAGATTGAAGTAATCATGAATAGGTCTAAGTTTTGTAGAGATTCGACCACTACAATCAAAAAGTTTAAGGGAATGTTCTATTAAAGGCGAGCACAAGCGACAAACTCGAACTCGCTAGAGCAATCACAATGTTCAAGGAAGAAGAAATTCGTCACCTTGCAGAACCTGATGACGTTGATTTTGTCCTCACCTTAGACGTGGCTGACGTCGGGGTTTCAAGGATTGTGATTGATGTTGGAAGTTTGATAGATCTACTATTCTTGAATACGCTTAGAGAGAATGGAAAGTAGCGAGGCTGACATAGTTGGAGCCCCATGAATTTATCGGATCAAGGTTATCCCATCAATCTATCGTCAATACGTTAAATATTTAATCCCGAATGGTGTGAAAATTTTACAAGGAGATCAAGAATCTTCAAAATCTTGTTATTTAACGACTCATCGGATGGTTATCCAATTAACAAATCTCGTGAAGAAAGAAGTGATTTTAGGAATATCAAGTGGGCAAGTAGTGGATGACGTCATAATCAAAGTGACCATCGATTAGAAAGATCATGAACGAACAATTTGGAAGAAAGTAGCATCTAGTTACAACCAAATAACGATGAATGATATAATTCAAATTAATCCTAAGACCACTCTCTCTAATAAAGAGTTCAGTTGCAGCACTTAGGGAACGAATTCACAAAGTTCTCGGATCACGTAGTAGACTAATGGCTAATCGAATTAAGCTAAGTAAAACAGTAAAGAAAATAAAAGAAAGAAAAAGTAAGCAATACCAATCAATTGGGAGTTGTGAAAACAAGATAATAAAGGCGTCAGGCTTAGAGTATTCTCACGAAATAGATGATACTAGATCTAGAAATAGCTAGGTTATTATCGAACCGTTCTCGAATTCAAACTATGATTTTAGAGTAACCGGTGGTGTTCCGTAAAACTCTCTATACTCAGAGCTAAGATTACCAGAGAAGCCGAGAAATCCTATACTAAAGCATGCATTATTATCAAGTTTGATTAGTTCACCTAGTATCTAAACCAGAGCCCTTCTATGAGCCTACCTGTTCTTTCTTAAATGCTTAGGCTCATCTATGATAGGTCAAATAACAAGTACCTATGGTGGCATACCTATTATCTAATATCAAGTTCTAGTTAGCTACTCTAGAACTAGCATTAAGAACAATGAAGATGAAGAATCCTACAAATAACGTAGCAAGGGGGCTATCTACTAAATCATCTAAATCCCTAATGAGAAACCCTAAACCTAACAAGAAGATTACTCAAAAATGATCATAGAAGAACAAAACATGATTGAATAAGAAATCAATGAATAAAACAAGAGGAGAATAAGGTTTAGAAGGATCTTCTCTACAGGGAGCCTCCACAGGGTTTTGCTCCCAAAGTACAGAATTGAAAAAGGGAATAGCCTCCCAAAGCTTAGGGTCTAAACATTGACCTTAAAGCTATATAAATATGTCTTAAGAACGTGCTGGGCCTTCATTAAATATAGGAGAAAGTTTTGGGCCGAATTCAAAAATCTTCAAAACAACAATAAGTTGCGTCTTTCAATTGTCGTCAGGTATTGGTGTCGCTCGACACCAAGGGTGGTGTCGTTCGACACCTACGTGCATTTTCGTCTCTGGATTGCTTCTGCAGCTTAAGTTGTATGTTTTGCTCCAGAAAGCTCCATAATCTTCAAATGCATCTCAAAAATGTAAAGACCTGAAAAAGACTAGAAAAACCTCTAGAAATAACAATTAGACTCTAAAGCCTATACCTAAAACATAGTTAAAAACGGTAAAAATAGGGATATATCAATGAATCCTAACGATTAAGAAAAATACTTCGTTCAGCACCAACCGAGGTATATACTATTTTACGATCATGCCGTTTGAACTAAAAATGTACGTGTGACATACCAACGATTTGCAAACAAGACGATCAAGAATCATCTCGAAAAACAGTGGAAGTCTATATAGATGACATGCTCGTGAAATCGTCAAAAGAGAGCGGTCACATTTAAGACTTGGGAGAATGCTTTAATATCTTGAACAAGCTTCAGATGAAATTGAATCTTGCCAAGTAGACATTCTGAATCCCATCCGGAGAGTTTCTCGGTTACTTAGTAACAAAGAGAGGAACCGAAGCAAACCCAAGACAGATTAACGCTCTTTTCGAACATGCTAGCCCCGAGGAACATCAAAGATTTCTAAAGACTCTTTGGTAGGATTACCGCGTTAAACCAGTTAACATCTCAATCAGTCGATAAATGCCTTTCTTTCTATCAAATACTAAAAGGGAACAAAGAGTTGAGTTGGATGAAAGATGTGAAGATGCTTTCGGTCAACTAAAGACATATTTAAATGTTGCTCTAATTCTCTGAAGCCTGAAGCCGATGAAAAGTTGTACTTATACATCTCATTTTTGAATCATGCCGTTAATGGAGTAATAATTTCTGAAGACAGAAGAGAGTGAAAGCCGATTTGTTACATGAGCAAGTCATCAACCAGTCCAAAATTAAGTTACACTATAATGGAGAAACTTGCGCTTGATGTAGTCACTTCAACATGGAAGCTGCGACTTTATCTATGTCTCATCCGATCATAGTTGAAACTCTTGGGAAAATTGGTTAATTTCTGCGGAGTTCAAGTGTCCAATTCGCTCTTCACGAAACTAGATCTCATCCCAAGAAGGTTTTTCCAAGTTTCAACTATTGGAAAAATAAGATTAATTTGGTGGACTATAAGTGTTGAGTTCGCTCTTCACTGAACTATCTCGTGAAGGGTTTTTCCAAGTTCCAACTCTTGGAAAAATAAAATTATCTCTATGAAATCGAAGTGTTGAAATCGCTCTTCAGAACTTAAGATACCACCTCACATCAACAAGCATTTATGACGAAGTCTTTGAGTACAAGCTCGGAAGAAGATCTTCAGTTTCTCTCTAGATTGCTGCTCAATAACCTGCCTTTGTATTCTATAAGCAGAATACACTTTGTAAACTCAAGCTTATAGCTCCGATTCGAAAGTCGAGGTCACTTCGCAACAACTTAACTCGAGGTCAGCTCCCATGCATAACTCGAGTTTAATTCTGATACCAAAACTCCATGATTGAACTGACGAGTTTGAGTCAATAAAATGGATTTAATCCTCTTGGACAAGGTGTCAACTAACAAAACAAAGATCTTTACGGTGTTTAGTCGATCAACCTAATACTTCTTAGGCGGAGCACAGTTCTCGCAATCAAAGAAAGTCCAAAGATTGAGTTTGGGGGTTTGATCAACCTATTTCTCTCTTAGCCACATGGCCCAAATTTCTTTTAGCCACATGGCCCAAATTTTTGAAAAAGTACGGACTTTGCAAATCCGGAAACTTAGAAGCAAATACTTTAAGCAAGTGTCATTCCAAAACCCCTATGGACAACGAAAAGGAAAAAGTTTTAAAAAACCCTCTTGGGTACACAACCAACAAATTTTAAAACCCCTTGGGCAAATAAAAATTCCCTTTCGGGAAAGTCGTATTCATAAAATAAATTGGGACAATCCCCAAATTTTATGGTAATAGCCATAAAAGAGTTAAGATGAGCTGAAGCCTCATCAACACTCGGTGTAATCTCGTCTTGGTTCAAAGTAGCAGTGGCCGTTCCTTGATCGTGAGTTTCAGTTTCATAATTAGGAGCTGAACATGAGCTGGATGAAGCATCGACATTAGTTAGGATACACATTTTGGCGAGTTGTCATTCAAGTTTATGAATTGATTGTGGAAGTTATTTTATGAATTAGTTCTATAATTAATGGTACATTGGAAGAAGGAAAGAGGCTATAAAAGAGAGGTGAAGACTCCAAGAAAAGGGGGGCTAGAGATTCTATATTTTGACCCCAAATCTTAAACTTAAGCATTGTTATTTTCTCATAAATACACAAAAGATTATTTCTTCTCCTTTTATGTAAGATCTTTACATAAGGAATACTATACCATTTTCTATGTTCAATTCTTCATACATTTTTATATACTCTCTTAAACTTTTATTATGCATATTTGTCGTGTACTTTTGTACTTGAGAGTTTTAGCCCACAAATAATATAAATACTTGAGTGTGTGAATCCGACATCCATAAAAGTCTGTATGAGTCTCTGGCTATGTATCTTCTAGCATGGAAACACAACTTTAGCTTTTAGGATCTGGTTGTGGTTCTAGTTCTTATAGTCAATCATAGACATGACATCTAGTCATATTTGACTCCAAAACACTAAACAAGCTTCTTCTTGCTTCTCAAAGCTTTGATGGTGACATCGAAGTCCGTATGAGTCTTTGGCTTTGTATCTTCTAACAAGGAAACATTACTTAGGCTTTTAAGATCCGGTTGCAGTTCTTGTTCTTATACTCAACCATACAAATGACATCTAGTCATATTTGACTCCAAAATACTAACCAAGCTTCTTCTTGCTTCCCAAAGCTTTGATCGTGAAGCCGAAGTCCGTATGAGTCTTTGGCTTTTTATCTTCTAACAAGAAACTAATACTTAAGCTTCCAAGATCCGGTTGCGGTTATAGTTCTTATACTCAATCATACACATGACATATAGTCATATTTCACTCCAAAACACTAACTAAGATTCTTTTTGCTTCTCAAAACTTTGATGGTGAAGCCGAAGTCTGTATGAGTCTTTGCCTTTCTATCTTCTAACAAGGAAACACTACTTACGCTTTCAAGATTCGCTTAAGGTTCTAGTTCTTATACTCAATCATACACATGACATCTAGTCATATTTGACTCCAAAGCACTAACCAAGCTTCTTCTTGCTTTTCAAAGATTAGATACGGAAGCCGAAGTTTGTATTAGTCTTTGGCTTTGTATCTTCTGAAAAGGAAACACTACTTACGCTTTTAAGATCCAGTTACTGTTCTAGTTCTTTTACTCAATCAGACACATGAAGTCTAGTCATATTTGACTCCAAAACACTAACCAAACTTCTTCTTGATTCTCAAAGCTTTGATGGTGTAGTCAAAGTCCGTAGGAGTCTTTGGCTTTGTATCTTCTAACAAGGAAACACTACTTAGGCTTTTAAGAGCCGGTTGCGGTTCTAGATCTTATACTCAATCATACAACTGACATCTAGTTATATTTGACTCCAAAATACTAACCAAGCTTCTTCTTGCTTCTCAAAGCTTTGATGGTGAAGCCGTTGGCTTTGTATCTTCTAACAAGGAAACAATACTTAGGCTTTCAAGATCTGGTTGCGGTTCTAGTTCTTATAATTAATCATACACATGACATATTGTCATATTTCACTCCAAAACACTAACTAAGCTTCTTTTTGCTTCTCAAAACTTTGATGGTGACACCAAAGTCCGTATGAGTCTTTGGCTTTCTATCTTGTAATAAGGAAACATTATTTAGGCTTTCAAGATCTGGTTGCGATTCTAGTTCTGATACTCAATCATACAGATGACATCTATTCATATCTGACTCCAAAACACTAACCAAGCTTCTTTTGAATTCTCAAAGCTTTGATGGTGTAGCCGAAGTCTGTATGATTCTTTGGCTTTGTATCTTTTAACAACGAAATATTACTTAGGCTTTTAAGATCCTTTTACGGTTCTAGTTCTTATACTCAATCACACACATGACCTCTTGTCATATTTGACTCCAAAACACTACCCAAGCTTCTTCTTGCTTCTCAAAGCTTTGATGGTGTAGCTGAAGTCCGTATGAGTACTTGGCTTTGTATCTTCTAACCAGGAAACACTACTTAGGCTTTTAAAATCCGTTTGCGGTGCTAGTTGTCATACTCAATCATACATATAACATCTAGTCATATTTGACTCCAAAACACTAAGTAATCTTTTTATTGCTTCTCAAAGCTTTGATGGTGTAGCCGAAGTCTGTATGAGTCTTTGGCTTTGTATCTTCTAACAAGGAAACACTACTTAGGCTTTTAAGATCCAGTTGTGGTGCTAGTTCTTATACAAAATCATACACATGACATCTAGTCATATTTCATTCCAAAACACTAAACAAGCTTCTTCTTGCTTCCCAAAGCTTTGATGGTATAGTCGAAATCCGTATGAGTCTTTGGATTTGTATCTTCTAACAAGGAAACAGTTCTTAGGCTTTTAAGATTCGGCTGCGTTCTAGTTCTTACACTCAATCATACACATGATATCTAGTCACATTTGACTCCAAAACACTAACCAAGCTTCCTCTTGCTTCTCAAAGTTTTGATGGTGTAGGTGAAGTCCGTATGAGTGTTTGGTTTTGTATCTTCTAACAAGAAAACACTACTTAGGCTTTTTAAGATCTGATTGCGGTTCTAGTTCTTATACTCAATCATAGACATGACATCTAGTCATATTTGACTGCAAAACAATAACCAAGCTACTTCTTGCTTCTCATAGCTTTGATGGTGTAGCCAAAGTCCGTATGAGTCTTTGGTTTTGTATCTTCTAGCATGGAAACAAAACTTTAGCTTTTAGGATCTGGTTGTGCTTCTAGTTCTTATAATCAATCATACACATGACATCTAGTCATATTTGACTCTAAAACACTAACAAGCTTCTTCTTGCTTTTCAAAGCTTTGATGGTGAAGCCGAAGTCCGTATGAGTCTTTGGCTTTGTATCTTCTAAGAAGGAAACAATATTTAGGCTTTCAAGATCCGGTTGCGTATTTCTTAGACTTAATCATATACATGACATATAGTCATATTTATCTCCAAAACACTAACTAAGCTTTATTTTGCTTCTCAAAACTTTGATGGTGAAGCTGAAGTCCGTATGAGCCTTTTGCTTTCTATCTTCTAACAAGGAAACACTACTTAGGCTTTCAAGATCCGATTGTGATTCTAGATCTTATACTCAATCATACACATGACATCTAGTCATATATGACTCCAAAACACTAACCAAGCTTCTTTGTGCTTCTCAAACCTTTGATGGTGTAGTCGAAGTCCTTATGACTCTTTGGATTTGTATCTTCTAACAAGAAAACATTACATAGGATTTTAAGATTAGTTTGCAGTTCTAGTTCTTATACTCAATCATACACATGACATCTAGTCATATTTGACTCCAAAACACTAACCAAGCTTCTTCTTACTTCTCAAAGCTTTGATGGTGGAGCTGAAGTCCGTAGGAGTCTGTGGCTGTGTATCTTCGACAAGGAAACACTACTTAGGCTTTTAAGATCCTGTTAGGGTTCTAGTTCTTATACTCAATCATACACATGACCTTTTGTCATATTTGACTCCAAAACACTACCCAACCTTCTTCTTGCTTCTCAAAGCTTTGATGGTGTAGCTGAAGTCCGTATGAGTATTTGGCTTTGTATATTCTAACAAGGAAACATTACTTAGGCTTTCAAGATTCAGTTATTGTTCTAGTTCTTTACTTAATCATAAACATGAAACCTAGTCATATTTGACTCCAAAACAGTAACAAAACTTCTTCTTGCTTCTCAAAGCTTTGATGGTGTAGGCAAAGTCCGTATGAGTCTTTGGCTTTGTATCTTCTAACAAGGAAACACTACTTAGGCTTTAAAGATCCGGTTGCGGTTCTAGTTCTTATACTCAATCATAAAAATGACATCTAGTCATATTTGACTCGAAAATACTAACCAGGCTTCTTCTCGCTTCTCAAAGCTTTGATAGTGAAGCTGAAGTCCGTATGAGTCTTTGGCTTTGTATCTTCTAACAAGGAAACTCAAGATCCGGTTGCGGTTCTAGTTCTTATACTCAATCATACACATGACATATACTCATATTTCACTCTAAAACACTAACTAGGCTTCTTTTTGCTTCTCAAAAATTTGATGGTGATGGCGAAGGCCGTATGAGTCTTTGGCTTTCTATCTTCTAACAAGGAAACAATACTTAGGCTTTCAAGATCCAGTTGCAATTCTAGTTTTTATACTCAATCATACACATGACATCTAGTCATATCTGACTCCAAAATACTAACCAAGCTTCTTCGTGCTTCTCAAAGCTTTGATGGTGTTGTCGAAGTCCGTAGGAGTCTTTGGATTGGTATCTTCTAACAAGGAAACATTACATAGGATTTTAAGATTAATTTGCGATTCTAGTTCTTATAGTCAATTATACACATGACATCTAGTCATATTTGACTCCAAAACACTAACCAAGCTTCTTCTTACTTCTCAAAGCTTTGATGGTGTAGCCCAAGTCCGTATGAGTCTTTGGCTTTGTATCTTCTAACAAGGAAACACTACTTAGGCTTTTAAGATTCGGTTGCGGTTCTAGTTCTTATACTCAATCATACAAATGACATCTAGTCATATTTGACTCCAAAATACTAACCAAGCTTCTTCTTGCCTCTTATAGCTTTGATGGTGTAGTCGAAGTCCGTATGAGTCTTTGGATGTGTAACTTCTAGCATGGCAACACAACTTTAGCGTTTTGGTTCCGGTTGCAGTTCTAGCTCTTATACTCAATCATTCCGATGTATTCCTATTTTTACTGTTTTTAGTTATGTTTTAGATATAGGATTTTAAGTCTTTTTGTTATGTTTAGAATGTTTTTAGAGTCTTTTCGGGTATTTTATGGAGAGGGACAACATTGGAGATGAGGAGACATTTTGGAGCAAAAGCTAAGGAAAAGAAGCTAAAGGACGAATTCACTTTCAGAATCAGTCGAGAGTGTCGATCAACACACGCTTGGTTTTGATCGACACCAATTGCGGCCCAGAGAAGTCCATCACGCTTTTAAGTTTGAAAAATTGGAAAATTGCCCCTGAAGACTTTCCTATTTTCATTATTGGTCTTGAAGGTTTTATAGGTCATATATATAGTTTTAGAGGTCATTGTTTAGACCTAAGAACTTTTTATTATCTTGTTTCACAACCTCTAATCTTCTATTTGCTTACTTTGTTTTCTTTTTACTTTCTTATATTCTCTTCTAGCTTAATCATGACACTTAGCTCAATGTGTGAACAAAAGGACTATGGAATCCGACCCTTAAATACTGCAATTGATTTCTTATTTGAGAGAGAGGTTTTAAGACAATTTAAACCATATCACATACACATGACATCTAGTCATATTTGACTCCAAAACACTAACGAAGCTTCTTCTTGCTTCTCAAAGCTTTGAAGACGTAGCCAAAATCTATATGAGTCTTTGGCATTGTATCTTCTAACAACGAAACACTACTTAGCTTTTAAGATCCCGTTGCAGTTCTAGTTCTTATACTCAATAATACACATGACATCTAGTAATATTTAACTCCAAAACACTAACCAAGCTTCTTCTTTGTTCTTAAAGCATTGATGGTGAAGCCAAAGTCCGTATGAGTCTTTGGTTTTGTATCTTCTAACTAGGAAACACTACTTAGGCTTTCAAGATCGGATTGCGGTTCTAGTTCTCATACTCATTCATACACATCACATCTACTCATATTTGACAATAAAACACTAACCAACCTTCTTCTTGCTTCCAAAAGCTTCGATGGTGTAGCCGAAGTCCGTATGAGTCTTTGGCTTTGTATCTTCTAGCATGGAAACACAACTTTAGCTTTAGGGATTCGGTTGCGGTTCTAGTTCTTATACGCAATCAAATACATGACATCTAGTCATATTTGACTCCAAAACACTAACCAATATTCTTCTTGCTTCTCAAATATTTGAAGGTGTAGCCGAAATCCGTATGAGTCTTTGGCTTTGTATCTTCTAACAAGGAAACACTACTTAGCTTTTAAGATCCGGTTGCTGTTCTAGTTTTTATACTCAATCATACACATGATATCAAGTCATATTCGACTCCAAAACACTAACCAAGCTTCTTCTTGCTTCTCAAAGCTTTGATGGTGTAGCCAAAGTCCATATGAGTCTTTGGCTTTGTATCTTATAACAAGGTAACACTACTTAGGCTTTTAAGATCAGGTTGCAGTTTAAGTTCTTATACTCAATCATACACATGACATCAAGTCATATTCGACTCCAAAACACTAACCAACCTTCTTCTTGCTTCTCAAAGCTTTGATGGTGAAGCCAAAGTTCGCATGAATATTTGGCTTTGTATCTTCTAACAAGGAAACACTACTTAGGCTTTCAAGATCCGGTTGCGGTTCTTGTTATCATACTGAGTCATACACATGATATCTACTCATATTTGACTCCAAAACACTTACCAAACTTCTTCTTCCTTTTCAAAGCTTCGATGGTGTAGCCGAAGTTCATATGAGTCTTTGGCTCTGTATCTTCTAGCATCGAAACACAACTTTTCCTCTTAAGATCTGATTGCGGTTCTAGTTCTTATACTTAATCATAAACATGAAATCTAGTCAGATTTGTCTCCAAACACTATTATGGCAGATCATGTTCACCAGGCAATGACCCAAAATAAGATCTTGGTCTATTTGAGATTGTTCCTTTTAATCGTAATCAAAGATGTTTTCTTGTTTCTTGTTTCTTTTCTGAACAAATTGAAGAACCTAATGGACCGAACACATCCTACGAAAGATATCGGAACACCAAAACCAGAAAGAATGAAAGTAGAGCTAATTGCGAGAGTTCCTCGTTAAAAGTGCAAGCTGTAATTCTACGTTTCAGAATCCCACTTAATAATTTCCTCAAAAAATGAAATCTGCTTTTCGTAAGACGTGTCTTTAGAATACTGTTCAAAACAAGTAAAAAATTTCAAGGAGTAAAGGATTTGATGGGAATTCCGGGTCGAAGAAGAGGCAGATCAAAATATGGTGCAAAAAAACCCAAATCGATATGAATGGAAGATGCCTCTGGAACTAGTATTGGTCAGTCGGTGGAACAATCACAACGTTACGCCCTAAAATATCAACTATCTCAATCCCCAAGTAATTGTTTAATATAAAGAGATTCGTCGCCCCGTCTGAGAAGGTCACGCTCTATTTTGCCTAGAAATAAGATCTCTAGGCGGTCCATTTCGGTCGGGCTAGATCGGGGGTTCGAAATGTCCAGCGCGTCCCGCCTCTCGTTCAAGAAGTCGAAAAAGCCTTTTGAGGAGAGTAGGCGAAGCTTCTTCTTGCCTCACAAAGATTTCATGGTCTAACCGAAATAAGTATTAGTCTTTGGCTTTGTATCTTCTAACAAGGAAACACTACTTTGCTTTTAAGATTTGGATGTGGTTCTAGTTCTTATACTCAATCATACACATGACATATACTCATATTTGACTTCAAAACACTAACCAAGCTTCTTCTTGCTTCTCAAAGCTTTGATGGTGTAGCGGAAATCCGTATGAGTCTTTGGCTTTGTATCTTCTAACAAGGAAACACTACTTATCTTTTAAGATCCAGTTGTGGTTCTAATTCTTATACTCAATCATACACATGAAATCTAGTCATATTTGACTCCAAAACACTAACCAAGCTTCTTCTTACTTTTCAAAGCTTTGCTGGTTTAGCCGAATTCCGTATGAGTCTTTGGCTTTGTATCTTCGAACAAGGAAACACTCCTTAGGCTATTAAGATCAGTTGCGGTTCTAGTTCTTATACTCAATCATACACATGACATCTAGTCATATTTGACTCCAAATCACTAATCAAGCTTCTTCTTACTTCTCAAAACTTTGATGGTGTTGTCAAAATCTGTATGAGACTTTAGATTTGTTTCTTCTAACAAGGAAACACTACATAGGCTTTCAAATCCGGTTGTGGTTCTAGTTCTTATACTGAATCATACACATGACATCTAGTCATATTTGACTCCAAATAACTAACCAAGCTTCTTCTTGCTTCTCGAGGCTTTGATGGTGAAGCTGAAGTCCGTATGAGTCTTTGCCTTTGTATCTTTGATATAGGTCAAATTGCCTTAAGCCCACTCTCTCAAATAAGAGAACGATTGCAGCACTTAAGGGTCGAATTCCACAAAGCTCTCGGATCACATAATAGACCAAGGTTTTGAATCTACATATACATTTTTGGTAGGGTTTTTGGCAAAAAATCCCTCACATATTTACTATTTTCAATATGAGTCCTTACTAATTTAATCTCAATCAAATACTTTATAACTAAAAACTTACAATTAGTTTAATTGCATTAATTAGTTGAATTAACTCTTTCCTAAATTTTTATTCTCAATGAAATCATTTTTTATTGAACTATATTCGATTGAGGCTAATTTCCTAAAATCTATATATCGACATTAAATATAAATATCTAATTTTTCTGTCACTATATTTTTTTATATATCTAATTTTGCTTTTAGTTTATATATAAAACACATATAGTTAATAAAATATTAAATTTTTTCGCATCTGATGAAATTTAAATTTTTTAAAACAAACATATAATACTCCATCGATGAAAAAAAAAGTTTTAAAGGTCCCACATTGCTCAAAAAAAAAAGAGGAAATCGTTAAGAGTCATACGAATTAGCAAGAACTTTAATTTTACTTCGGTCCTTTATTTTAAATAACTAAAAGTTTAAAAGTTTTCAAAAATATTTTATATATTTAAACTTTTAAAAAGTGAAACTCTATAACAAATATTTAAATATTAATGATTTTAAAATTTTATTAAGATTTAATTTCACCAGACGAGGTTAAAATGATGTTTAATTTCACGAGACAAGGTTATATGGTGGGATGGGTTTGGATCGATATTGATACGGGATGATATACTATGGACGAAATCCTAAAATTAACAATCAAAATACAATTATACTATTTTAAACGCTAATTTTTTTTTAATTAGTTATAAAAATCATATAGAGGTGTATCATAGTATCATACATAAAAAACAATAGCATGTATCTATAGATCATCTTTAATACAAAAATATTATTTTTTATTTAATAAAAATATAGGGTCTTTGATGTACCTCTTGCATATGTTATATATATAAATAATATTAATTAAAATAAAAATATCTAATTTTCTAACAAAATTATCGACCCCTGTGTGCTGCGGGTCAATATCTAAATTATACAAGTTCTAGATCTTTATTAACTCTAAAAAACCCAAACCAAAAAAATAAATACTGAATTTTAATATTAAACTAACAAATATGAGAAAACAAAACATGAATTGTCCAAGATTTACAAAGATAAAAATTATGTGTGAAGAATATATAATTTATATAGAATTAATAATTCCTATAAAAAATATCTATAACTTCTTAATGAACATATCCACCCGCGGTGTACCGCGGGTCAATATCTAGTTAAGCTAAGTAAAATAGTGATGAAAATAAAAAGAAACAGAAGAAAGCAATAGCAGATTGTGAGGTTGTAAGACAAGAAAGTAAAAACGTCAGGCTTAGGGTATTGTCAGAAAACTATTGGTTAGTAGATCTAATAATAGCTAGGTTGTTATCGAACCATTCTTAAACTCAAACTCTAATTTTTGAATAACCAGTGGCGTTTCGCGAAACTCCCAATGCCTATAGCTAAGAATAACCGGAGAAGCCGAGAGATCTTATACTTATACATGCATTCTAAACGAGTTCAATTGTTCACCTTAGTAGATAGGCCGATTCTTATTACACACCTATAACCGAGGCTCATCAAATAATTAAATCCAACTACAGATACTTATGGCGGGTATATCTATTGTTTGGATTCAAGTTCTAGGTGATCAAGCTAAAACTAGCATTAAGAACAATTAAAGATGAAGAATTCTAAAATACTCCATTATCTCCAAATGAATCCCAAACATGTTAAGACCTTAAGTGGATTAGAAAAGACTCTAGAAATAACAAATAGACTATAAAACATATACCTAAAACATAGTTAAAACATGTAAAAATAGGGATATATCAATCTTCTAACAAGTAAACACTATAGTGGTGGATCTAGAACATTTTTTTATATAGGACACCATAAATTATTTATAAATAACGATATAAATGAAATATGTAATATAAGATTTCTAAAGTGTTGCATGTACAACTTTTCATAAATTTTGTATTGATTTTGAAGTATATTTATGAAATCATAAGAATATTTAATTATTTTGTGAAAACAATTACTTGCACACATATAGCCATTAAGAAAAGAAAAGTAAACTAAATTTAGGAAAATAAACATACAATTAGTATCGATGATGATAGGATACTGAGATTATAGAAAAGGAAAAAAAAAGTTTTGTGTGTATCCACTTGTGTGTTCCATGATCTCATTCTTGCACCCGTTGTTAGTCTATCATGAATTGCAAGCCAAGTGATGAAAGAGAACTTCGGTGTAGCTTCCGAGAACCAAATCCCTTTCGCCCATCTGCAGATTGGTTTTTCAACTCTCAGCATTTTCCAAGTTTCATTTGAGGAGAAGTTGTGTTTAAAACCTGTCTTCCATTTCCATAGAATCAAATCTTCCTCTTCAACCTTCCTCCTCACCCTTGTTTCCCTGATGACCTCTTCCACTCTATTTAAAGTCTCCACTCTATGCCGTCTACCTCTGTGAGAGGCCAAAACGTCACCCACTGTGGCTTCTTTTGTAATACCCAAATCAATGCACCCTCGATCTCCAAAAGTATCAAATAAGTTCCCCAAAGAGGACCATGTATCATACCAAAAGGATGTCCCTTTGCCACTTTTTATTTCAACCTGATAGAAATCCTTTGCCAAGTCTCTAAGCTTTAAGAGTTTCCTCCACATCCAAGAACCTTGAGATGAATTTCCCTTGACTGTCCAAAACGTTTTTTTCCGAATTAAATAGGCATGAATCCACTTACTCCACAGAGACGTTGGTGCTGCAAATAAACGCCAAATTAGCTTCAGACCACAAACTTTGTTTGTCTCTTTGAGAGGTCGAAGACCTAGTCCTCCTTCTCTAATCGGCAAACAAACTACATCCCACGATATCTTAGCTTTAGAAGGATTTAAATCAGGCCCACTCCAGAGAAAAGCAGAACAAAGTTGTTGAATCTCTTTAATACAAGCGCCGGGTAAGCTGAATGTTGAAGACCAAAAGTTTGTGATGCTCATCAAGACCGCTCTTATCAACTGCAATCTCCCTGCATAAGATAGAAATCTACGCGTCCAAGAGCTTATTCTGGTTCTGATTTTCTCTACTAGAGGAAGATAATCATGAGCTGACATAGCTTTAGTCATCAAAGGTAACCCCAAATACCGTACTGGCAGCTTACCTGTTGCAAATGGAAAACTCCATTGAATGGCAGCTTGAGTAGTAGGGTCAATCCCTGCCATATAGAGAGTAGATTTCTCAATACTAATTCTCAAACCCGAGCATTTTGCATAACCATCAAAAACAGAGATAATTTCCTCGATCGACTTCTTTGTCCCTTCTGCAAAAACCATTAAGTCATCTGCAAAACACAAGTGCGTGAGAAGAACATTGCGACATTGTGGGTGATAACCAACCTTTCCCTCCATTGCCGCCTTATTGATCATTTGGGTTAACACATTCATGCAGATCACAAACAAGTAAGGTGATAAAGCACAACCCTGTCTTAACCCTCTCTTACTCCCAAAAAACCCCGGAAGCTCACCATTGATTTGAACTGAGAACGTAGCTGTTGATATGCAGAGATGGATCCAGTGAATGTATTTGGTCGGGAAGTGCATAGCCTTAAAAGTGTTCAGCAGAAAAGTCCATTGTACCGAATCGAAAGCTTTTGAAATATCAATCTTCATGGCACACCTAGGGGAGATGTCGTCTTTGTGATAGTTTTTAACAATCTCGGACGCCAACAAAACATTCTCTAGTAAGAGTCTGTTTTTTATGAACGCAGACTGATTTGGCGAGATAAGCTCCGGCAAGTAGAAGCTTCAACCTATTGGCCAATATCTTAGATATAACCTTGTAGAGAACGTTACAACAAGATATCGATCGATAATCTTTCATTATTTTGGCATCTTCTTTTTTTGGAATCAGAGCAAGGATCGTTGTGTTTATACCTTTTGGAAGGAAACCCTTAATGAAGAAAGATTGAATCGCAGTCGTGAAATCATCGCCAATGACTAACCATGCCGATTTAAAAAATTCACTCGTATAGCCGTCTGGACCGGGAGCTTTGTTCGCTGGCATTGCAAAGAGAACCTGCCTAACTTCTGCAGCAGTGACTTCCTTTTCAAGCATTATCTGTTGAGCCTCAGTACATTTGAAAGGTAGACAATGTTCCAAAGTCTCAGCAGTCATTTCTTCATAATCCAAAGGTACATGGCCTAAGAAATCCGAGAAAAAATGCTCTGCTTCCTTCTTTATTTCATCTTGACTAGATGCAATTGTTCCATCCTCACATTGTATTTCCCAGATTGTGTTTATTGACTCCCGTAACTTAGCTGCATTGAAAAACGTTTTGTTGTTTTTATCCCCCACGTCAAGCCAATGCAGCTTTGACTTTTGCTTTAGAAATGACTCTTCCAGCTCTGACAAATGAGACCACTTTCTATAAGCTTCTGTTTCGTCCTGTGATGCTGCATCAGTCGGACAAGACAAAGTTTCACTCTGTTTCTTACATAGATCTTCAAAAGCTGCTTTTACTCTCTTGTGCAAATCACCAAGTTTCTCCCTACTTAACTGTCTGATATGAGGTTTCAGATTTTTGAGCTTCTTGGCAAACCGGAATATTGCAGAAGTAGAGTGAAACAGGGTTGGTGTATCTTGCCAATAAGATTGCAAAAGGGGTTGAAACTCTGGCATCGTAGCGTTTGAATTTGTGAACTTAAACGGTTTCCTTTTTACCACTTTCTCTCTTTGGATTTGAATTCTGCAGCGGAGATGGTCTGAACAACCGCCTGGTTCAAAAACGCAATACCCTTGTGAGTAGTGGTTGAGCCATTCACTATTGATAAGAACCCTGTCAAGCTTCTTACAGATTACCCCCTCATCTCTCTTGTTACTCCAAGTGAATAACGGACCATGAGATCCTAAATCCATTAGTGAACAATCTCTGATAACCTCTTGAAAATCTCTCATACCTACTGGAATAGTAGGCATGGTTTCGAAATTTGAATGTTCTACTCCATCCAATATCTCGTTAAAATCACCCATAAGCAGCCATCTTTTATTTCTAAACAATGGCGAATCCTGGTGATTCCTCAAGTCTTCCCATAACACTTTCCTCTCTTCTGCTAAATTCTTTGCATAAATGAAAGAACAGAAAAATTCATCTTCTTCTCCCTCCATTAGTATAGAACATGTGATTAGCTGGTCACTTTTGAATATTGGAGTTAAACGCACGGTAGATCTCCAAAGAACCCAAATGCGACCATGCTGATTGAACTCATAGTTTGCCATAAAAGACCAATTGTTGAAAACTGTAGACACAATACTCGTTGCCTTCTTTTCCTTGACTCTCGTCTCTAATAAGCATCCAAATTGCATCTCATTATTGTTTGTCCAGTGACGGACCACAGAATGTTTAGAAGTTTTATTAAATCCGCGCACATTCCATGAAAAACACGACATTTAGTATGCTTTACGGTGTGACTTCTTGCCTGCAACTTTTGGAACTGAGTTCTTTGTTGCCTGATTCGAAGTCACCGTAACATCCTTCTTCTTTCTTGAGTGAGTACGTGTTGCATTTGGCTTATCATTTGTCTCTGTCTCCTCGACAAACTTTGTTTCTACATCATTTGCCTCTTCCGTAGCGCTATTCTTTACATTGTTTCCCACATTTTCAAATATTTCGCCTTCCTCCTCTTCATCCATACTCAAGACTGCATATTTGGAAGCTGACACCAATGTTGATTCCTCTACTTCTGCTCCTTTGCTCGGGCTACGACTTACTTTAGAAGGAGAAACCTGTAACCATTCACCCTGTTCCTTCATGTGAACATCCTGTTCCTTCACTATTACTATTTCTGATGCTGGAGCAGAGACAGTTTCATGAGTGGTGATTTGATTAGTAGATAAAATAGCGTTTTATACTTTTATGCTATAGTAAACAACCCTCATTTTTTCTATAACGTTTTAAAATTTGTAAACGCTATGTTTGGGTGATTAAAATTTTATTCTGAGTCACGCTTCTAGGTTAAACATATAATATAGCAGTTATCATTTATTAAACGCTGTGTAATGTTTAGAAAGAAACCAATTTTGCCCGCTAGTACTTAGCAAAATTTTGGCCACAAAAAATTGAGTACCCTCTCAATATTCCACACTACTCTCTCCAGCTCTCTCCATTGTTTCACGCATGAACCTTTTCTTAACAATTGCTTTAATCTTCAGCTCTCTTCCATTAATTCTAAAGAGATAATCGATACTAATTTGGGGTTTTCAGCAGATCTAGGTTTGGATACACATCTCTCTCTTCTTCCACCTCATAATTTCTTTCGACTGTTATTCATCTAATTTAGGTTTTTGACTGCAGGTTGCTTTGCTGAGAGATTGGTGTGCTCGAGTTAAGGAGAAATTTGTGTGAGCAATTGGTATGATTTTGTTTTGGTCGATTTTGATTTAGGGTTAAAATTTCCCTATTTGATTTTTTCAATTTGCTTTGACAGGGAGGAGATTTCGACAGAAGTAAAGCTATATAATAAGGTAACATGTTCTTTATGTTCGATTGAGACAAGTAATGTTTCGATTTTTGGAGTTGGAGTCAAGTTAATGGCTTCTTCTGTCTTCTGTTTTCTAGCTTTTTTTGTTTTGACAAGAATGTGTGATGGTTTCAAATTTGGATTTTGTTTTCAGTTTGTGCATTGATAGAGTTTTACAGAGGAAGCTTTCGGTCATTGATAGAGTTTCAAATCAAAGGTATCTCAAATTTTAAAGTATTTGTTCAGTTTGAAGCGAAGGGATTTGTTCTAGAGAATCTCAAATTGCTTCCTTGTTTCCTTGAAGTATCATATCCATCAATCAACATTCTTGTTACTTAGAGGTATATATATCCTTCATATTTCATTGAATTTTCTTAAAATATGTTTCATATTGTTATGTGATTGCGTCTAGTTATTATAGTGTCTAACTCTAGAAACTTTTATCACAAATGTATGTATCTTTTGGTCGTGGAGCTGTGATCTTATTGTAACATGGACAAGTCTTGGGTTTGGCTTTCAAGGTATGGAGTTGTATCTTAATCTTAGTTATGTTAGTTATGTTCTCCAGGACTAGCCTTGAGTATGAGCAAGGAGCCACAGATTTTGTGAAAGCATCAGCGAGGAAGCTGGGTAATCCTCCCAACATGTTTTGTCCATGTGTAGATTGCCGCAATGTGTGTCATCAACCAGGAGACACGGTACTTGATCATCTGGTAATTAGAGGTATGGATCACAAGTATAAGAGGAATTCATGCTGGAGTATTCATGGGGATTTAAGAAATGAGACAAATGAGATTCCGGGTTCTTCATTTGAAGCTTATGAGTTGTTTAGAACAGCTTTTCATGAGGAAGAGGAAAATTTACAGTCTCGGGAAAGACTATGGAGATCAAACAGAGGACAGTAAAGAAGATTCAGAGTTTAAGAAGAAAGTAAAACAAAGGAATAAGAAGAAAGAAGATAGGAAGTGCACGAGAGATAATTTGTCTGATTTTCTCATGTAACAATCGAATAATTATAACTATAGCATAAAACAATTAATAGTAGTGTGGGGCACGTGCCTATGTATGTAGTCTTGCACCTTGATCCGCCTTTGAAACATTATATACGCTTTTAAGATTCGGTTGCGATTCTAGTTTTTATACTCAATTATACATATGACATTTAGTCATATCTCACTCCTAAAATACTAACCAAACATTTTTTTGCTTCTCAAAGTTTTGATGCTGAAGCCGAAGTACGTATGAGTGTTTTGTATTGTATATTCTAACAACGAAACACTACTTAGGCTTTCAAGATCCGGTTGCGATTCTAGTTCTTATACTGAATCATACACATGACATCTAGTCATATCTGACTCCAAAACACTAAACAAACTTCTTCTTGCTTCTTAACGTTTTGATGGTATAGTTGAAGTCCGTATAAGTTTTAATAATGATACATTACTTAGGCTTTCAAGATCCGGTTGCGGTTCTAATTCTTATGTGCTACAAGAAAACTGGGTTTTAACGACTACACGATAAGTTGCCAAATAGTCGTCAAATAGTGAGTTATGACTAATAAGCGACTAAATCTGGTAATCGTAAATCGATGGTAAAATAAATAGTCGTAAAAATAGTCGCCAAATAGCAAATATTGTACGACTAATATTCATAGTCGTAAGAGTAGTCATAAAATAGTCGTCAAATTTAGTGACTAATTAACGACTAATCTGCGATTAAATTTCACGAACATAAATTGTTTGTAGTGTTATAGTAGTAGAGTAGTCGTTAAAATTCGTTAAAATTTAGTGACTAATTTACAACTATATTGCGACTATGGTCGTATTTATGTATTTATACAGTATTGTATTTATTTGTACAGTATTTATATATTTGTACAGTATTTATGTATTTATTTCAGTATTAATATTAATATTTATTATTAATTATTTCCAAACATATTTTTATTAATTATATTTAAAAGAATACCATATTACAAAATAAAATTACAAACATTATAAAACATAAGAATACATCATCCTCCTAAAAACATAATTAAAACAAAGTAGATAACAATCTTTAAGAAAGAACTAGGTCTGAACAAGCAGTCAAGTCCTATGAACACTAACCGTTTTGATGAGGTAAAAGGAAGAGCAGTGAGATTAGCTCCAGCCTCTGTGGATTATGTTGGGAATGGATGTCCTTAAGTTTGTTGATTGGCTTTCTGGATTTTCCATTAGTCGGATTGATTTGAATCTGTAGTACTTAAACCATCCATGCAATAAAGTCGAGAGATAGATCATAATTTCAGACAAAAGTTTGAACTCAATAAAACGACTATCTAAGTTAGAATGACACAACTAACTCATGAGAACAGAAAAGATGAAGTTAGTAACTGACTCCCCCAGACTTAGATAACACTGTCCCCAGAGTTATTAAGCTAGAGGAATCGAAAGAAAAATAATCAAAAGTTAAAAGTTAGAGAAACGTGAGTTGCCACCTGAGAGTGGTGTCGATCGATTATAGGTTGGTGTCGATCGACACTCAGGTCATTTTTGCATCGCTTTGCGAATTCAGCTCATCGTTGTAGAGCTTTGGTTAGTGTTTTCACATGGATCCTCCAGTTAGACTGTTAAATCACTAAAACACACATCAATAGTTCCAACAAAGCATAGGAAAATAAGTGCAACAAGTTCCAATACAAATAACAAATAGTCTGAAATAAAGCAAAAACAAAATCAGAAAACAAAGCAAGATAGGGAGGATGGCTAGTCCTCATGCTCCGAACGGGAGGTGGGTGCGGGAGAACGATGACGACGACGAGTGGAAGCAACACAACCGCAGCGAGAGAGTGTAGCTATGGCGTTCCAAACGCTCTTGAAGCTGTCGACGATGTATTGCTGAAAGACATATGGCTCTATCGGGATAGGAGTAGGAGAAGGCAGCTCATATGGTAGAGCTAAAGAAGAAGATCCAACTCTCGGACCCCCAACAGGGTCAATGAACTCATCCTCAGTCTTAGTCGGTGCGGATCCTTAAGCTGAACCTCTCTGTCTCCTGGAGCATGGCGCGGAATGCAGACGGGAAGGATCATGATGAAAACCAATCTCCTCATGCTCTCCTGTAATCTCTGTAAGTGCAGGGCAAGGTAACTGAATCATATGGTGTCGTGTTTGTCTCTGAAACACCAGAGGAAGTTGCCTTCCAGCCAGTGAAAGTTCTTAAGATATCTTTCATCCATTGTGAATCTCGTGGTGTTGACCTCTTCTCCTTCAAAGCTGATGCAAAAATGCTCAAATATAGGAGTGAGAAGACTGCCAACTCTCTTAGATTTCTTCCTTTTACGTGTAAAGGGCTTGGTCTTGAGTGAGACCAAGTGATGAGCAAACACAACGCCAAAGTTGACATCGCGGACAACCTCCTCAACTCCAAAGCTGTCAGGAAAGAAATCATGAACAGCATGATAGAGCAACAACAACATAGAAAGTCTCATCTTGCCAGGCTTCATCTTGCACAACAGTGTGCTAGTAATCAGCCTAACCAAATATCGCAGAAACCGGGTGACGAATCTCTGAGTGAACTTCGTCCTTGGAATCATAAATTCCATTACCAAACCTGCACTAGATAACCTGAGCATCCTTAAACTGTTCAAGCAGGGAAACTCCAGTGAGATCATTGTCAAAACCATAGATGTCACACAAATCTCTTGGTGGTAGACTCTATCTGACACCGCGAATGAAGTAGATAAGAGCTCCTTCCTGAGCATTGCGTTTCCTGTCATTCACATAGTAGACCCTAACAGAAACCATGAATTGTCTAACTAGCTCAGGATACAAATCGTACTACCTACAACAGATGGTTCCCAAACCTATATCATCTAGCAGTCCTAAAACAGATTTCTCTATCTGCAAACGCTGCATTAAACCAAAATCCGCAAACCAAGTAGGCAAAATTTCTACACTCATCTAGGCATTATAGAAAAAAGTATCATAATAATCCCAATCTGGAGAAAAAGGGCGATTGATATAGTCACCTTTAGTGAATTCTTTGTTGACATCCTTCTCCCAAACTCTTTGAGAAAGAATTCTTTCTGTTAGTAGCTTTGGCCATGGAAATCAATAGATAAGAGGTTGAGGCGCTGCAGCAGGTAGTGGCGGTTGGGAAGGGCGGAGACCGGCGGCGGTGGAGAAGGTCCGGCGGTGGTGAGCAGCGGCGGAGGAGGAAGGTGGAGGAACACTCTTCCTTGCCTTCTTTTTCTGCCCAGTCATGTTGCAGATCGATGAAATAGATACTTGGGGAAGAAAGAGGATGAAATTCTAATGAGATTAGAGCCTAAAATCACAAGAACCTGTGAACAATCTAGAGAGAAAAGAGTGATTTCGGCGTTTTGAATAAAAACTGAAATGAAGAAGATGAAGTTTAGGTGAAATCGACTTACCTTGAAAATGAGAGTGTCGATCAACACCATCGCGTTTCGTCGACACGATGTTTTTTCTTTTTTTCAAATGAGCATCCATTTGTTATCACTATTGCATCATATCACCACAGTTTTATACCATTTCTCATCATTTGTCATCACTTTGCATGTTTAGGATAGTTTTGCATGCATGTTGCATATTTGTGTTGTTTTCATCTGATTTAGAGCTATTGACGACCTAATTAGAAGAAGCGAACCTGATCATATCAAACCACTCGACCCCCTGGTCGAGTAGATGTTTCACGACTTCAACAGACCACTCGACCCCCTGGTCGAGTAGAAGACCTCATCACTTCACGTCACTACTCGACCCCATGATCGGGTACCACCAAAACAACCACTCAATCACATCACTCGACCCCCTGGTCGAGTATCATCACCTCCACCACCTGACCATCACTCGATCACATCACTCGACCACCTGGTCGAGTAACTCTATCACCTTCACTCGATCACTCTACCACCAAGTCGAGTACCACCATCTCTATCGCTCCCCTTCATACTCGACTGCCAGCTTCGGAGTCTTCTATATTCTGCATTCAACTAGACACTCGAGCAAAAGGAAGAAAAGAAGACTCCAGCTTACCACTCGACCATGCACTCGACCACTTGGGTCGAGTACCGTTCTTAATCCGTCCCAATACTGCGTCGTTTTGAGTATTAGGGTTATTTTGCTATAAGTAGCATGTACTTTACATTTTTGTTATCTAATTTTTATCCCGCAGACACTGTGTTCTTGACGTTTTTGTAATCCGGATTTCTCTTTATCTATTCAGTATTCAGTATTTGTTTTTCATTTCGCTTACTAAGTTGTTCATCCTGTTATCATCCTGCTATTACTCTGTTGTTATAATGTTTTCATTCCATTCAACGTTTATGCTTTCTTCAGTGATGTTTGAGTAGTGAATAGGTTTCTAAGGATGGGTTAGAGTAGTTTAGAATTCTTAGTATGTTAGGTGGTTGAGTTTGATTTATAGATCCCTTATGGATTAGTTGTTCTTAATGCCTATTGCTTTCTGATGAACTGGAATTTGAGTCCAAACATTTCCGCGCCCAAAAGGTGTTCGATGAAATGTCTGAACCACTAATTCTAGAGATTTGTGGCTCTGTACCAAGGTATTGGTTGCAGGGAGCATTTTGGCTTTAATTTGTTGATTCGTAATGCTTGTTAGGTTGGCTCTCGTCAATGGTGATTGAGTCAGGGACTAGTTTAACTTGAGTGTCTTTGTTGCGGTGGCACTTAGATTTGGTTAACGAACTTGTTGTCTAGGATAATTTATTGAGCATGTTAATCACCTGTAAACTGAGTAAACAAAACTACTCAATTACCCCATCCTCGGGAATTCTTTATCTAATTGATTTCTTTATTTACTCTGCTATTGTTTACTGCATCTTGTTACTTGTTGCTTAGTTTTTACATTTCTCTCCTGTTACTCGACCAGATACTCGACCACCTAGTTGTCGGGCAACAGACTGTGTAGTCGAGTATCTTTGTCAGTGTCTTTGCTGTTTACTCGACCTGTCACTCGACCACACCCAGTGCTTGGCAGCAAGCTGTGTTGGTCGAGTTTTTTGATTTATCTGTTTGAATTTATGTTTCCTGCATGTTCACTTAGGACTACTAGAACACCCGAAAACCTGTTATTGCTTGGCTTGACTCTGTGAACTCTGATTGCATCTTGATTCTTAGCATCACACCCATTTGGATTGACAACCTAAAATACTACAACGACATGATTGGTGTTTTAGGATAATTGACTAAAAAACAATTATCAATTTGGCGATGTTGCCAATTGGGTGTTTGTTTGTTACATTTGAGATTTCAGACTTGTTTAGATCAAGTTCTTTTTCAATTTTCATTTCGATTACTAACTGTGTGTTTTTATTGTTGTCTTCTTAATCCAGGTACACCTCTACTTTATGCAAACTCGATCAACAGGCAACCAGATTCTCCTTTACAACGATAGCATCGACCGCATAGCTCGTGAACTCAAAGAAAGGAGAAACACAATCAAACTTGTACCTCAGCAACCATTCGATATGGCTGACGAACAAAATCAACAGAATGGCCCTACAAACATTGGTGCTGGAGATGCACCAAGGGACCACCGTCAAAGGAAAGGAATTGCACCTCCTGCTATCCAGAACAACAACTTCGAGATCAAGAGTGGTCTCATCTAGATGATTCAGGGAAACAAATTCCATGGTCTGCCAATGGAGGATCCACTCGACCACTTGGATGAATTTGATATGCTCTGTAACCTAACAAAATTCAATGGTGTCAGTGAAGACGAATTCAAGCTCCAGTTGTTCCATTCTCTTTTGGAGACAAAGCACACATCTGGGAGAAGAATCTGCCCCATGACTCAATCACCACCTAGGATGATTGCAAGAATGCTTTTCTAGCAAAGTTTTTCTCCAACGCCAGAACTGCAAGACTCAGAAATGAGATTTCTGGTTTTTCACAGAAGACTAGTGAAAGCTTCTATGAAGCATGGGAGCGCTTCAAGGGTTACACCAACCAATTCCCTCATCATGGTTTCACGAAAGCCTCTCTGCTCAGCACTCTATACAGAGGAGTTTTACCACGCATCAGAATGCTTCTGGATACCGCCAGCAATGGGAATTTCCAGAACAAAGATGTTGAAGAAGGCTGGAAATTGGTTGAGAACCTAGCTCAATCAGATGGTAATTACAACGAGGACTGCGACAAGACCATCAGAGGCACAACCGACTCTGATGACAAACACAGGAAGGAGATCAAAGCGCTGAATGCCAAGCTGGACATGATTCTTCTTAGCCAGCAGAAGCATGTGCACTTCCTTGTTAAGGACGAGGATTATCAAGTCCAATATGGGGAGGGAAAGCAGTTAGAAGAAGTTAGCTACATCAACAATCAAGGTGGCGACAAAGGGTATAACAACTTCAAGACCAACAACCCCAACCTCTCTTACCGCAGCACCAACGTGTCTAACCCACAGAACCAAGTGTATCCACCACAGCAACAACAAGGTCAGAACAAACCTTTTGTTTACTACAATTAATGTTTTGTTCCCAAGCAGCAATTTCAAGGGAACTACCAGCAGCAACCACCACCTGGGTTTGCACCTCAGCAACACCAAAGTCCTTCTGCTCCTGACGCTGATATGAAACAAATGCTACAACAGCTGCTTCATGGACAACCATCTGGCTCCATGGAGATGGCCAAGAAGATATCTGAGTTACACAATAAGCTGGACTGTAGCTAGAATGATCTAAATGTCAAAGTGGAATCACTAAATACCAAAGTCCGTTACTTGGAGGGACATTCTGCATCTACTTCCGCTCAAAAACAGATAAGCCAACTTCTAGGAAAAGCAATTCAGAATCCAAACGAATATGCTCATGATATCACTCTGCGCAGTGGAAAAGCACTTCCAACCAGGGAGGAACCTAAAACAGTCAATGAGGACAGTGTAGATCAAAATGGGGAGGATTTCAGTCTCAGTGAAGATCAAGCTGACAAACAACTCGAGCAGTCACCCGACCATGTTACTCGACCACCATTCCCAGAAACATCACCAATTGCTCTGAAACATGTTGCTGTCAAGAACCAAGAAAAGGTCTTTGTTCCTCCTCCTTACAAACCCCAGCTTCCATTTTCGGGCTGTCACAAAAAAGCATTGACAGATAAGTATAGAGCTATGTTTGCAAAGAATATCAAGAAGGTTGAGTTGCGGATACCTCTTGTTAACGCTCTAGCACTAATCCCAGATTCTCACAAGTTTCTGAAAGACTTGATCGTAGAAAGAATTCAAGAAGTGCAAGGGATGGTAGTATTGAGTCATGAATGCAGTGCTGTCATACAGAAGAAGATCATTCCTAAGAAGCTTAGTGATCCTGGTTCATTCACTCTACCATGCTCTTTAGGTCCATTGGCTTTCAACAGATGCTTATGTGATTTAGGAGCGTCGGTCAGTCTCATGCCACTCTCTGTTGCCAAAAGATTGGGGTTCACTCAATACAAATCCTGCAACATATCCCTTATCATAGCTGACATGTCAGTAAGGATCCTGCATGGTTTACTCGAAAACCTACCTGTCAGGATTGGAGCTGTAGAGATACCAAGTGACTTTATAGTCCTGGAGATGGATGAAGAGCCCAAGAATCCTTTGATTCTGGGAGACCTTTCTTAGCAATTGTAGGAGCTATGATTGATGTCAAGAAAGGGATGATTGATCTAAACCTTGGCAAAGACTTTAGGATGACCTTTGACGTCAAAGACGAAATGAAGAAGCCTACCATAGAAGGGCAACTCTTTTGGATTGAAGAAATTGATCAGTTAGCTGATGAGTTACTGGAAGAGCTTGCAAAAGAAGATCATCTTAACGGTGCTTTAACCAGAAGTGGTGAAGATGGGTTTATGCATTTGAAAACTTTGGGATACCATAAGCTGTTATACTCCCATAAAGCGATGGAAGAATCAGAACCCTTTGAGGAATTGATTGGACCAGCAACAGAGGTAATGGTAATGAGCGAAGAAGGGTCAACACAAGTTCAACCTCCACACTCGAGAACATACTCGACCAATCACTCGACCTTGACTGTCAGCGACTCTAGGAACTGATCATCCCAACTTCACATAACTGGTCTGAACTCAAGGCGCCTAAGGTTGATCTCAAACCACTTCTTAAAAGTTTAAGGTACGCATTCCTTGGTCCAAACTCTACTTACCCCGTGATCATTAATGCTGAGTTAAATGATGATGAAGTGAACTTGCTATTATCTGAACTTAGAAAGTATAGGAGAGCAATTGGTTATTCATTATGTGACATTAAGGGAATTTCACCTAGTTTATGCAATCATAGGATCCACCTTGAAAACGAATCCTATTCTAGCATTGAACCAAAGAGGAGGTTAAATCCTAACTTGAAAGAAGTAGTGAAAAAAGAAATTTTGAAACTGCTTGATGCTGGTGTCATCTACCCTATCTCTGATAGTATTTGGGTTTCTCCAATGCATTGCGTCCCTAAAAAGGGCGGAATGACCGTCGTTAAAAATTAAAAGGATGAACTGATCCCTACTAGAGCTATAACTAGTCATAGAGTGTGTATTGATTATAGGAAGTTAAATGCTGCATCTAAGAAAGATCATTTTCCTTTACCATTCATTGATCAAATGCTAGAACGCTTAGCTAATCATCCATACTATTGCTTTCTTGATGGATATAGTGGTTTCTTTCAAATACCAATTCACCCTACTGATCAATAGAAAACTACTTTCACATGTCCTTATGGAACTTTTGCTTATAAGAGAATGCCATTTGGTTTATGCAATAATCCTACAACATTTCAGAGGTGTATGACCTTTATATTTTTAGATTTAATCGAGGAGATAGTGGAGGTCTTCATGGACGATTTTTCGGTCTATGGCCCCTCTTTCTCCTCATGTTTGTTGAATCTTGGCAGGGTATTGACCAGGTGCAAAGAGACAAATCTTGTTCACAATTGGGAAAAGTGTCATTTCATGGTGAAGGAAGGCATAGTGTTAGTTCACAAGATATCAGAGAAGGGTATAGAGGTTGACAAAGGAAAAATTGAAGTGATGTTGTAGTTGCAGGCACCAAAAACGGTGAAGGACATGAGAAGTTTCCTCGGTCATGCTGGGTTCTACAGAAGATTTATTAAAGACTTCTCCAAGATAGCCAGACCGTTAACCAGACTATTGTGCAAGGAGACCGAGTTTGAATTTGATGAGGACTGCCTCAAATCCTTTCACACCATCAAGGACGCCAGCCGGACGTTGGATGAAGCTCAAGGAAGATATGCAACAATTGAGAAGGAGCTTCTAGTTGTTGTATTCGCATTTGAGATGTTCAGAAGCTATTTGGTTGGATCAAAGGTAACTGTCTATACAGACCATGCAGCTTTGAGGCATCTGTATGCCAAGAAGGATACTAAACCAAGACTGTTAAGATGGATACTCCTGTTGCAAGAGTTTGACATGGAAATAGTAGACAAGAAAGGCATCTAAAATGGTGCAGCTGACCATCTGTCAAGGATGAGAATCGAAGAACCCATTCCGATAGACGATTCAATGCTAGAAGAGCAGCTCATGGTTGTAGAATTCTTCGGTAGGAGCTACAGCGGGAAAGAGTTCCACCAACTGAACGATGTTGAAGGAAGATCTCCAAGGTATGCTGATCACGTCAATTACTTGGCGTGCAGAGTAGAGCCTCCCAACCTGACCAGTTATGAAAGGAAGAATTTTTTCCGAGACATACACCATTACTACTGGGATGAGCCTTATCTTTACACTCTCTATAAAGATAAGATCTACAGGAGATGCGTCTCAGAAGATGAAGTAGAAGGTATCCTGCTGCATTGCCATGGCTCCACATATGGTGGTCACTTCGCGACGTTCAAGAGGAGGACCACACTCACTCGATCTAGCAGCAAGAGAGGACGACTTCTGCAGTCTTGTAGTTTACGCGACCGGGGTGCTGGCCGAAGCAGAAGAAGAGAGGTCGAGTATCGTCAAAGCGGTGCTGGCCGCCACAGAGTTGATGAGGTCGAGTACCCACATGTTGGAGCTGACATAGAACAAGGCGAATCGTCTATGGCCTGGGAGCAATCACAGGTAGCCATTGACGACCAACTCCGTTTATTCTTCGACTGAGGTAAGCGCCTCATGGATACCCTGAAATACTTGGATTTTCTTATTCATTTTAATCACTCTTGTTAATCCAAGTAGCTGACTCTCCTTATTAGAGCAGTTAACCCAAACCCAAACCTAAACTTTCTTTCAAGCCTTATATCACTTGTGAGTGTTTGTGAGGTCTTATTCCGATTAAGTTTAGTAGAAAGTGTCAGGTTTGTAACGACAGAGATAGTGTCTCATGTAGTTCTAGTTTACATTTTTCGGACTAGATAGGACTAGGTGAGCGCTTATACTTTAAGTGGGGATGTGTCTAAAAAAAAAAAAAAAAAAAAAAAGAGGAGTTGATTCATTGATAAGAAAAGGTAAAAGACTCTAGGTGAAGTAAGCTAAAGGAGCAGAAAAAGTCTAGTAAAGATTTTGGGATTTGTAAAGAAAAGAGTTCTTGTTAGCTAATGAAGAAGGGTAAAAGCCCTCAGTTTTAAAATTTTAAAAACAGGAACCTTAGTTGTTTAAGAAATCCAAATCCGCTAGATGTATCAAAGTGTTGAGGAAGGTTCTCCTAGAGTCAAGAGAAAAGAAAAGAATAATTAGAAAAAGGGCTTAAAAGATTCATGAATGCAAAGGGTATAGTTAAGTTTTTATATTGGGAGTGGAGATGGGATTACCATTAGAGCTTCATTGGTTATACTCTGGGTAGATGGGATCTTATCTCTGTATGCATAGCTTGGGACTTACTTTTAGCATTCTACTAAAGCTTAATCATTTTTTGAGAGATCCCATGTTACTAAAGCTTATTTTGTAAGGGACCATCTTTGTCTCTTGACCTTTACCTTAGCCAAATGAGTTCATTGATGATGCATTGCTTGATTCACGTTCCAGAACTAATGAATGTTAAAGGGATTGGTAGATTTGAAAACATGTGTAGGTCGAACATAAGAGTCGGATTGATTAATAACAAGGCATGGCTAACGTTTTTGAGTACAATTCGATCATATCCCAGCTTAGAACTATCAACTTGGACATTGATTTCATTTGATTTATCTGGTGCTTTGGCTCTGAGTCCTCGCCTTCAAACCTCACCTCCAGCTTGTTCTTAATTGTTTGCTTGAGGGCAAGCAAAGACTAAGTTTGGGGAGTTGATAAGTGTGCATTTTACATGTTTTGAGCATCCATTTGTTATCACTATTGCATCATATCACCACTGTTTTATACCATTTCTCATCATTTGTCATCACTTTGCATGTTTAGGATAGTTTTGCATGCATGTTGCATATTTGTGTTGCTTTCAGGTGATTTGGAGTTGTTGACGAGCTAATTGGAAGAAGCGGACTTGATCATATCAAACCACTCGACCCCCTGGTCGAGTAGATGCTTCACGACCTCAACAGACCACTCGACCCCCTAGTCGAGTAGAATACCTCATCACTTTACGTCACCACTCGACCCCATGATCGAGTACCCCCACGACAACCACTCGATCACATCACTCGACCCCCTGGTCAAGTATCATCACCTCCACCACCTGACCATCACTCGATCACACCACATGACCACCTGGTCGAGTAACTCCATCACTTTCACTCGATCACTCTACCACCAAGCCGAGTACCACGATCTCTAGCACTCGATTTCATACTCGACAGCCAGCTTCAGAGTCTTCTCTATTCTGCACTCAATCATCCTGCTATTACTTTGTTGTTATCATGCTTTCATTCCATTCAACGTTTATGCTTTTTGCTATGATGTCTGAGTAGTGAATAGGTTTCTGAGGATGGGTTAGAGTAGTTTAGAATTCTTAGTATGCTAAGTGGTTGAGTTTGATTTATAGATCTCTTTTGGATTAGTTGTTCTTAATGCCTATTGCTTTTTGATCAACTAGAATTTGAGCCCAAACATTTCCGCGCCCAAAAGGTGTTCGATGAAATGTCTAAACCATTAATTCCAGAGATTTGTGGCTTTGTACAAAGGTATTGGTTGTAGAGAGCGTTTTTGCTTTAACTTGTTGATTCGTAATGCTTGTTAGGTTAGCTCTCGTCAATGGTGATTAAGTCTGGGAGTAGTTTAACTTGAGGGTCTCTGTTGCGGTGGCACTTAGATTTTGTTACCGAACTTGTTGTCTAGGGATAATTTATTGAGCATGTCAATCACCTGTAAACTGAGGAAACGAAACTACTCAATTACCCCATCCTCGGAAATTCTTTATCTAATTGATTTCTTTGTTTACTCTACTATTGTTTACTGCATCTTGTTACCTGTTGCTTAGTTTTTGCATTTCTCTCCTTTTACTCGACCACCCAGTTGTCTGGCAACAGACTGTGCACTCGAGTATCTTTGTTACTGTCTTTGCTATTTACGACCTGTCACTCGACCACACCCAGTGCTTGGCAACAAGCTTTCTTGGTCGAGTGTTTTGATTTATCTGTTTGAATTTCTGTTTTCTGCCTGTTCACTTAGGACTGCTAGAACACCCGAAAACCTGTTATTGCTTGGCTTGACTCTGTAAACTCTGATTGCATCTTGATTGTTAGCATCACACCCATTTGGATTGTCAACCTAAAATACTACAACGACATGATTGGTGTTTTAGGATAATTGACTAAAAACCTATTATCACGGAACACCACCGGTTATTTCATAATTAGAGTTTGAGTTTAAGAATGTTTCGTTAACAACCTAACTATTATTAGATCTACGAACCAATAATTTCCTTAGAATACCCTAAGCCTGACGTTTTTACTTTCTTGTCTTACCACCTCACAATCTGCTATTGCTTCCTTTTGTTTCTTTTTATTTTCATTACTATCTTACTTAGCTTAATTCAAAACCTTGGTCTATTGTGTGATCCGAGAGCTTTGTGGAATTCGACCCTTATGTGCTACAATCAATCTCTTATTTGAGACAGTGGTCTTAAGGCAATTTGACCTATATCAAATTTGGCACCGTTGCCGGGGATCGACCCGGGTCACCCGCGTGACAGGCGGGAATACTTACCACTTAGTACAACGACCCAATATAGTGGTAAGTATTCTCGCATGTCACTCGGGTTCGATCCCCGGCAACGGCGCCAAATTTGATATAGCTCAAATCGCCTTAAGCTT

**F7B19 sequence**

gaattctttctgttagtagctttggccatggaaatcaatagataagaggttgaggcgctgcagcaggtagtggcggttgg

gaagggcggagaccggcggcggtggagaaggtccggcggtggtgagcagcggcggaggaggaaggtggaggaacactctt

ccttgccttctttttctgcccagtcatgttgcagatcgatgaaatagatacttggggaagaaagaggatgaaattctaat

gagattagagcctaaaatcacaagaacctgtgaacaatctagagagaaaagagtgatttcggcgttttgaataaaaactg

aaatgaagaagatgaagtttaggtgaaatcgacttaccttgaaaatgagagtgtcgatcaacaccatcgcgtttcgtcga

cacgatgttttttctttttttcaaatgagcatccatttgttatcactattgcatcatatcaccacagttttataccattt

ctcatcatttgtcatcactttgcatgtttaggatagttttgcatgcatgttgcatatttgtgttgttttcatctgattta

gagctattgacgacctaattagaagaagcgaacctgatcatatcaaaccactcgaccccctggtcgagtagatgtttcac

gacttcaacagaccactcgaccccctggtcgagtagaagacctcatcacttcacgtcactactcgaccccatgatcgggt

accaccaaaacaaccactcaatcacatcactcgaccccctggtcgagtatcatcacctccaccacctgaccatcactcga

tcacatcactcgaccacctggtcgagtaactctatcaccttcactcgatcactctaccaccaagtcgagtaccaccatct

ctatcgctccccttcatactcgactgccagcttcggagtcttctatattctgcattcaactagacactcgagcaaaagga

agaaaagaagactccagcttaccactcgaccatgcactcgaccacttgggtcgagtaccgttcttaatccgtcccaatac

tgcgtcgttttgagtattagggttattttgctataagtagcatgtactttacatttttgttatctaatttttatcccgca

gacactgtgttcttgacgtttttgtaatccggatttctctttatctattcagtattcagtatttgtttttcatttcgctt

actaagttgttcatcctgttatcatcctgctattactctgttgttataatgttttcattccattcaacgtttatgctttc

ttcagtgatgtttgagtagtgaataggtttctaaggatgggttagagtagtttagaattcttagtatgttaggtggttga

gtttgatttatagatcccttatggattagttgttcttaatgcctattgctttctgatgaactggaatttgagtccaaaca

tttccgcgcccaaaaggtgttcgatgaaatgtctgaaccactaattctagagatttgtggctctgtaccaaggtattggt

tgcagggagcattttggctttaatttgttgattcgtaatgcttgttaggttggctctcgtcaatggtgattgagtcaggg

actagtttaacttgagtgtctttgttgcggtggcacttagatttggttaacgaacttgttgtctaggataatttattgag

catgttaatcacctgtaaactgagtaaacaaaactactcaattaccccatcctcgggaattctttatctaattgatttct

ttatttactctgctattgtttactgcatcttgttacttgttgcttagtttttacatttctctcctgttactcgaccagat

actcgaccacctagttgtcgggcaacagactgtgtagtcgagtatctttgtcagtgtctttgctgtttactcgacctgtc

actcgaccacacccagtgcttggcagcaagctgtgttggtcgagttttttgatttatctgtttgaatttatgtttcctgc

atgttcacttaggactactagaacacccgaaaacctgttattgcttggcttgactctgtgaactctgattgcatcttgat

tcttagcatcacacccatttggattgacaacctaaaatactacaacgacatgattggtgttttaggataattgactaaaa

aacaattatcaatttggcgatgttgccaattgggtgtttgtttgttacatttgagatttcagacttgtttagatcaagtt

ctttttcaattttcatttcgattactaactgtgtgtttttattgttgtcttcttaatccaggtacacctctactttatgc

aaactcgatcaacaggcaaccagattctcctttacaacgatagcatcgaccgcatagctcgtgaactcaaagaaaggaga

aacacaatcaaacttgtacctcagcaaccattcgatatggctgacgaacaaaatcaacagaatggccctacaaacattgg

tgctggagatgcaccaagggaccaccgtcaaaggaaaggaattgcacctcctgctatccagaacaacaacttcgagatca

agagtggtctcatctagatgattcagggaaacaaattccatggtctgccaatggaggatccactcgaccacttggatgaa

tttgatatgctctgtaacctaacaaaattcaatggtgtcagtgaagacgaattcaagctccagttgttccattctctttt

ggagacaaagcacacatctgggagaagaatctgccccatgactcaatcaccacctaggatgattgcaagaatgcttttct

agcaaagtttttctccaacgccagaactgcaagactcagaaatgagatttctggtttttcacagaagactagtgaaagct

tctatgaagcatgggagcgcttcaagggttacaccaaccaattccctcatcatggtttcacgaaagcctctctgctcagc

actctatacagaggagttttaccacgcatcagaatgcttctggataccgccagcaatgggaatttccagaacaaagatgt

tgaagaaggctggaaattggttgagaacctagctcaatcagatggtaattacaacgaggactgcgacaagaccatcagag

gcacaaccgactctgatgacaaacacaggaaggagatcaaagcgctgaatgccaagctggacatgattcttcttagccag

cagaagcatgtgcacttccttgttaaggacgaggattatcaagtccaatatggggagggaaagcagttagaagaagttag

ctacatcaacaatcaaggtggcgacaaagggtataacaacttcaagaccaacaaccccaacctctcttaccgcagcacca

acgtgtctaacccacagaaccaagtgtatccaccacagcaacaacaaggtcagaacaaaccttttgtttactacaattaa

tgttttgttcccaagcagcaatttcaagggaactaccagcagcaaccaccacctgggtttgcacctcagcaacaccaaag

tccttctgctcctgacgctgatatgaaacaaatgctacaacagctgcttcatggacaaccatctggctccatggagatgg

ccaagaagatatctgagttacacaataagctggactgtagctagaatgatctaaatgtcaaagtggaatcactaaatacc

aaagtccgttacttggagggacattctgcatctacttccgctcaaaaacagataagccaacttctaggaaaagcaattca

gaatccaaacgaatatgctcatgatatcactctgcgcagtggaaaagcacttccaaccagggaggaacctaaaacagtca

atgaggacagtgtagatcaaaatggggaggatttcagtctcagtgaagatcaagctgacaaacaactcgagcagtcaccc

gaccatgttactcgaccaccattcccagaaacatcaccaattgctctgaaacatgttgctgtcaagaaccaagaaaaggt

ctttgttcctcctccttacaaaccccagcttccattttcgggctgtcacaaaaaagcattgacagataagtatagagcta

tgtttgcaaagaatatcaagaaggttgagttgcggatacctcttgttaacgctctagcactaatcccagattctcacaag

tttctgaaagacttgatcgtagaaagaattcaagaagtgcaagggatggtagtattgagtcatgaatgcagtgctgtcat

acagaagaagatcattcctaagaagcttagtgatcctggttcattcactctaccatgctctttaggtccattggctttca

acagatgcttatgtgatttaggagcgtcggtcagtctcatgccactctctgttgccaaaagattggggttcactcaatac

aaatcctgcaacatatcccttatcatagctgacatgtcagtaaggatcctgcatggtttactcgaaaacctacctgtcag

gattggagctgtagagataccaagtgactttatagtcctggagatggatgaagagcccaagaatcctttgattctgggag

acctttcttagcaattgtaggagctatgattgatgtcaagaaagggatgattgatctaaaccttggcaaagactttagga

tgacctttgacgtcaaagacgaaatgaagaagcctaccatagaagggcaactcttttggattgaagaaattgatcagtta

gctgatgagttactggaagagcttgcaaaagaagatcatcttaacggtgctttaaccagaagtggtgaagatgggtttat

gcatttgaaaactttgggataccataagctgttatactcccataaagcgatggaagaatcagaaccctttgaggaattga

ttggaccagcaacagaggtaatggtaatgagcgaagaagggtcaacacaagttcaacctccacactcgagaacatactcg

accaatcactcgaccttgactgtcagcgactctaggaactgatcatcccaacttcacataactggtctgaactcaaggcg

cctaaggttgatctcaaaccacttcttaaaagtttaaggtacgcattccttggtccaaactctacttaccccgtgatcat

taatgctgagttaaatgatgatgaagtgaacttgctattatctgaacttagaaagtataggagagcaattggttattcat

tatgtgacattaagggaatttcacctagtttatgcaatcataggatccaccttgaaaacgaatcctattctagcattgaa

ccaaagaggaggttaaatcctaacttgaaagaagtagtgaaaaaagaaattttgaaactgcttgatgctggtgtcatcta

ccctatctctgatagtatttgggtttctccaatgcattgcgtccctaaaaagggcggaatgaccgtcgttaaaaattaaa

aggatgaactgatccctactagagctataactagtcatagagtgtgtattgattataggaagttaaatgctgcatctaag

aaagatcattttcctttaccattcattgatcaaatgctagaacgcttagctaatcatccatactattgctttcttgatgg

atatagtggtttctttcaaataccaattcaccctactgatcaatagaaaactactttcacatgtccttatggaacttttg

cttataagagaatgccatttggtttatgcaataatcctacaacatttcagaggtgtatgacctttatatttttagattta

atcgaggagatagtggaggtcttcatggacgatttttcggtctatggcccctctttctcctcatgtttgttgaatcttgg

cagggtattgaccaggtgcaaagagacaaatcttgttcacaattgggaaaagtgtcatttcatggtgaaggaaggcatag

tgttagttcacaagatatcagagaagggtatagaggttgacaaaggaaaaattgaagtgatgttgtagttgcaggcacca

aaaacggtgaaggacatgagaagtttcctcggtcatgctgggttctacagaagatttattaaagacttctccaagatagc

cagaccgttaaccagactattgtgcaaggagaccgagtttgaatttgatgaggactgcctcaaatcctttcacaccatca

aggacgccagccggacgttggatgaagctcaaggaagatatgcaacaattgagaaggagcttctagttgttgtattcgca

tttgagatgttcagaagctatttggttggatcaaaggtaactgtctatacagaccatgcagctttgaggcatctgtatgc

caagaaggatactaaaccaagactgttaagatggatactcctgttgcaagagtttgacatggaaatagtagacaagaaag

gcatctaaaatggtgcagctgaccatctgtcaaggatgagaatcgaagaacccattccgatagacgattcaatgctagaa

gagcagctcatggttgtagaattcttcggtaggagctacagcgggaaagagttccaccaactgaacgatgttgaaggaag

atctccaaggtatgctgatcacgtcaattacttggcgtgcagagtagagcctcccaacctgaccagttatgaaaggaaga

attttttccgagacatacaccattactactgggatgagccttatctttacactctctataaagataagatctacaggaga

tgcgtctcagaagatgaagtagaaggtatcctgctgcattgccatggctccacatatggtggtcacttcgcgacgttcaa

gaggaggaccacactcactcgatctagcagcaagagaggacgacttctgcagtcttgtagtttacgcgaccggggtgctg

gccgaagcagaagaagagaggtcgagtatcgtcaaagcggtgctggccgccacagagttgatgaggtcgagtacccacat

gttggagctgacatagaacaaggcgaatcgtctatggcctgggagcaatcacaggtagccattgacgaccaactccgttt

attcttcgactgaggtaagcgcctcatggataccctgaaatacttggattttcttattcattttaatcactcttgttaat

ccaagtagctgactctccttattagagcagttaacccaaacccaaacctaaactttctttcaagccttatatcacttgtg

agtgtttgtgaggtcttattccgattaagtttagtagaaagtgtcaggtttgtaacgacagagatagtgtctcatgtagt

tctagtttacatttttcggactagataggactaggtgagcgcttatactttaagtggggatgtgtctaaaaaaaaaaaaa

aaaaaaaaaagaggagttgattcattgataagaaaaggtaaaagactctaggtgaagtaagctaaaggagcagaaaaagt

ctagtaaagattttgggatttgtaaagaaaagagttcttgttagctaatgaagaagggtaaaagccctcagttttaaaat

tttaaaaacaggaaccttagttgtttaagaaatccaaatccgctagatgtatcaaagtgttgaggaaggttctcctagag

tcaagagaaaagaaaagaataattagaaaaagggcttaaaagattcatgaatgcaaagggtatagttaagtttttatatt

gggagtggagatgggattaccattagagcttcattggttatactctgggtagatgggatcttatctctgtatgcatagct

tgggacttacttttagcattctactaaagcttaatcattttttgagagatcccatgttactaaagcttattttgtaaggg

accatctttgtctcttgacctttaccttagccaaatgagttcattgatgatgcattgcttgattcacgttccagaactaa

tgaatgttaaagggattggtagatttgaaaacatgtgtaggtcgaacataagagtcggattgattaataacaaggcatgg

ctaacgtttttgagtacaattcgatcatatcccagcttagaactatcaacttggacattgatttcatttgatttatctgg

tgctttggctctgagtcctcgccttcaaacctcacctccagcttgttcttaattgtttgcttgagggcaagcaaagacta

agtttggggagttgataagtgtgcattttacatgttttgagcatccatttgttatcactattgcatcatatcaccactgt

tttataccatttctcatcatttgtcatcactttgcatgtttaggatagttttgcatgcatgttgcatatttgtgttgctt

tcaggtgatttggagttgttgacgagctaattggaagaagcggacttgatcatatcaaaccactcgaccccctggtcgag

tagatgcttcacgacctcaacagaccactcgaccccctagtcgagtagaatacctcatcactttacgtcaccactcgacc

ccatgatcgagtacccccacgacaaccactcgatcacatcactcgaccccctggtcaagtatcatcacctccaccacctg

accatcactcgatcacaccacatgaccacctggtcgagtaactccatcactttcactcgatcactctaccaccaagccga

gtaccacgatctctagcactcgatttcatactcgacagccagcttcagagtcttctctattctgcactcaatcatcctgc

tattactttgttgttatcatgctttcattccattcaacgtttatgctttttgctatgatgtctgagtagtgaataggttt

ctgaggatgggttagagtagtttagaattcttagtatgctaagtggttgagtttgatttatagatctcttttggattagt

tgttcttaatgcctattgctttttgatcaactagaatttgagcccaaacatttccgcgcccaaaaggtgttcgatgaaat

gtctaaaccattaattccagagatttgtggctttgtacaaaggtattggttgtagagagcgtttttgctttaacttgttg

attcgtaatgcttgttaggttagctctcgtcaatggtgattaagtctgggagtagtttaacttgagggtctctgttgcgg

tggcacttagattttgttaccgaacttgttgtctagggataatttattgagcatgtcaatcacctgtaaactgaggaaac

gaaactactcaattaccccatcctcggaaattctttatctaattgatttctttgtttactctactattgtttactgcatc

ttgttacctgttgcttagtttttgcatttctctccttttactcgaccacccagttgtctggcaacagactgtgcactcga

gtatctttgttactgtctttgctatttacgacctgtcactcgaccacacccagtgcttggcaacaagctttcttggtcga

gtgttttgatttatctgtttgaatttctgttttctgcctgttcacttaggactgctagaacacccgaaaacctgttattg

cttggcttgactctgtaaactctgattgcatcttgattgttagcatcacacccatttggattgtcaacctaaaatactac

aacgacatgattggtgttttaggataattgactaaaaacctattatcacggaacaccaccggttatttcataattagagt

ttgagtttaagaatgtttcgttaacaacctaactattattagatctacgaaccaataatttccttagaataccctaagcc

tgacgtttttactttcttgtcttaccacctcacaatctgctattgcttccttttgtttctttttattttcattactatct

tacttagcttaattcaaaaccttggtctattgtgtgatccgagagctttgtggaattcgacccttatgtgctacaatcaa

tctcttatttgagacagtggtcttaaggcaatttgacctatatcaaatttggcaccgttgccggggatcgacccgggtca

cccgcgtgacaggcgggaatacttaccacttagtacaacgacccaatatagtggtaagtattctcgcatgtcactcgggt

tcgatccccggcaacggcgccaaatttgatatagctcaaatcgccttaagcttactccctcaaataagagttgtcgttag

cacttaagtgtcgaattccactgagctctcgatgttcacacaatagaattatgatgttattaaataatctagacaaagta

attgaatgtaaaagacaaataaccaagtaaacgaagtgtagattcaagtgattaaagcgtcgggtctaaggaattgtctc

gggagatagataaattgtagatctagctaatataaggattgttatggcaccgttctcaaactcaaactaagattctagag

taaccggtgatgttccacaaaactctctttacttagagctaagataaccggagaaaccgagaaatcttatactaaagcat

gcattgttatcaagcttgattagttcacctagtatctaaaccagagcccttctatgagcctacatgttctttcttaaatg

cctaaactcatctatgatagttcaaacaacaagtacctatggtgggcatacctattatctaatatcacgttctaggtgat

caatctaaaactagcaataagaataatcaagatgaagaactataagaataatcttaaggggttttcgatctactaatcca

tctaaatccctattgagactccctaaacccaacaaggtgattactcagatatgataacagaaacacaaatcataatctga

ctagtttgcattgatgaattagaaatagagtaaaaggagtccagaatcctctctacagggattggattcttctctccaaa

atgtatctcaatctctgaaaaaacaaaacttaggtctaacaatgaccaaataattaggcatattcgtgtctaaaaaaagt

gcaaggactactttgcaaataaggaaaactctagggcttctcaaagacttggcagaaaacaggaattccctcagtcgaaa

taagggtttcaatcgacataggtatggtgtcgatcgacaccaacttctgaaaaacgtctttgataatcttcattgctgaa

aagctttcaaaattccttattatgcttcatagctcccagatgtgtaagtgtacttgaaatgactcaaaacatacgaaaaa

gactctaaaacccattatgaaactatgttaaatatcagtaaaaaccaagatatatacggggttattatctacacgtgtgt

caaaatgatcaatagaatcagaactatggtaaatcctttcttcacctgaaaagagtagtagttcactgtcatatacactt

ataagtagcaggtgataaagttatatgtatacttttgagtaatatcaaaagttaattcactaacctgttagcttggaaag

catatgatcgttaattgagttaacatcgtcattggtgggacacaaaatacctctatcttggaaaaacgttggatctctct

cttgagcaaaggttgtaccatagactgcttgaatgattaattctatagggttgtctccttcagaaatacgattatcatct

ggtatttgtatctgagttactccgtcgttcggctcatttagttttccctcgccaactgcaaggatccatttcaaaaattc

ttcaatctcacgtgcctcatttatgtctatatcttggagaagcctcatgttcttcgtcaactttaaaactttgcaatgct

gctaaagataagacgaattgagtgacgatttgacaatcagttccctaccggctgcagggatgacaggaagaatttgccta

aaatcacctctgaaaagaataacttttccaccaaacggcttatcttctggagtcgatagaatatcttttaaactcttatc

taatgactcaaaacagtacttactcatcataggagcttcatcccaaatgatcaacttagttgctgttactaattcaacaa

gattagtaccacgttctatgttgcatgttgaggtctcatgaggagttagaggaataccaaacctagaatgtgcagttcgg

ccaccttctaataacagagatgcaatgccgcttgatgcaacatttaaacaaatatctcctttagatcttatcgctacgga

tagaaccttctacaaaaaggtttttcatgttcctccaaaaccatataagaacaaaacacctcctttgtcattaaggacaa

catccattatttcatcatataccttcttctattccgcagttaacatctttaaccaattatcatgttttgttttcaatgac

tcacggttgtagttgcgttcatcgaggatcaactgattaaaggcaggttcatcgccaggttcaagctgaggcaacatctt

atacctccaaagatttgttccatgctttttcaatattgtttgcaactcttgtaaacataactgaatcctgtcttcgtccg

acaatatgtattctacatataaattaatattagattgaatgtactaaactatttataaaaacatctataaaattgtatat

ttaattaaaaataataatttaactaacatggttgtctaactgatttccaagtgtgttcccatacagacagtatcttctaa

gtgtgttcccacacaacggctggggaacacaagtttcggaaataagcataataacaaacaacttgcgaacatacttagga

gaacaccaaaaattagccttctcaataccgtggatatattccttatcatcatcaagcaaacctagagtatagcatgaatc

tctgtatgattcatgaacaacaccctcgacggttttgatatcatcaaatcgttttggaccatttttgttgttaatcagga

tgcgaaggtgatatgcatcatctatctttggaggtacatgatttattctaccaacagcaaaacctggcatttttcttctg

tgaaactccttatctttaccattccacgtatagaaattaggtatttgttcatataaaagttttgctgcttccagatcacg

cttattaagctcgaaccttgcggtaaattgagtctcatcattattcactctatacaaaacatattcagcagtctcacctt

ccttgacataaacgggttacttgcttttctcatggaatgtaagtttaaatactgatgtttgtcgataatgtataggatac

cctctaatcttccacatcgcttcacatgcagatacgtatctgtaaaacggtatttgagttatttttcattaagtttcata

tttaaaatagtctcctaagaatgtttacaataaactgaaataaaataatgaaaaatacctgcaatcaaaataatcctgaa

cttgatttcgttcttgtggatctttatttgtttctcctacattgtttttctcggtgacatcttcttttcgatgaggctcg

acagagacggttacacgatcaggtccctcgttaacatatttgaataagtatttaacagagacgtttggttacaccactct

acattgatgtgagccctgtatttccgtaacaggacatcgttgtatggaacaacataccggttgtcacattgatacttatt

cttctcgatgaaaagaccagtatctatgcgtctatatattggataaccttcattgtcaagagacgtgttctcaacattgt

tctttggataaaacttggaatatttaccattttccatgcatggcaaattaggattaaccaatccgcatggttcatgaatc

atacattccgacacaacttgatatagctctggatctttttctttatctggaatttcggcaaagataatctttttaacgtc

atcagcggtaggaaacttgtatctaggatccattcaaaaaattatgtgagcatgtggaagacctctctactgtatacatc

tgtttcaacataccaattatgagttaatgtcacttgcaaattaaaattcatgaaatatatgttttgaaaatatgaaatca

tttagtacctgatttggtttttccaaagatatgattctttgttagatcatccatcaagttgtcgagtttgattttataaa

tcctgcaaatgatatccagcctgtcctcagaattcaaattccgttctttaacaaatcttgtgatctctggccacttagga

ttacatgtgaaggttataaacaagtcgggaaagccaaaatttttataggtcgccattgcatctaagtagttctgctgcat

ataagctggaccaccagagaaagatggaggaatgatacaactttttcctttattagaaagatcattatttccggcatcag

aagcatcttgaactgcctgcttgttggtactgcgcaacttcgtttgatttttttttatataccttaaccgattagactct

atcatggtaaaagcgtcaaccatgaattgttgaagtaaacgctttgagcgaaggaggatgtgcttctcatcttttctcgc

ttgtaaacggtagtcaaaccactgcctcatactaacatcttcctgcttcttctttccttttgttattttggtaggagttt

tttttattccaagcctataaccatcctctccttttggaaataacaatggatactaaaaagctagatatgaaacgtgacat

tcaagtattctccttaactttccagacttcatctgtaggactatatctcttgtatctagattgtcgtcaaaatctccagg

tatcaatgcagcaacttcagctacagatggaaggttatacactctcccatctttgtcgcgcttagatataatcttcatat

gaaaatttgtctcttctctctttatgttgaatctatcacgggcaatcctaaatgcaccaacatgagggtttatcttgttt

aacatttttagtaaagctgcaactattcttgaacttttgtttaccaccttctgtcccaccattattacctttgctgcaat

tttggataataacaaaagacaaataataaacaaaatgtattaactggtacaaaatataaatttttcataaagctcaattg

aagttaacacttacctcataacttcaattcgattatcaacttcattcttagtgtccatgatataaagctgttggaatttt

gcatatacgccaggttttggtttcaacgcgcctatcaagtgattgttttctctttgtaaaacaaacatagatggaccacg

tctcttttttactgatcgatctaccttgccaccaatagatgtgaatgaaaacaacatgttgtatggtctgatatttgctc

taaacaatttagcatcttcatggtcgcttgtaagcaaccaccacatatatttaggtgattcttttaacataagtagaaca

atctgtccttgcatacaacatcctgtaaaacctggattagcactcctccttcttctatttagtcctcactgtaccagaaa

attacaccacaatgtagacatgtgtgattcgcatcaccttcgttatgataacctgaatcgaaaaaacatcaataacaaat

atgttatataagtttttccttgtttctaatatatataatatattaacaaatcatctttgtttgaagctgcaacaccaaac

tccttgtttaaattccttattaacaaatctttcatgtcgatcatatattttggtccaacaacctgatcgctcggtaaatc

tttatccgactcatactcttcttactactacagtcaaatatatgatcatcaaattcttctgacctatctgtagacaggag

atgaaatgaaagtagaaaaaaataaagcaattttacactgtttatataaaggtagctatttttaaaagtatgattaacat

tataccattattagaatatattgcttcagaatgatcaatttcttcatcattgagcgtattggcacgtacaatacttgtag

aattttcctttgattatgtttttgttgaaagtgggactcctaacaatcaacaaacaaaaaatattaaattttttatggaa

gtaataatataatttctgttaaactattgaaaattaggcaaacatagtaaaacataatataaatagctaattttcaaata

tgaatcgaacgttgcccatttgatacttttttcttcttattcgacattaatcaaatggtatagaacttggatcctctcct

agaacctcccgatatatttgttatatcctataagacatttccagagtcatctaccccaatcaatatatttaagtttgggg

tagtttgattaggagttaagaagtctggtgatcttactgagaaaaacaacaatcaaatgaagataaataaaaatattaac

taaaatgtattgtatctaatacatataactttataacctgatgagattgatctttttctggccattcttttaacagcaga

caaccttcgaacatgtgttttgttaacaatatcataattgttcgttgcttcatcattggtcttctctcgaagtaatgatg

atattcttttagtattggataaatttcctgcaaagaaaatataaatatcaacacataacatataaaaaataattttttta

tatttaaaatttagatgataatattgtatatcgaataatatataagttactaacctgatttcctatttttgcggcagctc

ttgtaaaagttggattaatcacttctgagctattagatcgattgactatatttcctcttgtgtttggtatattataaatt

actctgtctcatttggagttcaaacatatatgaatcaaaataaagtcgaaagaagtaatgatgaggatatatgagcatac

tacctgtaagagttgaattcatcgaattaataggggtaatgggtgaggaactcccattattatttcccgagtataagggt

aaggacacatgagtatgaggagtctgactcctttttgtaagatcgatccaaatgttttattatgcgaaggaaagttggtt

ttatcgaacaaacgtcttgttcgttttgaagttttctgattttttttgttgttgatctacatcattttcatcattatcct

ttcttttcttcattgtaatcgacgaataaaaacaaagaatattgatttggtttcagtagaaaggctaaatgtttcagaaa

ccaaataaaatgataaacataacaatgctaaaaattaggaaacaatatatatgaaaatgtattgttaacaaaaatatcta

taatataattctatatggcacaacatacgaaatgtatattctagtttagtttatctactctattattatgcaaattgtaa

cacaacttatgagatgcttgattaagaaactcatcgttgaaataattgatgtttccaacttagaagattctctaacgttt

taatgacatatttcgttggacaaatattttaataaattctaagttttgcatatttattttctttgtttaatacattcata

tctaacacatattttagtttacatttgatttcaaaagttccatttcaaatacattattttaaaatcaaaaaaatatctaa

ataatttaaatccaaataacctaataagtggccaatatcaaaaaaaagttataattttatctaagattacggaactccag

ctgatttagtaagcattaactaaaacgaagcataatcaaaattaatgtttaacaaaaataatgcataggaaatgaaaaaa

aaacatatacaaagtcttaatagtacctgttttaaatataatagagaagattttataaaaacgatggaaacaagtctggt

attgatgttttccgttctcatcaacaacttcacctatttcagctcgttgcattcgttcaaggaattgagtcctgaaacag

tagcaaaaaaagaaggaaataaatagccaagaataaaattatttataatacactaaacagttaagagataatgaaaatat

aaacgttcttacgtgatgcgatccatgttaatctctgggtaactttaattgaatgtaattcttgaagcaccattatgtgt

gattagacattgacgacctaaaatatttatgtttttattatatgcattagctataaaaaaaacatatgatgagaagagag

ttaaatttaccttcatgatcagcaacgttccaaaatcgaagcacgcatgttataggattcataccgaaggtccactgata

accaaggtgtagaccaagttccctccacaaacgcacaaacaaatcacaagtatatccacttgcaacacatgtaagagttt

ttcccctaataagaatatcttaattatttctctctaacatctaagttctataaattaagccacctataaagataacaatg

cttactatgtttctacattaaaatatataaccaaaaatatgtcgaactatatagtcggaaatactaaccaagtattttta

agctcaaactcgagaaattgagaaagtcgaggttttcggaatattgaggaacataatctggtcttgttatattcccaact

cgcacgacaacaccataagcatctacaaaacattttcagaaatttgagagaggtatgatattcctttgtaataaccacct

ctatcatctctcaacatgtttttattttttttcaagttaataggtcagaagaaagatgaagaaggtaagtaatggatata

aagaagggaggaggttactttttttttatgggttaggatatgtgtaggcgtatagcttagaatattttagtgtatattac

ttttacaaaataatttaaattattacaataatttatatgccatacaaatcttgtcagtcgaatttggaatatcgatggta

agttataatgctttttatggttacttgcaaaaagtaattaattttgaaaggaacaaaatatgacataagttgtcatatct

cttggatgagttgtcaaattttgttaaagttgtcaacaatatatttcattgagacaggaagatatgtaaataaattgaat

tcaatataaaagtcaatatttttatctactcaattaaagaacagaattattccacaaaatatgtataaaagcacaatata

attataatagaccgaaatcttatatatatatattacattaaataaaggttaaattttaacagttatatatgtattgttat

tttttaagtcataaaagtaatgatgtgactttgatatgtatccaaaaagaaactttaaaaggtgtgcatagatttccaaa

aagaaaattcttttaatataatagaaaggatattgaaagataatgcttgtgtagggttttaaatctaaagagtccaaata

tatatgattatgttttgtaaaagacaatgacattgagttgaaaagaaatgaacttaatatttaaagatccttgataatag

gtttttggtcaattatcctaaaacaccaatcatgtcgttgtagtatttttggttgtcaatccaaatgggtgtgatgctaa

caatcaagatgtgatcagaagtcactaagtcaagccaaacaataacaggttttggtgtgttttagcagttctaagtgaac

atgcagaaaacagaaaatcaaacagaaacagtaaaacactcgaccaacacagctctttgcagagcattgggtgaggtcga

gtgacaggtcgagtaacagacaaaaaataaaacagggatactcgactgcacagtctgttgccagacaactgggtggtcga

gtatcaggtcgtgtaacagtaaagaaatgtagaaattaaacaacagatgacaagatgcagtaaacaacagtagagtaaac

aaagaattcaatcagataaagaattcccgaggatggggtaatcgagtagcttcgttttctaagtttacaggtgattgaca

tgctcaataaattaaccctagacaacaagttcattaaccaaatctaagtgccaccgcaacagagaccctcaagttaacct

agtcccagactcaatcaccattgacgagagctacctaacaggcatgacgaatcaacaagttaaagccaaaacgctccctg

caaccaataccttggtacagggtcacgaatctctggaattagtggttcagacatttcatcgaataccttttgggagcata

aaatgtctgggctcaaattccagttgatcagaaagcaataggcattaagaacaactaatccagaagggatctatcaatca

atactcaatcacctagcatactcagaattctacactactctaacccatcctcagaaatctattcactactcaaacatcat

aacagaaagcataaacgttgaacaagttgaaaacatgataacaacagtgtaacaacaggatgataacagaatgaacaaca

gtaaacgaaatcaagacaaaagctgaatactgaatactgaatagataaagagaaatccggattacaaaaagactagaaca

caaggtctgcggaataaaactaggtaaaaacttctttttgcgaaaatgtgaagtacatgctacttatagccaaatatccc

gaaaccctaatactcaaaacgacgaagtattgggacggataaagaactgtactcgacccatgtggtcgagtgggaggtcg

agtgagtagctggagtcttctttcctttcctgtgctcgagtgtctggttgagtgcggaatgaagaaggctctgaagcttg

tcatctagtatgcagtcgagtggtgtgatggtgatactcgacttccaagtagagcaatgaggtcgagtagtggtctggca

atgaaggtgaagacactcgacctcgtggtcgagtgaagtgatcaagtgtttgtcaggagatactcgacttccaggttgat

tgacgaggtcgagtggttgtctgatgatggaggtgaagacactcgacctcatggtcgagtgatgtgatcgagtgatggag

atgagggcactcgacctcgtggtcgagtggtgaagtgaagtggtgatgtcctctactcgacctgggggtcgagtggtctc

ttgagatcttgaagctcctactcgacctctgggtcgagtggtttatcatgatcagatccgcttcttccagatagctcgtc

aacagctccaaatcacctgaaatcaacgcaaatatgcaacatgcatgcaaaactatcctaaacatgcaaagttatgacaa

atgatgtgaaatggtataatcagtgatgatatgatgctaaagtgatgacaaatggatgctcaaaacatgcaaaatacaca

tttatcaactcccccaaacttagtctttgcttgccctcaagcaaacaatcaagaacaagctggaagtaaggtttgaaagc

ggggattcagagccaaagaaccagatagaccagatgaaatcaatgtccaagttgaaagttctaagctgcaagatcaaatt

ctacttaaagacgttagccatgcctcattatcaatcaatccgactcatatgctcgacccacacatgttttcaaatctacc

aatccctttaacattcattagttctggaacgtgaatcaaacaatgcatcatcaatgaactcatttggctaaggtaaaagg

tcaagagacaaagatggtcccttacagaataggcttcagtaacaggggatctctcaaaaaatgattaagctttaatagaa

tgctaaaggtaagtcccaagctatgcatactgagataagatcccatctacacagagtatatcaaatgaagctctaatggt

aattgttgattctgtaatcaaacaatgaatttgtactaagggatttagacaaattcactcctctcttagctcacaaataa

tcaaaggaaaagaactaagttgcttgaacaaaaagctagggtttgctctgtaatagtagtctcttgtattaggttaaaaa

tggtctcaatggggggtttgacccctctatttatacaatgggtcgaatataccaaaatatgataaacttctaattatcct

aggtctaaatcttcattacttctattcttcataagatcctcttctcttaaatggaagttttccctttacacgacctaaga

agttggttatggcgatcttgattgtcgacttaagtcttcgggcgagctcatgacttaggcccattaacaggctaaccggg

ataccccggttcaatcgacttctcgattttattcacattattcctcggtttaaattggtattgtcggaggtgctaattcc

tgcaccaacaattagtccccccccagtccggagaatccgagttgcctaattcgaaatttccgaacttagaaattgaatat

ttgatttaaatcttggaagacgtgaattaatcattagatctccgaatcgattcgtatttcgaacgtatttgacacgtgtc

aaatcgtcgcatctgtacgcgcgcgtgaagctcaagaaccgaagcgtcccgtaggtttaatgatgacctcgtaggcttgc

cgcccattcgtctctatttatattcgaccaaaccctcgggaaatcattttctgccgcttgcaagaagaatcaaaggtagc

tttcgctctctcctctacgaattattttgtattctcgattctttatcgatttttccggcaatgggtaaggctgttcttgt

ttctccgtcctcttcttcctccgagagcgaagcgtcgttagttcgccgaaactctcggttaaccgggaatccaagtgacg

aagctcaatctagtcgatcgaatcggatcgaaattccgacgttgcttgatgagccagatcgggaaggttccggtggacat

cccgctgctcctgaagattcggtggattttttagctcagttcgacccactcatcgacctggaggatgcttacaacacgca

tccgcagtatacagaggctgaagttaatgcgagattacttcatgaggattatgtcgaatttgacctcgacccaattgttc

attggaacaccgagatcaccgtaaaaaagaccctttcaacaaccgagtcagtcgcggaagtatttaccttgtgcggctcc

ggagatcgcccactcacttacatgattctcggagaagatgagcgtccttggaatccccctcgaggatatgtctgtatgta

tgaagcctacttcagacagtgtcatctttggtttcccattccgtctctgatcatctcctttctaaatcgccgtcgtatgg

cgttctcccagctcactccggtggcgatctgcaatttcgttgttgctctgaccttcggcgctgaagaaggttatctggta

aacgttcgttgtttcgaggaaatgacaacgttgaaagctatcaggtcgcctggctattgggtggtgaataaccgcccaaa

gtacaactttcttcccggtcccaaagtcagtaatttcaagaactgggaagaatattacttctacatcagggtcgacctcg

agtcgtatgagcgtccgttctcgtggaggaaaaggatgtggacggagtttcctggtagacttcttttaaatagatagggt

aaagacatgtcgatcccgattagattctcatagccttttcttttgttgcagatcggtacaggcctagcccggagtttccc

atggagttcgagggagttttagaggcaatcttccaagctcaacgtcgtgcgtggaaagacatcacgaggtcgcgcgtcaa

caggataaaggggaaggtcaggaagccctttattagctttgctgccgggcttttgaatatgccgcctcctcagctcccat

cgaagaggagactcatcctctagacggagaggcggctacgtcgaatcctcccgttagtgctggcccttccggtgttgatc

aggtctcccatgagacggtcaatccagattctcaagataggacgcgtcttgaaggggccgacgtcgttccagctcaagag

atcgccgagtgcgatcaaagtcaatccgttcaggtcgtcgagcctgaaattcaaaatgttaccagcgaccgctccatcgc

cgtttatgccgccgtgccagcaggcgaagaggtcaacacgggcctagttatcgttgatcaagacaaggatgaaaccgttg

aggagctcgagacttccaggaggtcccgtgaagagaaagggaaaggagtggctaaccaatccaagaagaggtcggcctct

gaagccggtttagatgaagttgctgctccgaagacgtttcgcctgtcgcgaggtgaaactcttaattctgaccagttttc

gtttaagtattctggtgcgaaattccttgtgagagatcaggaggccgccagtcatctttggcgtaacttgatgctccctg

ggacgaaggatttccctaatcttgacgacctggttctgaaagagggatatcaaaagtttgccagatcatcacttgaggta

cgtgctagttacatgtcttcctgtggcttaatatctttgtattttagctttaaccgtaactctttttccagaccgccgcc

ctggccaatgatctgattgcgacatacgatcgcaagttgaagctgaagctcgccgacagagaggccttcgataacctgaa

gaaatgcgcagaccaggccaaagccatctacgccaaagacatgaaggaaatggctgcattgagggatgctgccgagattc

ataaggcggaaatgagctcgttgaacgacgaggtcaagaggctcaatggtcgagaagctgatcttcagaaagagatcagc

gatcttcaggtcgcgttggttgctgtgaaagagcacggggagaaggaatgcaacaggctgcggaatgacctagttgctaa

ggtcgcccgtactacaaagaaagcccaagctcgtcttgataaagtgaaggcttatctcaaggagcaagaggaccttgtcg

ggccaaaggtggatgttgagaatcaagccaaaggcgctgaggagatagtgggcattctgaactcaacggccttaaggagt

taaccaaaagggcgactgacgaagttaatgccttgaacgtcatcgagctcggggatgatgacctgaacatgtccccggat

cagctcggttttttgaggcaagcctcccaaattgctcctgtggcggaccatcacagctcaaatgtcgacctcgttagcgg

aagcgatattcgacgtgtaagcgttgaagaaacgaaccagacgacctgaatcggtcgtccttgtaatttattttgataca

ttgatcgtggacccaactttcggggggatctcaaaactcatgtttaaccgtggacccactttcggggcggattttaattt

taaactctattttcgatgattttccatttttcctgtttccgaggcaaaatagtattttctctatgttttccatttatgag

ctttggcatttttggaaaaggaaattaggggaagtcaacctgttaatcaagtaaatttccattttcccagttttggagaa

aatgcaaatattagtctttcttgggaaaggaaaacgagaatttccagaccgcatttaagcaaattcccaattcgatttct

gtatttgcccctataaatacttctctgttgatcaccaattgttttatcggcctttcctccttttgtttgcaagatggaga

gtgctggatcttcttcttctgttggcaaaaatgttccgactttctcgaacgctgaggcgattgaacgtgggatgccgttc

ttcaataaccttcgagttccctggactcttctattccaggcgagctcggaggcaagacgtgaagctgaggggtgtttatc

atgtgttgaatcaaggcataaggaataccgcgagatgttgaggtattatgatgaacgaaagtctgatattgcccgaaaaa

tggctatgctggagtcgcatcctcgccattggctcaagaatggttttcagcttctggctccgatgccggacatcattttg

tcgtactgtgaggattgtgctcgagatgctgctgccgcctcaaaggagaagaatccaacaatcattgctgatggggactt

cattcccaggacgccttctctattctatcttgggccaggtgctactgatgctgataccaacgagtgtcagaacttcgtca

accaggtaaggattactttgattcgtgtcttcgattttgggatgatttttaagattttcgttgctttggtttcccaggtc

agatttctacatcaggatcaatgccgcgagctgtagtacttccgaaccaagaaacgaagagtggattttcgtctcgagga

gctcgaaactctgattgctaagctgaagaagaagcgaagaagggtcgtcattcgtctggatgaggtcgaacagacgatta

gggcactggagattcgctcagaggattgggagaaggttggtctgcagctaatgtggcccatgccagatcgcctccttcag

gataatcgcgttgtttctgacagggcgatctgatgtcctatctggccggtagctctgctggatgccaggatgatagaaga

tgatccttagtggcttttcagatcgtttttccttttgtctttttgttttcgaataaacgcctttgttatggctgccgacc

ttgtttaattctctaatttattctttgtaatagctcccgatcttgtttggtcgttgattaatataatacaacttttatga

attggcttgtttcccgtgcgacctttgaggtcggcttttgagttctttttggagtaacgcaattttttctgtctaagttc

ggattagatgttaggtgcgagactgggctcggcttttgagagacaatattcagcgtgtgctggacgcgagacgtctctcg

gcttatgtccagcaggcctatgcatgttttaaagagggagttctgggctctgtttcaagaataagtgagacggactcagc

atattatttggagggcgagatgctgggctcggcttactccccgtagagcatttgttcggcagatcgcgaaatacagggtt

cggcttatatttctgtccgaaccagagcttaagtgcgagaaaactggtctcggctatccctattagaaaaattccattga

tacagagccaattccaatagacttatgtttctaatagggcgtggattatttcaccactgcatccatgtgagacgcgcatc

gaacacctggccagggcgccgagagttgacataacagatcttaaagcgaactccgtattgagaatgcaaactttaatttt

ataaagtattatagctgcatccttgcgaaggactgcttacgtacccgatgaagggatcaagccattcgtagtttgtatta

cataaaggagatcgacagacgatctgcatacatagtatattgttcttttttttttttttttttttttttgagctctactt

agctaactcctagcctagtgtagtaccttttgaggtgggctgcattccacggattttccacctctttcagcttcatggtc

ttcagcagatagactcctggatgaagtaccttggagatcaagtaaggtccttcccatcgagctccgagctttccggcgtt

cttctcttcggtgttactgtaaacttcctgaagcacgaggtcacctacgttgaagcgacgttctcggatgtttcgattat

agtatttcgccgctgcgttctggtaattctggatccggatggcggcttgatcgtgaagttcttaggcaaaatcattgtgg

tcgcccaaaagctgatcgttcagttcatggtcgtttgacatcatgcttctccgtaaagtcggaactccggcttcgtttgg

agccattgcctcgatgccgtaagccagggagaaaggggataatgatgtaggtcgccgaggcgtagttctgtacgaccaga

gcacgccatccagctcgtttgcccagttcccctttttaggaccaagccttttcttaatcccagctatgatcattttgttt

gtggcttcggcttgaccattcccttgtgggtatcttggagtcgagttgctcatgcgaatgttccatttggcgaggaagtt

tctagtaatgagcgagacaaactgcttcccattatctgtaacgatctcataaggaagaccgtgccggcaaataatgttct

tccagatgaatgcgacaacatcgcgtgacttgaccttgtggtacgcctgtgcctcgacccatttggtgaagtaatcagtc

aggactaacagaaatcttttggatccggaaggaggcatcggtcccacgatatccatggcccatcgcatgaatggatatgg

caccgtgaccgaacacagtatctcgggagggacgttcttcatcgggccgtgcctctgacacggctcgcactttgccgcgt

agacctcgcaatccgcagcatagtgggccagtaatgaccatcttttttgattttaaggcagagatctcgcccgcctgaat

gattaccaccgtctccgtgatgcgtctccttcatgagtgtcagggcttctttttgatccacgcaagtgaggaaagctttt

gtcgcacttcgtctgtaaagttcgccatctagcatcatgtagttcgccgctttggtttttagtcggcgagtttcccatcg

atctgttggaactatgccgtcggcaatgtacattttgatggggagtcaccaatcggatggttccggatcctcttctatac

ccggcgcgttgattgcgttgccgtctatgtccatggccgttacggggtctacgatcacgcaggtcgaatttggtggctcg

atgctcggttttgctatgctttcgacagggattgttcgccttaaatcggggttggcgcttgaggccagtgcggctagtgc

gtctgctgctgagttgtcgtttcgaggaattttgaccagggcgaactggtggaactggctggaaagttcgtggaccagtg

agagataagcccgcatcctttcgttcttggcttcgaattccccgctgaactgactgactacgagttgagagtcgcaaaaa

gctttgatctgtctagctccgagtcctttagcgagtcgaagtcctgcaagaagtgcttcgtattccgcttcgttactcga

tgctttaaactggagccaaagtgactgttcgaggatttctccggttggtgactgaagtaaaattccaagaccggagcctt

gtaatgatgaggagccatccgagtaaagtgtccagttttcgtctagggtaacgtcaatcacatccggggacatttcggtc

atgaagtcagctaggacctgagatttgaggctcgggcgagatcgatattccacgtcatattcacttagttcgattgccca

ttttgcaattcgtccagactagtttaggctgtggagcactatgcgtagtgggtggtcagtgaagactactatggaatgtg

actggaagtaaggtcggagtttccgagctgcctttatcaccgctaatgccagcttttaaaaggtcggatacctagtctcg

gcatcattcagagctttactggtgtagaagataggtttttcgttaccgcgatcatctcttaccagtactccgctcaccgc

cgaacttgacaccgagacgtagagatacagggtttctctgacctcaggtttggctaagatcgccccactcatgaggtagg

atttgagctcttggaaggcgttttcgcatttgtcgtcccagtggaaatccttctttcgctgcttcttctcgattacgtaa

cacgtctgtgagaccttctggttaccgtacagggtgaagattcctgtgtttatcgggaactttaggcaaaggtggtatgt

tgagggaattgccttcattgagtagatccaaggacttcccaggatggcattgtagattggttccgagtcgatgacgacga

acttgtttttttgcgtgatgccgcccacttggacctggagttttatctctcctattgacatttttgcttcgccgtcgtat

cttatcggtgggcggatcttaggcattattttgctctaagagattcccatttttaaaagggtcttcaagaacatcaggtt

gaccgagctgccgatatcgattaggactcgttctacgtcgaagtcctccattaccagtttgacgaccaatgcgtcgttgt

gaggatgccgaatcccacttgcatcttccgaagtaaagatgatgggttcctggcttaatggctcggcttctggggcgacc

ctaacgctgcatacttgtcgagctcgcttcttcaaagccgagactgaatcttcatcgtcagatagtccggccataatcat

ggaaatccttccgcggggatggttcggagctcgttgcggttgctgttctctccatctttttgggggaccggggagttcgt

tctccgggaccctcacttcttctacattatcgtcctctaggagcaattggtgatccacgtgtggctcttcatgcgttggc

tcagcctgggactgcttggagtcgctcttgcgtttatttttcccattcttgggatggtagaccgcctctacttctccaga

tttgtacttcccgaggaggatgttcaaaagatgtttacactcttttgtggaatgtcctccgaactggtgaaacgacagta

cgagttcttcgagccattcggtttggccggctgttgaggtcggtcttccgaatcgactgcggcaaagatcgttcctcgct

tgttcgcctttggatcatagtgttgacgcggttcttgataatctccccgctctttttccttggcctttggcggttgagat

ttgcatgccaactttgcgtcttcctctttgaggaaagcatatcatgaagctctatgcagggcgtcgtcgagatatcgcgg

ttctcttattgcgaggtcttttctcagcggggatccttggagtagtcccttcctaaaagcagccaccgcggcttcatctg

tgatggtaactctagacagtatttctttgaaacgacctagatagtcgttcagtgtttcgccattttcttgagtcatgctc

caaaggtcggaactagaggcacctggttgaataaatacccgatattgtttcaagaaggcagttgacaactgggtgaagtt

gtcaattgagtttgcctctaatctggaaaaccaggttaaagcgtttccgaagaggccctcgacgaagagctggcagcagc

cgatatctcggatttcgtccaaaaagtgagctttggcgactgttgtcatgaaggatgtcatgtattgtttaggatcgctt

ccacccttgtagattggaatcctaagcttcacttgagggattctcgtacgtgtaacccgatatccgtgaaaggagtcttc

tgggtttcttcgatcattcggtcgatttctggggccaaactgctggctctatggacttgggatccgatttctcggagttt

caaatagatatcatctaactttccatcgtgatctaaggttgcggatctggagacatgacccggcgacgtcaacggaggca

taggcggtctgccttcttggttgataggatggagtgatcctgggaacctgggtttcatcctattggattgtgcatccgtg

aggtcgaagctaagcccgcgcggatggatgattctcggcagggtcacttcggagctcggacgttgggagattcctttagc

cagctcgtcgagtcggcgattagtctccatgaccgattcagaaagatctttcgtcacggttgtgaggatggctttgaggt

cctccaaggagaaagatggtgggtctataggggtcgccccgtgtgccggttgattcgcggtcgctttggccgctacttcc

acagtagatggtggatctcctccattgaggagacttgactccgtgccatcatcgttgactggaggaatgacggggtcgat

gatgatttgaaccatgcttgctttgattaaaggagcgtagagaaaatttgtttatattcccctacctggcgcgccaattg

ttgattctgtaatcaaacaatgaatttgtactaagagatttaaacaaattcactcctctcttagctcacaaataatcaaa

ggaaaataactatgttgcttgaacaaaaagctagggtttgctctgtaatagtagtctcttgtattaggttaaagatggtc

tcaatggggggggggggggttgacccctctatttatacaatgggtcgaatataccaaaatatgataaacttctaattatc

ctaggtctaaatcttcattacttctattcttcataagatcctcttctcttaaatggaagttttctctttacacgacctaa

gaagttggttatggcgatcttgattgtcgacttaagtcttcgggcgagctcatgacttaggcccattaacaggctaaccg

ggataccccggttcaatcgacttctcgattttattcacattattccttggtttaaattggtattgtcggaggtgttaatt

cctgcaccaacagtaatcccatctcccatctcaatataagaacttaactctaccctttgcattcatgaatcttttaaacc

cttgttctaatcattctttccttttctcttaactctaggagaagctttctcaacactttgttacatctagcggatttgga

tttctttaacaactaaggttcctgtttttaacatttcaaaaccaagggcttttgcccatctccaatagctaacaagaact

ctttcttttctttacaaatcccaaaacctttactagactttttctgcttctttagcttacttcacctagagtcttttttc

ttttcttatcaatgaatcaaccccctcttttttttttcttttaaacacatcccaacccaaagtataagcgtccacctagt

cctatctagtccgaaaatgcgaactagaactacatgaaacactatctctgtcattacgaacctaacactttttaccaagc

ttaatcggaataagacctcacaaacactcacaagtgatatagggcttgaaagaaagtctaggtttgggtttgggttaact

gctctaataaggagagtcagctacttggattaacaagagtgatcaaaataaataagaaaatccaagtattttagggtaac

catgagttcaaatctgatgcagacatgataattaaagatcagattcaggttcaaattgatagggaatggtatcagatcat

gacagtgtggtcgagtagagcaatctaggcatggttcagtgttatttcaaattcattgactacgcaacagacaactagca

acgagggcactgagtttatcaggtaaatcaaaatgcttacaaggctatctcatcatcatcccctatgcatatgcaacaat

cctaaatgaaacactctagactcgatcctaaatgtaatgcaactacatgaacactctttttttgtgaaatcattttctga

aaattttcaaaattttttggtttttctatgccttagatgaatgcagactcaaaaatatatacaatgcaacacacactttc

tgagccctcccccaaacttaaatcacacagtccactgtgcgaataaacttggaagagagtactcaggacaaatccaatca

tagaaacaaaaaatgaagaaacaaatcacaacagatgatataatacaatggtgaagtgaggcgcttacctcagttgaaga

atgaacgtagttgctcgtcaatggctgcctgtgattgctcccaggccatagacgaaccaccttgttctgtatcagctcca

ggaagtgggtactcgacctcatcagctctctggcggccagcaccgctctgaggaaactcgacctctctccttctgctgcg

gccagcaccgcggtcgagtgaccttctcaagtgaatgaggcgtgatctgctgctagatcgagtgagtgtagccttccttc

ttctcttgtgctcccgggattcgaacgacgaatgcctagccaggacatgtgatactctctgctcgctaagctcaggcctg

tgctcaggctcatcaaatctccttgatggcatctccagaggaggctgtccctgtggaatagcagtggtggaggaggaaca

actcagcttatctgtcagaagcttgatagtcttgaaacacttggcgagcagcttgtcctgcttcttgcaccacttctaca

gtttactgttatttttatgagcttcagtcaagctcttgctctgcctcgctggaggtacatgttcaccaaagtgatacatg

ctagtgtcgaacgccactgccctatcttcatccatcccatccgaagcggcttcttcaagaagatcgtcctcatccaatgg

cggagcgttctcatagtagaggcgtccaacctcaggcctaaaatcaatgttatctccctctattatccttgtaacctcag

ggctagggagaaggatgttagctctcctgtctgtagagtgctcaaacctgaacctgtggaaatcgtcaaccattgcaaac

tccaggaattcgcagtgacgtaggtgctcgatatccattgctcgtggctcgagtccagcagaaaggattgggactccaca

agctatcaaaatcggtgtcacaacgccacctatgcacagagcgcctcgtgctctcttgcgattattgctgatagcccagc

tcttgtatccacacagatggatcagaagaagtatagagagaggtgagtcattgatttcaccctggagggacatgccattc

ttagtttggcggagagttcccttgagggccattgcgatcatttccatatcagagttagtgacagtccctgtaatctctcg

ggagtagagtacattggctatagaacgctggaagtacctgatgacagggttgcgtatctgattgctctttgacctggaag

tattcaacggtacaaagctgccaatggtgatccacaattctttcaactcttctctttcatacttgggcttagatcccgtt

ccactggggaagccaaacaatccttccaagcgcttgattgataacctatactcatgaccatacacagaaaatcgcaagaa

tcccaatccttcacaatccaactcatcagaggtcataccttggtagagctgtactcgcagcgtggagaggaattgtattg

tctcatcttcatacgctacatacggataagccatcagagtgtccagatgacaactttggtacaggtgctgaacatcttcc

aacaatccgagttgtgcaagtgaggtcgagcacggatacctcgtgctacagaactcattcagcttgaaaagcctaacata

ttcctcagcaggcaatacgtcgggcttgtgcagaagtttggtagctctgcgagtctgttcaggcatgtactcgtcttcca

gatcttcgtctataagctcatacctctctgtcatagctctcttccctcgggctatctcagctttccttctgaagctttca

tattctctctgttatctctctggccttgaagatgacgactctgtctcatccatgttgtagtcaggatcaacagaagaact

gccactgtaattgctcatgattctcctgtagagatttagaactcaaagctcaaatgtcaataggttagtctcaaagatag

caatgaaaccaaaattattgtcaataggcaatgaaatagattgaattggtcgagtacaattctgtactcgagtaacctga

gctgttcctgagcttagacgaattggatcgagtatcttctcagaggtcgagttgtaatgctacagcaggtcgagtgaatc

gtggaggtcgagtgagtcgtgaaacctagaggtcgagtaacttctctgaagttggaaggctctactgaattgatgaacaa

gaagccgagtagattttgcaggggtcgagtgctttctggtggtcgagtgagttgttgaagtcggctagggcgacgtcggt

cgaatgccgctatgagaccgggaaacgaggtcgagtagcttcttttgaaagagacggcggttgatgtggatgaattggtc

gagtatatcaaaggaatcgaaggaagtggtctggtcgggtgacggagggaatgagctagtgacggcggcgcggaggtcga

gtgggtttcagaggtcgagtgaaattctcaggatcgagtagctctttggacggcgattcgtggtgtagagagattagagt

gtcggcggtgaaatgaaaagaggaaggtcgagtgaatttcgtgaagtcgagtgatgcttcaaaatgagtggtctaggttg

agttttagattctctctccagcttatcctcaacgcgtggtccacactctgttgactgagaaaatttacagaaaacctact

gtaaactgcaggaaatttctgcaagtctctgtagcttctgttggtcgagtaaaatgatgatttaaaagtttttgggtttt

aaacatcacacatactcgaccaatgtactcgagtgcaaggtctccaggtgcaacagaagattttcaaaattttgtactcc

aacgggctgtacactcgatctccacgtcttctgtagagtaaaagttttgaatttttactctatctccacgttgttctctt

gtctctgaaaaaccccaaacacaactaaaacaaataaaaaataacaagaaagtaaaaattatttccaaggatagtcatgg

gacttcctcccaagtgagcttgtttaaagtcattagcttgactcctttaatcatcaagaagctcctggagatgaaccgat

ggcacctctggaaggatttggtctgctaagtatttcttgagcctttgaccatttactgtaaaatctccactcttaccagc

tagagtgactgctccataaggacggacctcagtgatgtagaaggggccagaccatctggatttaagttttcctggaaaga

gtttcaagcgagagttgaatagcagcacctgatcaccaaccttgaaatccttaatgatgatcttcttgtcatggaaaagc

ttggttctctccttgtagattttagaactctcataagcttctagacggatctcatcgaggttacttagttggatcaatcg

cttctcctcagcagtttttatgtcaaagttcaaaagttttaccgccaacattgctttgtactcgagctcaacgggtagat

gacatgattttccatagagaagattgaaaggagttgtacctatgggggtcttgaaggctgtcctgtaagcccataatgca

tcatctagctttacagaccagtctttccttgtaatcccaacagtcttttctagaattgtttttatctccctattggagat

ctcaacctgcccgcttgtctgtggatgatccaaccaaatagcttctgaacttctcaaatgcgaatacaacagctagaagc

tccttctcagttgttgcatatcttccctgagcgtcatccaacgttcggctggcgtaatatatgacatgaagctttttgtc

tattttctggcctaaaatagctcctactgcgtaatctgatgcatcacatatgatctcaaatggatagtcccaattaggcg

ctcgaacaaccggagcagataccaaagcatccttgatggtttgaaaggatttgaggcagtcctcatcgaattcaaattcg

gtttccttgcacaatagtctggttaacggcttggctatcttggagaagtctttaataaatattctgtagaacccagcatg

accaaggaaaacttttgatgtccttcaccgtttttggtggctgcaactgcatcaccacttcaacttttcctttgtcaacc

tctatacccttctctgatatcttgtgacccaacactatgccttccttcaccatgaaatgacacttttcccaattgagaac

aagattcgtctcttcgcacctggtcaataccctgccaagattcaacaaacatgaggagaaagaggggccatagaccgaaa

aatcgtccatgaaaacctcagcatctcctcgattaagtctgaaaatatagaggtcatacacctctgaaatgttgtaggag

cattgcataaaccaaatggcattctcttataggcaaaagttccataaggacacgtgaaagtggttttttcttgatcatta

gggtgaattggtatttgaaagaaaccactgtatccatcaagaaagcaataatatggatgattagccaaacgttcaagcat

ttggtcaatgaatggtaaaggaaaatgatctttcctagatgcatcattcaacttcctataatcaatgcacattctatgac

cagttatatttctagtagggatcagttcatccttttcatttttgacaacagtcattccaccctttttagggacgcaatgc

actggagaaacccaagtactatcagagatagggtagatgacaccagcatcaagcagtttcaaaatttcttttttcactac

ttctttcaaattgggatttaacctcctctgtggttcaatgctagaataggatttgttttcaagatggattctatggttgc

ataaactaggtgaaattcccttaatgtcagataatgaataaccaatcgctctcctatactttctaagcttagataatagc

aggttcacttcatcactatttaactcagcattaatgatcacagggtaagtagagtttggaccaagaaatgcgtaccttag

accttttggaagtggtttgagatcaaccttcggtgccttaagttcagaccagtcatctgaagttggaatgatcggctccc

tagattcgtcggtagacaaggtcgagtggttgctcgagtacgtcctcgagagtgcaggttgaactcgagttgactcttct

tcgctcattaccattacctctgttgctggtccattcaattcctcaaagggttctgattcttccatcgctttatgggagtc

taacagcttctggtatcccaatgtttccaaatgtagaaacccatcttcaccacttttggttaaaccactgttaagatgat

tatcttcttatgccagctcttccagtaactcatcagctaactgatccatttcttcgatccaaaagagttgcctttctatg

gtaggcttcttcatcgcgtctttgacatcaaaggtcatcctaaagtctttgccaaggtttagatcaatcttccctttctt

gacatcaatcatagctcctgtagtggctaagaaaggtctccctagaatcaacaggtccttgggctcttcatccatctcca

ggactacaaagtcagttggtatctccacaacaccgatcctgattgggaggttttcgagcaaaccatgagggatccttgct

gatctgtcagctaggatgagggatatattgcaggacttgtattgagtgaaccccaatcttttggcgacagagagcggcat

gagactgactgatgctcctaaatcgcataggcatctattgaaagccaatggacctaaagagcacggtagagtgaatgaac

caggatcactaagcttcaaaggaatgatcttcttctgtatgatagcgatgcattcatgactcaataccaccatcccttgc

acttcttgaattctctccacgatcaagtctttcagaaacttgtgagagtctgggattagcgctagagcgtcaacaagagg

tatccgcaactcaacctccttaatgttcttggcaaacatagctctatatttgcctgccaacgctttcttgtgacggccag

gaaatggaagctgcggtttgtagggaggagggacaaagaccttttctttgtttttgatagcaactggttttggagcagtt

ggtgatgctgctgggatgacagatccagtggttggtcgagtaacgctgtcgagtggcagatcgagtggttgctcgagtga

caggtcgagtggttgctcaagtgacaggtcgagtggttgctcgagacttaaatcctccccatcttgatcttcactgtcct

cagtgaccgtctttggttccttcctagttggaagtgcttttccactacgcagtgtgatagcatgagcatattcttttgga

ttctgaactggtttgcctggtagttggcttgtctattttggagctgaagaagatacagaatgtccctctaagtaacggac

tttggtattcagtgtttccatcttgacattgagatcattgtagctacagtccagcttgttctgtaactcaaatatcttct

ttgccatctccatggagtcagaggcttgcccgtgaagcagctgttgtagcatttgtttcatttcagcaacaggagcagca

ggacatggggcagattcttctcccagatgtgtgctttgtctcccaaggagaatggaaacaaacggagcttgaattcgtct

tcactgacaccattgatctttgttaggttgcagaacctatcgaattcatcaaggtggtcgagtggatcctccattggcag

accatggaatttgttcccctgaatcatcaagatgagaccactcttaatctcgaagttgttgttctggatagcaggaggtg

caatttccttcctctgacggtgatcacgtggtgcatctctagcaccaatgttggcaaggccattttgttgattctgttcg

tcagccatttcgagtggttgctgaggtacaaggttaactgtggttctcctttttctgagttcgcgagttgtgcggttgat

gttgtcgttgaaaatgaggttctgattgcctgttgatcgagttcgcatacagcagaggtatacctggatcaagaagacaa

caagtaaaacacacagttagtaactgaaaagaaaattgaaaatgaacttgatctaagcaagtctgaaatctcaaatgtaa

caaacaaacacccaattggcaacggcgccaaattgataataggtttttggtcaattatcctaaaacaccaatcatatcgt

tgtagtattttaggttgtcaatccaaatgggtgtgatgctaacaataaagatgtgatcagaagtcactaagtcaagccaa

gcaataacaggttttggtgtgttctagcagttctaagtgaacatgcagaaaacagaaaatcaaacagaaacagtaaaaca

ctcgaccaacacagctctttgcagagcactgggtgaggttcgagtgacaggtcgagtaatagacagaaaatgaaacaggg

atactcgactgcacagtctgttgccagacaactgggtggtcgagtatcaggtcgagtaacagtaaagaaatgcagaaact

aaataacagatgacaagatgcagtaaacaacagtagagtaaacaaagaatttaatcagataaagaattcccgaggatggg

gtaatcgagtagcttcgttttctcagtttacaggtgattgacatgctcaataaattaaccctagacaacaagttcattaa

ccaaatctaagtgccaccgcaacagagaccctcaagttaacctagtctcagactcaatcactattgacgagagctaacct

aacaggcattacgaatcaacaagttaaagccaaaacgctccctgcaaccaataccttggtacagggtcacgaatctctgg

aattagtggttcagacatttcatcgaacaccttttgggcgcggaaatgtttgggctcaaattccagttgatcagaaagca

ataggcattaagaacaactaatccagaagggatctatcaatcaatactcaatcacctagcatactgagaattctacatta

ctctaacccatcctcagaaatctattcactactcagacatcataacagaaagcataaacgttgaacaagttaaaaacatg

ataacaacagtgtaacagcaggatgataacagaatgaacaacagtaaacgaaatcaagacaaaagctgaatactcaatac

ttaatagataaagagaaatccggattacaaaaagactagaacacaaggtctgcggaataaaactaggtaaaaacttcttt

ttgcgaaaatgtgaagtacatgctacttatagtcaaatatcccaaaaccctaatactcaaaacgacgaagtattgggacg

gataaagaactgtactcgacccatgtggtcgaatgggaggtcgagtgagtagttggagtcttctttcctttcctgtgctc

gagtgtctggttgagtgcggaatgaagaaggctctgaagcttgtcatcgagtatgcagtcgagtggtgtgatggtgatac

tcgacttccaggtagagtgatgaggtcgagtagtggtctggcaatgaaggtgaagacactcgacctcgtggtcgagtgaa

gtgatcgagtgtttgtcaggagatactcgacttccaggttgagtgacgaggtcgagtggttgtctgatgatggaggtgaa

gacactcgacctcatggtcgagtgatgtgatcgagtgatggagatgagggcacccgacctcgtggtcgagtggtgaagtg

aagtggtgatgtcctctactcgacctgggggtcgagtggtctcttgagatcttgaagctcctactcgacctctggtagag

tggtttgtcatgatcatgtccgcttcttccagatagctcgtcaacagctccaaatcacctaaaatcaacgcaaatatgca

acatgcatgcaaaactatcctaaacatgtaaagtgatgacaaatgatgtgaaatgttataaaaaaagtgatgatatgatg

ctaaagtgatgacaaatggatgcttaaaacatgcaaaatacacacttatcaatcctgaatgtttccaagaatgatttgtc

acaaagtttttaatgttttattcttggtacccaaacaattattttttaaaatgtaaaagataatcatcaataattatatc

tatgctaaacatggtcgaaaatattaaatcataagcatagtagtttaacagaaataagtataactataactatactttta

tgtaatcgttgcaaactttgataggagtttgtttggagtttctttatgtcatatttgtaaactttgttaggagtttaaca

aaaagtaagatagagaaaaatccaacgggaatacaataattaaataggttcaaaacataagcaaagtttaacaaaaaaaa

caatttgcaatgacaaagtttggagaaacttaaaagaagttcaaaacataatttaaagaagaataagagctgccaggtca

aactcctaaatattttcaatcacttccatcctttggttctctggaacttcataataatcttcgaaaccgcggctgtaaaa

gagaaaaataaaaatttttattgattaattgatgttatggttaactaatacaattaaaaaatgttaaaagtcttacatgt

aacttcgaatatctatctcagggaaagtggttggattgatgtaaattcttgaacatccaaattcactcatcaaaacattc

tcacctgttacaaaatttttatttaaagttatataatacaaataaaaatcataagtaaataggattacaatagtattgaa

aataactaaccctcaatctcagcaattctccaaaacctcacatgcctctgaactgatacatttcgagaattgtgtaacaa

agatttcacagcatcttccaacagcatagcatttcattttctctcccctaaccaatatgataaaacattacactaagcaa

tacatttttaagctcaattttcagacggaagacacattactaaatatacttataaacactaaaacttacatattacccaa

aagatcgaaagagacatatctcgattcatagtcggtttcaccctggcgacaaacaaatgagaatttcttaatagccgaga

tatttgacactacacccaacattgcatctaagtatagaataatatattaatagagtagttcaaaaaaataataacataaa

ctactaacaaaaattcattctttaaatgagaagaacatagattgactaaaaacttacccgtgatataatcccaatcctca

agacatggatacttaaggtttatgggaaaaatgaaatcaaagaaatctcttcaccagttccggaagaggcaaaacacttg

tttcccaaatgaacttgatgtggaaacaaaacgaagaatacctaaccctttcttgaggaacaacgacccgaaagtcagag

atgtgaatccatgtgtcaggaattgcatctacacgattattctgaaaaaattcccagataactcaaagattaaataattt

aaaatgatctgtttcaataataacaaagtaaacttggattaaaagaacgaaacaatcaaaaatacaaaccttgtcatcat

gaagaatcatggttgtaatttcaccatgactgtttggaggatgattccagattgacaggatcttcacgttaatcttccaa

ttagtcgagttatccctcaaatcagtgagtttgtgaacagacatcatgtttatccaaggaagtttttttttttgtttgaa

agataagagttaaacctaattgttttaggcttgaggtttttgattttatataattacgaaaagatacaatgatggacagt

tgatgtgtaaaaaggaaacgaattgtccacaaccaataggatttcttataggttagaacaataatgttgatatttatttc

ttagcctgttactaaaaaattgtaattatttaaaggagaatgtatgaaaaaataggaataggtttgtttttcaagattct

catgattaatcatatttaataggattgattatttagtgtacaaaatctttcctaattctgattttgttggttttttttgt

ttaaaatgaccaaaagtgtttatagttctccgtctgattaacattgtaatataaaagaatatgtttcttaatcataaata

taactaaaataataaaattgtttaacaaaacgacatataaatgaaattttttttattaattccaaaacaacaaataaaat

aaagatagtttaaaacctaaaaaatgaaaacaacatcgataactttaagcaaagaaataataatgaagaaaacgatccaa

gatgaagtagtcattaagcatcattcaccgaagccttcctcaggcatggttatcttagaacacttcttcctacttgtgga

agcctgatcatttccatcaacatcatctaaatcttttcgttttgatgatggaatagaggttgatgacaaatccacatcag

aaagatcaacaccattagtgagcataagggaaccttatatgaaaatacaaaaaatattttaataagacaaccataaaata

taaatataaatgattggaattgtttaatgttattagacaaacctgatcgatagaaactatggatgatggatcaacgtatg

catcagagtcttcctgtatgatttcatcagcaatgttaccggtgtagactctggcaacaatgaatgaatcatatccacca

aatatgttttgcctttgaatacacaatagaaactgaaaagtcatccctttcaaaccattgatcacatcaggcaaaacagt

tgggtcttcgatctacagaaaaaatctactctattataacatgaacgtagtcattttaatgaaagagtttatttttaaaa

aagcgatatacctcatcaaatgaaccatcaagcaatttaggggcagtagtgccaagaatatccattgcatgactatcgaa

taacatgcatctaacttccctagtttgttccataatatggagatgtaaccaaaacctaagagttaaataggattattatc

tcttaaacaccgaattataattcataatacattcaatataaaggtttcacaaaagactcacttggcaacaacagattttg

caaaaccattgcatatgtcacaccaaaatcttggcttttgaggtatcttgacatcttaaagtttgataacttcctctttg

acaaccttcttgttgtgagcacgacaacaaaaatagtaccacggccaatctgtatcaatggccgaaacagtacaaattgt

tttgcatttacccaccttcaaatgaatataaagaggatttcacaaactaagttaaaaaaagaaaaaaaaaaagaagaata

aaaggttagtagataaaataaattcatgaatagtacataagtagctgtaaggacttccttgattatttgccttggataat

caccatcgtcatccccagttcttggtgcatagagcctcttgtggaaagatccagaactttctaaagtcaacccatcgatt

ggtatgctatatataaaggtaatataccgtgcttaatattataaagaagaaaatatatttagtgaaatagaaatacaatt

acctttgcttaaaaagacccaactcgtccaatgtagaatcaacgaagacttgtgaaacatcaaatgaattggaaacactc

ctattatctgtgtataaaaaaggacatgttttaaaaaacaatacatatatgtagtagtacatatcaatataattaccgtt

gtagagattgatcttcgcaaatcggattaagcaaacaacaataccatcggttaactgctggcaagcttggtcaccgatct

cagcatacttcccccaaagtgtacatgccaagcgagtgtcacttaaatgaaaagaataaatttattagtaaatagtaaat

aaataataaacatctaaataagaaggaatatgaatatagattgttagtactgctgatctctcaaatggaaatcgattttc

ttggtggacttgttgttagcgttaagatcttggatttcagaccgatcaacaacctataccattacatctatatagacaaa

gagaataaatctatgaatatatagactatagaagagataatgcatgtatggtcaaaattaatatataaattttaatataa

ccgtacaagatattagcgttagcggacccagagaggatcacgtcaaacttagcaagggagagaaacaaagcattagagac

gctatcacatggagagacaatggttttgaaaacaaaaccaatcttgtattttaagtcagaaatacgatactgaccacttg

taggatttaaagtgatggtcgtaatgatcttccaaacaccctcggttaagcacctttcaaacttcttgatctgctctctt

ctcaaagcagcatacatcttattaccctataagacaaaaataatcattaataagtgaaaatgatgcttgaccacactata

aggctggacaaaatataatgttaaaactataatacgtacagcttcatcaaccaaaatcttttcgatagattcaccggcct

taacagaatattacctccaaacatgaagcaatttaggggccgattggtaacgactcttaattttggttgtaaagacttta

actgtaaaaatttgagctgtagagaatttagttgtagacactttggctttcaaaactttagctgttaaagtctgattggc

aacagaaagctatataagctgtaaattttattatttattattctatattaattacaaacaaatttgatatttaaatatta

ttttttttctaacaaaccaataataatttcaaatgtagattgttatcaattattttcttagtaagtttcaattttttttt

tttttgttagttattgaatgattcttgatcgattattatcactaataataataaccttttttaaaaagataatatttttg

aatgattctttttggttgagtagttgtatagttttaagcagcattaaaatatatttcattaagaacaaaaatagctaaca

ggaaaaatatttaatgaaataattgaatgacataaataacgactgaattatatatcttatatagagatctttacatatgt

atgtatgtatgtaaccattgattcatgctatgtttattatttatgtatatgaattctgaagaatactatttagaccaaca

aggatgataaaaataaaatgattatttgttaaatggatctttcatttttaaaagaaagagaaaaaaaaaaaattatccaa

aaaattgtgaaacaaaatatatataaatttgtagagattttttaattattatggaagaagaaaaaaacaagttttctacg

catttgttttaacaaccgtgtatttgagggttttaaaaaaaaggagaaataaagaagaaaaattaaaaatggctttaaaa

actttaaaaaaaataaagcataaaaagtttgattggtagaaaaaaaaactgtgaggactttagaatactcttaaagtcct

ggaagtcatgtgccaatcaccccctttaacctgaatacgccaagcgtttttgtaaggcttaagcgatttcaaaggagtaa

atggaagaggattagcgagcatcatttaaaaaaaacaaaattagaaatgaatgtatgagtctaagatgagtgagtagatt

gaaatgaggggtccgatatttataagaataagtggttaagtaaaaaacaaatacgcaaaagatattattcctgaaaatca

agtttattccatatcacaaaatctcataattataggaaaatgtttagcatttaccgtaaaagtgggtatgtatatatgga

atacgtgtggaaaatgtaagataattccaaatgaagatccaaatggaatattagatgtgctataattatggagaagtgat

atattcgaatgctggaaacgaagatacgcaataaattaaatgaaattaatacgattcgttgcctgatatatagtcagata

atttttttgcatgaaactacaaaatcgtacggctagtaattaatctattcaatggctaggattaaacggagaatccgact

tttttaaaaaagaataaaaagaaccgcgttttttttttgggaaatacgaaatcgaaaggctaaaaacaactcaattcaat

ggccaggattaaaggcgagtacccaaatttttaataccaggtaaaatgatccgcgtgttttctaagtgaaactgcgaaag

cgaacgactaaaaacaattcaattgaatggccaagattagaagttatgacccattattttaaaatctgggtcaaaaggat

ccgcgtgttttgtaagtgaaaccccgaaatcgaacagctaaaaacaattcaattaaatgaccaagattagaagttatgac

ccattattttaaaatttgggtcaaaaggatccgcgcgtttctaaaaaggtttttttcatgacataaaatttttaacctta

tggcaaatttattattttagcgaaattcatgcatactttattaaacgtaattatcaaataatagagtagattataattga

atagtataaacatattttcattttatttttgtttttgtttaactaagctcacagcgacacaattccgaatattagcaatt

ggagaaagaataaaagacacagagcaccatattattcttttagcccaaaaatttccacaagtacaaatccggcaaaaaca

catacatagaccaatttttggtttactgacgaaattttcagatcagaaaaccggatttgattcaagtgggtccctaaacc

gtcggttttaaacaatattttcaacgatgagctttttcaagtcaaactagcgaaacagctatcagacctagacaaaacat

tagaaacacaatcatgatgattatcttgatcgtctttagatggactattaggcatagatctaagcattcttggtgaatct

aagctttttctcaagatctgtctcatcgcagagatcaaatgtcttgtcagattcgatggtgggcttgaggatttgaactt

attacacttgttcatgtgtctcgccatagcttcttcttctgttgttaaaaatctttttgttctcatgactttgtacttca

cagcttcagaacaaaatccagagatccatttccctatgtaacgctctcttatcatctctgtgtacgattctgtacactcg

tcggttaatccgcaacagtcacacgtcaccgaatctagctcatcgctggatatttgtgtcaccggaaccgttgagataat

tattacatccggtgttgaaattaacattgttggatcactaagtacactatcagacatggtttcttgatttaaaaccctgt

tgagaaaatttcgatcaacaatctgattcttacttcaacataagaaactataaagaaaccaagaggagaagttttatcag

tagcagaagatgagaagataacgcaataagcttgtgatatatcttctttgttttgtctctgtttctgaaacagagaaatg

gtttttaaaaagggattcttaaaagaaattggaatacatttttttttttttaacgttcgttttgccactctctgtcttct

aaattactttcatctccgagaaaaatttcacaaaacaaaaccattgcaaaaacagagtattaaaaatgctttttatcttt

ggcttttcttgaaaacaaatagaaaaagttaccgtttctatttacttcacggaaaaaacattagaagttcataaaaattc

actcgaatctctcgttacttcagaagagagaaaaaaaaaagtattgttttggaaatatatgaggaagagaaaatgagaga

agaagtagtgcaggcgaaggagaagcaatgtttggtgttttataagagagacgatagtagtaactagtgacgtggaatat

tgattttaattttttagttttattttatttattgaacattttatcaaaacgccgcgtattagagaggatgccctagttta

atgtcttggtataaaagcctatattttgcatttttcatccgactctctgcgttaggtttcattttagcggcagacgagag

agcaaaagcaaggagaaaccctaaccatggcgacacaaccagctactgaatctgtgcaatgcttcggaaggaagaagacc

gcagtggccgtcactcactgcaagcgtggatctggtttaattaagctcaacggttgcccaatcgagcttttccagccaga

gatcctccgattcaagatcttcgagccgattcttctcctcggaaagcaccgtttcgcaggtgtgaacatgaggatccgtg

tcaacggtggtggtcacacatcacaggtttacgccatccgtcagagtatcgcgaaggctcttgttgcgtactaccagaag

tatgtggatgagcagtcgaagaaggagattaaagatatcttggtgaggtatgataggactctgcttgtggcggatccgag

gaggtgtgagccgaagaagtttggtggtcgtggtgctcgttctcgttaccagaagagttaccgttaaacaaagctgggtt

ttttttttttcaatttcgattcatctcaaggtttttggagtttttgattatggacttgtgagactcttagaatttctgtt

ttatgatttattggataactgtgattctccaagaacttatgtcttatatgattttgtcttcatttcgttgcttcttattt

cttgcctgtgatgtattgcatctgcatagttggattgaaagttaggttcttgggtttataagaaggctgtgacttgatgt

tacttagccatagctgatcaaatatcgtctaacaaagtggtctgttactctgttatattggttcgcgtaaattttggtta

cagtttgtttccgagttggtatctcgtaagtcgtaacgtttgtgtacttattcactggaagcaagttgtattgttgttag

tcttttgttacctctcctggtacgattttttttgtttgtgaacaagatcagaaactgggactgtgaatttgaccgataat

caaagaaccaaataacttttgatccaaaaatcgagtaattaataaccgaaaagatctttctactaagttttttcctactt

cgacgtgatttttgacttgttgcaaaatgttatgctctttgtgcgttccaccacttccgtagtgaaagctcttgtatccg

taaagcaaaaagtcaacccttttgagtttttgtcatggcaatttctgcacatttttcattgcagccttgccatggagaca

ccatgtgttcccaagcttcagcgggcaagacgtccgtagaacctttctcagtcaccttctgaaggaattccggaacaagg

gaatacgcacgttcatggataatgatatcgagagaggccaaatgatcagtcccgagctcatacaagcaatccgagaatct

aggtttgcggttgtagtgttgtcgaagacatatgcatcttcaaagtagtgtttggatgagttggtatattctttataccc

agtatcatgattcatataggtaaagaaacagagttacacataaatgcgtatacacaaagatatggtgtttgtgctgattt

tgctctttacttgttgttttcaaacttatgaaaagagatatatcacagagtgtatgaaccataaccgtacatgtcccaaa

tccaaacaagtcctctatcgagtgtgtttgacgcccaaccatttgattaatgagttgattacgcgatggtgtcttgttag

caatagcaaatatgatcggccagccccaaaaccatctgatattgaaatagtaacggagctgttcacggatggcatcgaat

tgttgcttcagcggatttcctctccttcctcctcggttgcagatcagacagaagctgcaaaagagcttgccctccaatcc

tcgaaatttgtccaagtccgtgacttctttatcaaggaactttctgattcgatcaccaggttactcactccgctctctgt

tttgggcgatgaggtagactcgaatcccgagcttcaagagaatatcgtcacaacattgttcaacatgtcgactttcgagg

agaataaaacactacttgctgaaaaccatcaagtgatccctgtgcttgcaaagtttatgaagcagaagtcagttgtgaca

agaagaaacgctacattgacttgagtgtcacttttagacattgattctaacaagatcatcatagggaactcagtagcact

caaggctctgatagatcttatcggggagttggatgacttatcagctactcatgaagctctttgcgctgttatatacctct

gttgtgatgaaatggagaactggaaaaaggcaatctcgttggggttgactccttttgcagtcagaaatatcaaagcaaga

agaaattcgtttgactcgttagttatcttggcattgatttctttacacgaacgcgttatccaggaactggctaatgggtt

atctatgatctgttaagtatcttgattcttgaggaacaaaagttgcatggtgacttgtgagaacgttgtagtgatcgtaa

gcaacatgtatgttaaaagcaagaaaggtctattatgaaaatcctggcagaagaggagaatcaacacaagacatttacaa

agcttgcaatgcaaggatcacctgttgcggtgatgaaagcacaagggatttacattgtattagggtatttcctgctcgga

atgtgccacagagggcttgacagtatcatcaaaaatcaggtcttgttaataccttaatagtttccgcaaagttacatttg

atacatatagatgaaagtgtagaaagaaagaattcgagttgaaagtacaagaatttgagacttgtaatctagggattgtt

gttgcttatattgcttgaaatactatacgaaaaaaatactcaagattgaactttaacttcctccactcttatcttgttta

cattacatctttgatgtatatataaaaaaaaaatggaaaaatgtttatgagctacatgaagtataagccaacccatcggc

acacatgcaaactttaaaaatgtcttgttagctacacgaaatattgacatcacatacggacatacacgaacaaacataac

tcatgttgccaacaacaaatattgttcgtgtatgtccctatgtgatgtcaatacttcgtgtagctaacgaaatattttta

aaatttgcatgtgtgcgatgtgttggcttatatttcgtgtagctgataaacattttttcaaaatttttttatatattaac

taaccattgataaaaaaaaataaaaaagacattgcatggaattttcaattagagcatatagcttaagaatttttgttaaa

tcaccgaattataagaccaatcaaagtcgggtgtcaactaaagctagtgagaatgcggatatgtcgcggtagacaggtgg

cgtaacgtaattaaagataaatagaggaataaaacaaaatcatatcttttttgacattcttttttttacatctaaaaaca

aactcatatcatataatagtttcttggcttctctctaaattttaaacagaaaactttccctcctaattcaccaagtttct

agggttttcagttctttgtttgggttctttgtgttggtagagatatctccggctgaaatggcggattcgccacggacgag

tcaacaaacgccaatactatgcggcgggagttacacaaggtactgccagagatttggtacgacagaggaggcaaagatca

cggtgagatagataaggctattagtatcctgacttgtctaaggaaaatagaatcgaagaagccggaatctgatatatctc

tgataaaagttccaaaagagtttatatgtacactctcgaacaagatcatgattgaagctatgctcatcgcttctcgccag

gtttgatctttcttagttttccatgtttttgacgatctcttgtgattcttgcttcatttttcaatatttcatttatacgt

ataagcatataaagttttagtatcaaagaaaagaaaggtccatccaattctggtgtgttcatttatgtcttatgcaaata

cagagttaggatccaagacacatattctctggtgtttactctttttgtttttttattcagacgtttggaaagagctaaat

cgtagagtgggtgaagcatgaacgcactacaacaaatatggacattcttatcacgagaaaaatgctataatatgtcttta

atagctttttcatgaacgctattttagggcactgttattataagtaccccacattcagtagcgtgagttcagtatgctat

gttatagttttataacatagctataaagaacagttgtattatcacgtttaattttcttaaaaaaattatataatatttga

ctttttgtgctatgttatatatctctaatatagctgttgcgaaatgttgttttatcgtggtttgccttttttcaattgga

aaataattagtcaaattatgtgctatgttatgctcttatatcaatttataagctacgaaagatgattaaaacatagcatt

agtgatatgttaaatattctattacctgtattcttatagcattttctgagatgtgattatagtacacataacacataacc

tgctttataaaatctcatacattgacctttgattaaacataagtttagatccaaaatacataagaaaagtctcaagcaca

aagtaaagcaatacaggagctaagtgcataagttcaggtacttagcattacacaagaaaaccatcagtttaagacataat

gaccttgttctctagctaagcaatacaggagctaagtgcatcttccataatctctatctcatctgatggcctccaaactt

ttacattggccaccttcacaatatctatccacactttaactgcattagaacctaagggagtgtggtgaaccttatgtgct

ggatcatttgttgcccatcgtccttcagccacaactctattctttccagttaaatccatcaacttgcacttcttattctc

tttggaaataactttgttaatgcgctgccacgtatacaatataaaccatcagtcatcataattaacaaaaacaacttcat

aacatatgaaagcacttactggagacttcctgagaggagactatgctggtaaagtattccttattggagaacttggacct

gttgcatcatcaataggagattcagaaccagatgcagacttagaagacatagctgcagatttggactctgacgcagcctt

tgaagctgttgctgaagttttggacccgtctgcagacttcgaagctgtggctgcagacttagccttcgatgcagacctag

aaacttctgatgtagaattatgaccggttggagatgagggattttcaaaatcaactttgctcaggggccaggctacaaaa

gccatcaaacagtcctcaagatatgatatttcagctgtaggtctccatagtgatgtatcaggctgctttactgaatccac

aaatactttaactgcttttggtccaagaggaagtccattaaccaatgcacttggttcttgagtctcccaacgcccctctg

caacaatgacttcagcattcttcaaatccaatagtttacatttgtttccttttagaccctatcaaacacaatcagtaaac

aaatccaatacaaataatcagatgatacacaggggaaatacattgataacacagtgcaacaaattttaattaccaaaggt

gcaatgtcttcaatttggatgtctgtgtctagaagtttacacttatcagctggccatgcgattatatgtccaacagcttc

tttcatattaaagatcttgtgtgcaggtctccatagaaatgcatctggttgatatgcagtttcaactaacactttcaggt

ccgtagggcctagacgacaatcattcactatatcatcaggatctgaagaaagaatacgaccctcaccaacattttcatca

gtctcagaccaatcaacaaacacacacttacgatgtgattttctgttaacactctgcaaaaaaatctgagaaaataagag

tcataaacttaaataagaagaatcatttagatggtatgaaaagaataaggaatatgttactcttgcagctgagttttcac

ccatttcagcttctggcctctgtaacataaacaaaactaaagtcaacttatataactctaactggttgatgaatatttta

aaactcttaaagtatcttacctgattcttgattctgatgatttcatcttccaagtcattgacctgtttttttaatttcac

ttgcctctcttccatttcagccatacacgtgttcttcacttgtaaacaagctaatttggtcttactcatgcctctcccca

ttaccctcagcctaccaggattgtcaggtcctaaaagcttggcgaggtgatcttcatctggatttgttgaaaatgttgga

gcatcacttccaacaatttcagctgcttcttgctggaaaacatgaagacagtgtcaggttttactagtcgaagacacact

caatcaacacctctaatttagacttacaatcttttcagctgcattagtatctactggtgtaccatcctttttggtacgag

acttgatccaaacctgtagtcttgtcacgctggaaggatctccactttcaagtttctgtacaaaaaacaacatttcagtg

actgtcacaacatacaagcaataaccgtataataataagcaaaataaatgaagtagatatcttgccatttcttctgttag

tctgacaatccctttacggctagtggtatgaggtatctgtttctttcttcttgctttaaatgtatcactcacagcctaac

aatgaaacatgttactaatccccatttaaatacaaaactttatattcacaagtaattaccttgaatgctgaactggtttt

gagtttgacaaacttttgccactcaacaggattaatattagttggtctcaaattcatcctctcttggttatttgcagcca

agttaatagccttcaccctgcgtgacttgtgtgctctccacagatttcccatctggtttatcacagatatcctttgccaa

tcttcatcaagctcaaaccttgcctgcattcacgaacaatcacaccataattatagaagaaccggatcaattacagacaa

cttaaaaaaaaacactacttgttacctgaatagatttccacagaactgttttcagatctcgagtcactttcttccagtta

acaatgttgatagggacgtgttcccgcatcaatgtacctacatatgatgacaatttaactgaccctggaccaatagcttc

tcccattagattgtaatcaactttcactctaactgttggatccttagctaggtttttcatctttgttggtcctctttgtc

ttttacgcggctgctcatgtcctccagaaaattcaacctctccttctggctcttctgaattgggaacatctctctcagta

tctatagatggttggtgttcagttggaggttcttcatctatagctggttgcttttcagttggtggttattctgaatgatg

atcctctacacgctgttctgtagctacaggttccacaataccaagatactcaggttcatcatctatagacttcttctttc

tcttttgtccactcttcttctttcctttagccattttaattcacctgcaaaaaaggaataattaattagttataacttga

caattatacaaaagcatatagaagatctataaaaatcacacatcaaccataaacaaaagcatatagaagaccgtatataa

atgaatcaagaagttacatttggcacatatatgccttcacaatcagatctagcatattcagcttcatccatttcagtatc

catgtcaacgtctactggtaatggaccaattatatctgcatttccatcctcagactcatcgttactatatcttcttgaag

cacctctcataacaacagaccaatttgatccttcttcacttgaataaaatacttgctttgcttgtgatgctaggatatat

ggatcctttagaaagcttacttggctctgattcaagttaacaagagtgaacccatcttccactttaactccattacctct

tactgcccattcacatctaaatataggcacatagaacacattgtagtctagcaatatgatgtcaatgattctcccataat

atgataccaaatcaactacttgtgaagtatccttagcactagatctacacatagccgttgcctcataataaacaccacta

ttttgactctgcctttcaagtgagcgtgtgtgaaaacgttgtccattaactatataaccagtataggacctagcagtaca

tcgtggaccatatgctagccatttaactatgtcttcatgttcatcagaagaattagatatctgcaaaataaaaaattatg

attcagtcaagtatagatttataaacagactcatcattacaaatataatttacttgcctctccttttaaccaagcagcaa

aattcttagcatgcatagaccataaaactgaagcatcacgtctgcatcttgtatttgagtcttgtaaatattgtaaatgc

atactaccaaatcataaacaccttgagttagaatcagctagataaatgtaatttgatatggaaaattacacaaggaactt

actccacataatgatcaaaggcagctgtattttgaatgacagctagatgggctatatttttgtccatttcagtaagcgtg

aatgatgttcctgctgatattggacggccttctaatatagagctgttcacatactctacattcctgtctagcttttcttc

tacattggttgtctttttcagaaactcattacaaaactgcattcattcttctgcaagataggactcagctatacaaccct

ctagtcttgctgggtttcgcacaaaatcttttaaaactttcatgtatctgtaaaatgaaaatgtaaggattagcaataat

aaaacaataactagataatataagtgtttaaattactaatagagataagatttacctctcaaatgggtacatccacctaa

aatgaactggtcctcctaatctagcttctcttcctagatgaattgtcaagtgtaccataatatcaaagaaggttggaggg

aaaaacctcttaaacatacaaagagtttcaacaatttcagtttccaatactgaaataacctcaatgtcaataactctctg

acacaaataattgaagaatgcgcatagccgtagtatagctgttctaggaccttttggtaacagtcctctaagtgcaaccg

gaagaagctgctgcatcatgacatgataatcatgtgatttcagacctgatatcttacactctcctactgacacgcctcta

gaaatgttagagcagtatccgtctggacctttaaaatcaaatagtcgcttccagaaaatcttcttctcattcttcgacaa

ggaccaaggtgctgctggaaggtatgttctctttccttgtgtggtaggatgcaaagcgtgtcgtagaccaagatgttgca

gatccttacgtgcattaagaccatccttagattttccacaatgcaacaatgtggatacaatactgtgagccacgttcttt

tctgtatgcattacatccaaattatgccttactggtagatcctacaatatagacatacatatattttagaagtaatcgat

gtatcatacttaaacatataaaagctatttaaatctgaaaacaagtaatcatacctcccaataaggcaaagtaaagaaga

ttgatcttttcttccatctatataactcatcctcatctacttctacttcctcatcttcctcggattcactggatacatca

tcactatcatagactggttctttatagacagttctctttctcttcatcccagattttttcacatttccaaaatcattctt

gaagtttttcagattttgataaacttcatgaccagttaaaattcttgtctttctcccatgttcaacttttccatcaaacc

aagtcttcttttccctatatatgtgtgttggagccaaaccttttctatgacacatatagacatgcttcctgctaaacttc

aaccacatactatctgtatttttcccacacaaaggacagcccattttccccttaactttacatccagcaaggtttccata

cgcaggaaaatcactaatagtccatagaagcattgcctttagagtaaaagtacttttactaaaagcatcatatgttaact

ctccattcttccacaaattattcaaatcctcaataagaggttctaagtagacatcgatattattacctggttgttgtgga

ccaggaatcaataatgtcaaaattatattctccttcttcatacaaaggtgaggaggcaaattgtagtttaccaatagcac

aggccagcaactataattactattcttcatgttcaatgggttaaatccatctgtagacagccccagcctaatgttccttt

cttaagctgcaaatgaaggatacttatcattcatcttatcccatgtaactgaatcaaccggatgacgaagtttttcatcc

gtgctcttattagtgaaatgccactgtaagtccttggccatttcatctgatctaaacaatctcttcagccttggtataat

tggaaaatatcttaatactttctgtgggacacctttttttacctcaccagtgtgctgattagtcttccaccgtgaagcat

tacatttgggacatttttcaagcttcttgaacttctttctaaagaggcagcaatcattaacacatgcgtggatcttttca

tatcccatatgaaagctgtttaaaaatttcttgacatcatacaatgatgtgtgaaggacattatcatctggcaacatgct

tggcaatgtctgaagcagctcgttgaagctcttgtccgaccagccattatgtgtcttaatcctaaacaaagtcacaatag

ccgataacttgctgtggtttgaacagtttgggtatagtggggtttcagcgtccaatatctttgcaaggaactcatcttct

cgttggtcttcacactctgcaatctcacttaagtcacccaccagattctcacctaagtcacctctacaagccaactcttc

atcaaaaaattcagcagctttatataattcaaaaacttcctcattccactgatttagtttgctttgaactttatccactg

acttctcatctccatgatgataccaatcactatgcagcttgtatgcctcatccatccctcttctaacaagatgatcaact

acaacacagtcagactgccgtactacattacgacagtctttacaaggacatattatcatgtccatgtctcctaacaccgc

agcaacatcccgtacaaatttccaagcacctctttcatagccaggatcagctcttcaatggataagtaaagaaaaaaata

gaaactctaagtatccaaaccccatatgaaacctaactataattataattccttacctgtttagatgaacccatgccttg

tccaacattatatgttataatataaatttaaccctttatagataaaatagagatacagattatacaattttccaattata

atccacaataagcaacaattcagacaattccatgtaaatattcaaaccataagcaactttaaaagagtttttcatattag

aaatcagtcaatagtacaccaaatcaggcacataagcaattccatgtacataaactaatctcacgataacttaagataat

taagcaagtatactaatctcatcacataaacaacaacattaacaagctatatacgaatcagactcaaaggacagaacatt

gcaatcaactaattttgttgaatgatactaacctgttgtgtgagtacagagaaatgagaagcttcagttccccaacattg

caatcaccagcacttggaaaaaaaaaactcattcaatataagtttacacatgaatcgttacagattgaaacatgaatcgt

ttatgcaacaaatctgtaaccaaatcagtaccctaatttgtataagtttcacaatcgtttaggaaacaaatcgtttatga

aacaaatcagtcgaaatcataatctaaatttgagtcgaaactattatcacacatagaggtcgaaatcacaatcaaacagg

tcgaaatctcaatcaaacaggtcgaaatcacagagatatgaaacagagaatgaaatcacagaggatagaatacatgcctt

cgatgagagttcaattgagctgagatggagatttagggcttctcacgaacagcgaaattggggcttttcacgaacggcga

taatgctggagatttgatagagatttcacttgtacacacagagaatttggaagaagaaattaggttttcacgagagagag

aatattgagcttaggttttcacgagagagaagtatcgagagtttttttttttttagaagaaataataattcactaagtat

tggcggactaagtcactttttttttccgctccataatttttctatcatagcgtttcttaggtgctattataaagtaaccg

aaaaaaatcacatcttttatagcaggtacggtaaatgagctatcctcgtctgctatcaatgtccatatttgttgtagtga

cgtacatgccctagaaccaaacaagtcctttaccaccgttttatgataccaaaccatttgatcaacgaggttattatgga

atgttgtcgaattcgcatttttgattggccaaaaccatctgatgaagtcattgacctgttcagtggtgacattgaatcat

tgcttcagcgtatttcctctccttcctagattgaagatcagacagaaactgcaaaagagcttaccctcaaggccaagaga

ttttccactgtttgtgtttactttgtcgccaaaatccctgattccattgccaagttgctcacttcgctctctgtttccgc

tctctatttcaggagacttgaatctcgagtttctagagaacatcgtcatagcattgcgcatattctcgacttttgagaag

aataaaacactacttgttgaaaaccctcttgtgctccctgcgcttggaatgtctatgaaggggtggtcagttgtgacgag

aagaaacactacattgactttagtgtcactcttagacattgattcttgtaggagctaaaattcgtaaacactacataaag

gtaattgaaattgaaactacaaacattaaggaaaaaacatgggaggagtcgagttaatgaactctttccttaaccctttg

aatcactcgtagtgtgatgatttctgtgtgattcaaagatcccaggatacaacctctatttactgatttcgcatcattcg

aaactcagctctaacgaacatagttactacgatactctcgagacttacttttaacatagagagattgttggagcttttgc

tattgctatttgtgtgtaacgatcaaatacttcactgtgtctatcaaagacttgaagcgtaggtttatatagagattaga

ttacaaggtttaaatttgtttttcaacaaatcaaatttagtcaaccacaaatctaggagtggacgacgtgaaggagtaac

cactctctccatgaatcctattctcaagggatcgtgaaacagaagagaccaattccttcagtgacttgttttctcctttc

aaattcgacccgtcatctaccggagctccatcttgagcaaacaaatccattatggcccatttgatattcacattctttga

ttatactaaacccaattgggcttttgtaaaaatcagctcaacaatcccccacatgaatagaaatgacaaactaaacatat

gaaagactcgacaaactttaacaaactaaactagacttgactagaccagacacaatttgactagactagacagactttcg

agagtatgcgaaaatataactatatctgaaaggtaacatttttcagctttgaaccaatcatttttaatatatgtcgggtt

tactcggccaggaggtgaacatgatgtcttgaaccacccaaattttgatgtaaaccgagacaacatatatcgcacatcga

cattttctcatagaactttagttctctattgtgttcattttggccttgaacatgcctcgttattcatgagtgaatttgga

gaatatagcctctcttttattctcctagaaacggccccatttcacactcactaggtgattatccattagctttatttata

taaacatcctgcaacactccatgaaatatctcaaagctatggtatcattagaagctaatgcttaacctcaacacttacaa

gtaacactatttattttatctttaggaactggatatatgcatagtcttacggtgctcaactcttaatcaacttgatttgg

ttttccctttgaacccagattttgggatctccaatctcataagtggggtttccatcaagtttcatatttattcgtaggct

ttaaactcattcctcttgatagtctaacaacttgatctcatgtcaagcttttaataaaatgcaacctacggtcttaggtc

caatggtgactttttcccatcacttaatgcggtattttgttgaagatgtgatttgtagtaagctaaacctccccccacat

tttctaggctaagcagaattccttcaacattgcattcaacatttccgtaagaattcaattttttattttccgcaactcca

tatacttccgataaataaggaacagtaagttgatgaataatgccatgttcacaaaatgcattaataaaaagggtaatatt

aagggatgaccaaatttgacagcattattaagaaaataaccttcatgcagaaaagttgggtaactaaccttttttgggtt

gcgaaataactattttaccctttatatatgagagaaactctttctttccaacaatagctatctagatgtagggaacaaaa

aacattaggaagtaatgattacaccgctacttcttaaaaaatcaaacaatattagacaactaattaaaatcatacccaat

atctacaagccttaaaaatgtacatgtagaagaaaacttagttaaaagatagccatttagaaacagttgtgattagaagt

gtctaggcccaaatagtgtaatgaaatttaatctataaaatcataaaaatgaaatttttacccgcagtcttagcggacca

gcccgttgggcttaccggggcgggcctgaaggtattatttctgcccgcggtctgagcagaccagcccgttgcagtcttaa

aaattgaaagctcattgcgggccagcccagatggacgcacgccgacgcacgctgacccgtttgacacctctagttgcgat

tgagtacagaaatgttgcataggatatttaaagaaatgctatttatttttattacatcaaaacataggtttattgctttg

acagtatggtaaatgggcttcaacaaagataagcaaaccacatccgtgttatttatagattacataagattgtatgaatt

tacatgtgattatagaaaaacacatgaatcatgagtctgtatcctacgtgtgattttagtaaaaagcatggattattagt

aaaatgcacattatctttaaaatagttgtcttatcgaaaaaatacaaatgttttaggaaaacttacatgtcaaataaaaa

aaataacatgaacttttcaaaaaaagtacatgcaatcatttgaattgagagaaacagatcattgtaattttagtaaagaa

aatgtattatttaaaagaatatttgaaaaatacaatgttttataaaaacaatacatgtcaacaaaagtaaatggcgttgg

tgaagaatacggaagaggcagtggtggtggtgaatacggaggaggtggaagtgtttacagatctggtgatgctaaatacg

caggagacggtggtgttacggatatggtggtggtgggtgtcgtgagagaaaaaacgatggtggaaacgaataataatgcg

tgtgataacagatagggtggtggtggttattgccgtaagggaggaggaggttatggaagtttaagtggtaaacgcatcac

caagcttgaattcgtcatctacaaccctgaaaagtaaaaatttggtgattaaatcatgaatcatttatactctaaaacac

actccgatgtagaagaaattgggaatgaatatgtgataaataattggcttatctgtaatcgttagtagcttccatgagta

tcaaaacttttttcttcaagctgcgctaagagagccaatttctataaaatgtgaaaccttctctcaacgtatttttgttt

ttattttttaaagttggctatagtattttaattgacaaaaaaaaaaacaaaaaagctatagtactttatgtctctttact

ctcgagtttgggagtaggatataaaagtaattttctaccaattccgacaagtcatatacatctattgttagtttttctat

tcttttcaattttatagttagtttatgcatatactctaataaaaattcatcatatttttctcctcttccacttcgaatta

tcttgaagtttatcaagtattctttgaatttgttttaaaatttgtacttcatagaaaatacagtaatcataaagtctaaa

atgactttgatttttcacgaaactcaagtgagtaaaccacagcttaatacttaattattattatttgcaagcaaacaaaa

ctggaagtttcattcacttcacattatttgatttgcatagacataataatattgacaataaacaattgcaattattaaag

ccaattgttggtcaaagccgttgcaggtcttacgaaccaaactgcaggtttaatataatttttcaaatttttagaatatt

aaaccgaaccaatgggtcttgatgcagtattaattcactactaccatccaagcacttttacttgatcgtttatattttgc

aaagagagttgcggcttctatcacaaactctgaacttagtttcaagttttctactgaaataaactgcaggttctaatctt

tagacacttagagtaagcatcaatgagtctcaaccacaaattctgagaaatcacattttctcgaaatcaaatataattgc

aggtctctacaaatatatcagaatgatgttcaaatgaagcaatctctaaacatcttaaaacatcaatgcactacaggaaa

agcaggtattaatagcatcggaaaaacactattgtatcaaacgacatcgttattttaaacgctttatcatcgcccgtgat

aataggtctccccctttctatagcgtttttccctcttgctattaatattgttttaatgataaagtttcttttaatgcaat

tgtcgtgtttttaatagctctctatttttgttattaaaaagttcatttatagcaagagatattgtagcattttattataa

atacaattgagtatatacaatagcagttttcttttgctcatatagattattttgataacatcttatactatagcagttaa

acatcatataagctatagcgtctgattattatgattgtgaaactataattttaaatttattatttttaaaatctgtattt

aaatttttattgggaaataaataaattgatatcataaattttaataatttcgtaaattatattattttatttgggtttta

gataataatttatatattttgggcataaatttaagttttgaattttgttgttgttcttttaaagagcccatcaaatttct

tatgttgttggtatctataaaatagcagattagagttttcagacataaacaaatgaaaattttcaattttgtgaaagccc

attacactttactttgtctagaaaattacatttaattatttaaaaaaaaaatatgaacacaaagcctctttgtattcttt

gtagatcctatttcaatggaaacttgatgagtctcccaaaccttattcgtcgtcttcttcggttctactaactacatcat

caaaaacaacaacaatatgagccatatgttttcgttattttttcttcccgagttggaaattagtaattcttacgaccgtt

ctctatattcccagctccgaattcatagaataacttcaggtacatgtatagaggcgacatctccatctgcattctccgaa

cctgtaaataatatgaattagtgaaaacaaatagagagagttgaataaaatgttgaaatgttagaatcaaaggagatcaa

aggaaataatacacggcttatggctctttgttttgaatctgaaaaaaaatcgagagaaaagaagtcgagaagcttcgtca

gtcgggtttcaatctgagagaacgaagattagggattttagggattaaattctcaggagctcttcaatgggcaaaaatat

tctcagatactcctcttataaaaattaaactaatttctatttttttttaatcgacatttgtgaaaaagactttagaatcc

cttagaaattgagtttattaccgacccaaccaagtataacaaataataaaatattccaaatttaatgtaatcgggtcaaa

cgttgatagatttttttttccttcttttattgttattttttttttggttcaacttggtataactacataaagaaataaat

tggtagaattcgtatcgatctatttcttctccaatttaattacgttttatatgtttttcttctatactttttcgtcgatt

ccaatttagcttggtctaatatgttagtttattttcaattttgagtagaattagattgaaattagataaatttgttgtga

atctaattaattttgtttatagtttatgaaagaaagtaatgactcgaacaaataacccaattaataactgagaaaatctt

tggaaacgaagaagctccttcagaaatagatttttatgcaacaaatccaatctctcacagtgattcgacaatataatcta

taatcctcagacaactagtagaacttggtttaactatgtttctttcttaactaagaaattttctgataataaaaagtaaa

actaagtataattttgtcagatgctctagatacatttacatactttgcttcatgaactgttcttctagatgatatatatc

aagtttgataaagctttaaatgtgtgtatatcagccaacatatatttgcagtcgcttggctccactaatagtaagctaat

gtgttgggagggtttgagttgattctctttgatatgtgtaactcaaattcatatgtaaaagaaagaggagaagagtagca

gcgtcggctaaagtggattcttaagcaacaaattcaatctctaacgacgattcaacgatatgaactttaaactttacaca

actagcggcgattccaagtaaatgaggcgttaaaaagctgaaggtggtaaagaagatggcgaaggcgaagacgaatgcga

agccggtgtatcggagccctattctagcaaaaaatgcaaagacaaagacgaagaatgtttaaacacttttaattggttgt

ttgtttttgaaaactttgtttttcgattttgattatgtgacgtgaatcgggttgttgttttattttggtataactgtgtt

gttagttgaaaactaggtacaactgttaaacatgtttattgttatttttatatcttcttatgttttctaaaattaggtac

aactgttgcatgtttattgttttcttttggtgtatctccttatgtttgtaacactaggtacaactatatgtctttataat

tgataagaaaaaatatgaacccaaaaaattaggttttctctactacaaacacaacactaattagtcttcattattaaatt

tatcacatcaaattcaacacatagtcttcataattagatttgaatctgatctaaaaattttctcaacgattccatcgcca

caccaacactgctttggaattccaagaacctttaatcttggtcgggcaacggaggttccacacgatttcaaaggtcccga

gaggtaactcataattcctagagttttggatcttaaattcatgaaaatatccaacagataaaccaggttgtactatatga

atcttcaatcttaaaatcgtgaatagtcaattgaatatcacaaatctataattgaaacaaaaattgaatgaaaaatgtac

ccagaaatcgaaaacttactcaaaaccatctcctaatacttccactattctctcttatttgtaataatgaagaaatttaa

atttttagttttagggggaagaggaaaacttttgaaaaagaaaaaatgtggtgccgcaaaaaaccgtgagacccataaat

gcaggtacactggcacaaaaagatgcatgagtacatcaggtgccgcaaaaaaaccgtagggcccattaaaaaattgcaca

cagattttcctagtttacaagttgttctcgtatatacacagattgacgtttaggagtttttgtatttaattagatatact

tagattaaaacttaacaaatttagttataccaaagatttttgttgttttagatacatttcatatagtttcatacaaaaat

atgtttaaaaacacttattttgtgtgttttgcaatgttatgatgataataattataaggtcatcttaaaagtataaggtc

tttcttctataacaaatgaattgatgaagactcgttttaaaattttgagtaaataattattttatcaatcagtagtttta

ataaccaacataagtagtataatttgtataagaaacaacattaaactaaattttattgcaatttatgcagatttgttaaa

ttagtcatagttgtaccattagaaatcaagtttaatcaagttttacaaaatcagaaagtacataaaaacacgaatacatt

aattttgcttggatcttggccacttccctggctgtcactttcgcgatttggaagaaatcagctctctaagtttctttctt

tcacacgcttttcctttggcgacgttctccaactgatgggtgtctagtttcttgaattcttccatttttaacataagtca

ccggaggatatgcaaacaattcttggatttcacttagaataactcactcactcagacttatgcggcacacggaaaatggt

tctaacctaagccataacccacatctcaagaaagtaatatttttgtgaccaaatcatataaactttgtacctcccgatgg

aatgtaactcaatttcagcttgaacatagattcatctatcttaatcttcatgcatatcttatccactaactgaaaataag

ttatctccttcattgatgatgtcctaaacaatataccatacaaacaaactacttttggatttcactgatatgcttctccg

atatttgaatagtattcatcataatcaaaatatatatttattggaaggttgctcattctcctgggaaaaaaacattagct

gttatctcagaaattccagttgtaccagatataccacttgaaagagttataccacgtttctcacttatacccggattttt

atgttctatcaaattgattctcggacccaaaacactaatctgcctaagattaactatgaatcaaccattaccgtacattc

aaatgtcttaatcgacccagaatacatcgttaccaacataatctccattaattttaacaccaatttcaaacctatcaaat

ctaattgaaatctctaaaatacaaacataacaacacaagacttgatgaattaagatgtgaaaagaagaaaaagagaaggg

taaagctcactttgaataacagaagaagaataacaacgagattgatcgattggattcgacgatggaggattcgacgatga

ctgcaaaagggcgtcgtcgattttgtcctgatgaggaaaaagggaaggtatttccaggtatttctgattggttaattttg

tttttattttatgaattaaataaatagtattgcattggtttggaaaataaaccgtaatcggtttgttttgttcggttcgt

tgggtaagggtaaacaaggatttttaaatttcattaagagtatgagagtaaatcagattatagaagagtatttagactca

tttttgaagagtagagagtattatagatcattgtccggatatatatttttttttttacagtcgtgtatgaaaggcgaaag

gtcgtgtgcaaatgtggtttttatttgttgagtgaagtaattaaaaaaataagataattagttatgccatagatatcgag

aatcagatgactatctaatccaatggtctcctaatacgccacctcagctatccgagtgatctcttattattgttgatttt

ttttgttttattttaatcatattttctagactaatgaaaaacaattaattgtagtgtaaatttcgaaagtaatatgttta

gaaatctcttatttatgtactttagcattattgaataactttttcaagtttaattagctagtatattacaagtacactaa

gtttcaaaattgaattctctatacccttaaccctatatcctaaagggtttagagctagtttagggtttagggtatagaaa

tttcaattataaaaccatttagtaatatatttattagttttaaatttatttgtacttaatattattaaataactttatca

agtttgattagataatttattaataaactaagtttcaacattgaactctctataccctaaactctaaaccctaaaccctt

cagagtttagagtttagggtatagaaagtacaatagtcaaacatattaagaattatcaatttaaactaattatatatata

tatatatatatatatatatatatatatgatctttagatatgattatgtaattatttgattttatttgcttttagtaatta

atcaaattaattatttgcaattatttgattttagtaatatataaattaaattaaatgtatataagttaagtttgataaag

tatacggtttaatatgtacttgtaattaaatgtactgagtttttttccagaaatattagttcagatttctatgtcatcta

atagactttgtttttgttttttgtatgtgcacattttcactagaggaaaaaatggttaaaaaacataattatccaaaaac

acagaacttttatttaatttaaggtaagtacttagatgaacaagactttgtcatttggccaagccattgtcattcctatt

gcatcagaaatgtacacgatttctgaatttggtctccacacacatgcatcaccaatcttagaaacttctacccacacttt

gctagcattcagacctaatgggacgtgatggataacagcagctgagtcagtggagcatactctgccttctgcaagtttgc

ggccggagttgttacaggccaacaaaatgcactttttctttgctcctttacttgttcttgtggtgttacttgcactctag

cacccaaaatcaacacaaattaattactctcatgacaacaacatcgataccatacacatcaattttttccacagtaccaa

aaaagctagtgctcaaaatttaccggagtagatgactttctgcctcattcaggtcgtcttttaactgtgctggttgtgtt

acttgcactgtagcaccagggaacacaaatattaatatatgcatcagagtgacatagtttttatctaaaggggacttacc

atagtagatgatcttctgcctacttcctatacaggttcgtcaacaaagtttgtctattctatgatcaggccatgctatct

ttgttttaaatgcatcagccatcacaaactttttgggatttggtctccacaaacaagcatcaggtttaaggactatgtca

actttcaggataattgcattcacacccaaaggtatgttattgatcaactcatctttgttgcttgaacagataacaccctc

agctataacttcatccattttccaatccaaaactctacatctaccaagttgaccatcatctacaccagtatcctatagta

gagtagtatttattattaatcgtttgatggaacaagtgaacatatagttatagttaaaatacattaccttgcctccattt

gccgtttccgagtcattctgactctccaatccagcgtccaatgtaactttatcaactagccatgcaattttgactcctaa

agcttgttgaagtaagaacacatctggcgttggcctccaaacagatgcttctagaactcatacagacgtgacgattacag

ctgcgccatattcacccaacggaatacgacctatccgatagttaggttcagcagatcaaaactctccttcaccaacaaca

atatctttacttgaataccaatccagtatctaacacctaactcctttgctaacatcactgacctccgagtgagaagtcca

agtggagtttttctaattaaaaaaaaatagaattgatgcagtcatatttaactatagacagttaattcaacatagaaaag

tgagagaagaacttacattctttccagccatgtcacgaacaatattcttcaagtcttcaacctcgttttgcaattcttca

agtctcgagtctctagcctgattgaaagctagcttggtagcagttatccctctgcccaaccctttgtcacgtcctgattt

gtcttttcccaaaacttgactcacaacatcttctcttatgttatcacctaaagtggagtccatttgactatcatgtgatt

gtatatgttcctcatacaaaacattgagtataatttggttatatagaatgcctttttgattagtgatagggatattaata

agataaagactgcttacaagtgtttcagcaaactcaggtctcacgggtctaccataagagtgagtgtgtcctgctatcca

aactttactccttgtgatctttttagggtcatcactgttttttctacaagtaatccaaattacttacttatcaggatttt

ggtaccttctattgattgtatctaattcagtacagaaaatatctcaccatttcatgagctaggtgaaacattcccttccg

attggttgtgtggggattttgagcctttctcaatttcctaaacttgttgctttttttcctgcaatagcaatgacgttggt

tgttgagtagactctaatacatactaatagtaggtacacacaatgaaaaaattgaaacaatttcaccttcaaagttgtgc

tagtccaactcttaatccaagaattccaaactgtaatagatggtatattgcttggtttcagtttcattctctctgtgcta

ctcttaccatctcgcacctgagttaggagcttaggctttgcaaacctccatatacaacccatctgtttgaaaatagcatc

ttttctaccactcttccttcaagttaaacctcccctataaaaaacatggtatatgttaatcttgatcaaacacgtataat

atatgaatttgtaaattgaaattcttaaacctgaatttccagcttcagactgtacttgctcatcaggttagtgacttgtg

tgtacatcttctttttcagcaacatcttcctctacaacttctttctcaacagctacatcttcttgatcaccaacaaaaac

ctctataccatctttctggacattttcctcaccaacccgcaccttaacaactacttcctaagcatctttttcaccttcca

gaatctcaagattatcttcttgaacatcaatcatcagttctgctatttttgtcttctttgttttcatagtttttggccgc

tttgtgatctgtctagtctccaagcctccctggcattcactgggatggactaatcctcctcttgtcctcatccatcaaga

tcctgaaaaccatgaattaacagttaaaaatatttttcaaattcaaaataagaagcaatgtcctactctgccattgtgaa

cccttcacaatcatgcctaacaaagaaactctcatcatcagaagattgattcccaaatcatcgtctaattggtctgataa

aggtacaccaacaaactcttcctttgtctccaactcgtgatagcctctcggaggtgccatcataacaacataccacggtg

atgtgtcattctctctagattaaaatacttgtttagcttgtgatgggagaatatagggatcttgcatgtatgacgcttaa

ttcatatggaggttcacaagcgtgaatccatcctcctgcttgacaccatatcatctatcggctcaattgcatctgaataa

tggtactgtgaacatgtgatactctagaaatattatttctttgaacactccataataggtaactatgtcagcagtttgtc

tagtatcccgtgttgtagatctacacatgccaaaagcttcataagtcactccactgttctgagttagaagggatctaatg

ttcatataacaatatcagatacataaattaatttaaaaacatgagaaagagtgttaaagtgacgataaaaaccaaataga

tatgtacaaagcttttttttaattcattgggcaactgttttgtatgaaatttccatagcaatgtttcatttctagcgcat

tgaccatcctgagactataatacttccaaatgcatcctgttttccatagataaagctgttatcttactagtataattact

aaagaagctataaacagttcaacttactccacatagggatccacaggtgccatgttcatcaagacatatcaatgtgctat

gtgatataggtcaaattaatcataagaccactctctcaaataaagaggtcaattgcagcgcttagggatcgaataacaaa

gttctcggatcacgcaatagactaacggcaaatcgaattatgctaagtaaaatagtaaataaaataaagagaaacaaaag

tatgcaatagcaatcgattggatggttgtgaaacaagataagaaaagcgtcaggcttaggctattctcaggaaatagatg

atagtagatctagaaatagctaggttattatcgcaccgttctcgaactcaaactatgattttagagtaacctgtggtgtt

ccgcgaaactctctatacttagagctaagataaccgaagaagccgagaaatcttatactaaaccatgcatttttatcaag

aatgattagttcacctagtatctaaactagagcccttctatgagcctatctgttctttcttaaatgcctaggcccatcta

tgatggatcaaatagcaagtacctatggtggacatacctattatctaatatcaagttctagttagctactctataactag

cattaagaacaatcaagataaagaactctacagataacctagcaagggggcaatctactaaatcatctaaatccctaatg

agaaaccctaaacctaacaagtgaattactcagacatgatcaaagaaacacaaatcatagtctgaataaggaatcaataa

tagcaagaaaaagagtaaagaagatcttctccaaagggagtctccacagggttttgctcccgaagtacaaaaaaatagag

aatatcctttccaagcttagatctaaacaatgaccctaaaaacctaattatatgtctaaaacacgtgatgggccttcatt

aaacataggagaaagttctgggccgaatttggaaatcttcaaaacatcaataagttgtgtctcgaaattgccgtcaagta

atggtttcgctcgacacctaagttcattttcgtctccggattgtttctgcagcttaaattctctgttttcctccagaatg

ttccattatctccaaatgaatccaaacatgtaaagacctgaaaaggactagaaaagactctagaaataacaattagactc

taaaacctatatctaaaacatacttaaattaggaaaaatagggatatatcactatgtctctctctttctctgaaaggaca

acttcttgacccctatgcaatggtctgccttcaacaacacttccatcagcctcaacatcgtcattacggcttacagcctc

ttgcactggtataaaattttggaggaactctaagcagaaggcaatgcactctccgactaagtatccttcagccatacacg

cttctggtctagcatagtttgttacatatcccttaagtgttttcatgtacctgcaactcatacaatttgtagtcattatg

tctgaaagtaacttctgtcaaagaacataatgtaaagatagaaataagaccatacctctcgaatagatacatccaacaaa

agtgctttggacctcctaaccgtgtttctcttgctagatgaattgggaggtcgaacatgatatcaaacagagaaggagga

aagtacctctccagttgacacattgtctctacaatttcagactcttaggaaattagattctcagggtcaatggtatgttg

gcacaatctgttaaagtagttgcagactcgagtcactgcagccctacgtctgcttggcagcaagcctctcaaagcaacat

gtaacaaattttggatgagtacatgatgatcatgggactttagacccccaatcacatgagggttgactgagacacaatta

gcaatattcccacaatagccatcacgacctctaaacttagacaacctctgacaaaatacctttttctcgtccttagacat

ccagtaggctgctggaggcaaatatatttctttccccgagtctttgggtgcagatgccttcgaattccaatatcatccag

gtattttcttccttttaagccatctttagacttagcactttgcatcaatggagacaataaagcatcagacaagttcttct

ccacatgcatcatatcaatattgtgatgcacgggtaaatcataagacaaagataatcaaagaatcagtttgactatcatt

ttaataactgtaaagtgctacaattaagacttagaaaaatataaaatattaccttctagtatactaactcaaacaatatt

gaccgcttcttccaccgccacagatcaatgctttcatcacactcttctaacaagtctgatctttttcttttagcttcctt

atttaaaggtcttccaaaatcattcctgaagtccttcaatatgtcatatatctcactatcagactgaatccttcttgcgg

tgccttcctcgacagtgttatcaaaccatgatttctgcgtctataaggatgtcctggtctcagtctctttctattaccca

tatacaaatacttccggcaattcttcaaccatctgcaagatgtatcctttccacatgcattacacgcttgttttcctttc

actttatagccagacaaagtgcctaatgtttgatagtcacggattgtccacaacaacatagctctaagggttaacatctc

tttcaaaaaagcatcgaagacctcaataccctcagcccacaaatctttcaaatcatcaatcaatggtgcaaggtacacat

caatattgttgctcagagttgttggaccaggaatcaacaaagtcaacataatattctcaaccttcatacaaagagatgga

gctatattgtagttcactaacaagattggccaggtactatacttggtgttctgaatagagaaagggttcatcccatctgt

agacagaccaagccgaagatttcttggttccatagcaaaatctggccatttatcatttacttgagcccacattatatagt

caacagggtgtcgcattgtaccatctccacttgcattgctataatgcccctgcaattcctctacaatcctctttgatcaa

aacatcctcctaaatttgtccttgattggaaaacatctcaataccttcgctagaatccccttcttttcttcaccactatt

cttatctgtttaccatcttgaagcactacacctagggcaactttcaatcttatcaaaatattttcgatagaggatacaat

cattcttgcatgcatgaatgacatcatattctaacccaaaaatctttaggaatttcttcattgcatcagtagactagcaa

gggcattctctttaggaagcatgtcagggattaacgtcaatagcagatcaaagtagctttctgagattccactcttcacc

tttatgtggtatatgcccattattgcagaaaccttggtttaattagagcacgtcgagtacaatagagtttcagcatctct

cagtttctgcctaaactcagagtcttctatagtttcaccatccaaattttctcctccaaagttgtttgatggctgtgtat

catcaccattacaataagccgtccttaacaaattataagcctcaaattctaatgttggaccattatcagtaatatcactt

tctttcaccatgcatactccagcaagaatctctcatgtatttcttaatcatacccttgatcacaagatggtccaatactg

tgtctacttcttgatggcaaacattacggtaatcaatgtagggacaaaaaagttcagatggatttcccaatctccttgat

gcaacaataacaaaatttgttgctccttccacatactcttggcttgccctatatgtttcatcagacaataaagagttcat

atcactttattttgacttagtttatgttattaaaatcaatgaatcaaacaagactcagtatcaacaagactcataccttg

gaagccacacccatcccttgtccatatcacgacaataggacaacaactccaccaccaattatagattttgtaatgataaa

ttcaaacaataatacactcagctaaatttcattcaaatcaacacatacttggaataaacaaattagtatgaaaataacaa

cttacagagtaaaacagtcgccaacaacaaaaccttgcagctttgaaaaccgtctgcaaaacaaaatcaatatcatcact

accttaaaattaaaacccaacactttaatgtagttggttcgattaaatccaaaatctcacatacctgtcggttactccct

aaactgaactgcaaaaccatatcaaatcatattgagaaattagaaatcgattatcgaaatcaaaacaattaaacctaatt

cccaaatctgaaatcaaaaccctaatattaccttataacccaaattcgaccagacaacttcgatttagaccgatgaagac

gatggcgattaaaaagatttggatctactaaacattcacagagatcgattgatagagagatgtcgtgagaaccaaagtcg

aatagtcgggagagaaaggggattttttctgggtaatttttttattttttatcttttaatctgggtagtcaacaatttaa

ttttgcggtcaaacaaaatttattttcgtggttcaccatttgatagcattttcattatgcatatgctatcgaataaccaa

taattaaagcgggagacaaatacttgttgccgctcaacatagaatagcgtttgttttgaacaaacgctatagaaaaactg

gtcaaaccaaaactatagcatatacttacaagacgctatcttcaactgctcttaatacccgcttttcctgtagtgatgtc

aactatgaaagactcaaatccaataaaagaaaaagaactaaatttttattaattggaacaaaggttacaacttagtctta

ttcttcttcaaggtcaaagccttggttacatctagttgcgaataacagattcaacaaagccttttccatgatacattcca

tctagttaaaaagaccatcacaaacaaagccatttccaagatacattccctttttcgtgatgactagcttatctgcatca

atcttgacagcgaagccatttccaagttcttgcgcatgttaggcacatgaaccacgttggttagaatcagctcacgtaca

gaggtgagctttaagatgactttcccgtcaccttcaatctttgatgtggcggaggcatatgtgcctagtagcattggtat

cgaaccaccaagtcatgggatggctttcagcgacgttggcttcagtgccgacagcacacatgtcttcttgagttaggtta

gcttcattcttatttgaatccttcttactgcgacattcaaaagacttgtggccctccttgccaaacttgtaacacgttcc

ggtgaacttgcttgtgttctctccggactttttttttaacttttgagtattctgcggaccaaccttccctttccccttgt

tctgccacttttcttttggcgtgttgtgctctgccacatttgcattggggtcaacaatctgcacaccaactcgcatttat

tttctattcgttgactcgatgtggagtctaacaatgaggtctttgagactcatcctctttctcttcagcttgaggtaatt

tttgaagtcgttcaagcctggaggaagtttctctatcatacaagctacttggtatgtctcgcatatcctcattccttcag

cgtgtatcttgatatggttcaaattaccctaagaactactctctcaaataagagatccattgcggtatttaaggatcgaa

ttccacaaagttcttttcttcaaacaataagttcaatgtcaagattaagctagaagggtatgatcgaaataataagaaaa

caaaggaagaaaacagtagattgtttcgttgtaaacgattaaataaaaagctaggaacagggtattctcatgaaactatt

ggttagtagatctaatgaaagctaggttgttatcgaaccattcttaaactcaaactctaattatggaataactggtggtg

ttccgcaaaactccctataattatagctaagaataaccggagaattcgagagattattaacctaaatatgcattcttaac

gagttcgattgttcaccttagtagataggccaatttttattacacacctataaaccaggctcatcaaataatagatccaa

ctacagatacctatggtggacatatctattgtctggattcaagatctagttaattactctagatctagcattaagcataa

tcaaagatgaagaattctacagataacctagcaaaggggaaaaattactaaaccatatgaatccctagtgagaaacccta

ttcctaacaagcagattactcagacatattgattgaagcgaacaacataattgtgtatgaaagcataaacacaacaataa

aaatgagggaaagaaagtatctcttcactgtattgaaaactgaatgaatctctgaaaacaatgggtgaatagcttatgtc

tctcagtaacagggttttgcaaaagcttgataatagaaactagggtttagaacaatgtatataaaccctataaaacgtca

agggactaataatgcaaataggaaagtcttttgggcaaatttcctcttctataaacttgtaagcgtcatagacttttgca

gggccgaaactggtgtcgatcgacaccaagagtgtgtcgatcgacactcctcttgattcctgggtccaaaatcgtcctta

gcttccttttccttacctcttgctcgaaatgtctcctcatctccattgttgtcccgttgcatagaatacctgaaaagaca

ctacaaagactcgagaaataacataaagactcaaaatccattaccaaacacatagataaaatcggtgaaaataggatata

tcaactccccaagacttagcttttgcttgccctcaagcaaaacacaaaagtcgaacccgtggaagagattttgaaaacaa

aggaactcccaacattctctagactattgccatgatcatccaaactaaatccacatgcctagcaagtctaatcaaatcct

aaccaacatgtacttctctacaagcttacacaatattctagattccatacctaagttcaacaactatatccactaggctt

tcatcgctgggtctcatcaaacaatttaacattcaagatccttctagactcactcatgtactataccatgataaatcgac

cattctttatatagttttttctttggtgttctttctctcccccagacttattcattttagagagtaaacaaagggaaact

cgcatactggatcgacaccctcggcatttttccaagctttgtaattgatcattccaaataatgtataataatggggtcat

aggaaacattcctacccctatacactatccgaaaatcctgtcaatctctcattatcttttttttttataagggaagagaa

acacattctttttatcacattacaagaaaacacggtttttacgactgcaatagtagtcgcaaagtagtcgttaaaggagt

agttacgactatgttacgactaattcccgatgtcgggaatccatagtcgctaacccaaatagtcgtaacattagtcgcta

aataacgactacgagacgactaatactcgatgtcgtaagatctgtcgtaacatagtcgtataatatgacgactgagttac

gactaagttaatattgaatcacatgtatatcgactcgtgtatggactactttgcgacaatttagcgactaaatagtgact

aattaacgacagttaagcgactaccgtttcagccaaaaaaaaaaaaaaaagggtgtttgattttgtttgtatccaaattc

attccaaaacacataaaaacaagacaaaatcatgattcaaaacaaactaaaaacctgaaaagattcaaaaatcaccaaat

tcaatatccaactatgattttagagtaaccgatggcgttccgcgaaactccctatgcttatagctaagaataaccggaga

agccgagagatctttaacttaaacatgcattattatcaaatttgattagttcacctagtatctaaaccagagcccttata

tgagcctacctgttctttcttaaatgcctaggctcatctatgatagatcaaatagcaaatacctatggtgggcataccta

ttatctaatatcaagttctagttagctactctagaactaccaataagaacaattaagatgaagaatcatatagataacct

agcaaggggcaatctactaaatcatctaaatctctaatgagaaaccctaaacctaacaagtggattactaagacatgatc

aaagaaacacaaatcatattctgaataagaaataaatgatgaaaataacaagagaaaagagtaagaaagatccaaaaggg

agttttcacaggtttttgctctccaaagtacaaaagagatccagggaatagcctcccaaagcttacgggtctaaaacaat

gacctaaaaactatatatatgtcttaaaaacatgatgggccttaattaaacataggagaaagttctgggccgaatttgga

aatcttcaaaacatcaataagttgcgtctcgaaattgcagtcaggtattagtgttgctcgacactaggggtggtgtcgtt

cgacacccacgtgcattttcgtctccagattgcttctgcagcttaagttgtctattgatctccagaatactccataatct

ccaaatgcatcctaaacatataaagacctaaaaagaactagaaaagactctagaaataacaattagactctaaaacctat

acctaaatcgtagctaaaatcggtaaaaatagggttatatcagaatttcatgagagttataaagtagggaacttaaaact

gagatggtgaagattagataggatcggttgtggttgcttcagatgaatgagtagccataaaggtgagaaagtctggatca

gtttggcgtaaatacttttccaccaaggccaagtgatccagcttgtctttctgttcagccacagctcggacttgctctgc

ctcacgtctagcatgatgagcagcctgctcttcgatcttctgttgagcttctttcagttgctcttgcaatgacacaaagg

aggtagagtctgctgcatgtttgccctttccagtgactagatccggaagattatctttgaggcttccaactacataatag

ttccctcttgaatttgtctctgtggcctatgaaaaaaatagccataccattaataaagagtttgagaaaatgcagacaat

atgcaagtgaaaatgtaaactttacctccagaaagatgactgtatgatcatctattctgaactcccgaggtcttgattcc

ccgtctgaaacatttgaagtctctgtctccatttcagacaacttcttgcagcttcttctcataatttttaacaatctgct

ctgcctttttgtcgacataagtaccatctggtttggtatgagtcttgatgaaaatctcacgaacactcaccgtccttcct

aactctttttcctgtgatgttaaaaatgaacaaggcttaatacaacatctgaaattaagacagagacacattaaaaaaaa

aacagagatagaagttaataaccagttcatgtttgatattttgatatgactttggccctcacaaatggatgtgaggaccg

agcccattacggtcagacaagcgtgctttggaatatatatgacttctcgctattgcttcttcagtatcccaatactcaca

catcgctttccagatataagaatcgatgatccatttagggcactctcgactagtccttatcgtgctaaccatgtctttca

tccttcgtttacagatttcttcaaaataccattgaactgtacctgttatcaaaggatcccaatggtgtgttttatacaaa

taaaaaagagaagatgttaatgaaaacaaagcagcttaacttaatactctgaaggcaagaggaaggaaaactataccaca

aactcgagaaaatatttttctcgtctatcctgaggaacacatgaccagctatagaacagaccatcaaatttgtttgtcca

tactttagtagtcttcctaaccaggcttgaatttgtgtcccgagtaaacctgcaagcaatacacaagacacttaatacaa

tcaaaaccaatacacatgagactcaaaaccacaataactaatcatcacaaacacataagactcaaaaccacagacaacta

atcataacaaacacttaagacacttaagacacatctaattcaaacataaaaacacacgcttaagacacagacacattcac

tcatcaaaacaagacacttaataagttgtgtttggcatcagactctaatcaatacatcaaaaccagccatgtcatacaaa

cgatatgtcatatactcattactcagtccacataatcaaagacaagaatcatgatgaattatgcaacaagattgatctag

acatcaaaacacaatacacatataagtcacacaacataaaaactaattaatacacaagacacatctaattcacacatcaa

aacaaacacttaagacacatctaatacaatcaatacaaaacaatccaatcaatacacttaagacacatctaattcagact

ctaatcaaaacactcacacaacaaccaataccttcaggttccaatcaatacaccaatcaacatttaattcagactctaat

cacaatccaatcaatacacaagacacatctatactagacactcgcacgactacactcaatacactcacacaacaaaccaa

tacacatgagactcaaatccggacaactaaccaagacacatctaagaacttaccaagttgtttttggcatcagtgtggga

gaaagcacggtggtccacgcctcacgaccaggcacttgaagcacatcatttagagccctcaaactgtcctcccggagttc

cggtaacacatcagccacaggttcgtggaagttgttgccttgggaagatggataacttgcatgagagttctgaagtggag

aacttgaaggatcctgttgtggaatcggtcgattagatgcctcgttctggacttcttctgacaaaagaggtacagggact

tcacgattagaggagttctggaaaagggcttgcggaggtggatagtcagagactcggggagtgaatgacctagcttgttg

gggttgaacccggactcgctcttgggatgactgaggtggaggattgttgtctgccggcgtaaagttgtattgaggaggta

gagggtttattcttttccccattgggttccatttttctcttccttcctcgcttagctagaggcatcaaatcgaacttgtt

tgcaaaccatagaggagacgattcaatgaaagtgagagagatccgagagattcgaagaagaagatagcagaaggagaaca

attgatgaagatttagaggaaggattgaagaaacgaaagagagagatgaagattctttagggtgaatcgaagaaacgaga

gagagagagaggcggaagacaaatgaattagggttcttcattctcgttcttcatcactcgctaatcaacacaaaaattgg

gctttgtttgggcctcgttaattttggcccgcgatagttttctcgggttttaattcagcccaatgttttgattgtagtcg

ctaattagtcgttaagcagtcgcagcgtatattaatttttttaataatcataggttttcataactttggtctatatccaa

aacaaagttatttatgtgattaacccttcataccaaaaaaaatgttcaatcactatatatgcattaatatatatcctttt

ttaattaattaatgagagtgatatatatactttgtacaattttttaattaaagatagtgcatttttaaacaaaatcataa

gttttaattatttatgtgattaacccttcataccaaaataatgttcaatcactatatattcattgatctatatccctttt

aattaattaattagagtgatatatatactttggacaattgtttaaagaatgcatttttaaaataaagctttgaactgaaa

tatgttataatttttacttatatcttgacaattacaaaactaatagtctaattttagcatatctaattttacttagtata

cacaaaattataataatataaaaattctttgattattgttacaattttaacgatttattagcgactcttaattctctgac

ttacttatagtcgtaacgtagtcgttacctgacgactatttggacgactatttataacagtcgtcatttagcgactactg

aacgtcccttttattttagtcgtagatctgtcgttgaatagtcatttaatagtcgctaaatgttacgactacttatacag

tctccagatgtagtcgtaaaaaccgtgttttcttgtagtaatattaggtctgaacaaagagtcaagatctatgaatacta

accatttgaagaggtaaaaggaagagcaatgacattagctccagccttagtggttttgttggaaactaggatatcctcaa

gtctgttggtttgccatctagattttccaatagttggataggctcgcaccgtagcactcattaatcaggcaataatcaag

aggaaatatagatcacgattcttaactaaactaaagaaaacattttttttttagggaaagaaaatgactcaataaattca

aaaacgaaaaataataagaattaaaaataaaaacataattagtaagaacctcctccagacttaaacgacattgtcccctg

ttgcattcaaatccagagaaaacgggaaaaataaatcaaaaataaaagaaagtaaacgaaagggaaagaaaaatcaaacc

aatgggttgtctcccactaagcgcttgtttttagtcaatagcttgacttggtggagttcaggatgtaggtggagtggtgg

aggattgctgctgcctgctcgccttccaagacacccagtggtgtgtcgatagacactcatctattcacctgcaagaacaa

caggagaaacaagctttcgaagaactctaggcttgttatctagtgaagcagacttcaaagaagctaaagaattaatcgaa

ggtaaagcaagagaagagatagaagagaacttttcacataggtcctcaaatggtgtgggctgaagagattgtgtagaacc

ctctgtctgtgaatcccgagggtgtcgattgacaggagtggtggtgtcgatcgacaccgttgctctgcaactctcattgg

cgagttgagctgatgggagcgtcgtgtgaggctctttcaggggatttctgagtagtcagaggaggtgtgtctatgctaga

ggcaatactactaatatgcttattaagctctgaaccatccatgccaaactccatctccaaatcacaaacctttagagaga

ttctacctctctacacatcaaacctagcaccagctgtagctaggaatgctctttcaagaataagaggatccttggattcc

ttctcatagtccaggaccacgaaatcagcagggattatggagttaccaactttaacaggaacatcttcaagaattccttc

agggattcgtttggaacgatccgcgaagaggagagtgattcgagtcggcctgtagtgagtcatgcctactctctcagcaa

cagacttaggcatgaggttgatacttgatcccagatcactcaaagagcggctgaacctacttgtggagataaagcaatca

agaacaaagctacctggatctggaaccttctcagtggtctggtctggagagatagtgtagatgtactcaacaatgtccac

tggttgtgccttctctttcttgaccttaggcagcatgatggcactgatgtcccgtgtcagcagcttagcttcatcggagc

taataccattgtcaacaagtcgcttggcatacctgttgagtggagaagaacatgtctcagatgcatctttagggagagaa

gtgaggatcttctccatatagtatctccaatcaactcctcttaaaaaggtaccaagaagttgtacctcactgaaaccatg

atgttggtaatcccgttgatactccttgaaacgaacccaagcagccttgaaagcttcagctggaccttgtgtgaaggtag

acagcttgtttcttaactcctcagacttagcatcatcgtagaaattgttcaagaaggctatcttgatgcttctccaagtc

ttgagggaacctgctttaagttgtttcagccaagaagcgccttccccgacaagtgaataaggtaagagcttgcacagtag

gtagtcctctgagactccattagctttgatgctcagaacaaggtcctcaaaacgctcgatatggtccattggctgctaat

gagataagccatggaaaggatgttggccaactaaagcaaagtagccaggcttcagctcatagtcgctcctcttgaagggt

gggggtacgatcgctgatcgattagcatagaatagatctggcctattgaaatcctgagagtacgacgtggttccgcagct

agaggaatagctgcaggaatctgggattgatgtcgatcgacacctctgttgtgtcgttcgacaccaatctcgtcttcatt

ctactcctgaaggtgtcaattgacacctctgttctgtcgatcgacagcaagccctgctccgtctctggtagctaattgcg

cattgaggcgcgcctgatcttcattggcgtcctcaggtaggatgagtccatgatcatccaatctctgaccagcctcattg

taggcttgcccattctcatctctaaaactccttctgcatcagctgtgatgactctgtcagccattgctaatctctgagtt

tttcggttggttcgttcaagttttcccaagtctaagaccgacaagttgataagctccttctgtattgaaaactgaatgaa

tctctgaaaacgttggataaatagcttatgtctctcagtaacagggttttccaaagagcttgataatagaaactagggtc

tagaacaatgtatataaacaatataaaacgtcagggactaataatgaaaataggaaagtcttctggggcaaatttcctct

tctgtaaaattgaaagcgtcatggacttttgctgggctgaaactggtgtcgatcgaaaccaagagtgtgtcgatcgacac

tcctcttgattcacggatccaaaatcgtccttagcttccttttccttatctattgctccaaaatgtctcctcatatccat

tgatgtcccattgcatagatacctgaaaagacacttaaaagactcgagaaataacataaagactcaaaatccattaccta

aaacatagataaaatcagcgaaaatagggatatatcatatatcatggaagagacaggtcagctcctcaacttgcaccatc

ataggccgtgagtccaccattttgtagtctagaaactttgccgcggcatatttctgtgatccagcatctttggtttggta

cttcttatcaagagaatcccacagctcgttagaggtctcgacattagagtacacattgtacagcggatcggagaggcggc

tttggatatatcccttacacatgaaatcaccgtgttcccacgcatgaacactcgcaagcgcacgagggtccatgttgtcc

gctgggaggattggtttctcttcctgcgtgtactggctcagcttcagtgttgtcaagtagaaattctgccacatcttaaa

tgagctcccatcgaactttttgggaaggaggcctgctgaatttgccgcagtgtactggctcagcttcagtgttttcaact

atgttttaggtataggttttagagtctatttgttatttctagagtcttttctagtttttttcaggtttttacatgtttga

gattcatttgaagattatggaacattctggagcaaaacagaaagcttaagctgcagaagcaatccggagacgaaaatgca

tgtaggtgtcgaacgacaccacccctggtgttgagcgacacgaatacctaatagtaattttgggatgcaacttattgaag

ttttgaaaatttacagattcggcccagaagtttctcctatttttaaataaagcccaacacgtttttagaacctatatata

tagtttttaggtcattgtttggaagtaagctttgggaggctattccctttttctattttgttcttgggagcataaccgtg

tggaaacttcctttagagaagatttttctaaattcttttctcttgttattttcttataagatttctattctaatcatgtt

gtgtgctacaatctccatgtttgagtagttttcttgttaggtttagggtttctcattagggatttagatgatttagtaga

ttgcccccttgctaggttatctgttaggattcttcatctttaattgttcttaatgctagttctagattagctaactagaa

cttgaatgcagacaatagatatacccgctataggtatctatagttggatctattatttgatgagcctggtttataggtgt

gtaataaaaatcggcctatctactaaggtgaacaattgaacttgattagaatgcatgtttaagtataagatctctcggct

tctccggttattcttagctataggcacagggagtttcgtggaacgccaccggttattccaaaattagagtttgagtttaa

gaacggttcgataacaacctagctatcactatatctactaatcatctatctcccaagaataccctaaacttgactctttc

ccatcttatttacatcttaatacttattgcttttctttatttcttattttataatctattggtctagcttaattgaaatc

atcataagtctattgtgtgaacatcgagggttcaatggaattcgagctttaagtgctaatgacgatctcttattttaggg

agtaagcttaaggcaatttgagctatatcacctcccaaaattttcttaccaaaaaccaaaaccttaacacaaatgacaca

atttctgatatatagagttagatcgtcgactcttgtgagtgatgcttttttttcgtttgagtgttctcattttgaaatgt

ttagtgaagataacgaataaagatatgattgtatataactatatatggtggaaacattattcacttgtcatttatactct

atacatatttccaaatatgtccaaaacttgaataagctaacgtatatacagataaagacaaacaccatctcaaacccaat

aaaattagtctgtttacacgcattcatccagaagattatgccactgcgaatgacaatactttaaaccttttgatttgtga

atgacaacactcttgctactttcacgatttccaaagatttatcttatagtcaaattttcataatataatgctagcgtgta

aattattttggatcaatataaaatagagtattcaagcttgataagtgtgcattttacacgttttgagcatccatttgtca

tcactttagcatcatatcaccactgttttataccatttctcatcatttgtcatcactttgcatggttaggatagttttgc

atgcatattgcatatttgtgttgtgttcaggtaatttggagctgctgccgagctaatttgaagaggtggacctgatcaaa

tcaacatactcgaccacgagctcgagtaccgtcatcctggagatcctgaaatactcggccacaacttccacaacaaatca

ctcgaccgcgacactctatccaccactcgaccacgaggtcgagtagatgacttccccacttcacaacaccactcgacccc

ctggtcgagtagctaccttcaccgcttcgattcaccactcgactacatggtcgagtcccaccatcatcattcactcgacc

aatcactctaccaacaggtcgagtatcaccatcatcattcactcgaccaatcactctaccaacaggtgcagtatcaccat

catcattcactcgaccaatcactctaccaacaagtcgagttccaccatcatccaccactcgacatcatactcgattgcca

gcagagtcagaacaaaccttttgttccctacaatcaaggctttgctcccgaacagcaatttcaggagaactaccagcaaa

caccacccaggtttgccccacagcaacaccaaggtccttctgcacctgatgctgacatgaaacaaatgctacaacagctg

ctccaaggacaagcatctggctccatggagattgctaagaagatatctgaattacaccataagcgacattgtagctacaa

tgatctgaaagcaaaagtggaggccttgaattccaaagtccgatacttggagggctaatcaacatctacctctactccca

aagttacaggactttcagggaggtcaatacagaatccaaaggagtatgtcacagctcatgttatccctatctgccatgat

cgagagctgccaactcgacatatccctacctccatcactgaggacagttaaattcaagaaggggaggatttttgtcagaa

tgaagctttagttgaaaacgcaacagacaagcccccactcgaccacactactcgatcactagctcctgttgaagaaccag

atgttgctaataatatggaaaaagtctttgttactcctccttacaagcccccacttccatttcctggtcgacacaagaaa

gagttagcagataagtacagagctatctttgccaagaacataaaacaagttgaagccaggatacctcttgttgatgctct

cacgcatatcccaaattcccaaaaattcctgaaagacttgatcatggagaaaattcaagaagtttagaaaacaacaatta

tgagccatgagtgcagtgccatcatacaggagaaggatataccagagaagctaggagaccctggttcattcactctacca

tgctccttagattcattgacttttaataaatgcctttgtgacctaggagcatcagtaagtctcatgccactctcagtagc

taagaggttgggtttcaacaaatataaatattgcaatatctccttaatcttggatgatgggtcttttagacaacctcatg

ggttattagaagatttgcctatcaagattggaaatgtggaaggtgcctatagacttcatagtgctgaacatggatgaaga

accaaaagatccctttatcttaggaaggtcctttttggctacagctagagcaatcataggtgttaagcaaggaaagattg

atcttaatatggggaaggattttaaaattaagtttgacatcaatgatgctatgaagaagccgacaatagaagcacagacc

ttcttagttggggaaaggatcggctagttgatgaactgctagtggaacttgaagaagcaaataactcaaaaactgtttta

atcaagagtggtaaagctgggtatctccctaatgaaactttgaattctgaaaagtcattagactcacacaaggcagcagt

tggttcagaggtttacaagggcttgatggggtctaaaccaaatgatatgagagctaatgaagtaagttcaacacatactc

gaccaactgattccaccaacaactcgaagaagcctccaactttccctgagagctcatgcttgaccgaacagctcaaggca

agcactcgaacgtcagatgattggttagaactcaaggagagatctaaatggcaggacaaagccatacaagagctaacata

tattgtgaaagagatgaaggatcgaatcaaggagctcaatggaaaagctaaccaagtaccactcaacatcaaggacgtcc

cagattatggagccactattctggtcagcaaagaaagatgtgaattcacgttggaatggtctagtggagaagactatcct

gataaaaaagaagcttacttcgaggacatggccactaagttctcaattgcagacctgtcaagaaacactaatgagtacga

tgatcaccctactcaagaaggagaggagacctcatctcctctctgcaacccagcctaagccaaatgagtatgagaagtca

agcttggagacttaaaacaagctcacttgggaggaagtcccatgtctgtctttgtacaaatatctatttttccttgttat

ttttgatgcatgttattagtgttttcaggagataagtatgtagagctgagtaaagtggattctggctttgagagtacaac

agtgtactcgaccatagacaaagaaaaatactcgaccatagacaatgaaaaatactcgaccttattttggcttaatcacg

ccagaagatcactcgaccgcggtgctgactgtagtagaagaagaggggtcgagtatacttgtggcggtgctggccgtagc

agaggcgatgagctcgagtaccctcagggagctggagctgagacagaacagggagacacttcaatgggctgggagaattc

acatgcatccattgatgaccaactccattaaaacttgactgaggtaagcgcctcacttcaccattgtactataccatctc

ctgtttttattttgtttctgtgatgtggtttgtcatgagtactctcttccaaatttggttacacagtggactgtgtgatt

taagtttaggggagggctcaggaagtgtgtgttacattgtatatactcttgagtctgcattcatctaaagcatagaaaaa

ccaaaaaagttttgaaacatttcagaaaaatatttcacaaaaacagagtgttcatatagttgcattgcatttaggattga

atgtagaatgtttcatataggattgttgcatatgcataggggataatgaggagataaccttgtaagcatgatgattcact

aaatgagttattagttctcttatgcatttgaatgactttgaagtaaaaccgcaccatgttctatagaaaccactcgttgc

atgtcatgacaccttttcctgtcaatttgaacttgaatctgacttataattatcatgttagcatcgattttaaactcatg

gctagaacacatatttggattttctttaatttattcatcactcttgctaatccaagtagttgcttctattattggagtag

ttccccctcccttaaccaacccttctttcaagccatgttattgtgagagcatatgaggcctattttcaggattgagtttg

gtagaacgtgttaggtttcagccgacaagagtagtatcccatgtagtttgagtctgcgttttcggacttggtaggagtag

gtgggaacttatttgcagattgagattgagtgtgaaaagaaaaaaaaaaagaaaactggttagaatctttaggagaagga

agctgagaatcatattgaggtctagtgaatgtttgggaagcatagaattgataagatataccctaaaaaaaatggcaata

aaaacagaaaggaaaagagaaaaaacaaaaatcataagagccattagagtttaagtaaaaccaattcactagattagact

catagactttaagaaaaacttctccttaagagcaagagcaaaaaaaaagaaaaggaaatgagttgatccaagaaagaatc

gaaatatgcctttagtgcaaaagggtagacttaggatcatcattgggtttagagataggaccatctacttgttattcacg

gtttacgtcattgggtagatggggtgctattcttgtatgcataggttggtacttatctttagcattcttctaaagcccaa

tcctttttcattaagagttccctgttattaaaatctgaaccaccttaaagagaccatctttgtctcataacctgtcctta

gccaaatgagttcactagcgacgcatatcttgattcatgttcatgaacttaatgaatgttaaagggattggtcgatcttg

acatattgtgcaactgagtgtgaatttggatcataacagagaatggctatagtttttaagtagaagtcgatcacctcgca

tcttagaactgttagcttatacattaatcttaattgtcatatctcatgctttgcttgtgagtccccgctttcaaaactca

cctctagcttgttcttaattgtttgcttgagggcaagcaaagactaagtttgggggagtagataagtgtgcaatttacac

gttttgagcatccatttgtcatcactttgcatgtttaggatagttttgcatgcatattgcatatttgtgttgttttcagg

tgatttggagctgctggcgagctaattggaagaggtggacctgatcaaatcaacatactcgaccacgagcttgagtacca

tcatcctggagttcctaaaatactcggccacaacttctacagcaaatcactcgaccgcgacactctatccaccactcgac

cacgaggtcgagtagatgacttcaccacttcacaacaccactcgaccccctggtcgagtagttaccttcaccgcttcgat

tcaccactcgactacatggtcgagtaccaccatcatcattcactcgaccaatcactctaccaacaggtcaagtatcacca

tcatcattcactcgaccaatcactctaccaacaagtgcagtatcaccatcatcattcactcgaccaatcactctaccacc

aagtcgagttccaccatcatccaccattcgacatcatactcgactgccagcttaagagtcttctcgattccgcactcaac

cagacaatctagcataaggaagaaaagaagactccagcaaatcactcgaccaatcactggaccacctaggtcgagtatcg

ttcttaatccgtcccaatactgcgtcgttttgagtattagggtttcagaacattttgctataagtagcttgtactttata

ttttcgagaaaaaagttttcttccgcagacactgtgtgttattgatattttgtaatccagatttctcttaatttattcag

tatttgctttcattttgtttactaagttgttcatcctgttatcatcttgctattactctgttgctatcatgttttcattc

ttaccatcgtttatgctttctacaatgatgtctgattagtgactaggtttctgaggatgggttagagtagtttagaattc

tcagtatgctaggtggttgagtttgatttatagatcccttctacattagttgttcttaatgcttattgctttctgatcca

ctggaatttgagcccagccatttccgcacccaaaaggtgttagatgaaatgtctgaaccactaattccagagatttgtgg

ctatgtaccaaggtattggttacatggagcgttttggctttaactttttgattcgtaatgcctgttaggttagctctcgt

caatggtgatagagtttgggacgtgtttaacttgagggtctctgttgcggtggcacttagatttagttatcgaacttgtt

gtctagggataatttattgagcatgtcaatcacttgtaaactgaggaaacgaaactactcaattaccccatcctcgggaa

ttcctcatctaattgatttctttgtttactctgctattatttactgcatcttgctgcttgttgtttagttcttgcatttt

atgcgtgttactcgacctgatactcgaccacccaattgtctagaaacagtctgtgcaatcgagtatccttgttatatttt

ctgtctgttactcgatttgtcactcgaccaaatcctgtgcttggtaacaagctgtgttggtcgagtgttttaatgtttct

gcttgaatttctgtttcctgcatgttcacttaggactgctagaaaccccaaaacctgttattgctcggcttgactttgtg

aactctgattgcatcttgattgctagcatcacacccatttggattgagaacctaaaatactacaacgacatgattggtgt

ttagaataattgattaaaaacttattatcaaatctcttgtggttatcgtggtaaaaatgaaatccaataattattctata

aaataatatttcgatataagatcgatacattaaattaaaactccaataaattaattcagaaaacaataaacagttacaat

aatttaaaaagggataatagaaccccatgtatattacaaacattttatccataaaatataacactttaaacactaaaata

attcgacatttttcgatatctataaccactcaatcatcctatacaagatttccaaaaagaaatgttaattaccaatattc

gacatagcttagagaatttcacatattctcgatttcttacatacgatgaaacataacgtacttattacaaatgatttata

catcaattgtaacgcataaaaatatcaaagttataacatattttggtagcgttatccataaccactcaataatccaagtg

taatcatcaaaaaaagataaaaagtttccaacataatgatcgtatataaacttgcaaattatatatgcgcatttttcaag

atcaaatctttaacatcaaagaataattctacaacatcattcaacgaaacaatgcaaataactttgtaataactataact

ttgatcatcctttgaagtatttaagtattttacatacaaatttgtaatttaaagggtttacaaacgaaataaaaaaacaa

ataaagatttataaacgaatttgcaattttaaagaggtctaaacagaagaaatataaataataatgatatggtttaaagt

ttttttgtccacacaatgagttgaatgtcaagattaagctagtagagattgattgtaataagaagtaaacaaagtaaaaa

gacaacggattgattggttgtaaacgataaaataaagaggtaggaacaaggtattctcaggagactattggttagtagat

ctaatgaaagctaggttgttatcgaaccattattaaacacaaattttaattatggaataaccggtggtgttctgcaaaac

ttttgtgcctatagctaagaataaccgcagaagccgagagatctttaacctaaacatgcattctaaacgagttcaattgt

tcaccttagtatataggccgattcttattacacacctataaaccagactcatcaaataatagatccaactacatatacct

atggtgggtatatctagtgtctggattcaagatctagttaattactctagatctagcattaagattaattctacacataa

tttagcaagggggtgatctactaaaccatatgaatccctaatgaaaaactcaattcctaacaagaaactactcagacaga

ttgattgaaacaaacaacataaatgaataagaaagcataaacacaacaaataaaattagggaatgaaaggatctcttcac

tgtaatgagaactgaatgaatctctgaagaacaacggatgattagcttatgtctctctgaaaatagggattaaaaacttg

ataaaaggaacttaggtctaaacaatgacctttaaaactatatataaaccctataaaacgtccagggactaataatgcaa

atagggaagtcttttggggcaaatttccacttttgtaaacttgaaagcgtattggacttttctgggccgaaactggtgtc

gatctctgaatctgaaatctgaaatcatccttagctccctttccttagcttttgctccaaaatgtctcctcatctccatt

gttgtcccactgcatagaatacctgaaaagactctaaaaagactcgagaaataacaaaaagactcaaaatcctaaaccta

aaacatagataaaatcaataaaaacagggttatatcaactcccccagacttagcttttgcttgccctcaagcaaatcaca

aaaacagaacccgtggaagaggttttgaaaacaaaggaacttccaacattctctagcctattgtcatgatcatccaaact

aagtccaaatgcttaacaagtttaatcaaatcctaaccaacatgtacttctctgcaagcttacacaactttctaaattcc

atacctaagttccacaactatatccaccagggtttcatcgttgggtctcatcaaacaagctaacattcaagatccttcta

tactcattcttgtactataccatgatgaatcgaccattctttatatatatatttttcattggtgttttttctctccccag

acttattcgttttagagagtaaacaaaggagaactcgcatactggatcgacaacctcggtattttccaacctttgcaatt

gatcattccaaataatgtataataatggcgtcataggaaacattcctacccctatacactatatgaaaatcctgtcaatc

tctcattttctatttttataaggggagagacacattctttttatcacattggtctcaatatcaggtatgaacaaggagtc

aagatctatgaatactaaccgtttgaataggtgaaagaaagagcagtaagattagctccagccttggtggttatgttgga

aactaggatatcctcaagtctgtttgtttgccatctagactttccaatagtcggactgagtcgaacctgtagcactcatc

aaccagacaataattaagagaaatatagatcatgattcctaactaaaataaagaaaaatgtatttttttaaaagaatgaa

agaaatgactcaataaactgaaaaacgaaataataagaataaaaaataaacgcataattaataagaacctcctccagact

taaacgacattgtccccagtggcattcaaatccgaaaaagcgagaaaaataaatcaaaaataaaagaaaataaaagaaag

aaaaagaaaaatcaaacccgtgggttgcctcccactaagcgcttgtttagagtcaatagcttgacttggcggagttcagg

atgtgggtggagtggtggaagacttttgctgcctccttgccttccaaggcggtgtgtaaacaaatcctgtctgggcagct

gcaggtgttgatcaaaaatcatggtggtgtcgatcgacactctcctcgtttcctgcaatatcaaaaggagaaacaagctt

tcgaagaattctaggcttgttaacttgtgaagaagacttcaaagaatctaaagaattaatcaaaggtaaagcaggagaag

agatagaagagaacttagaacaaaggtgcacaatgggtgtaggttgtagagagtttgcagaatcttctgtctgtgaaacc

tgagggtgtcaatcgacatgagggatggtgtcgatcgacaccgattctctgcagttctcattggcgagctgagctgatgg

gagcattgtgtgtagctttgtttggggattctgagcagtctaaggagatgtgtcatggctggaggcaatgctactgatag

ccctagtgatctctgaaccatccataccaaattccatctccaaatcacaaatgttaagagagatctttcctctcttcaca

tcaatcctagcaccagctgtagttaggaaagctcgcccaaggataagaggatctttaggttcctttccataatccaagac

cacaaaatcagcagggattaaggaattaccaaccttaacaggaacatcctcaaggattccttcagggatttgtttggaac

aatccgcgaagacgagagtgattctagtcggcgtgtagttagtcatgcctaatctttcagcagtggtctgatcaggagtg

atagtgcaacgtccactctctgtgccttctctttcttggcctttggtagcatctttttttattttttcttatttattttt

ttttaattttaaatttccattaaaaagttacatggcaacagcccaacaaggcacacatgcacaaaagaaaaagaaaaata

aaaaaggaaagaaaacttacaagcccaaaggccaacaaagcacacaggcccaaaaacaaagaaaggaggaagcctagaac

agaaagcagcggatggcttgagaccgaagaaaaagcacctgaaacagagaacgaagaagaagaaccaaaacagagaagcg

gtgactaatcaactttggaacacagcaaaccaagtcatcagaagcgagtcacccacaagaccctgacccatcctcctaga

aagtggatcaagacgggcccttacaatgagctggatttccttaatgagctgagtatgattcaaaaatgaggatgtgtgaa

tccgagagttgcgctccctcaacaacagataaatggaagcctaaaagaggagcttgattataagtgataggttctttgac

gtcgaagccgtcttcaaccagaggagagaagccatgaaggacgttggaggagatagtccaacacacctagtgaaataagt

ccagacagcgctgctatacgggtagttgaagaagaggtgttctctagactcatcccctgagttacagagcacacacactg

caggaatagagaaaccccaacttttcagcctatcccttgtatgcaaacggttccaagccacaacccaagagatgaaagca

tgcttggggacccgatccttgaaccaaattgatctatgccaattaacctggacacccggagggtgaagagccatccaagt

ctttgaagcagagaacgagtttgaaggagcatgagcatcgacttttcacagataaacatcatctttagtacactcagcca

tactccccacaaccggtaagcagggctttaaaagaagaatggaggggttacgactcctactagcagacagccatcaaaca

ccacctcttaagccatcttttacctccgtattcagaggcaaaccaactaagataggtccagtaggccccgtcagattaat

gagaagaccaagactcgttcaattgtcttgccagaaacttgcagtttcacctgaaccaacatcacacatcaagaagggtc

tcgctaaaggtggtatcttacaaagcttcctccaaatctaactaccagaggaagacggattcaaatcccaaaagcatttg

tgtctaatgagattaattctgacccacgaaacccacaaggaccctgcagctgtaaacaataaccaaatgagtttgagagc

cataaccttgttccaatctatcaaacgtcgaagacccaacccgccacactccttaggggaacaaactgtttcccaagcaa

tttttgctcctcaaacggagtttggagctgctttccataaaaaagcattacacatctcctctagctttgtgagacattgg

gtaggcagcaggaagatcgaaacccaaaagttaattgttgaataaattacagacttgagtaactgtaatctgcccgtaaa

ggaaagatgcctagtagttcaagtagagaacttaaatttgatcctatccaaaagaggctgataatcatgttttttttcat

cttttgggccattaggggcactcctaaatatctgactgggagggctccttgtttcatccgaaaggtagccgctaaagaag

aagacctggcatagtctcctccatctagcataagagcggttttggacttgttaatgcctagtccagacccaagcttaaac

gcctccagaatgttgagaatccctgcaactgggctcatggacccatcaaagaaaaccaaaacgtcgtctacaaaactgag

atgagtaataaagggagccacacacctggggtgaagattgaataggccattgatagcacccctatccagtaatttagcaa

gaatatccattacaagcacgaacaggtgagatgacattgtgatctccctgtctaataacctttttactacaagaaaacag

acaaataacgactacaaaaagcgacaacagatgtcgtcgtcatttttggcgactacttgacgacagtgttacgacaacta

tacgacaacctgaacattagtagtcaaatagtcgttattttacgactaacttcgttagtcggcaactactcgtctattaa

cgactatttaactattatattattgaagggagacgtttgtcgttacatagtcgccaatgcacgactacaatttccgacta

cgatagttagtcgcctgattggcgactaaaataatacacatttctgcaatcgtaaaatagtcgcaacttagtcgcagagt

agtcgacttaagcagttggaaaacgttttcgcattgccaagtaggtacacctaatttgtttgttgtagtcgtaaattagt

cataaaaattcgtatatataatcaaaattcaggacactcttctttcaccaagaaatcaaaacaacaatcaaaaggaaaaa

aaaaacgattgaaacgaaaaaaaatagaaatttagtttattctataatggcggaaaactacaattacagcggtagtggca

gtttctatcgagattggatgtacaagagattcgatgaagtgacggagaatttgtcagccgaatacgtcgcaggagttgag

gaattcatgacattcgctaatagtcagcctatagtacagagtagtcgaggtaaatttcattgtccttgtagtgtatgcaa

gaatgaaaaacacatcatctcaggtagaagagttagtagtcatttgtttagtcaaggatttatgcctgattattatgttt

ggtataagcatgcatggagaagaattgaatatggatatcggaactagttatacgaataggacgtattttagagagaatca

tgaagaagtgggtaatattgtagaagatccatatgtggatatggtgaacgatgcatttaattttaacgtgggttatgatg

ataacgtggggtatgatgataactatcatcatgatggtagttataagaatgtagaagagccagtccgtaaccattcaaat

aagttctacgacttgttagaaggtgcaaataatacattgtacgattattgtcgtgaagggcagtcgcagttatcattagc

atctcgactcatgcacaacaaagcggggtataatatgagtgaaaagttggtggactccgtttgcaaaatgtttacaaatt

ttttaccataaggaaaccaggctaccacttcacattaccagaccgagaagttgatgcgcaatttaggacttccatatcat

acgattgatgtttgccagaacaattgtatgttattttggaaagaagacgaaaaagaagatcaatgtcgattttgtggcgc

acaaagatggaagcctaaggacgaccgtcgaagaaccaaagtaccatatagtcgtatgtggtatctacaagacagctgcg

gctatgcgatggcatgctgagcaccaatcaaaggagggagaaatgaatcatccttcagatgcggcagagtggagatattt

ccaagagttacatacctggtttgctaaagaaccccgtaacgtttatctcgggttgtgtattgatggctttaatccatttg

gcatatctcgtaatcattcattgtggcctgtgatcctaacttcatataatttacctcctggtatgtgcatgaatacaaag

tacttatttcttaccattctgaattctggaccaaatcatcctcgagctagtcttgatgtcttcctccaacctcttattga

ggagctaaaagagttgtggtgtactggagtcgatacgtacgatgtttcattaagtcagaattttaatctaaaagcagtac

tactgtgggcgataagcgactttccggcgtatagcatgttatcgggatggactactcatggtaaattgtcttgtccagtt

tgcatggaaagtacaaagtctttttatctacccaatggacggaagacatgctggtttgattgtcaccgtagatttcttcc

tcatagtcatccatctcgtaggaacaaaaaagactttctcaaaggaagagacgcttctagagagtatccacccggatctt

tgacaggggagcaagtttattacgagcggttggctagtgtaaatccacccaaaacgaaagatgttggtgggaacggtcat

gaaaagaagatgcgaggctatgggaaggaacataattgggacaaagaaagcatattgtaggagttgtcttactggaagga

cctcaatcttcgacaaaatattgatgttatgcatacagagaagaattttcttaacaacatcatgaacactctcttgagtg

ttcaaggtaaatcaaaagacaatatcatgtcaagattggatattgagaaattttgttctcggccaggcttacatattgat

agtacgggtaaaactccttttccagcgtatacattgatagaagaagggaaacagagtttatttcaatttgtgaaacatga

tgttagatttccagatggtcattcttcagatttggctagttgtgtagatttggagaatggtaagttttcaggcatgaaga

gtcatgattgtcatgtttttatggagcgactacttccatttatctttgctgaacttcttgaccggaatgtccaccttgca

ttatcaggtacaatactaacatgtttttatgtaattatttaacttgcacatatattttaggaattggagcatttttccgg

gacctatgttcgagaactttacagataagtcgcgttcaaattctcaaacagaacatagttttaatcatctgtaacttgga

aaagatctttccaccatcatttttgatgttatggaacatttacctatccatctcccatacgaagctgaattgggtggccc

tgtccaatataggtggatatatccatttgagaggtttttcaaaaagttgaaaggaaaagcaaagaacaaaagatatgctg

caggttcaattgttgagtcatatatcaatgacgagattgcttatttttcagagcactactttgccgatcatatacaaaca

aaatcaaggtaaatacctttatttatttaacaatttgctatactacttcgatcttaattaaattatatgtatcaggttaa

caagattcgatgaaggtgaagttcctgtatatcatgtccctggagtacctaacatatttatgcaggtcggccgtccaagt

ggttcaatgcatgtagaatggcttttagagaaagattaccaaaatgcacatgcatatattttaggaaattgtgactattt

taaaccatttgagaggtatttttaatcactttgatttattcataggtaagatcactttgatttatttgtctttttttgtt

gagactgtatgttcgagaattatctttctgcgaaatatccatccttggccgaaaaagaactctacgtcagaagagctgaa

gaatatcatctatgggttaaagaatatgcaagtctacattaattttggttcatgtaactatttgtctgttttcttgtagc

tagtgaatatgtaagtctacattaattttggttcatgtaactatttgtctgttttcttgtagctagtgaatatgtaaatc

tacattaattttggttcatgtaactatttgtctgttttcttgtagctagtgaatatgtaagtctacattaattttggttc

attccgggacctatgtccgagaactttacagacacactagatgattatacagccatctttctcgaggtaagatgttgtag

tctaattatgactttaagagtccaattaatattctgtcactaatgagcttctcctctatttcttgcagtccacagaaagg

gattcaagaggcaatccttatggacttggatgtctaaaagacactctaggcagtgccaaccgccaacactccggttcctc

atcatcctttcaagccctagaagaatggctgcagaaagctcaaaggaaaatagaagagcaggctgcatataatgagaaga

gagatgctgagatgcccgagaagctgagtcagcccgagtcacagctgagcaaaatgacaagctcgagcaattgtctttag

tcgagaagtatctccgccaaaccgatccgcagttcttggacttcatggccactcactctaccacaaccacagaacgtcta

ccaagtcctccacctaatgatccttagattatctttgcccaaacactacttgaacttatgtttatttgaataagttgttt

gtgtaataaataagataattgtatgtgtatttgaacttgattttctatatatacaagtatgtcttttcatcttttatagc

atttcaaataaaaaacgagaacaaaccataactttaaacaacaaacaagaaacccaacaatcgatgaccaaattcttatt

cggacaaaaccagtagtcgcacaatagtcgtatttaggtagcaaattagacgtaaaatagtcgtataatagtcacaacag

ttaccgattattatcagactaaccaacctagtcacaatataatagtccattagacgtataatagtcgtaaaacattatac

gactattatatgacaacgagaattatttccttacattgatcgaattagtcgtccagtagtcgtcaaatcgtcataaacac

ttatacgactattatacgactacgtgtttcacacgctctgtacaggacaatgaatcgtgcaatggtcgttaaatagtcgc

caatcttatacgactatacaacgacgattttgtactgattgggtttttgtcgtaatatagtcgtcgatcaacgactaatt

cacgactaccttactcagcgactatgtaataatgactagcatctttagtcgccgattagtcgtattatcattttttacga

ctactatgcgactgctgtgtaagtcgtttatcacctgttttcctgtagtgtttcccttggaagaacccaattagctcctc

cctgaaagcaatactatatgatggagtttggacgcaaaccttaatccaactgatgaaggcagcatgtaagtccaaagcaa

taagaatgttgatgaggaattcccaattgacattgtcaaaggctttagagagatcaatttgcaaacagccacaggtaact

ttcccatctgcctgaaagttctcaaccaactctgaggctagtaaaacattttcacataaaagacgacctttgataaaacc

aacctgattcccctagactgcttgagaaatgaagagctttaacctcttggaaatcagtctagtaattaccttgtagattg

ttttacaacaagaaacagggcgaaactttgagagcacatcagcaccaatttccttaggaataggagtgatagccgtggta

ttaaactttttgagaaggtgccctgtagtaaagaactctctaattgccgcaacagtgctttccttcactactgaccaaga

atcccaatagaactcagccgggaacccatccggaccaggagccttgtttttaggcatagagaaaagggtctgagtgattt

cctcgtctgatggaatggcaatgagcttttcagcaagagtagagtcacactaaaaggggtaaatttcccagagagtctcc

accgagtaaggggtagaagttgagctctccgttcccaaaagatgggtgtaataagtcacaatcatgtccttaacatggtc

aacattttccactctcaacccatcttccctcctaagataacgaatgatgtttctagcttggtgaggaaggacaactctat

ggaagaactgggtgttggcatccccttcttcgagccacttaattctggacttttgtctgaaaaaaagactccaatacctt

agcaaaaaaattccagtttttcctcgcaacgtgctcagccctgaagagcgaatcaaccggagcaaacaacaactaggact

gaatctcttccaatttagcaagggcctctgtcgttctctattgtaagttactgaaaccttccctcttcaaaacccgataa

gctttctttgcaaccttaagatgttccccaagagaaaacatgtaagagccaactgtaatctcacctacccatgcagccaa

aatacgagaaaaaaactgagagtggatagacagaaaggagaagtacttaaaggatttttttatctttccggctggacttc

gatagaatgatacaaggaccatggtcagagtcccccggagggtcaaacactgccatagaactatggtaggaggccagcca

caggtcattcacaattgctctctcgagtttcctgataataggattctcggcttgatgattgaaccacgtgtaaaacaccc

ttttgcttggaagatcaatcaactgagtaatgctcacctatggaagcaatctgattgaaatctcctgccgcaatcaagga

gaattactaagcggagacgaaatggcaagagtgcacaaatcagaccagagctctcttctttccacctcacagttgcgacc

atacacgaacataacaccaaaagtctgattaatattgggaagctttatagcgcactaaaggggaagcagcaaagcagttt

actcggccctttactctatcaacaccagaggggaagcagcaaagcagtttactcgaccagtcactctatcaaggcagcta

cagaggaagcaacagacaaccactcgaccccctactcgattctctactcgaccagttactcgatcaagggcaacatactc

tacaagacttgttgatcgttcaaagcgtcttctgatcctcctcattccccactcaaccagacatctgagcataagaatag

gagaggaagaagcaactactcgaccaattgatcactcagagcatggaggcaaaggcgcagactttgcaacaccctcttta

ctcgatccccaagatcgagcatccatccaaaccgcctcaagtctcttatcttctagtgaatctagggtttttgagatttt

agtagaaataggttctttggggtttgtccaaaggatcaagcttttatctcttagtttttctttgtgtttctgaatcttga

aaccttaattactcttaatctattttgtttttgcatttagttctttgagtaaagttgtttagcttgtttagatctatatt

ttgatatgatttcagtaatttctgggtttgtgttcttagttatgttacttgagtagtgattagtgttcttgaggatggat

tagattagggtaatatctaggttagtcaggttgttaagattaggttagatcccttctggattcgttggcccctatgcggt

tttcgttggctaatgtagaaatttgaccataggtatccaacgttcaaaagacgttcaaatggatgcctaatctacgaatt

ccagagatttgtagactttagacctagagattggttagactgcttattgactttggtagttgatatagctcaaattgcct

taagcttactccctcaattaagagatcgtcgttagcacttaagggtcgaattccattgagctctcgatgttcacacaata

gacttatgatattgtttaataagctaaatgaagtaattgaatattaaaggcaaataagcaagtaaatgagttgtagattc

aagtgattaaagcgtcaggtctaaggaattatctcgggagatagataaattgtagatctagataatatcaggattgttat

cgcaccgttctcatactcaaactataattctagagtaatcagtggcgttccgcaaaactctctatacttacaactaagat

aaccggagaagccgagaaatcctatgctaaagcatgcattgttaataagcttgaatagttcacctagtatctaaaccaga

gcccttctatgaacctacctgttttttcttaaatgcctaggctcatctatgatggttcaaataacaaatacctatggcgg

gaatacctattatctaatatcaagttctaggtgatcaatctaaaactagcattaagaataatcaagatgaagaactataa

gaataatcctaaggggttttcgatctactaatccatctaaatccctattgagactccgtagacccaacaaggtgattact

caaacattacaatagaaacacaaatcataatctgaatagttttcattgatgaattaagaataatagcaaaggagttcaga

atcctctctttttagggattggattcttttctctaaagtgtaacaaaagctcaagaacaaaaactaggtctaacaatgac

caaactattaggtatttaactctaaaaacgtgcagggactattttgaaaataaggaaaacttcagggcttctcaaagact

tggtgtaaaatgaatattctcctggcagcgacaagagtgtcgatcgacacaggtgtggtgtcgatcgacaccagcttctg

aaagacgtccttcaagatctttattgctgaaaagcttccaaaattccttattgtgctccatgtttcccagatgtgtgagt

gtacctggaatgacttaaatcgtacgaaaaagactagagagatctctaaaaattattctgaaactgtgttagaaatcagt

aaaaaccgggacatatcaattcccctaaactgtgttagaaaggttgaatctgcgaaagaggtttgaaaaacatagaaact

cccaatctctctggtttattaccttgatcatacaaaccaaatccatgaaataaacagttctaatcaaatcctgaccacca

tgtaattctctgctagcccacacaacatcctaaattccataccaaagcactcaactatatcaactaggctatcatcgctg

ggtctcatcaatcaagtgaatgcttaagaaccttctagactcattcctgaactataccatggtaaatcgaccattctttt

atttttttctttggtgttctttctctcccccaaacttattcttcttagagactaaacaaaggggaactcgcatactggat

cgacaccctcggcattttccaacttttgtaattgatcgttccaaataatgtataataatggggtcatacgaaacattcct

acccctatacactatctgaaagtcttctcaatctttctttatttattatcttctttttcttttttttggggagtggaaca

ctttctttttaacatattagtcgatctatcgggtacgaacaaggagtcaagtcctatgagcactaatcgttttgatgagg

taaaaggaagagccgtgagattagctccagcttttgtggattatgttgggaatggatgtccttaagtttgttgattggcc

ttctgggattttcattagtcggattgatttgaatctgcagtacttaaacaatccagggaataaactcgagagatagatca

taatttcagacaaaagttttgactcaataaaatgactatctaagttaaaatgacacaactgactcataagaaaagaaaag

atgaagttagtaactgactcccccagacttaggtaacattgtccccagagttattaagctagaggaatcgaaagagaaat

agtcaaaagttaaaaagtcagtaaatgtgagttgccacctgagagtggtgtcgatcgataccatgatggtgtcgatcgac

actctggtcatttcacattgctttgtgaattctgctcattgttgtagagccttggttagtgttttcacatggatcctcca

gttagactgttaaatcactaaaacacacatcaatagttccaacaaagcataagaaaataagtgcaacaagttttaaaaca

aataaccaatagtctgaaataaagcagaaacaaagttagaaaataaaacacgataaggagggtggctagtcctcatgctc

tgagccggaggtgggtgtgggagagcgacgacgacgacgactgggggcaacacaaccgcagcgagaaagtgtagctatgg

cgttccagacggtcttgaagctgtcaacgacgtactgctgaaagacctgtggctccatcgggatgggtggaggaaccaag

tgatgagcgaacacagcaccaaagttgacattacggtcaacttgctcaaatccaaggctttcaggaaagaagtcatgaag

agcgtgatagagcagtaacaactcagagagtctcatcttgccaggcttcatcttgcacaacagtgtgctactaatcagcc

taaccaaataccgtagaacagggtggcggatctctgagtgaactgcgtccttggaatcataaatttcattaccaaaactg

ctctagaaaatctgagaatccttaaactgaccaggcagggaaactccattgagatcattgtcaaacccatatatgtcacc

caaatctcgcagtggtaaactgtatctgacaccgcaaatgaagaagataagagctccttcctgaccgttgcgtttcctgt

catgcaaataagaaaccctaacagaggccatgatacagatcgtactgcctacaacagatggttcccaaacttacgtcatc

tagcagccctaaaactgatttctctatctgcaaacgctgcatcaaaccgaaatccgcaaatcgagtaggcaaaatttata

cattcatccaggcattatagaatagagtatcataatcatcccaatctggagaaaagggcgattgatatagtcacctttag

tgaattccttgttgatatctttctcccaacccctttgagaaggaattc

**T15D9 sequence**

aagcttgaatagttcacctagtatctaaaccagagcccttctatgaacctacctgttttttcttaaatgcctaggctcat

ctatgatggttcaaataacaaatacctatggcgggaatacctattatctaatatcaagttctaggtgatcaatctaaaac

tagcattaagaataatcaagatgaagaactataagaataatcctaaggggttttcgatctactaatccatctaaatccct

attgagactccgtagacccaacaaggtgattactcaaacattacaatagaaacacaaatcataatctgaatagttttcat

tgatgaattaagaataatagcaaaggagttcagaatcctctctttttagggattggattcttttctctaaagtgtaacaa

aagctcaagaacaaaaactaggtctaacaatgaccaaactattaggtatttaactctaaaaacgtgcagggactattttg

aaaataaggaaaacttcagggcttctcaaagacttggtgtaaaatgaatattctcctggcagcgacaagagtgtcgatcg

acacaggtgtggtgtcgatcgacaccagcttctgaaagacgtccttcaagatctttattgctgaaaagcttccaaaattc

cttattgtgctccatgtttcccagatgtgtgagtgtacctggaatgacttaaatcgtacgaaaaagactagagagatctc

taaaaattattctgaaactgtgttagaaatcagtaaaaaccgggacatatcaattcccctaaactgtgttagaaaggttg

aatctgcgaaagaggtttgaaaaacatagaaactcccaatctctctggtttattaccttgatcatacaaaccaaatccat

gaaataaacagttctaatcaaatcctgaccaccatgtaattctctgctagcccacacaacatcctaaattccataccaaa

gcactcaactatatcaactaggctatcatcgctgggtctcatcaatcaagtgaatgcttaagaaccttctagactcattc

ctgaactataccatggtaaatcgaccattcttttatttttttctttggtgttctttctctcccccaaacttattcttctt

agagactaaacaaaggggaactcgcatactggatcgacaccctcggcattttccaacttttgtaattgatcgttccaaat

aatgtataataatggggtcatacgaaacattcctacccctatacactatctgaaagtcttctcaatctttctttatttat

tatcttctttttcttttttttggggagtggaacactttctttttaacatattagtcgatctatcgggtacgaacaaggag

tcaagtcctatgagcactaatcgttttgatgaggtaaaaggaagagccgtgagattagctccagcttttgtggattatgt

tgggaatggatgtccttaagtttgttgattggccttctgggattttcattagtcggattgatttgaatctgcagtactta

aacaatccagggaataaactcgagagatagatcataatttcagacaaaagttttgactcaataaaatgactatctaagtt

aaaatgacacaactgactcataagaaaagaaaagatgaagttagtaactgactcccccagacttaggtaacattgtcccc

agagttattaagctagaggaatcgaaagagaaatagtcaaaagttaaaaagtcagtaaatgtgagttgccacctgagagt

ggtgtcgatcgataccatgatggtgtcgatcgacactctggtcatttcacattgctttgtgaattctgctcattgttgta

gagccttggttagtgttttcacatggatcctccagttagactgttaaatcactaaaacacacatcaatagttccaacaaa

gcataagaaaataagtgcaacaagttttaaaacaaataaccaatagtctgaaataaagcagaaacaaagttagaaaataa

aacacgataaggagggtggctagtcctcatgctctgagccggaggtgggtgtgggagagcgacgacgacgacgactgggg

gcaacacaaccgcagcgagaaagtgtagctatggcgttccagacggtcttgaagctgtcaacgacgtactgctgaaagac

ctgtggctccatcgggatgggtggaggaaccaagtgatgagcgaacacagcaccaaagttgacattacggtcaacttgct

caaatccaaggctttcaggaaagaagtcatgaagagcgtgatagagcagtaacaactcagagagtctcatcttgccaggc

ttcatcttgcacaacagtgtgctactaatcagcctaaccaaataccgtagaacagggtggcggatctctgagtgaactgc

gtccttggaatcataaatttcattaccaaaactgctctagaaaatctgagaatccttaaactgaccaggcagggaaactc

cattgagatcattgtcaaacccatatatgtcacccaaatctcgcagtggtaaactgtatctgacaccgcaaatgaagaag

ataagagctccttcctgaccgttgcgtttcctgtcatgcaaataagaaaccctaacagaggccatgatacagatcgtact

gcctacaacagatggttcccaaacttacgtcatctagcagccctaaaactgatttctctatctgcaaacgctgcatcaaa

ccgaaatccgcaaatcgagtaggcaaaatttatacattcatccaggcattatagaatagagtatcataatcatcccaatc

tggagaaaagggcgattgatatagtcacctttagtgaattccttgttgatatctttctcccaacccctttgagaaggaat

tctctcttttggatgctttggccatggaaatcgatgaataggaggttgaggggctgcagcttcggattccggttgagaag

ggctgcgaccgacggcggtggagaagcgtcggcggtgtggagggacggtggaggaggaggaaggtccgacgctcttccta

gccttctttttctggcctgtcatcttgtagatcgatggaattgatacttggggaagatagagggtgagattctattgagt

ttagagcctgaaatcacgagaaatggtgaagaatcgaaggatctatggaggttttagtgaattcggcggttttgagaaaa

atgaaatgaagaagatgaagttttaggttaaaacgacttacctgaaaaatgagggtgtctatcgacaccatcgcgttttt

ggtcgacacgctttttttatttttttttaatctgaatatttctataaaataaaaaaaatcctaagttagaaatcaaatga

atgaaatgaataaaaggagaaatacttaccagtgggttgcctcccactaagcgcttgtttatagtcaatagcttgacatt

gcagttgcttaattaacaggtgggtcctgaagatccaaaggcttttctactggaatctctgtccattcacagcaaatggt

ttgccattgttatttttgagaatgattgttccatatgacctaaccgagtgaactgtgaggggacctgtccaacgagaaga

caatttgccaggaaataatgtcaatttagaatcataaagaagtacctggtcattaggctcaaaggtccgagtaaggatct

tcttgtcgtggtagccttttgttcgttccttgtagagcttcgagttgtcataagcgtgaattcgtatttcatcaagctca

ttcagctgatttgatgtcgaaattcatcatcttgactgcccaagctgcttgctctaattccactggaagatgacaagctt

taccatatagaagatgaaaaggtgtgcttccaagcggagttttgaaagctgtcctgtaggcccacagtgcatcatctaac

ttgtaggatcactcctttttcgctttacccactgttttctcaagaatctctttgatttgcctgttagaaactacaacttg

gccgcttgtttgtggatggtagggggtagcaacccgatgctagactccatactgtaaaagcaatttcgcaagaatcttgt

taatgaaatgcttacctccatcactaatgactatgcgtggaactccaaaacgagggaagatgatagatttgaagagtttc

atgacaaccgttgaatcgttcttcggactagctatagctttctacccatttggagacataatccacagctactaggatgt

agagattcttgttggaaggtgggaatggtcccatgaaatctataccccaacaatcgaatacttcgacttcgagtataaat

ttctgaggcatctcattacgcttggtgatctttccccttctctgacaagggtcctattgtgaccagagtatcccgaaaca

ttgtaggccaccaaaagcctgcttgaagaactttggatactgttttgaaagttgcaaaatgaccaccatagctagagctg

tgacaatacgatagtatatcagggacctctgttgcagcaatgcatctcctgtaaattccatcagaacaatgcttatatag

ataaggctcatcccaatgatatcgcctaatctctcgtaggaatctattcttgttgtaatctgtaaaattatcaggctgca

catctgcagctaggtaattaacaatgtcagcataccatgggtaatctttctcgaccgttgtgacggtgcaattctcggtt

ggtgaccaatttcttgtcgatttagaagggtgtcgatcgacaggtgcaagggtgtcgatcgacaccatcgcgcaactaca

aatatcaacttcctctgaaatatgagtgtcgatcgtcataatgggtgtgtcgttcgacactgaagcacgatcctgcaacc

tgtcacgtttgtagtcatcgtcttcagctgtatcaatcatgtatatgttctcttcaggcaagaaatcatttatagggaca

tcatcatcgatcctaatgcgtgacaaatgatcagatacaccattctcaactcttttcttatctctaacttctatgtcaaa

ttcttgaagaagcaaaatccatctcaaaagtcttggcttagcatccttgttttgcatcaaatacttcaaggcagcatgat

ctgtgtgaacaataaccttggatccaacgagataagatctgaatttttcgaaagcaaacaccacagctaacaactctttc

tctgtagttgtataattcctttgagcatcatcaagagttctactttcatagtagattgcatgaagtttcttatccttcct

ctgtcctagaatagctccaactgcaaaatcacttgcatcacacattacctcaaaaggtaaatcccaatctggtggctgaa

caattggtgtgttgatcaaggcctgttttatctgctgaaatgcatcatgacacttctgtgtaaactcgaatttgacttct

ttacagagtaaagcagtcaatggtcttgcgattctgctgaagtctttgatgaatctcctataaaaaccagcatgtccaag

gaaattcctcactgctttgacattatccagcgcttgaagactcgtcatgacctcaatcttagctctatcaacttctatac

catgctcagaaatcatgtgtcctagaactattccatcctgtaccatgaagtgacatttctcccaaatcaaaactagatgt

ttctcctcacatcttgctaacactttgcagagattctctaaacaatcctcaaatgatgatccataaactgaaaagtcatc

catgaaaacctccatgatatcctcgatcatatctgtgaagatagacatcattcctctctgaaatgtcgcaggagcattac

aaagaccgaagggcattctccgataaacaaatgtaccataggggcaggtgaaagtcgttttctcctggtcatctggatga

atcgggatttgaaagaatccggagtatccatcaaggaagcaatagtacttatgatttgctaacctctccaacatctgacc

aataaatggtaaggcgaaatggtctttcctggtagcagcattcagctttctgtaatcaatgcacatccgatgtcatgtga

ttgtccgagtaggaattagctcgtctttctcattcttgactactgtgacacctccctttttagaaaccacatgaactgga

ctaacccaactcctatctgagattggatagataatccctgcttccaacagtttcataatctctttcgtgaccatttcttt

cagattcggattcaaccttctcttatgttcgactgaagacttagactcatcctcaaggtgaatcctatgcatgcatacat

cttgggatatacctgcaatgtcatcaagagagtagccaagagctttgcaatgattgcgcagcttgcttaggaataaggtg

agctctgcgggattaagagaaacattcacaataacaagataagtagaattttcactaagaaaagcgtaccttagccctgc

tggaagtggtttcaattcgattttaggagctttggctggatcccaatctgaagtaggagtcggcgttgaagtcaaagatg

gaagagcttcttcaactgtaagttgcatcacatgttcggtctcatctagcagcttagtatatgttgaagtttcacagtcc

aaatctgcactatcctctatggaagagaccaaaactcgcactagtggtttttttaattccatctctcaaatactatctga

aactgaggaggtgaaaggatctgttaagaaggttttcccttctatgatcggtttcttgaccaaagtgttcatatcgaatt

gcatagttagatcaccaacattcagccctatgctcccttgtcgaacatctatgattgctccagctgtgtgcaacaaggat

ctccctaggattaatggatccttagagcaggtccattgggagggccttaacagatccttaaccaaaatctttgagtaatt

ttgagaaaaaataaataaaaattttgattaaatgttaggatcgcacctaattttaggatttttgatgatgttaaggatcg

gatacgtggcatgttttcattggttgagcttttttgacaaaacaagtttttttcttctttgcctttttttttcatttatc

gatttctctctctctcttgaactcggctgtgcaattgaacgattgattatcgccggaagatcttcatgcttttatatgat

caaatcggaagagattatcgcccggaagtcgtctctaatccactgtgctcatcatctcaaccgacggtgctcaacgtcga

cgagtttggtgaacctaatcggagaggattgtgaatcatatcggagtcttgtttgaaaccatggtccagtctcatgataa

aggtatgattctatctttaagtttgtgtatgattctatctttaatttgggttggtagatgttattgaaattagagtttcg

tgaattgggaattcaagcaaaataaattagggctatttgatgtaaacgaattagggtttgtaagaatgcataatcagtct

ctttcttggttattagtgatgattgaagacgatttataggctgtagattgtttgttcacactcgattatgagatcaattt

tttatatgttttgtatgtagaatgtatttgtcatatgtctatcaattgaaatctctgaatattatgtctatgaattgtag

ttcgtacatctgaatttgagatgtgtttggattgtttttatttgaattaattgttaaggttttttatgttcctgtttctt

tatagtgaatgaattatttttatcttggtttggtcacaccgcattctttacttggtatgaaatcaggctcatcttggttt

gacgtgtgagattgtcaaggtatgaaatgcataatttaaatttacatgcttcagctttatgtactttttttttccaggtc

atatgtgctctgattctcatctttttcacaacattctttactttcataatactaaggtaacttttattttccttgttcag

atagtcttgtttttgatagaattctttcagttttgagtttttgaattagagaatgctttggattatggaatgctttgtat

ttacatgtttttttcttttagctgtaggtcgtcgacgacaacacttatagaagaccaacattatctcttttggtttgtca

ttgcaaatgtattttgatttgagagcacataacaatattcggacaacaacttctatgcaaatgaattattgtctttcatt

tttttttagaaacctacttttagaacttaccattggacaagaaaaaagtttaggattgtacaaaaattctaaacttcaaa

tttttcaaaatttattactaaaaaaatataaggattccattattaggactccttgatgggcatgctcttaggttccttct

cataaggcaagacgacaaagtttgtaggaatcatgctctttccaatcatcacgggaacatcaataagaattccatctgga

attcgggtagaacgatcagctaacacaagagtgataaaggtaggctcaagattggaatacccaagacgcatagcaactga

acgaggcatcagattcacactagaaccgagatcacataaagctcttgggaaggaatcctgattaatggtgacactaagta

caaaagttccaggatcaggtagcttcctcgggattcattcctgaataatgtcgcttccttgctatgaaatcatcataaca

atgcattctttcgtcaaattcttagtaaccattgtcttaacataccttatgattgtatgagatagatgaatagcttcaac

tagagacatctcaacaatcagcttatcaatcatagccttgcatctaacatcatctaaggcttgtcttgactttttaggac

tcttggggaagggaattcgccgatttgctgaattggttgcagttagaatttgcacattgtctaatcgaaccgggtgtcga

tcgacaccatcgggaattgtttcctcttcctccgcgaaattgtcgattccactcgacaaaggaaaaacagagtcgtcaag

agtaattggtgtcgatcgacaccctggtgtatcgatcgacactgatttgttttctgcaattggagaagattgatagtagg

attttagtcaattaatcctaaaacactatcctgtcgttgtagtattttaggatgtcaatccaatccggtgtgatgcaaac

agatgagatatgatcagaaagctctaagtcaagccaacaagttcatggggttatcgaatagtgtggtctccttaatctat

tcaggcggttgacaaaacttcatagaagcgatccctagacaacaagttcaacaacagattaattcactcccgtgatagaa

atcctcaagcaaagctagacactgacctaaatccctttagccatctctaactaagcaggcattattgaatcaacatgtta

aagccaataccctatctacagccaatcccttggggtagagtgagatatctctggaattaataggatccaacgcccatgaa

acgccttctgagcgctggacgtcgagactcatattccacattagctagtgaaacaagcaataagcgcaactaatccagaa

gggattctagtattctaaactcaaccacctaaaacgaccaagtaaaccacaactaatctatcccatcctcagaaatatag

ttcactactcagacataatcaaagacaatagcaaagcaacgattgagaacatgataacagaaatgatgaaagacttagac

gaaaactgaatatcaaatacttgaaagtgaaaatacaaagtcgcaggagaaacactgcgactatcaaagttgcaaaaatg

taaaagcgagataataacagataatagcgaatgccctaggaaaaaccttaagaggcagtttatataggcgagaaatgaaa

caattaaggaaactaaatatatagcgaatcttcacgtcatgatcctagaccgaatgtgtcattggcatctgacgggctga

aatgagctccctgtgatcgagtatgtcattgatcaaactgggcttcaggggctccgccttgcgatcgagtgagatgcagt

gaagtcgagattgaatggtccagcgaccctgtgatcgagtgcgatcgagtggatgtcatgaggtggtgatcgagtgattg

cttcggcttctattctcttcttcgttcttagcttgtctggttgagtgcagaacgagcagataggccttctggtgatcgag

tggtgatcgagtggttctgatggggtgattcctccggctttgcgattgagtgaaactgaggtgctggcttctccggggtt

gcgatagagtgatttgccttggcagggctccgaggctacgatagagtagttttgctaaaggcgtcattgggcattctgac

tttcttttctgtttctcctttgtttgaactccaaaagcatccaaagtacctgaaaacaaccaaatatgcaatgcacatgc

aatcctaatgcaaattcgtcctaagtatgcaaaacacactaaaacgactcctaatgagatgaaatgtggatgaaacaatg

acaaagtggtgaagaatgaatgcctaaatcatgcaaattatagacatatcaactcccccaaacttagtctttgcttgccc

tcaagcaaacaagaagacataagcggaaggagaggtttgaagttggggattcagaaccaaagcatgagatatgacaatta

agatcaatgtaccaactaacagttctaagatgttaggtgatcgacttctacttaaaaactttagccatgctctattatga

tccaaacctacactcaaatgcacaatgcgtcaagatcaaccaatccctttaacattcattaagtttgaaaacatgaatca

agatatgcatcgctagtgaactcatttggctaatgacaggttatgagacaaagatggtcccttttaagtggttcagactt

taatagcaggggactttcaatgaaaaaggactgagctttagaagaatgctaaaggtaagtgccaacctatgcatacaaga

atagcaccccatctacccaatgacataaaccgtgaataacaagtagatagtcctatctctaaacccaatgatgatcataa

gtctactcttttgcactaaaggcatattttgattttttcttggatcaatgtttccttttcttttctttggctcttgttct

taaggagaagcttttcttaaagtctatgagtctaatctagtgaattggttttacttaaactctaatggctcttatgattt

ttgtttttttctcttttcctttctgtttttattgccattttttctagagtatatcttatcaattctatgcttgccaaaca

ttcactagacctcgatatgattctcagcttccttctcctaaagattctaaccagttttcgtttttttttcttttcacact

caatctcaatctgcaaataagttcccacctactcctaccaagtccgaaaacgcggattggaactacatgagatactactc

ttgtcggctcaaacataacatgttctaccaagctcaatcctgaaaataggccttacatgctctcacaaaataacatggct

tgaaagaagggtttgttaagggtgggggaactactccaataatagaagcagctacttggattaacaagagtggtgaataa

gtgaaagaaaatccaaatatgtattctatctacgagttcaaaaacgatgcaaacatgatcattataagtcagattcaagt

tcaaattgacaggaaacggtgtcatgacatgcatcgagtggtttctataaaacatggtgcggttttacttcaaagtcgtt

caaatgcctcagagaactaataactcattttagtgaatcatcatgcttacaaggctattttctcattatcccctatgcat

atgcaacaatcctatatgaaacactctatacttgatcctaaatgtaatgcaactaaatgatcactctgtttttgtggaat

tcattttcaaaaatattcaaattttttttgttttttttctatgacttagatgatatgcagactcaaatgatattcacaaa

aaacaagtcaaatacgtcttgaaccctcccccaaacttcaattacacagtcccttgtgtaatcaaaatttgtgaaagaat

tcaagataaacacagaaacaaaaacaaaagcggtatttacaaagggttttggtgtgttacctcagtagaatgtcctagag

atgtagtcatccatatctgcttgagtgtactcagcaggcatgaccggttcttaatgtggctccgccatctcctcgtcatt

gtgctcgatcgcaaccccagcaggttgtgcagcacgacgctcatgtgttctccgacccgacatttctgacccttgcttga

tgaatgtacagttctagcactcttcctcctcttgtgttccattggctcatgagacgagtgccttgcgggaacttgcagaa

tggtgtgatccctttgttgataggtgaattgccttggtgggagaatgtcgtggcagctggatggcatgtcttcaggcggg

ttgtcattgaagttctgtcgggcattttcacctctccttgcgattgagtggtttgggcaattccactaggagaccactcg

atcacaccacttccatgttgcgatcgagtggtttgacattgtcgttgagaaagatgttgtcccaaaaccactagatcgca

ctcccgagcttttactctcgtcttgcgatagagtgattcatcaaaaatttgaatttttttatttgaacactcggttgcat

cactgctgttgctctagctgttatttatttcctgagaatcaaagccaaaaccaaaacaaatcaaaagtaacaaaataaaa

ttaaaactatatacagggataggcatgggacttcctcccaagtgagcttgttttaaatctctagcttgactcctcacact

cattaggctcaaggagggtgatagagtagaattgacgtctcctctccttccttagtagatacaacatcaaattcagcatg

ctctcgtgacaagtctgtaattgagtactcaatgcccctttcttggtaagctcctttttcatcatctggatagtcgtctt

ctctagactgttctaatgtgaattcacatccttctttactaaccataggaataacttcatcatttgggacgtccttgatg

tcgagtggtgcttggttggctttcccatagagatccttgacttgatccttcagctctttcatagtgtgtgttagctctct

aatagctttgtcgtgccatttagacctctccttgagttccaaccaatcatttgatgccctcatgcttgactttagctgtt

tggttgagcactctagtcgagtatcaattgagacttcctcgttcaatgcaacatcctctctttctaacgcaatgacacct

ttaaagatctctgatcctggtactttgttgtgtgagtctaatgacatctcatagctcaaggtctcactggagagaaatcc

agcttcattacttttggtcaaagcagtttgaaggggatctttgtcagtaagttcctctagcagttcattagctggttgac

ccatttcttcaactaagaaggtttgtccttctattgttggcttcttcataatgtccctgatttcaaacttcattttgaaa

tcctcccctagattaagattaatttttctttgcttaacatctatgactgctccagctgaagccaagaaaggtcttcctaa

gattagaggatctttagattcttcatttatctcaagcacaacaaaatcagtagggacctctactccattaatcattactt

gcacatcctctaacaggccaaaatgtctccttgaagtcctatcagcaagaatcaaggttaggtcacaaggcttgtactga

acgaatccgagcttccttgccatagagagtggcattatgctgactgaggctcctatatcacatagacaattgctgaaagt

caattgcctaatggagcatggtagggtaaaagatcctggatcttccaacttttcttgaataatcttctgctgagtgatgg

cactgcactcatgacttaatagcaccattccttggacttctttgatcttttctgtaatcaggtctttcacgtacttgtgc

tcgtcaggcatatcctttatgtgtttttccaggaggactctgtattttgcaaccaaaactttcttaaaacgaccagagaa

tggatggggaggtttgtaaggcggtggcacgaagactgtgtctttggtctttgcagcttcggatttttcaacaaagcgag

taggtgggggagcttgtggttgagtgggttgactggtgagtttgagctcttcgattgtgttgtcagccaaacaatcatct

tgataaaaatcctccccctctagaattacactgtcctcagtgtttgatgtagggacatatcgagttggcagctctcgatc

atgtctgatagtgatggcatgggcagtggcatactcctttggattctggattgctttctctggaagctggcataggttgt

tgttaacagatggtgaaacgaggatgccttccacacatctcatcctagtgctcattgattcgaacttgctgttaagttcg

acgccttgtcggtcgactttgttgtttacttcggacaacttcttagcgattttcattgctcttgtggcttgtccttgaat

gagctactggagcatagtcattatgtctgaattctgaggcacaggtgcttgttgttgctgtggtgtgaaaccaggttgtt

gttgttgttgttgatatcctccttaaaactactgctttggaatgaacccttggctttgtttgtaaggaacaaacagtttg

ggttggttctactgctgttgctgctgaggatagacttggtcctgagggttggcgacattggtgcttctgtttgacagatt

tggatagggtttgtaattgttttaccccatgttgtagccaccttgattctgaacatagctgatttcagcacactcatcat

tctctccctcttttacttggaatggttcatcttcagagataaagtgaacatgtttcttctgcacttggaggattttgtca

agtttgtcattgagagctttcatatctctgtggtgcttgtcatcagtgtcagaattggtgtggatacttctgtcatagtc

ttcattgtaattgccatccgaatgagaaaagttttcaactagctcccatccttcttcaacatccttcttcaaaaaattcc

catttgaagcggtgtcaagcagcatccttatcttaggcaagacgcctctatagagtgtgttgagaagagaagcttgctta
[truncated: 273,538 more chars]
